# An Expedient Method for the Umpolung Coupling of Enols with Heteronucleophiles**

**DOI:** 10.1002/chem.202201000

**Published:** 2022-06-17

**Authors:** Víctor García‐Vázquez, Alba Carretero Cerdán, Amparo Sanz‐Marco, Enrique Gómez‐Bengoa, Belén Martín‐Matute

**Affiliations:** ^1^ Department of Organic Chemistry Stockholm University Stockholm 10691 Sweden; ^2^ Departamento de Química Orgánica I Universidad Pais Vasco, UPV/EHU 20080 Donostia-San Sebastián Spain

**Keywords:** DFT, enol derivatives, hypervalent iodine(III), mechanistic insight, umpolung

## Abstract

In this paper, we present an unprecedented and general umpolung protocol that allows the functionalization of silyl enol ethers and of 1,3‐dicarbonyl compounds with a large range of heteroatom nucleophiles, including carboxylic acids, alcohols, primary and secondary amines, azide, thiols, and also anionic carbamates derived from CO_2_. The scope of the reaction also extends to carbon‐based nucleophiles. The reaction relies on the use of 1‐bromo‐3,3‐dimethyl‐1,3‐dihydro‐1λ^3^[*d*][1,2]iodaoxole, which provides a key α‐brominated carbonyl intermediate. The reaction mechanism has been studied experimentally and by DFT, and we propose formation of an unusual enolonium intermediate with a halogen‐bonded bromide.

## Graphical abstract

**A useful strategy**: Herein, we report an umpolung strategy mediated by a hypervalent iodine(III) reagent for the functionalization of enol derivatives with different heteronucleophiles including carboxylic acids, thiols, alcohols, primary and secondary amines, azides and carbamates generated from CO_2_. Relevant examples for the derivatization of natural products and pharmaceutical compounds are also presented. The mechanism of the reaction was investigated by DFT calculations and experimental studies.

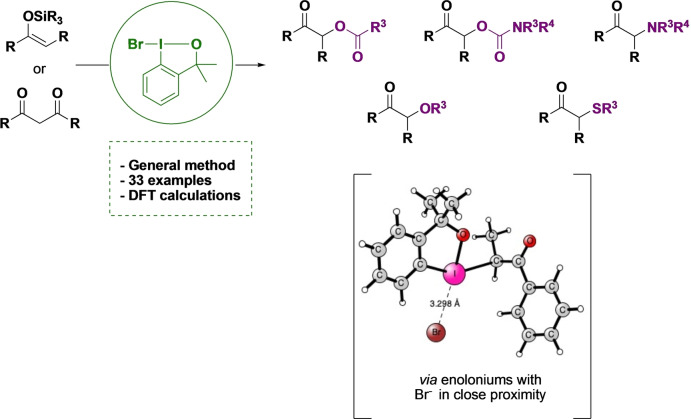

## Introduction

The introduction of functional groups at the α carbon of carbonyl compounds is a common transformation in synthetic organic chemistry. α‐Functionalized ketones are substructures found in many natural products, pharmaceuticals, and other functional organic compounds.^[1]^ The functionalization reaction relies on the inherent nucleophilicity of the α carbon of the enol (or enolate) derivative of the carbonyl compound, which reacts with an electrophilic reaction partner in this process. Many carbon‐based electrophiles can be used, leading to the formation of C−C bonds,^[2]^ but the use of heteroatom electrophiles becomes challenging. This is due to the high reactivity, and therefore limited functional‐group compatibility of these species. They are typically strong oxidants, and this can lead to the formation of by‐products, such as overfunctionalized or oxidized compounds.^[3]^ The structural variety of these species is also limited, and it is difficult to reconcile this with the idea of producing structurally diverse target compounds. Nevertheless, there are a number of heteroatom electrophiles that can be used in such reactions, designed for specific transformations and with specific functional‐group tolerances.^[4]^ Alternatively, organocatalytic methods have overcome some of these difficulties.^[5]^

**Figure 1:**
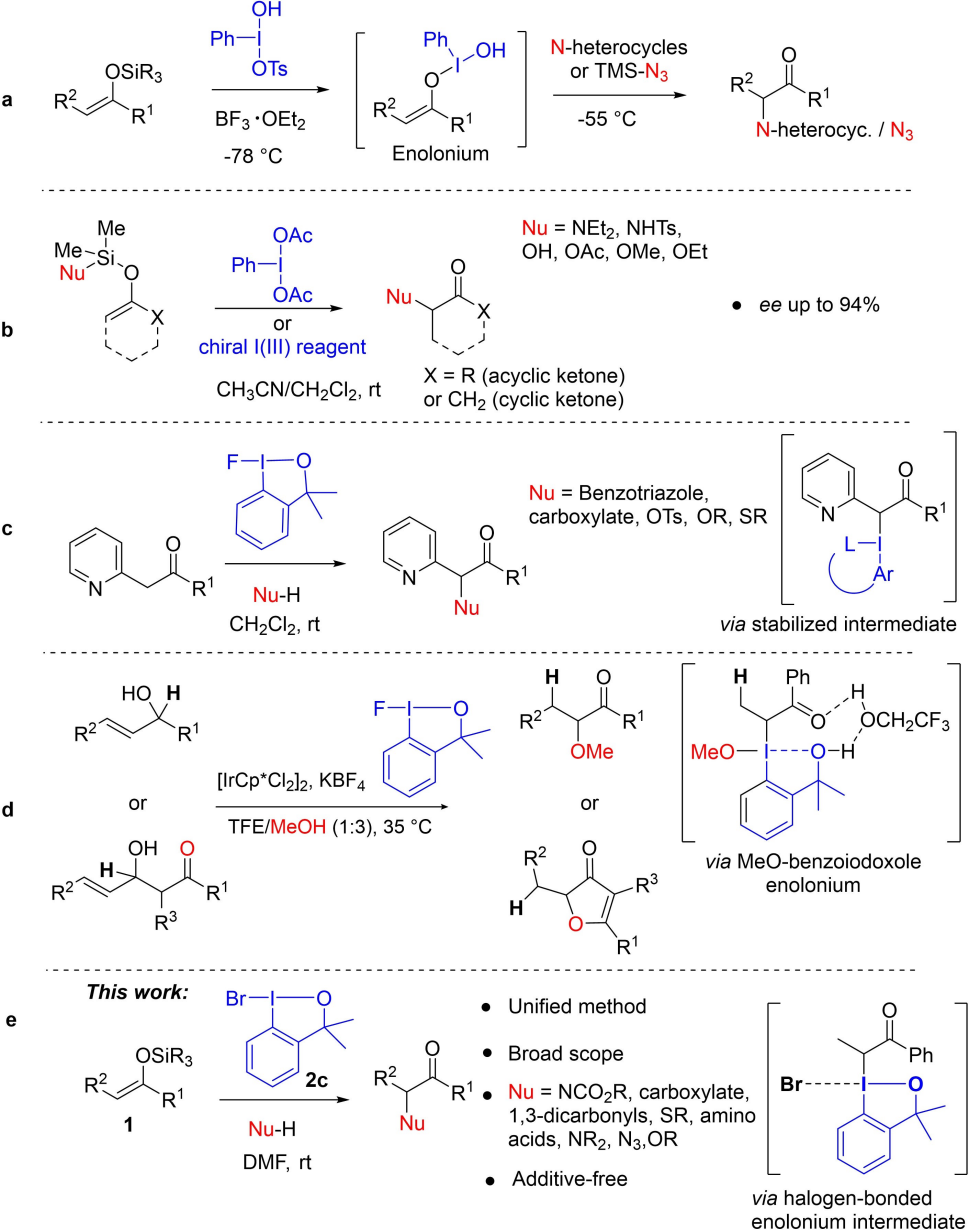
Strategies for the umpolung α‐functionalization of ketones and enol derivatives. a) Cross‐nucleophile coupling of silyl enol ethers with nitrogen nucleophiles.^[8c]^ b) Intramolecular cross‐nucleophile coupling of nucleophile‐functionalized silyl enol ethers.^[8a]^ c) Cross‐nucleophile coupling of pyridyl ketones.^[14]^ d) Cross‐coupling of allylic alcohols with nucleophiles.^[16]^ e) This work: general method for the cross‐nucleophile coupling of silyl enol ethers with nucleophiles.

An alternative approach is to use nucleophiles rather than electrophiles to react with enol derivatives. Iodine(III) compounds have been used in this context to mediate the coupling of the two nucleophilic reactants through two‐electron oxidations, thereby inverting the polarity of one of the reagents.^[6]^ This strategy has recently been termed “cross‐nucleophile coupling”.^[7]^ This area has evolved significantly^[8]^ since the first report.^[9]^ When it comes to heteroatom nucleophiles, the reaction usually requires a Lewis acid, as well as a low reaction temperature, in a one‐pot two‐step procedure, to avoid formation of by‐products (Figure 1a).^[10]^ First the I(III) reagent and the enol derivative react at low temperature to form an enolonium intermediate.^[10a]^ This ensures that the enol nucleophile is consumed before the second nucleophile is added at higher temperature (Figure 1a). In this way, side reactions such as homocoupling of the enol derivative or α‐functionalization with other nucleophiles derived from the I(III) reagent (e. g., OAc) are minimized.^[10a]^ Using this protocol, Szpilman *et. al* elegantly observed *O*‐enolonium species for the first time using ^13^C NMR spectroscopy.^[10a]^ The formation of by‐products is closely related to the outstanding leaving ability of the I(III) functional group (10^6^ better than triflate).^[11]^

In general, the nature of the second nucleophile is somewhat limited, but excellent results have been reported for arylations,[^10b^, ^10c^, ^10e^, ^12^] azidations,^[8c]^ cyanations,^[8b]^ and acetoxylations.[^8d^, ^9^, ^13^] The somewhat narrow scope is partly due to the fact that the nucleophile may need to be incorporated into the structure of the I(III) reagent.[^8b^, ^12^, ^13^, ^14^] General methods for the intermolecular reaction of ketones or enol derivatives with a variety of nucleophiles, using a non‐designer I(III) reagent, are scarce. The Wirth group developed an effective approach to the formation of nitrogen‐ and oxygen‐α‐substituted ketones through an internal umpolung strategy mediated by PhI(OAc)_2_ (Figure 1b).^[8a]^ Their strategy relied on the use of a tethered nucleophile, i. e., the nucleophile was attached to the silicon center of the enol ether substrate (Figure 1b). Importantly, they were able to extend the scope of the reaction to the synthesis of chiral α‐substituted ketones when using chiral I(III) reagents. More recently, the Gulder group reported another elegant approach aimed at expanding the range of nucleophiles that can be used in such reactions. Here, 2‐pyridyl ketones react by an umpolung coupling process mediated by a λ^3^‐fluoro iodane (Figure 1c).^[15]^ It was proposed that a noncovalent interaction between the F atom in the iodane and the pyridine moiety in the ketone substrate plays a key role in this reaction. Although this reaction is quite limited in terms of the ketone structure, a large number of nucleophiles could be coupled.

Considering the ketone component, the majority of reported examples, with the exception of the pyridyl ketones used by Gulder,^[15]^ rely on the use of silyl enol ethers.[^8a^, ^8b^, ^8c^, ^8e^, ^10a^, ^10b^, ^10e^] Our own group contributed to this area of research with an umpolung protocol using allylic alcohols as enol synthons, in a reaction mediated by iridium catalysts (Figure 1d). This method gave α‐methoxy ketones from allylic alcohols, or 3(*2H*)‐furanones from carbonyl‐functionalized allylic alcohols. For all the examples, 1‐fluoro‐3,3‐dimethyl‐1,3‐dihydro‐1λ^3^‐benzo[*d*][1,2]iodaoxole was used as an oxidant.^[16]^

In this paper, we report the results of our investigations into the development of a method for the general reaction of unbiased silyl enol ethers (**1**) with a wide variety of heteronucleophiles, including carboxylic acids, thiols, alcohols, amines, azides, and even CO_2_ in the form of carbamate anions for the first time. Conveniently, in all instances, the same I(III) reagent is used. Thus the synthesis of substrate‐specific iodanes is avoided, which contributes to the generality and applicability of the method. The mechanism of the reaction has been studied experimentally and by DFT calculations, and an unusual enolonium intermediate with a halogen‐bonded bromide atom is proposed. From this enolonium, an α‐brominated carbonyl intermediate is formed, which is key for the high efficiency and the broad scope of the umpolung reaction.

## Results and Discussion

**Scheme 1:**
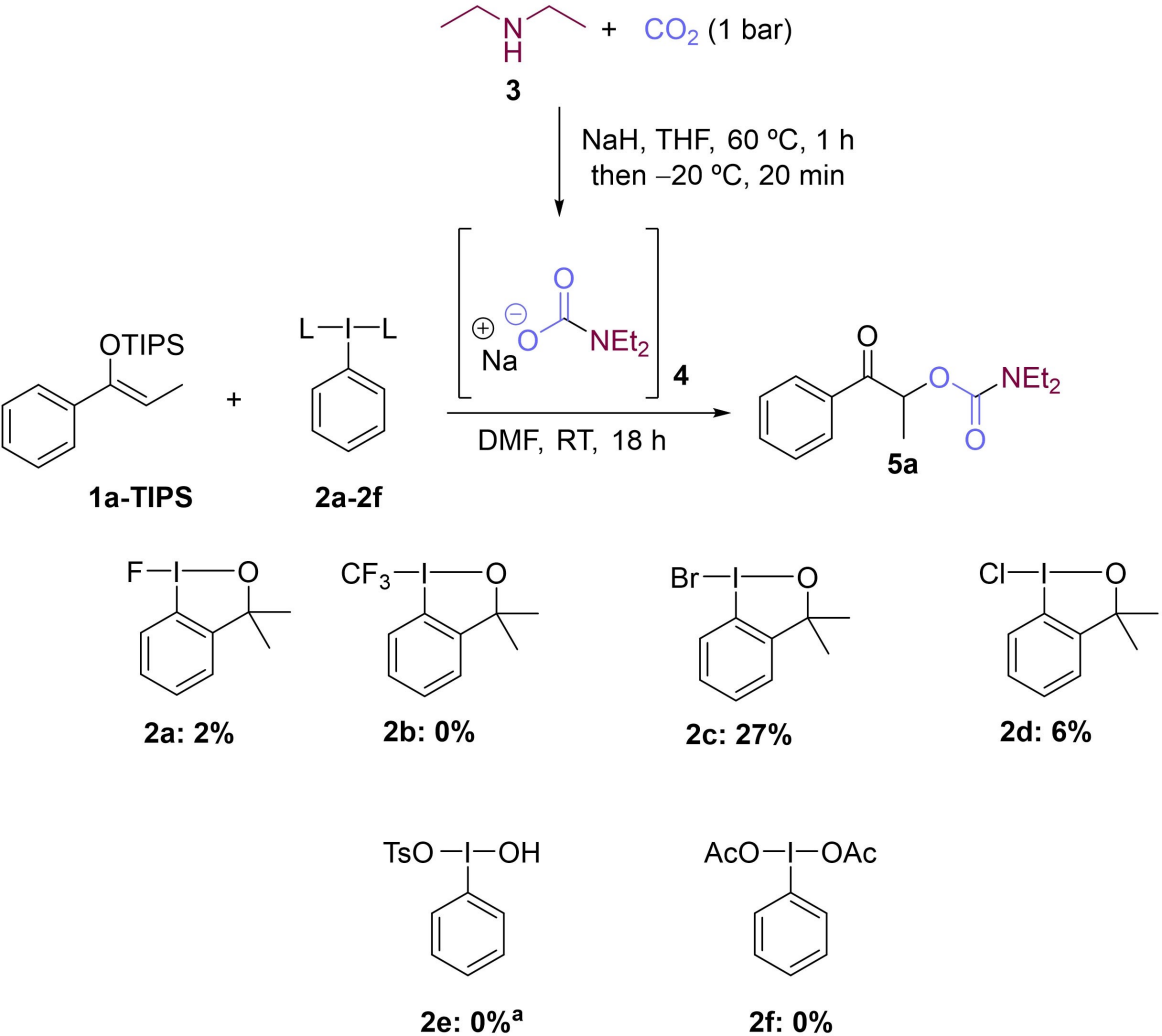
Screening of hypervalent iodine(III) reagents. Reaction conditions: **1 a‐TIPS** (0.1 mmol, 1 equiv.), **3** (0.2 mmol, 2 equiv.), iodine(III) reagent **2** (0.12 mmol, 1.2 equiv.), NaH (0.3 mmol, 3 equiv.), DMF (0.33 M), RT, CO_2_ (1 bar), 18 h. Yields determined by ^1^H NMR spectroscopy using 2,3,5,6‐tetrachlorobenzene as internal standard. ^a^Various products observed.

Initially, we focused on the coupling of silyl ethers (**1**) and CO_2_ derivatives, by using carbamates formed in situ from amines (**3**) and CO_2_. This umpolung strategy would give access to α‐carbamoyl carbonyl compounds, which are important scaffolds in medicinal chemistry.^[17]^ Reported methods for the synthesis of α‐carbamoyl carbonyl compounds from CO_2_ are very scarce, and typically require the use of high pressures of CO_2_ and high temperatures.^[18]^ We started by generating carbamate anions by treating amine **3** with NaH under 1 atm of CO_2_, a modification of a procedure described by Trost for the synthesis of carbonates.^[19]^ A variety of hypervalent iodine reagents (**2 a**–**f**) were tested (Scheme 1) for the coupling of silyl enol ether **1 a**‐TIPS with carbamate **4**. Surprisingly, only those benzoiodoxoles bearing a halide atom on the I(III) (**2 a**, **2 c**, and **2 d**) yielded some amounts of α‐carbamoyl carbonyl product **5 a**. Of these, it was 1‐bromo‐3,3‐dimethyl‐1,3‐dihydro‐1λ^3^[*d*][1,2]iodaoxole (**2 c**) that gave the best result (27 % yield of **5 a**). Togni reagent **2 b**, commonly used in radical additions of CF_3_,^[20]^ left the starting silyl enol ether **1 a**‐TIPS untouched. The use of Koser's reagent **2 e** led to the formation of by‐products, and with phenyl‐λ3‐iodanediyl diacetate (PIDA) (**2 f**) again the starting enol ether **1 a**‐TIPS was recovered.

Further optimizations were carried out with reagent **2 c** (Table 1). When the number of equiv. of NaH was lowered to 1.5, the yield increased substantially (59 %, Table 1, entry 3 vs. entries 1–2). Lowering the amount of NaH further, or lowering the amount of amine **3** did not have a significant effect on the yield (Table 1, entries 4 and 5). On the other hand, with 1.5 equiv. of **2 c**, a yield of 68 % was obtained (Table 1, entry 6). Importantly, when the triisopropylsilyl group (TIPS, **1 a**‐TIPS) was replaced by a *tert*‐butyldimethylsilyl group (**1 a**‐TBS, Table 1, entry 7), product **5 a** was obtained in 77 % yield. The less hindered trimethylsilyl group (**1 a**‐TMS, Table 1, entry 8) gave a lower yield. We also tested toluene, THF, 2‐methyltetrahydrofuran, and acetone as reaction solvents, but the desired product was not observed (Table 1, entry 9).

**Table 1:** Optimization studies.^[a]^

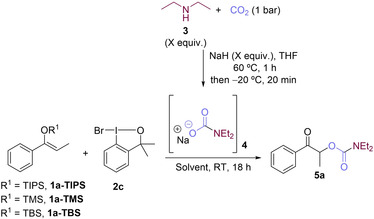					
Entry^[a]^	R^1^	NaH [equiv.]	**3** [equiv.]	**2 c** [equiv.]	Yield of **5 a** [%]^[b]^
1	TIPS	3	2	1.2	27
2	TIPS	2	2	1.2	43
3	TIPS	1.5	2	1.2	59
4	TIPS	1	2	1.2	51
5	TIPS	1.5	1.5	1.2	43
6	TIPS	1.5	2	1.5	68
7	TBS	1.5	2	1.5	77
8	TMS	1.5	2	1.5	52
9^[c]^	TBS	1.5	2	1.5	–

[a] Reaction conditions: **1** (0.1 mmol, 1 equiv.) DMF (0.33 M), RT, CO_2_ (1 bar). [b] Yields determined by ^1^H NMR spectroscopy using 2,3,5,6‐tetrachlorobenzene (0.1 mmol, 1 equiv.) as an internal standard. [c] In toluene, THF, 2‐methyltetrahydrofuran, or acetone (0.33 M).

**Scheme 2:**
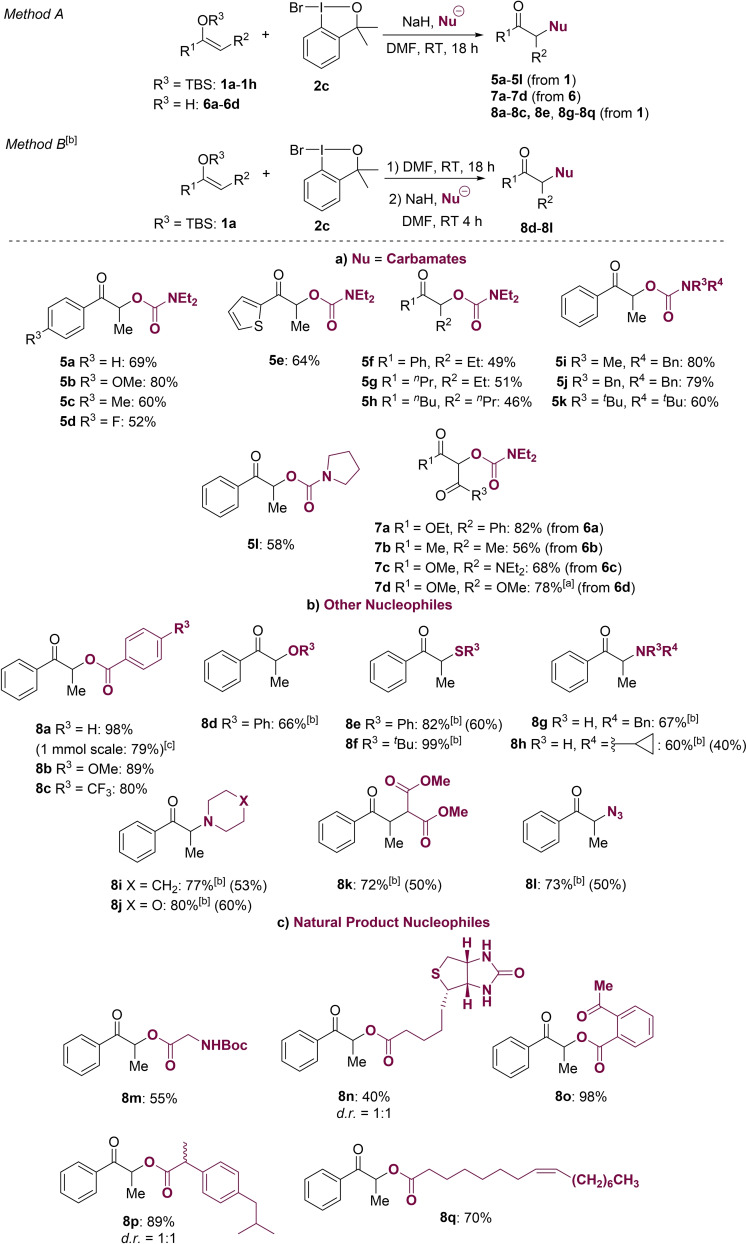
Substrate scope. Method A: **1 a**–**1 h** or **6 a**–**6 d** (0.1 mmol, 1 equiv.), **2 c** (0.15 mmol, 1.5 equiv.), NaH (0.15 mmol, 1.5 equiv.), nucleophile (0.2 mmol, 2 equiv.), DMF (0.33 M or 0.1 M), RT, 18 h. Isolated yields. [a]:1‐bromo‐3,3‐bis(trifluoromethyl)‐1,3‐dihydro‐1λ^3^‐benzo[*d*][1,2]iodaxole (2c'). [b]: Method B: **1 a** (0.1 mmol, 1 equiv.), **2 c** (0.15 mmol, 1.5 equiv.), DMF (0.1 M), RT, 18 h. After completion, NaH (0.15 mmol, 1.5 equiv.) and nucleophile (0.2 mmol, 2 equiv.) were added. [c]: 1 mmol scale.

We then applied the optimal conditions (Table 1, entry 7) to a number of TBS‐enol ethers (Scheme 2a). With electron‐donating groups at the *para* position of the aryl group at R^1^, the corresponding carbamates **5 b** and **5 c** were obtained with high efficiency (80 % and 60 % yields, respectively). Also, F‐substituted silyl enol ether **2 d** gave **5 d** in 52 % yield. Thiophene **1 e** gave the corresponding carbamate **5 e** in a good yield of 64 %. Unsuccessful examples are shown in Scheme S5. Thus, with an ethyl group at R^2^, **5 f** was obtained in a lower yield (49 %). For aliphatic *tert*‐butyldimethylsilyl enol ethers **1 g** and **1 h**, yields of 51 % and 46 %, respectively, were obtained. To assess the generality of the reaction, we tested different secondary amines to form the carbamate. Symmetrically and unsymmetrically substituted dialkyl amines reacted smoothly to give good yields (**5 i**–**5 k**, 60 %–80 %). Moreover, pyrrolidine substituted carbamate **5 l** was also obtained under the reaction conditions in 58 % yield. However, carbamates derived from primary amines gave a mixture of unidentified products (not shown), which represents a major limitation of this approach. Significantly, this umpolung strategy is not limited to silyl enol ethers, but could be extended to the use of 1,3‐dicarbonyl compounds. A β‐ketoester **6 a** reacted smoothly under the same conditions to give **7 a** in 82 % yield. 1,3‐Dicarbonyl compound **6 b** reacted with carbamate **4** to give **7 b** in good yield (56 %). The less nucleophilic β‐amidoester **6 c** and malonate **6 d** gave carbamoyl derivatives **7 c** and **7 d** in good yields (68 % and 78 %, respectively).

We went on to examine the generality of the cross‐nucleophile coupling of silyl enol ethers with a variety of other nucleophiles (Scheme 2b and c). Using both electron‐poor and electron‐rich benzoic acids as nucleophiles, α‐carboxylate‐carbonyl compounds **8 a**–**8 c** were obtained in excellent yields (80 %–98 %). When alcohols were tested as nucleophiles under otherwise identical reaction conditions, complex mixtures of unidentified by‐products were formed. However, these difficulties were overcome by modifying the protocol; first silyl enol ether **1 a** was treated with I(III) reagent **2 c**. This was followed by the addition of the alcohol nucleophile (Method B). Using this procedure, α‐phenolate **8 d** was obtained in 66 % yield. Thiols were also well tolerated, and thiophenol gave **8 e** in 60 % yield using the standard procedure described above (i. e., Method A), and in 82 % yield using Method B. An alkyl‐substituted thiol gave a quantitative yield (**8 f**, Method B). Importantly, primary amines are also well tolerated, and benzylamine derivative **8 g** was obtained in 67 % yield when using Method B. Cyclopropylamine reacted smoothly to give **8 h** in 40 % yield, which could be improved to 60 % by using Method B. Piperidine gave α‐aminoketone **8 i** in 53 % yield, and morpholine derivative **8 j** was obtained in 60 % yield. Interestingly, carbon nucleophiles such as malonates also reacted smoothly, and **8 k** was formed in 50 % yield. α‐Azido carbonyl compound **8 l** was obtained in 50 % isolated yield.

We went on to test a number of natural products and pharmaceuticals as nucleophiles in the reaction with **1 a**‐TBS. These compounds all contained carboxylic acid moieties in their structures (Scheme 2c). A BOC‐protected glycine derivative gave **8 m** in 55 % yield. With biotin as the nucleophile, compound **8 n** was obtained in 44 % yield. Acetyl salicylic acid gave **8 o** in quantitative yield (98 % isolated yield), and ibuprofen gave **8 p** in 87 % yield. With the aliphatic oleic acid, **8 q** was formed in 70 % yield.

**Scheme 3:**
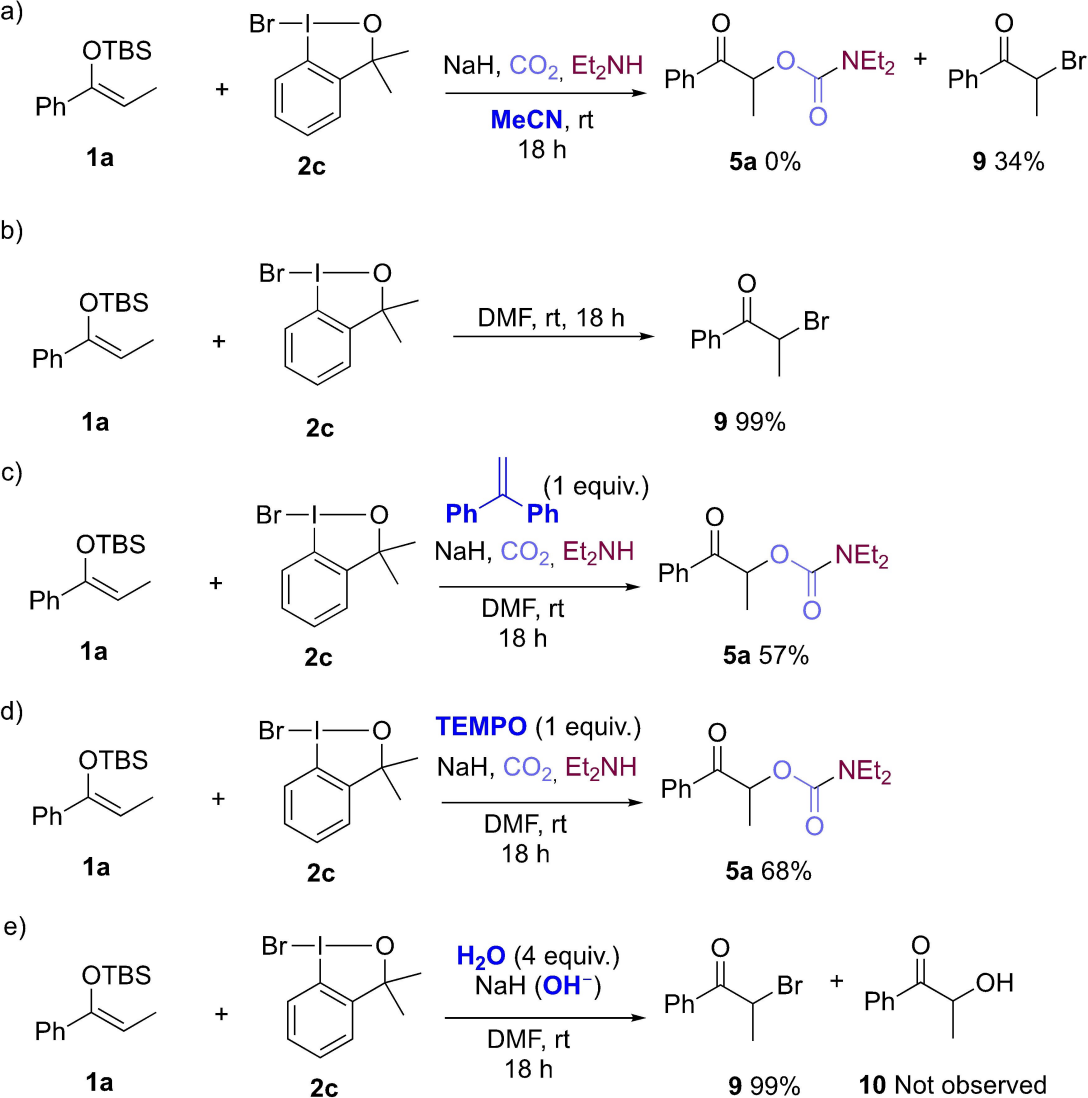
Control experiments and mechanistic investigations.

Next, we focused our attention on studying the mechanism of the reaction. In an attempt to understand the dramatic effect of DMF as the reaction solvent, a variety of other polar aprotic solvents were tested. When the reaction of **1 a**, CO_2_, and diethylamine with **2 c** was run in MeCN, 2‐bromo‐1‐phenylpropan‐1‐one (**9**) was detected as the sole product (Scheme 3a). α‐Bromo carbonyl compound **9** was also formed from **1 a** and **2 c** when the reaction was run in DMF in the absence of the nucleophile, in quantitative yield (Scheme S2b). We found that α‐bromo carbonyl derivative **9** reacted with the carbamate anion to give **5 a** in quantitative yield (Scheme S3). Therefore, it is reasonable to suggest that the reaction might proceed by umpolung bromination followed by a nucleophilic substitution step with the second nucleophile, in this instance the carbamate generated from CO_2_. The formation of α‐substituted carbonyl derivatives as intermediates that can react with nucleophiles in S_N_2‐type reactions has been previously studied by Maulide^[21]^ and Jorgensen^[22]^ among others,^[23]^ as a way to circumvent the inconvenience of using electrophilic reactants. Therefore, **2 c** seems to be an unusually mild brominating agent for the bromination of silyl enol ethers and 1,3‐dicarbonyl compounds. Other brominating agents such as NBS or Br_2_ lead to formation of polybrominated products in their reactions with silyl enol ethers.^[**24**]^ Moreover, the low compatibility of NBS or Br_2_ with functional groups such as alkynes, alkenes, and electron‐rich aromatics hinders their use in the direct α‐functionalization of enol derivatives with nucleophiles in a single umpolung step, resulting in a limited scope, and requiring multiple purification steps.^[21]^ Not even with simple unfunctionalized substrates these brominating agents afforded results comparable to those obtained with **2 c** (Scheme S4). Therefore, the use of the mild brominating agent **2 c** is key for this successful umpolung coupling. Note also that **2 c** is compatible with unsaturated functional groups, such as the double bond in **8 q**.

In the presence of radical scavengers, silyl enol ether **1 a** gave **5 a** in yields similar to those obtained in their absence (Scheme 3c and d vs. Scheme 2), suggesting a non‐radical pathway. We then tested the selectivity of the reaction towards the formation of α‐bromoketone **9** by adding OH^−^, as another potential nucleophile, to the reaction mixture (Scheme 3e). It has been shown before that water is able to displace I(III) in the enolonium intermediates, and form α‐hydroxy ketones (i. e., **10**). However, when adding a large excess of OH^−^, under otherwise identical reactions conditions, only α‐bromoketone **9** was formed in 99 % yield.

**Figure 2:**
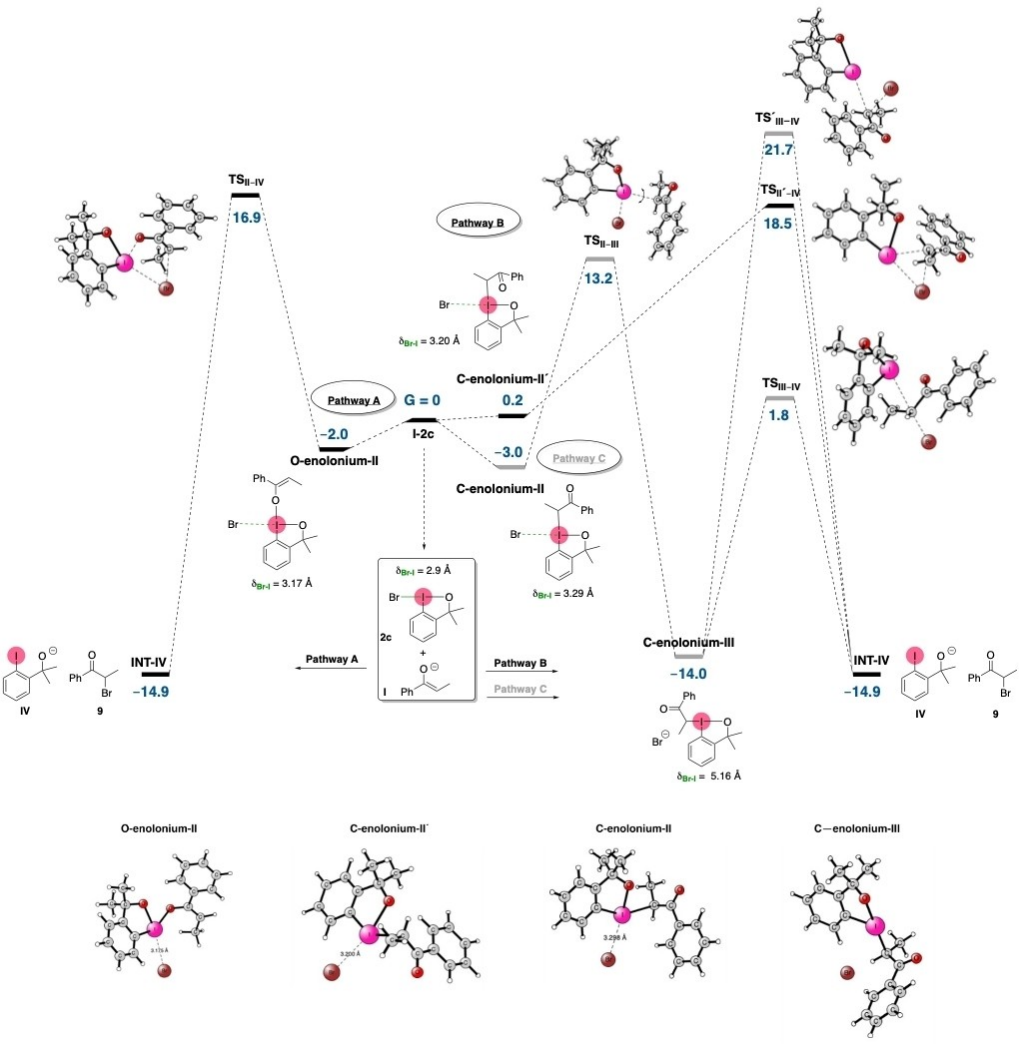
Top: Reaction mechanisms for the umpolung cross‐nucleophile coupling of enol derivatives mediated by hypervalent iodine(III) reagent **2 c**. Values are given in kcal/mol. Bottom: 3‐D Structures of enolonium intermediates.

To further understand the mechanism of the cross‐nucleophile‐coupling reaction mediated by bromobenzoidoxole **2 c**, we turned to DFT calculations at the B3LYP^[25]^ and M06^[26]^ functional levels using the Gaussian 16 software^[27]^ (see Supporting Information for more details). The calculations were initiated from enolate **I** as the model substrate. Formation of **I** from silyl enol ether **1 c** is likely to occur under basic conditions and in DMF, in agreement with previous reports^[28]^ Additionally, oxygen‐oxygen silyl transfer reactions have also been observed in intramolecular process,^[29]^ so it is reasonable to propose that a similar process can be operating between **1 a** and the oxygen atom of **2 c** (see Scheme S6). Therefore, the calculations were carried out using the sum of the energies of enolate species^[12]^ **I** and iodine(III) reagent **2 c** as the reference point (G=0 kcal/mol) of the energy profile (Figure 2, middle). The enolate can attack the iodine atom either through the oxygen or through the carbon of the enolate, leading to formation of at least three possible low‐energy enolonium intermediates (**O‐enolonium‐II** (‐2.0 kcal/mol), **C‐enolonium‐II** (−3.0 kcal/mol), and **C‐enolonium‐II'** (0.2 kcal/mol). In all cases, the formation of the enolate‐iodine bond occurs in the position *trans* to the phenyl‐I substituent, inducing an elongation of the Br−I bond. In the starting compound **2 c**, the Br−I bond is short, i. e., 2.9 Å. This bond length is increased in **O‐enolonium‐II** (3.17 Å), and even more so in **C‐enolonium‐II'** (3.20 Å) and **C‐enolonium‐II** (3.29 Å). These distances are similar to those found in structures with a halogen bond between iodine and bromine atoms.^[30]^ The three calculated intermediates are almost isoenergetic and they could, therefore, be in equilibrium. The direct reductive ligand coupling of the bromine and enolate fragments of these enolonium intermediates was calculated to be feasible. For example, **TS_II–IV_** and **TS_II'–IV_** have calculated activation energies of 18–19 kcal/mol from their respective enolonium intermediates, **O‐enolonium‐II** (‐2.0 kcal/mol) and **C‐enolonium‐II'** (0.2 kcal/mol). Interestingly, a transition state was found for the isomerization of the enolate substituent, which rotates from the *trans* to the *cis* position relative to the phenyl‐I bond, displacing the bromine atom from the coordination sphere of the iodine. This transition state (**TS_II–III_**, ΔΔ*G* ^≠^=16.2 kcal/mol) is low in energy, and leads to enolonium intermediate **C‐enolonium‐III**. From here, backside attack by the bromide (**TS_III–IV_**) on the α‐carbon in **C‐enolonium‐III** has the lowest calculated energy for C−Br formation (ΔΔ*G* ^≠^=15.8 kcal/mol). In contrast, the *syn* attack represented by **TS^'^**_**III**_‐_**IV**_ is too high energy to take place under these reaction conditions (ΔΔ*G* ^≠^=35.7 kcal/mol). Thus, our DFT calculations show that the formation of the α‐bromo ketone intermediate **INT‐IV** is feasible under the reaction conditions starting from the enolate and iodine(III) reagent **2 c**. This pathway is possible in the absence of any external nucleophilic anion. After that, intermediate α‐bromoketone **9** evolves via a nucleophilic displacement upon reaction with the heteronucleophiles (Scheme S2).

Both DFT calculations and experimental studies support the formation of α‐bromoketone **9** as an important intermediate in this cross‐nucleophile coupling. This umpolung event is mediated by benzoidoxole **2 c**, and can occur by three possible mechanisms. Pathway A and pathway B are analogous mechanisms involving **O‐enolonium‐II** and **C‐enolonium‐II’**, respectively, in which bromide interacts with the I(III) through a halogen bond (3.17 and 3.20 Å, respectively).^[28b]^ These species then undergo a reductive coupling step to give α‐bromoketone **9**. These Br−I interactions might explain the lack of by‐products. As mentioned above, the formation of by‐products such as dimers (Nu^2^=**1**) or α‐hydroxy ketones (Nu^2^=OH^−^, Scheme 3e) has been a major limitation in similar I(III)‐mediated umpolung approaches. We have also observed by‐product formation when using other I(III) reagents, for example, with **2 a** in our previous work,^[16]^ and with **2 e** in the optimization experiments described in this paper (Scheme 1). Alternatively, pathway C can occur via **C‐enolonium‐III**, which is formed by ligand rearrangement around the I(III) center of **C‐enolonium‐II**. In pathway C, the bromide atom is not interacting with the I(III) center (Br−I distance is 5.16 Å) of **C‐enolonium‐III**. From here, a nucleophilic attack of the Br^−^ on the very electrophilic^[11]^ α‐C of **C‐enolonium‐III** also leads to α‐bromoketone **9** (16.2 kcal/mol for isomerization and 15.8 kcal/mol for the S_N_2 step). The DFT calculations do not show a strong preference for any of the three pathways explored. However, if **C‐enolonium‐III** is involved (pathway C), we would expect the highly electrophilic enolonium^[11]^ (**C‐enolonium**‐**III**) to react also with any of the nucleophiles present in the reaction mixture, such as starting enol **1** or HO^−^ (Scheme 3e) to form by‐products. The control experiment in Scheme 3b also shows that α‐bromoketone **9** is the sole product formed in the absence of the heteronucleophile (Nu^2^). The absence of by‐products may be explained through a reaction pathway in which an intramolecular reaction results in formation of the C−Br bond formation (pathways A and B), rather than through an intermolecular nucleophilic displacement of I(III) on the enolonium intermediates (pathway C).

## Conclusion

We have developed an umpolung method for the cross‐nucleophile coupling of enol derivatives with a variety of nucleophiles, using a single iodine(III) reagent. The reactions occur with good efficiency, and by‐products are not formed. Using the method described here, CO_2_ has been used, in the form of anionic carbamates, in their reaction with silyl enol ethers for the first time. Additionally, benzoic acids, alcohols, thiols, primary and secondary amines, malonate and an azide have been used as nucleophiles in the transformation. We have also used this approach to derivatize natural products and drugs. Compound **2 c** has been demonstrated to be an efficient mild brominating agent that allows the umpolung functionalization of enol derivatives in a single step, with higher group functional tolerance than most common electrophilic brominating reagents. Mechanistic evidence and DFT calculations have shown that α‐bromoketones are formed as reaction intermediates. DFT calculations indicate that the mechanism may proceed via enolonium species containing I−Br halogen bonds. This may be key for the generality and high selectivity of the reaction.

## Experimental Section

### General procedures for the cross‐nucleophile‐coupling reaction

Method A: A solution of the nucleophile (0.2 mmol, 2 equiv.) in DMF (0.33 mL or 1 mL, 0.33 M or 0.1 M of **1**) was added to a vial containing **1** (0.1 mmol, 1 equiv.), NaH (0.15 mmol, 1.5 equiv.), and **2 c** (0.15 mmol, 1.5 equiv.). The reaction mixture was stirred at room temperature for 18 h. After this time, the mixture was extracted with EtOAc (3 × 5 mL). The organic phases were combined and washed with water three times. The organic phase was then dried (MgSO_4_), and the solvent was evaporated under reduced pressure. The residue was purified by flash column chromatography using silica gel as the stationary phase, eluting with a pentane/EtOAc mixture (5 to 100 % EtOAc), to give the desired products.

Method B: DMF (0.5 mL, 0.2 M) was added to a vial containing **1** (0.1 mmol, 1 equiv.) and **2 c** (0.15 mmol, 1.5 equiv.). The reaction mixture was stirred at room temperature for 18 h. After this time, the formation of **9** was complete. A solution of the nucleophile (0.2 mmol, 2 equiv.) and NaH (0.15 mmol, 1.5 equiv.) in DMF (0.5 mL, to reach 0.1 M of **1**) was added to the mixture. The reaction mixture was stirred at room temperature for a further 4 h. After this time, the mixture was extracted with EtOAc (3 × 5 mL), and the organic phases were combined and washed with water several times. The organic phase was then dried (MgSO_4_), and the solvent was evaporated under reduced pressure. The residue was purified by flash column chromatography using silica gel as the stationary phase, eluting with a pentane/EtOAc mixture (5 to 100 % EtOAc), to give the desired products.

All other experimental data and characterization is provided in the Supporting Information and the raw data can be found at https://zenodo.org/record/6638288

## Conflict of interest

The authors declare no conflict of interest.

1

## Supporting information

As a service to our authors and readers, this journal provides supporting information supplied by the authors. Such materials are peer reviewed and may be re‐organized for online delivery, but are not copy‐edited or typeset. Technical support issues arising from supporting information (other than missing files) should be addressed to the authors.

Supporting Information

## Data Availability

The data that support the findings of this study are available in the supplementary material of this article.

## References

[1a] J. Magano , J. R. Dunetz , Org. Process Res. Dev. 2012, 16, 1156–1184;

[1b] S. Rossi , A. Puglisi , L. Raimondi , M. Benaglia , ChemCatChem 2018, 10, 2717–2733;

[1c] P. K. Prasad , R. N. Reddi , S. Arumugam , Org. Biomol. Chem. 2018, 16, 9334–9348; 30516787 10.1039/c8ob02881h

[1d] L. A. T. Allen , R.-C. Raclea , P. Natho , P. J. Parsons , Org. Biomol. Chem. 2021, 19, 498–513. 33325975 10.1039/d0ob02098b

[2a] R. Cano , A. Zakarian , G. P. McGlacken , Angew. Chem. Int. Ed. 2017, 56, 9278–9290; 10.1002/anie.201703079 PMC6312110 28497890

[2b] Y. Liu , S.-J. Han , W.-B. Liu , B. M. Stoltz , Acc. Chem. Res. 2015, 48, 740–751; 25715056 10.1021/ar5004658 PMC6410712

[2c] D. A. Culkin , J. F. Hartwig , Acc. Chem. Res. 2003, 36, 234–245; 12693921 10.1021/ar0201106

[2d] Z.-T. He , J. F. Hartwig , J. Am. Chem. Soc. 2019, 141, 11749–11753; 31287682 10.1021/jacs.9b03291 PMC9059736

[2e] T. Hama , S. Ge , J. F. Hartwig , J. Org. Chem. 2013, 78, 8250–8266. 23931445 10.1021/jo401476f PMC3800138

[3a] E. Differding , G. M. Rüegg , R. W. Lang , Tetrahedron Lett. 1991, 32, 1779–1782;

[3b] J. A. Nobrega , S. M. C. Gonçalves , C. Peppe , Synth. Commun. 2002, 32, 3711–3717;

[3c] B. Sreedhar , P. Surendra Reddy , M. Madhavi , Synth. Commun. 2007, 37, 4149–4156;

[3d] T. Kösel , G. Dräger , A. Kirschning , Org. Biomol. Chem. 2021, 19, 2907–2911. 33734263 10.1039/d1ob00083g

[4a] S. Stavber , M. Jereb , M. Zupan , Synthesis 2002, 2002, 2609–2615;

[4b] T. Baumann , H. Vogt , S. Bräse , Eur. J. Org. Chem. 2007, 2007, 266–282;

[4c] H. Jia , A. P. Häring , F. Berger , L. Zhang , T. Ritter , J. Am. Chem. Soc. 2021, 143, 7623–7628; 33985330 10.1021/jacs.1c02606 PMC8297735

[4d] J. M. Janey , Angew. Chem. Int. Ed. 2005, 44, 4292–4300; 10.1002/anie.200462314 15945110

[4e] A. M. R. Smith , K. K. Hii , Chem. Rev. 2011, 111, 1637–1656. 20954710 10.1021/cr100197z

[5a] P. J. Chevis , S. G. Pyne , Org. Chem. Front. 2021, 8, 2287–2314;

[5b] P. Chauhan , S. Mahajan , D. Enders , Chem. Rev. 2014, 114, 8807–8864; 25144663 10.1021/cr500235v

[5c] G. Cecere , C. M. König , J. L. Alleva , D. W. C. MacMillan , J. Am. Chem. Soc. 2013, 135, 11521–11524; 23869694 10.1021/ja406181e PMC3786402

[5d] B. List , J. Am. Chem. Soc. 2002, 124, 5656–5657. 12010036 10.1021/ja0261325

[6] V. V. Zhdankin , P. J. Stang , Chem. Rev. 2008, 108, 5299–5358. 18986207 10.1021/cr800332c PMC2736367

[7] A. Bauer , N. Maulide , Chem. Sci. 2021, 12, 853–864. 34163852 10.1039/d0sc03266b PMC8178994

[8a] P. Mizar , T. Wirth , Angew. Chem. Int. Ed. 2014, 53, 5993–5997; 10.1002/anie.201400405 24846685

[8b] H. Jiang , H. Zhang , W. Xiong , C. Qi , W. Wu , L. Wang , R. Cheng , Org. Lett. 2019, 21, 1125–1129; 30714384 10.1021/acs.orglett.9b00072

[8c] A. A. More , G. K. Pathe , K. N. Parida , S. Maksymenko , Y. B. Lipisa , A. M. Szpilman , J. Org. Chem. 2018, 83, 2442–2447; 29334466 10.1021/acs.joc.7b03058

[8d] B. Sundararaju , M. Achard , C. Bruneau , Chem. Soc. Rev. 2012, 41, 4467–4483; 22576362 10.1039/c2cs35024f

[8e] L. Jiang , Z. Wang , M. Armstrong , M. G. Suero , Angew. Chem. Int. Ed. 2021, 60, 6177–6184; 10.1002/anie.202015077 33275325

[8f] E. A. Merritt , B. Olofsson , Synthesis 2011, 2011, 517–538;

[8g] G. C. Geary , E. G. Hope , K. Singh , A. M. Stuart , RSC Adv. 2015, 5, 16501–16506;

[8h] H. K. Minhas , W. Riley , A. M. Stuart , M. Urbonaite , Org. Biomol. Chem. 2018, 16, 7170–7173. 30246855 10.1039/c8ob02236d

[9] M. Fujio , A. Moriyasu , T. Tatsuo , I. Juichi , Bull. Chem. Soc. Jpn. 1978, 51, 335–336.

[10a] S. Arava , J. N. Kumar , S. Maksymenko , M. A. Iron , K. N. Parida , P. Fristrup , A. M. Szpilman , Angew. Chem. Int. Ed. 2017, 56, 2599–2603; 10.1002/anie.201610274 28128488

[10b] S. Maksymenko , K. N. Parida , G. K. Pathe , A. A. More , Y. B. Lipisa , A. M. Szpilman , Org. Lett. 2017, 19, 6312–6315; 29140710 10.1021/acs.orglett.7b03064

[10c] J. Li , A. Bauer , G. Di Mauro , N. Maulide , Angew. Chem. Int. Ed. 2019, 58, 9816–9819; 10.1002/anie.201904899 PMC6771532 31112360

[10d] A. Bauer , G. Di Mauro , J. Li , N. Maulide , Angew. Chem. Int. Ed. 2020, 59, 18208–18212; 10.1002/anie.202007439 PMC7589340 32808419

[10e] B. S. Martins , D. Kaiser , A. Bauer , I. Tiefenbrunner , N. Maulide , Org. Lett. 2021, 23, 2094–2098; 33635665 10.1021/acs.orglett.1c00251 PMC7985840

[10f] J. Borrel , J. Waser , Org. Lett. 2022, 24, 142–146. 34898230 10.1021/acs.orglett.1c03771

[11] T. Okuyama , T. Takino , T. Sueda , M. Ochiai , J. Am. Chem. Soc. 1995, 117, 3360–3367.

[12] P.-O. Norrby , T. B. Petersen , M. Bielawski , B. Olofsson , Chem. Eur. J. 2010, 16, 8251–8254. 20564301 10.1002/chem.201001110

[13a] M. Ochiai , Y. Takeuchi , T. Katayama , T. Sueda , K. Miyamoto , J. Am. Chem. Soc. 2005, 127, 12244–12245; 16131201 10.1021/ja0542800

[13b] T. Hokamp , T. Wirth , Chem. Eur. J. 2020, 26, 10417–10421. 32233006 10.1002/chem.202000927 PMC7496773

[14a] T. Nabana , H. Togo , J. Org. Chem. 2002, 67, 4362–4365; 12054975 10.1021/jo0200670

[14b] K. C. Nicolaou , T. Montagnon , T. Ulven , P. S. Baran , Y. L. Zhong , F. Sarabia , J. Am. Chem. Soc. 2002, 124, 5718–5728; 12010045 10.1021/ja012146j

[14c] M. Yoshida , K. Fujikawa , S. Sato , S. Hara , Arkivoc. 2003, 6, 36–42;

[14d] A. E. Allen , D. W. C. MacMillan , J. Am. Chem. Soc. 2010, 132, 4986–4987. 20297822 10.1021/ja100748y PMC2880471

[15] G. M. Kiefl , T. Gulder , J. Am. Chem. Soc. 2020, 142, 20577–20582. 33231441 10.1021/jacs.0c10700

[16] A. Sanz-Marco , S. Martinez-Erro , M. Pauze , E. Gómez-Bengoa , B. Martín-Matute , Nat. Commun. 2019, 10, 5244. 31748504 10.1038/s41467-019-13175-5 PMC6868166

[17] A. K. Ghosh , M. Brindisi , J. Med. Chem. 2015, 58, 2895–2940. 25565044 10.1021/jm501371s PMC4393377

[18a] Y. Peng , J. Liu , C. Qi , G. Yuan , J. Li , H. Jiang , Chem. Commun. 2017, 53, 2665–2668; 10.1039/c6cc09762f 28111646

[18b] E. Speckmeier , M. Klimkait , K. Zeitler , J. Org. Chem. 2018, 83, 3738–3745; 29504394 10.1021/acs.joc.8b00096

[18c] H. Jiang , H. Zhang , W. Xiong , C. Qi , W. Wu , L. Wang , R. Cheng , Org. Lett. 2019, 21, 1125–1129. 30714384 10.1021/acs.orglett.9b00072

[19] B. M. Trost , J. Xu , M. Reichle , J. Am. Chem. Soc. 2007, 129, 282–283. 17212401 10.1021/ja067342a PMC2533583

[20] X. Wang , A. Studer , Acc. Chem. Res. 2017, 50, 1712–1724. 28636313 10.1021/acs.accounts.7b00148 PMC5529030

[21] C. R. Gonçalves , M. Lemmerer , C. J. Teskey , P. Adler , D. Kaiser , B. Maryasin , L. González , N. Maulide , J. Am. Chem. Soc. 2019, 141, 18437–18443. 31714077 10.1021/jacs.9b06956 PMC6879173

[22] J. Blom , G. J. Reyes-Rodríguez , H. N. Tobiesen , J. N. Lamhauge , M. V. Iversen , C. L. Barløse , N. Hammer , M. Rusbjerg , K. A. Jørgensen , Angew. Chem. Int. Ed. 2019, 58, 17856–17862; 10.1002/anie.201911793 31595649

[23] C. Zhu , Y. Zhang , H. Zhao , S. Huang , M. Zhang , W. Su , Adv. Synth. Catal. 2015, 357, 331–338.

[24a] Y. G. Lee , K. Ishimaru , H. Iwasaki , K. Ohkata , K. Akiba , J. Org. Chem. 1991, 56, 2058–2066;

[24b] Y. Nakamura , Y. Ozeki , K. Uneyama , J. Fluorine Chem. 2008, 129, 274–279.

[25] A. D. Becke , J. Chem. Phys. 1993, 98, 5648–5652.

[26] Y. Zhao , D. G. Truhlar , Theor. Chem. Acc. 2008, 120, 215–241.

[27] Gaussian 16, Revision C.01, M. J. Frisch, G. W. Trucks, H. B. Schlegel, G. E. Scuseria, M. A. Robb, J. R. Cheeseman, G. Scalmani, V. Barone, G. A. Petersson, H. Nakatsuji, X. Li, M. Caricato, A. V. Marenich, J. Bloino, B. G. Janesko, R. Gomperts, B. Mennucci, H. P. Hratchian, J. V. Ortiz, A. F. Izmaylov, J. L. Sonnenberg, D. Williams-Young, F. Ding, F. Lipparini, F. Egidi, J. Goings, B. Peng, A. Petrone, T. Henderson, D. Ranasinghe, V. G. Zakrzewski, J. Gao, N. Rega, G. Zheng, W. Liang, M. Hada, M. Ehara, K. Toyota, R. Fukuda, J. Hasegawa, M. Ishida, T. Nakajima, Y. Honda, O. Kitao, H. Nakai, T. Vreven, K. Throssell, J. A. Montgomery, Jr., J. E. Peralta, F. Ogliaro, M. J. Bearpark, J. J. Heyd, E. N. Brothers, K. N. Kudin, V. N. Staroverov, T. A. Keith, R. Kobayashi, J. Normand, K. Raghavachari, A. P. Rendell, J. C. Burant, S. S. Iyengar, J. Tomasi, M. Cossi, J. M. Millam, M. Klene, C. Adamo, R. Cammi, J. W. Ochterski, R. L. Martin, K. Morokuma, O. Farkas, J. B. Foresman, D. J. Fox, Gaussian, Inc., Wallingford CT, **2016**.

[28a] B. Wang , H.-X. Sun , Z. H. Sun , J. Org. Chem. 2009, 74, 1781–1784; 19146386 10.1021/jo802472s

[28b] R. A. Fernandes , S. P. Gholap , S. V. Mulay , RSC Adv. 2014, 4, 16438–16443;

[28c] B. Chen , H. .-X. Sun , J. .-F. Qin , B. Wang , Tetrahedron Lett. 2016, 57, 253–255.

[29a] M. C. Hillier , A. I. Meyers , Tetrahedron Lett. 2001, 42, 5145–5147;

[29b] Q. Pu , X. Tang , L. Gao , Z. Song , Org. Chem. Front. 2018, 5, 2035–2039;

[29c] S. S. Kelly , T. .-L. Shen , M. Xian , Org. Lett. 2021, 23, 3741–3745; 33872038 10.1021/acs.orglett.1c01149

[29d] T. Hu , L. Huang , L. Gao , Z. Song , Org. Chem. Front. 2020, 7, 543–547.

[30a] L. Catalano, G. Cavallo, P. Metrangolo, G. Resnati, Halogen Bonding in Hypervalent Iodine Compounds. In Hypervalent Iodine Chemistry; Wirth, T., Ed.; Topics in Current Chemistry, Springer: Cham, Switzerland, **2016**, p. 289; 10.1007/128_2015_666 26809623

[30b] Y. Wang , P. Su , ACS Omega 2020, 5, 21862–21872. 32905280 10.1021/acsomega.0c03000 PMC7469379

