# Supplementary material for: An Expedient Method for the Umpolung Coupling of Enols with Heteronucleophiles
Source: Chemistry. 2022 Jun 17;28(44):e202201000. doi: 10.1002/chem.202201000 (PMC9400875; doi:10.1002/chem.202201000)
Supplement: Supplementary file 1 — Supporting Information [file CHEM-28-0-s001.pdf]

Chemistry–A European Journal

Supporting Information

# An Expedient Method for the Umpolung Coupling of Enols with Heteronucleophiles

Víctor García-Vázquez, Alba Carretero Cerdán, Amparo Sanz-Marco, Enrique Gómez-Bengoa, and Belén Martín-Matute*

## Table of contents


**S2** General Information

**S2** Mechanistic investigations and control experiments

**S5** General procedure for the synthesis of silyl enol ethers **1a-1h**

**S6** General procedure for the synthesis of organic carbamates **5a-5l**, **7a-7d**

**S6** General procedure for the synthesis of α-substituted ketones **8a-8c, 8m-8q**

**S6** General procedure for the synthesis of α-substituted ketones: one-pot two steps procedure **8d-8l**

**S6** General procedure for the synthesis of **8a** in 1 mmol scale

**S7** Characterization of **2c**, **1a-1h**, **5a-5l**, **7a-7d** and **8a-8q**

**S28** $^{1}$H NMR, and $^{13}$C NMR of products **5a-5l**, **7a-7d, 8a-8q**

**S63** Computational details

**S63** Optimized enolonium intermediates 3D structures

**S63** Cartesian coordinates

**S79** Reaction coordinates for the silylation process

**S85** References

# General information

All reagents were utilized without any further purification as obtained from commercial sources. Flash chromatography was performed with 60 Å (35-70 μm) silica gel (GC 60A 35-70 Micron, DAVISIL). Analytical TLC was performed on aluminum plates pre-coated (0-25 mm) with silica gel (Merck, Silica Gel 60 F254). Compounds were detected by exposure to UV light or by revealing the plates in a solution of 5% $KMnO_4$ in water. $^{1}H$, $^{13}C$ and $^{19}F$ NMR spectra were recorded at 400 MHz, 100 MHz and 376 MHz respectively on a Bruker Advance spectrometer. Chemical shifts ($\delta$) are shown in ppm, using as a reference the residual peaks of $CDCl_3$ ($\delta_H$ 7.26 and $\delta_C$ 77.00). Coupling constants (*J*) are given in Hz. Infrared (IR) spectra were recorded on a Varian 610-IR FTIR spectrometer ($\nu_{max}$ in $cm^{-1}$). Samples were recorded neat as thin films. High resolution mass spectra (HRMS) were recorded on Bruker microTOF mass spectrometer using ESI ionization.

# Mechanistic investigations and control experiments

## 1. Optimization studies

**Table S1. Optimization of reaction conditions**

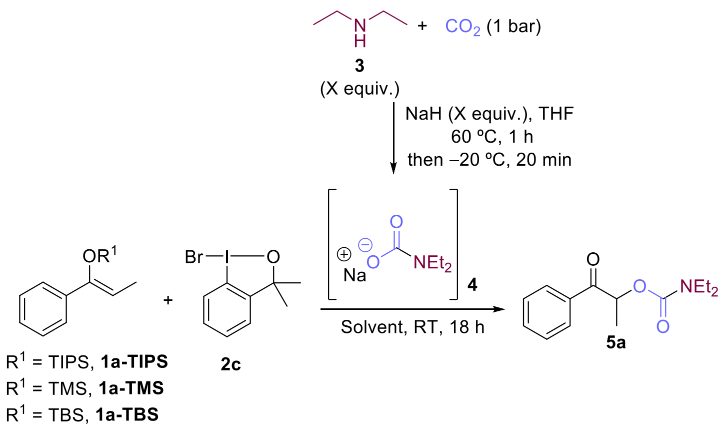


| Entry[a] | $R^1$ | NaH [equiv.] | **3** [equiv.] | **2c** [equiv.] | Yield of **5a** [%][b] |
|---|---|---|---|---|---|
| 1 | TIPS | 3 | 2 | 1.2 | 27 |
| 2 | TIPS | 2 | 2 | 1.2 | 43 |
| 3 | TIPS | 1.5 | 2 | 1.2 | 59 |
| 4 | TIPS | 1 | 2 | 1.2 | 51 |
| 5 | TIPS | 1.5 | 1.5 | 1.2 | 43 |
| 6 | TIPS | 1.5 | 2 | 1.5 | 68 |

| | | | | | |
|---|---|---|---|---|---|
| 7 | TBS | 1.5 | 2 | 1.5 | 77 |
| 8 | TMS | 1.5 | 2 | 1.5 | 52 |
| 9[c] | TBS | 1.5 | 2 | 1.5 | - |

[a]: Reaction conditions: **1** (0.1 mmol, 1 equiv.) DMF (0.33 M), RT, $CO_2$ (1 bar). [b]: Yields determined by $^1$H NMR spectroscopy using 2,3,5,6-tetrachlorobenzene (0.1 mmol, 1 equiv.) as an internal standard. [c]: In toluene, THF, 2-methyltetrahydrofuran, or acetone (0.33 M).

## 2. Control experiments and mechanistic investigations.

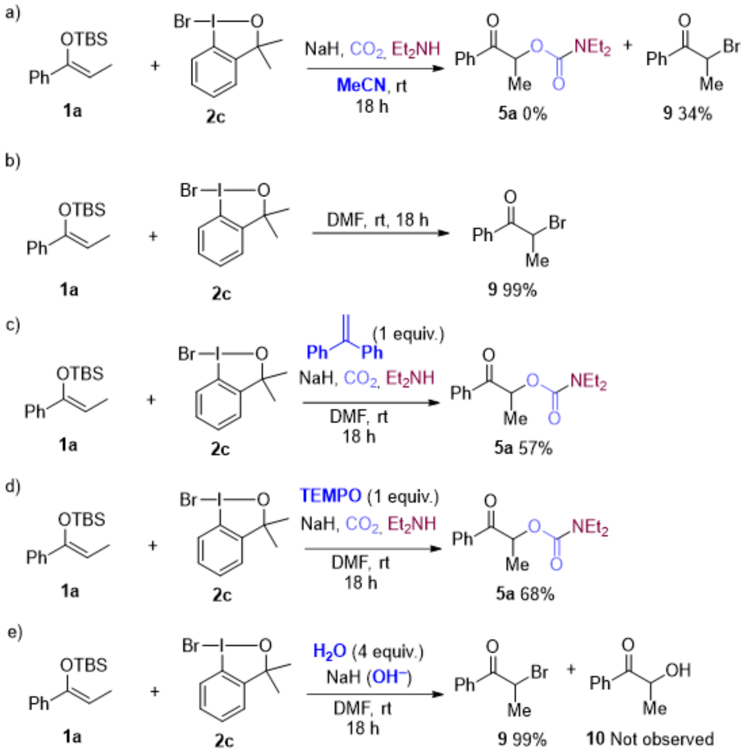


**Scheme S2**. Control experiments and mechanistic investigations.

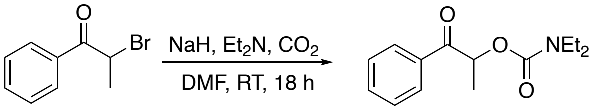


**9** **5a: 99% yield**

**Scheme S3.** Nucleophilic substitution reaction.

## 3. Other brominating reagents

Reaction conditions: α-Bromoketone (**9**, 0.1 mmol), NaH (0.15 mmol, 1.5 equiv.) diethyl amine (0.2 mmol, 2 equiv.), $CO_2$ (1 bar), DMF (0.33 M), 18 h.

Procedure: To a suspension of NaH (0.15 mmol, 1.5 equiv.) in THF, diethyl amine (0.2 mmol, 2 equiv.) was added, and the mixture was stirred at 60 °C for 1 h. $CO_2$ was bubbled through the mixture until THF (0.3 mL) was completely evaporated at –20 °C. To the resulting white precipitate (carbamate anion), DMF (0.3 mL) and α-bromoketone **9** were added. The reaction was then stirred at RT for 18 h. The crude was extracted with EtOAc (3 x 1 mL) and the organic layers were combined, dried over $MgSO_4$ and concentrated under reduced pressure.

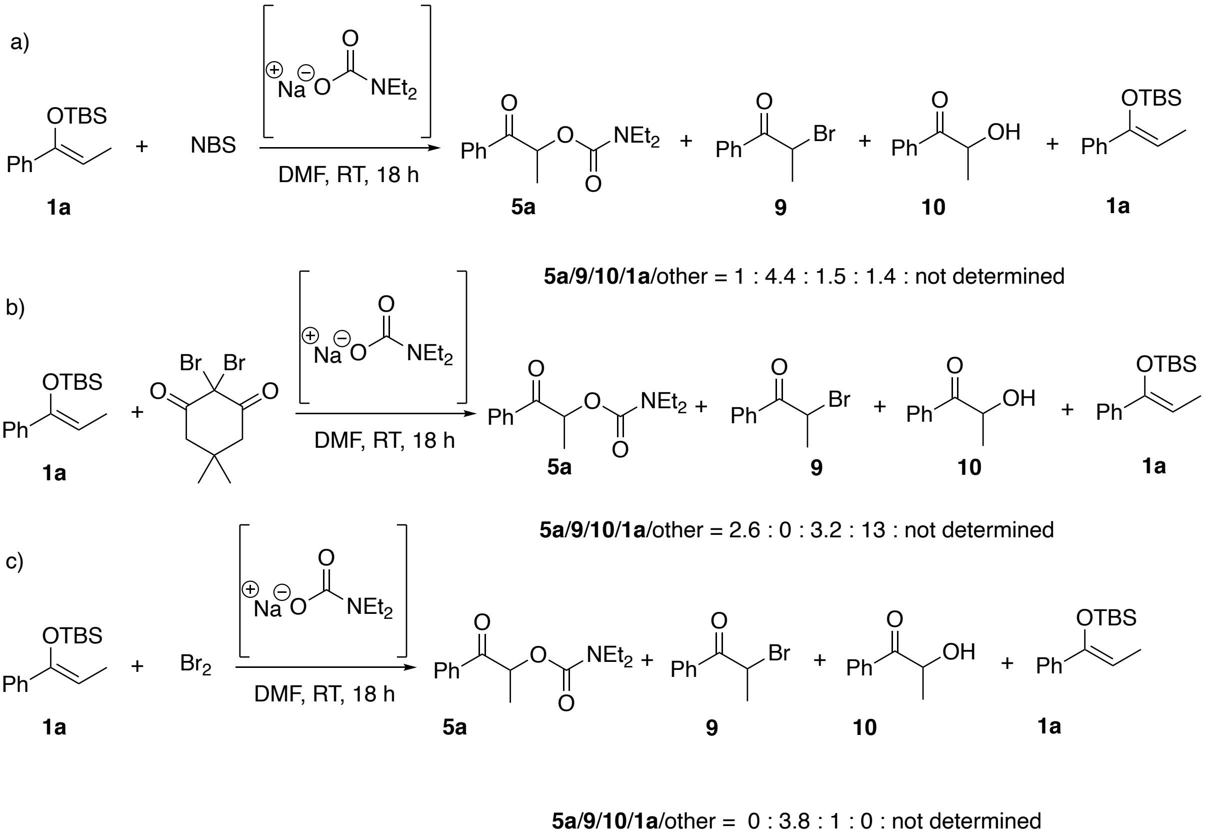


**Scheme S4.** Relative ratios observed with other electrophilic brominating reagents, only identified products are shown. The structures of other by-products formed in trace amounts were not elucidated.

4. Unsuccessful substrates:

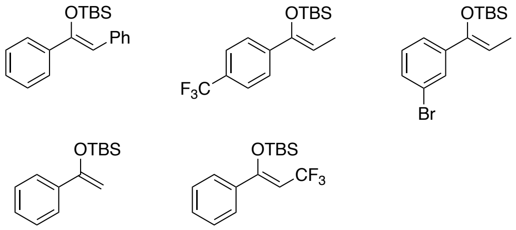


**Scheme S5.** Unsuccessful silyl enol ethers.

## General procedure for the synthesis of silyl enol ethers (GPA)

To a solution of the corresponding ketone (4 mmol, 1 equiv.) in MeCN (0.5 M) under an argon atmosphere, *tert*-butyldimethylsilyl chloride (730 mg, 5.2 mmol, 1.3 equiv.), sodium iodide (558 mg, 4 mmol, 1 equiv.) and triethylamine (0.5 mL, 4 mmol, 1 equiv.) were added at 0 °C. The reaction mixture was then warmed up to room temperature and stirred for additional 18 h. The mixture was then quenched using $NH_4Cl$ (8 mL), extracted with EtOAc (3x 10 mL), washed with brine (30 mL), dried over $MgSO_4$ and evaporated under reduced pressure. The silyl enol (**1**) ethers were isolated by flash column chromatography (FCC) using silica gel as stationary phase and a mixture of pentane:EtOAc (99:1).

## General procedure for the synthesis of organic carbamates (GPB)

To a suspension of NaH (60% in mineral oil, 6 mg, 0.15 mmol,1.5 equiv.) in THF (0.5 M) under an atmosphere of argon, diethylamine (24 µL, 0.2 mmol, 2 equiv.) was added, and the mixture was stirred at 60 °C for 1 h. The reaction was then cooled to –20 °C and $CO_2$ was bubbled for 30 min. After warming the mixture to room temperature (2-3 minutes), 1-bromo-3,3-dimethyl-1,3-dihydro-1λ3-benzo[d][1,2]iodaoxole (51 mg, 0.15 mmol, 1.5 equiv.), the silyl enol ether (**1**, 0.1 mmol, 1 equiv.) and DMF (0.33 M) were added slowly and the reaction mixture was stirred at room temperature for 18 h. After completion, the crude was extracted with EtOAc (3 x 1 mL) and the organic layers were combined, dried over $MgSO_4$ and concentrated under reduced pressure. The products were isolated by flash column chromatography (FCC) using silica gel as stationary phase and a gradient of DCM:MeOH as eluent (0 to 2% of MeOH)

## General procedure for the synthesis of α-substituted ketones (GPC)

1-Bromo-3,3-dimethyl-1,3-dihydro-1λ3-benzo[d][1,2]iodaoxole (**2c**, 0.15 mmol, 1.5 equiv.), the nucleophile (0.2 mmol, 2 equiv.) and NaH (0.15 mmol, 1.5 equiv. 60% in mineral oil) were placed in a pressure tube. The tube was sealed with a teflon cap. A solution of (*Z*)-*tert*-butyldimethyl((1-phenylprop-1-en-1-yl)oxy)silane (**1a**, 0.1 mmol, 1 equiv.) in DMF (1 mL, 0.1M) was then added under an atmosphere of argon. The mixture was stirred overnight (18 h) at room temperature. The final mixture was extracted with EtOAc (3 x 5 mL), dried with $MgSO_4$, and the solvent was evaporated under reduced pressure. The products were purified by flash column chromatography (FCC) using silica gel as stationary phase and a mixture of pentane:EtOAc as eluent (10 to 100% EtOAc).

## General procedure for the synthesis of α-substituted ketones: one-pot two steps procedure (GPD)

(*Z*)-*tert*-Butyldimethyl((1-phenylprop-1-en-1-yl)oxy)silane (**1a**, 0.1 mmol, 1 equiv.) and 1-bromo-3,3-dimethyl-1,3-dihydro-1λ3-benzo[d][1,2]iodaoxole (**2c**, 0.15 mmol, 1.5 equiv.) were placed in a pressure tube sealed with a teflon cap. The tube was flashed with argon, and DMF (0.5 mL, 0.2M) was added. The mixture was stirred overnight (18 h) at room temperature. After 18 h, α-bromoketone **9** is formed quantitatively, as confirmed by TLC and NMR. Then a suspension of the nucleophile (0.2 mmol, 2 equiv.) and NaH (0.15 mmol, 1.5 equiv., 60% in mineral oil) in DMF (0.5 mL, 0.2M) is transferred with a syringe to the sealed tube. The mixture is stirred at room temperature for additional 4 h, and the mixture is then extracted with EtOAc (3 x 5 mL) and dried with $MgSO_4$. The solvent was evaporated under reduced pressure. The products were purified by flash column chromatography using silica gel as stationary phase and a mixture of pentane:EtOAc as eluent (10 to 100%) EtOAc.

## General procedure for the synthesis of 8a in 1 mmol scale

1-Bromo-3,3-dimethyl-1,3-dihydro-1λ3-benzo[d][1,2]iodaoxole (**2c**, 511 mg, 1.5 mmol, 1.5 equiv.), benzoic acid (244 mg, 2.0 mmol, 2 equiv.) and NaH (36 mg, 1.5 mmol, 1.5 equiv. 60% in mineral oil) were placed in a round bottom flask. The flask was sealed with a septum. A solution of (*Z*)-*tert*-butyldimethyl((1-phenylprop-1-en-1-yl)oxy)silane (**1a**, 248 mg, 1 mmol, 1 equiv.) in DMF (10 mL, 0.1M) was then added under an atmosphere of argon. The mixture was stirred overnight (18 h) at room temperature. The final mixture was extracted with EtOAc (3 x

5 mL), dried with $MgSO_4$, and the solvent was evaporated under reduced pressure. The products were purified by flash column chromatography (FCC) using silica gel as stationary phase and a mixture of pentane:EtOAc as eluent (5 to 10% of EtOAc) a white solid (200 mg, 0.787 mmol, 79%).

## Characterization of 2c, 1a-1h, 5a-5l, 7a-7d and 8a-8q

**Synthesis of 1-bromo-3,3-dimethyl-1,3-dihydro-1λ3-benzo[d][1,2]iodaoxole (2c)**

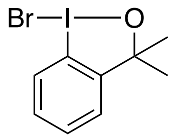


The title compound was synthesized following a reported procedure using NBS (1.63 g, 9.16 mmol) and 2-(2-iodophenyl)propan-2-ol (2 g, 7.64 mmol) in $CHCl_3$ (25 mL). The final compound was isolated as bright yellow crystals (1.6 g, 60% isolated yield). The physical and spectroscopic data agreed with the described in the literature. **$^1$H NMR (400 MHz, $CDCl_3$-*d*)** δ 8.19 – 7.90 (m, 1H), 7.69 – 7.50 (m, 2H), 7.27 – 7.06 (m, 1H), 1.58 (s, 6H). **$^{13}$C NMR (100 MHz, $CDCl_3$-*d*)** δ 149.8, 131.1, 130.4, 129.3, 112.0, 84.2, 29.2.

For complete characterization see: C. Braddock, G. Cansell, S. A. Hermitage, A. J. P. White, *Chem. Commun.* **2006**, *13*, 1442-1444.

**(*Z*)-*tert*-butyldimethyl((1-phenylprop-1-en-1-yl)oxy)silane (1a)**

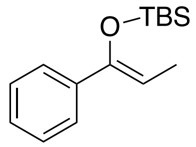


The title compound was synthesized according to the GPA using propiophenone (4 mmol, 536 mg) as substrate. The final compound was isolated by flash column chromatography (FCC) using silica gel as stationary phase and a mixture of pentane:EtOAc (99:1) as a colorless oil (794 mg, 80% isolated yield). **$^1$H NMR (400 MHz, $CDCl_3$-*d*)** δ 7.45 – 7.41 (m, 2H), 7.31 – 7.21 (m, 3H), 5.20 (q, $J$ = 7.0 Hz, 1H), 1.74 (d, $J$ = 7.0 Hz, 3H), 0.99 (s, 9H), -0.03 (s, 6H). **$^{13}$C NMR (100 MHz, $CDCl_3$-*d*)** δ 150.1, 139.6, 128.0, 127.3, 125.8, 106.4, 26.1, 18.4, 11.9, -4.0.

**(*Z*)-*tert*-butyl((1-(4-methoxyphenyl)prop-1-en-1-yl)oxy)dimethylsilane (1b)**

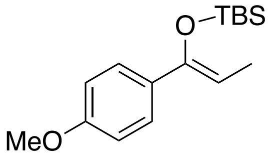


The title compound was synthesized according to the GPA using 1-(4-methoxyphenyl)propan-1-one (4 mmol, 656 mg) as substrate. The final compound was isolated by flash column chromatography (FCC) using silica gel as stationary phase and a mixture of pentane:EtOAc (99:1) as a colorless oil (1113 mg, 99% isolated yield). **$^1$H NMR (400 MHz, $CDCl_3$-*d*)** δ 7.35 (d, *J* = 9.0 Hz, 2H), 6.81 (d, *J* = 9.0 Hz, 2H), 5.08 (q, *J* = 7.0 Hz, 1H), 3.80 (s, 3H) 1.71 (d, *J* = 7.0 Hz, 3H), 0.99 (s, 9H), -0.04 (s, 6H). **$^{13}$C NMR (100 MHz, $CDCl_3$-*d*)** δ 158.9, 149.9, 131.7, 126.9, 113.2, 104.3, 55.2, 25.9, 18.4, 11.7, - 4.0.

**(*Z*)-*tert*-butyldimethyl((1-(p-tolyl)prop-1-en-1-yl)oxy)silane (1c)**

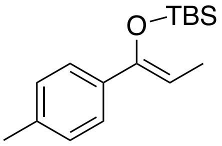


The title compound was synthesized according to the GPA using 1-(p-tolyl)propan-1-one (4 mmol, 592 mg) as substrate. The final compound was isolated by flash column chromatography (FCC) using silica gel as stationary phase and a mixture of pentane:EtOAc (99:1) as a colorless oil (891 mg, 85% isolated yield). **$^1$H NMR (400 MHz, $CDCl_3$-*d*)** δ 7.32 (d, *J* = 8.0 Hz, 2H), 7.10 – 7.07 (m, 2H), 5.15 (q, *J* = 7.0 Hz, 1H), 2.33 (s, 3H) 1.72 (d, *J* = 7.0 Hz, 3H), 0.99 (s, 9H), - 0.04 (s, 6H). **$^{13}$C NMR (100 MHz, $CDCl_3$-*d*)** δ 150.2, 137.0, 136.9, 128.6, 125.6, 105.0, 25.9, 21.1, 18.4, 11.7, -4.0

**(*Z*)-*tert*-butyl((1-(4-fluorophenyl)prop-1-en-1-yl)oxy)dimethylsilane (1d)**

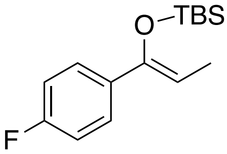


The title compound was synthesized according to the GPA using 1-(4-fluorophenyl)propan-1-one (4 mmol, 608 mg) as substrate. The final compound was isolated by flash column chromatography (FCC) using silica gel as stationary phase and a mixture of pentane:EtOAc (99:1) as a colorless oil (820 mg, 77% isolated yield). **$^1$H NMR (400 MHz, $CDCl_3$-*d*)** δ 7.57 –

7.28 (m, 2H), 7.00 – 6.93 (m, 2H), 5.13 (q, *J* = 7.0 Hz, 1H), 1.72 (d, *J* = 7.0 Hz, 3H), 0.98 (s, 9H), -0.05 (s, 6H). **$^{13}$C NMR (100 MHz, $CDCl_3$-*d*)** δ 162.2 (d, *J* = 246.0 Hz), 149.3, 136.0 (d, *J* = 3.0 Hz), 127.3 (d, *J* = 8.0 Hz), 114.7 (d, *J* = 21.5 Hz), 105.7, 25.8, 18.3, 11.7, -4.0

**(*Z*)-*tert*-butyldimethyl((1-(thiophen-2-yl)prop-1-en-1-yl)oxy)silane (1e)**

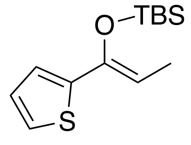


The title compound was synthesized according to the GPA using 1-(thiophen-2-yl)propan-1-one (4 mmol, 560 mg) as substrate. The final compound was isolated by flash column chromatography (FCC) using silica gel as stationary phase and a mixture of pentane:EtOAc (99:1) as a colorless oil (671 mg, 68% isolated yield). **$^{1}$H NMR (400 MHz, $CDCl_3$-*d*)** δ 7.10 (dd, *J* = 5.0, 1.0 Hz, 1H), 7.01 (dd, *J* = 3.5, 1.5 Hz, 1H), 6.92 (dd, *J* = 5.0, 1.0 Hz, 1H), 5.24 (q, *J* = 7.0 Hz, 1H), 1.71 (d, *J* = 7.0 Hz, 3H), 1.01 (s, 9H), 0.07 (s, 6H). **$^{13}$C NMR (100 MHz, $CDCl_3$-*d*)** δ 144.7, 143.6, 126.8, 125.5, 123.1, 105.6, 25.9, 18.4, 11.7, -3.9.

**(*Z*)-*tert*-butyldimethyl((1-phenylbut-1-en-1-yl)oxy)silane (1f)**

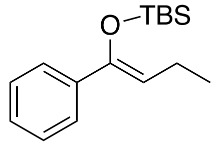


The title compound was synthesized according to the GPA using 1-phenylbutan-1-one (4 mmol, 592 mg) as substrate. The final compound was isolated by flash column chromatography (FCC) using silica gel as stationary phase and a mixture of pentane:EtOAc (99:1) as a colorless oil (859 mg, 82% isolated yield). **$^{1}$H NMR (400 MHz, $CDCl_3$-*d*)** δ 7.46 – 7.41 (m, 2H), 7.32 – 7.25 (m, 3H), 5.09 (q, *J* = 7.0 Hz, 1H), 2.22 (p, *J* = 7.5 Hz, 2H) 1.03 (t, *J* = 7.5 Hz, 3H), 0.99 (s, 9H), -0.04 (s, 6H). **$^{13}$C NMR (100 MHz, $CDCl_3$-*d*)** δ 148.7, 139.8, 128.3, 127.9, 125.8, 113.8, 25.9, 19.5, 18.3, 14.25, -4.1.

**(*Z*)-*tert*-butyl(hept-3-en-4-yloxy)dimethylsilane (1g)**

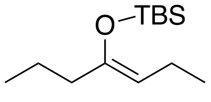

The title compound was synthesized according to the GPA using heptan-4-one (4 mmol, 456 mg) as substrate. The final compound was isolated by flash column chromatography (FCC) using silica gel as stationary phase and a mixture of pentane:EtOAc (99:1) as a colorless oil (456 mg, 50% isolated yield). **$^1$H NMR (400 MHz, $CDCl_3$-*d*)** δ 4.43(t, *J* = 7.0 Hz, 1H), 2.19 – 1.89 (m, 4H), 1.55 – 1.38 (m, 2H), 0.97 (s, 9H), ), 0.93 – 0.88 (m, 6H) 0.14 (s, 6H). **$^{13}$C NMR (100 MHz, $CDCl_3$-*d*)** δ 149.6, 109.9, 38.7, 25.8, 20.3, 18.6, 18.3, 14.5, 13.7, -4.1.

**(*Z*)-*tert*-butyldimethyl(non-4-en-5-yloxy)silane (1h)**

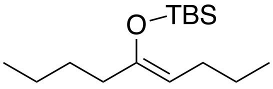


The title compound was synthesized according to the GPA using nonan-5-one (4 mmol, 568 mg) as substrate. The final compound was isolated by flash column chromatography (FCC) using silica gel as stationary phase and a mixture of pentane:EtOAc (99:1) as a colorless oil (450 mg, 44% isolated yield). **$^1$H NMR (400 MHz, $CDCl_3$-*d*)** δ 4.41(t, *J* = 7.0 Hz, 1H), 2.06 – 1.87 (m, 4H), 1.49 – 1.39 (m, 2H), 1.37 – 1.26 (m, 4H), 0.94 (s, 9H), ), 0.87 (t, *J* = 8.0 Hz, 6H) 0.11 (s, 6H). **$^{13}$C NMR (100 MHz, $CDCl_3$-*d*)** δ 150.4, 107.7, 36.3, 31.0, 29.4, 27.4, 25.9, 25.6, 23.1, 22.3, 18.3, 14.0, - 4.0.

**1-Oxo-1-phenylpropan-2-yl diethylcarbamate (5a)**

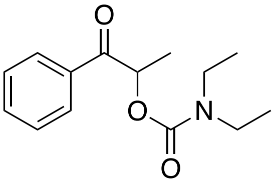


The title compound was synthesized according to the GPB using (*Z*)-*tert*-butyldimethyl((1-phenylprop-1-en-1-yl)oxy)silane (0.1 mmol, 25 mg) as substrate. The final compound was isolated by flash column chromatography (FCC) using silica gel as stationary phase and a gradient of DCM:MeOH as eluent (0 to 2% of MeOH) as a colorless oil (17 mg, 69% isolated yield). **$^1$H NMR (400 MHz, $CDCl_3$-*d*)** δ 8.03 – 7.87 (m, 2H), 7.62 – 7.51 (m, 1H), 7.50 – 7.40 (m, 2H), 5.95 (q, *J* = 7.0 Hz, 1H), 3.31 (brs, 4H), 1.51 (d, *J* = 7.0 Hz, 3H), 1.17 – 1.09 (m, 6H). **$^{13}$C NMR (100 MHz, $CDCl_3$-*d*)** δ 198.3, 155.2, 135.0, 133.4, 128.8, 128.6, 71.7, 42.1, 41.6, 17.4, 14.1, 13.6.

For complete characterization see: Y. Peng, J. Liu, C. Qi, G. Yuan, J. Li, H. Jiang, *Chem. Commun.*, **2017**, *53*, 2665-2668.

**1-(4-Methoxyphenyl)-1-oxopropan-2-yl diethylcarbamate (5b)**

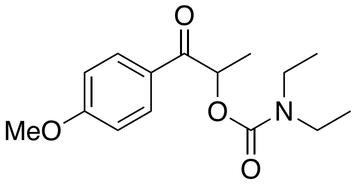


The title compound was synthesized according to the GPB using (*Z*)-*tert*-butyl((1-(4-methoxyphenyl)prop-1-en-1-yl)oxy)dimethylsilane (0.1 mmol, 28 mg) as substrate. The final compound was isolated by flash column chromatography (FCC) using silica gel as stationary phase and a gradient of DCM:MeOH as eluent (0 to 2% of MeOH) as a colorless oil (22 mg, 80% isolated yield). **$^{1}$H NMR (400 MHz, $CDCl_3$-*d*)** δ 8.05 – 7.78 (m, 2H), 7.04 – 6.78 (m, 2H), 5.93 (q, *J* = 7.0 Hz, 1H), 3.86 (s, 3H), 3.31 (brs, 4H), 1.49 (d, *J* = 7.0 Hz, 3H), 1.23 – 0.87 (m, 6H). **$^{13}$C NMR (100 MHz, $CDCl_3$-*d*)** δ 196.6, 163.8, 155.3, 131.0, 127.8, 114.0, 71.4, 55.6, 42.1, 41.6, 17.6, 14.1, 13.6.

For complete characterization see: Y. Peng, J. Liu, C. Qi, G. Yuan, J. Li, H. Jiang, *Chem. Commun.*, **2017**, *53*, 2665-2668.

**1-Oxo-1-(*p*-tolyl)propan-2-yl diethylcarbamate (5c)**

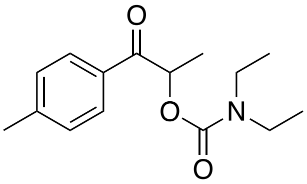


The title compound was synthesized according to the GPB using (*Z*)-*tert*-butyldimethyl((1-(p-tolyl)prop-1-en-1-yl)oxy)silane (0.1 mmol, 26 mg) as substrate. The final compound was isolated by flash column chromatography (FCC) using silica gel as stationary phase and a gradient of DCM:MeOH as eluent (0 to 2% of MeOH) as a colorless oil (16 mg, 60% isolated yield). **$^{1}$H NMR (400 MHz, $CDCl_3$-*d*)** δ 8.00 – 7.75 (m, 2H), 7.27 – 7.24 (d, *J* = 6.4 Hz, 2H), 5.94 (q, *J* = 7.0 Hz, 1H), 3.32 (brs, 4H), 2.40 (s, 3H), 1.49 (d, *J* = 7.0 Hz, 3H), 1.22 – 0.97 (m, 6H). **$^{13}$C NMR (100 MHz, $CDCl_3$-*d*)** δ 197.8, 155.2, 144.2, 132.3, 129.4, 128.9, 128.7, 71.6, 42.0, 41.6, 21.8, 17.4, 14.0, 13.6.

For complete characterization see: Y. Peng, J. Liu, C. Qi, G. Yuan, J. Li, H. Jiang, *Chem. Commun.*, **2017**, *53*, 2665-2668.

**1-(4-fluorophenyl)-1-oxopropan-2-yl diethylcarbamate (5d)**

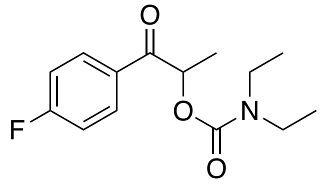

The title compound was synthesized according to the GPB using (*Z*)-*tert*-butyldimethyl((1-(4-fluorophenyl)prop-1-en-oxy)silane (0.1 mmol, 26 mg) as substrate. The final compound was isolated by flash column chromatography (FCC) using silica gel as stationary phase and a gradient of DCM:MeOH as eluent (0 to 2% of MeOH) as a colorless oil (14 mg, 52% isolated yield). **$^1$H NMR (400 MHz, $CDCl_3$-*d*)** δ 7.99 (dd, *J* = 9.0, 5.5 Hz) 2H), 7.22 – 7.05 (m, 2H), 5.89 (q, *J* = 7.0 Hz, 1H), 3.45 – 3.11 (m, 4H), 1.50 (d, *J* = 7.0 Hz, 3H), 1.13 (dt, *J* = 15.5, 7.0 Hz, 6H).**$^{13}$C NMR (100 MHz, $CDCl_3$-*d*)** δ 196.6, 165.8 (d, *J* = 255.0 Hz), 155.0, 131.2 (d, *J* = 3.0 Hz), 131.2 (d, *J* = 9.5 Hz), 115.8 (d, *J* = 22.0 Hz), 71.4, 42.0, 41.5, 17.1, 14.0, 13.4. **$^{19}$F NMR (376 MHz, $CDCl_3$-d)** δ –104.7 (s).

For complete characterization see: Y. Peng, J. Liu, C. Qi, G. Yuan, J. Li, H. Jiang, *Chem. Commun.*, **2017**, *53*, 2665-2668.

**1-oxo-1-(thiophen-2-yl)propan-2-yl diethylcarbamate (5e)**

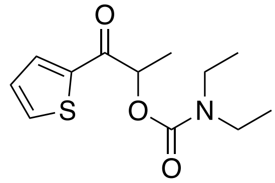

The title compound was synthesized according to the GPB using (*Z*)-*tert*-butyldimethyl((1-(thiophen-2-yl)prop-1-en-1-yl)oxy)silane (0.1 mmol, 25 mg) as substrate. The final compound was isolated by flash column chromatography (FCC) using silica gel as stationary phase and a gradient of DCM:MeOH as eluent (0 to 2% of MeOH) as a colorless oil (16 mg, 64% isolated yield). **$^1$H NMR (400 MHz, $CDCl_3$-*d*)** δ 7.81 (dd, *J* = 3.5, 1.0 Hz) 1H), 7.66 (dd, *J* = 5.0, 1.0 Hz, 1H), 7.14 (dd, *J* = 5.0, 4.0 Hz, 1H), 5.73 (q, *J* = 7.0 Hz, 1H), 3.40 – 3.20 (m, 4H), 1.55 (d, *J* = 7.0 Hz, 3H), 1.23 – 1.08 (m, 6H).**$^{13}$C NMR (100 MHz, $CDCl_3$-*d*)** δ 190.8, 155.0, 140.9, 134.0, 132.5, 128.1, 72.5, 42.0, 41.5, 25.7, 17.7, 14.0, 13.4.

For complete characterization see: Y. Peng, J. Liu, C. Qi, G. Yuan, J. Li, H. Jiang, *Chem. Commun.*, **2017**, *53*, 2665-2668.

**1-oxo-1-phenylbutan-2-yl diethylcarbamate (5f)**

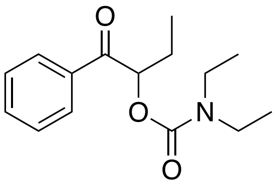

The title compound was synthesized according to the GPB using (*Z*)-*tert*-butyldimethyl((1-phenylbut-1-en-1-yl)oxy)silane (0.1 mmol, 26 mg) as substrate. The final compound was isolated by flash column chromatography (FCC) using silica gel as stationary phase and a gradient of DCM:MeOH as eluent (0 to 2% of MeOH) as a colorless oil (13 mg, 49% isolated yield). **$^{1}$H NMR (400 MHz, $CDCl_3$-*d*)** δ 7.96 (dd, *J* = 8.5, 1.5 Hz) 1H), 7.61– 7.52 (m, 1H), 7.51 – 7.42 (m, 1H), 5.80 (dd, *J* = 8.1, 4.4 Hz, 1H), 3.48 – 3.20 (m, 4H), 2.03 – 1.76 (m, 2H), 1.24 – 1.06 (m, 6H), 1.03 (d, *J* = 7.5 Hz, 3H).**$^{13}$C NMR (100 MHz, $CDCl_3$-*d*)** δ 197.9, 155.4, 135.4, 133.2, 128.7, 128.5, 76.5, 42.1, 41.7, 24.9, 14.1, 13.5, 10.0.

For complete characterization see: Y. Peng, J. Liu, C. Qi, G. Yuan, J. Li, H. Jiang, *Chem. Commun.*, **2017**, *53*, 2665-2668.

**4-oxoheptan-3-yl diethylcarbamate (5g)**

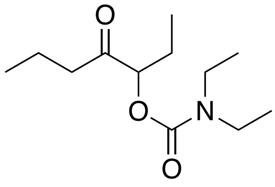

The title compound was synthesized according to the GPB using (*Z*)-*tert*-butyl(hept-3-en-4-yloxy)dimethylsilane (0.1 mmol, 23 mg) as substrate. The final compound was isolated by flash column chromatography (FCC) using silica gel as stationary phase and a gradient of DCM:MeOH as eluent (0 to 2% of MeOH) as a colorless oil (12 mg, 51% isolated yield). **$^{1}$H NMR (400 MHz, $CDCl_3$-*d*)** δ 4.90 (dd, *J* = 8.0, 4.5 Hz, 1H), 3.38 – 3.25 (m, 4H), 2.66 – 2.31 (m, 2H), 1.89 – 1.70 (m, 2H), 1.62 (q, *J* = 7.5 Hz, 2H), 1.21 – 1.11 (m, 6H), 0.98 (t, *J* = 7.5 Hz, 3H), 0.91 (t, *J* = 7.5 Hz, 3H).**$^{13}$C NMR (100 MHz, $CDCl_3$-*d*)** δ 208.8, 155.3, 79.9, 42.1, 41.5,

40.6, 24.2, 16.6, 14.2, 13.8, 13.5, 9.8. **FTIR** $\nu_{max/cm^{-1}}$ (neat) 2964, 1748, 1707, 1428, 1269, 1236, 1160, 1021, 763. **HRMS (ESI):** m/z calculated for $[C_{12}H_{23}NO_3Na]^+$: 252.1570; found: 252.1583.

**5-oxononan-4-yl diethylcarbamate (5h)**

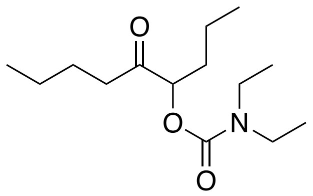

The title compound was synthesized according to the GPB using (*Z*)-*tert*-butyldimethyl(non-4-en-5-yloxy)silane (0.1 mmol, 26 mg) as substrate. The final compound was isolated by flash column chromatography (FCC) using silica gel as stationary phase and a gradient of DCM:MeOH as eluent (0 to 2% of MeOH) as a colorless oil (12 mg, 46% isolated yield). **$^1$H NMR (400 MHz, $CDCl_3$-*d*)** δ 4.94 (dd, *J* = 7.5, 5.5 Hz, 1H), 3.43 – 3.26 (m, 4H), 2.56 – 2.34 (m, 2H), 1.76 – 1.65 (m, 2H), 1.65 – 1.52 (m, 2H), 1.47 – 1.38 (m, 2H), 1.35 – 1.26 (m, 2H), 1.21 – 1.07 (m, 6H), 0.94 (t, *J* = 7.5 Hz, 3H), 0.89 (t, *J* = 7.5 Hz, 3H).**$^{13}$C NMR (100 MHz, $CDCl_3$-*d*)** δ 209.1, 155.4, 78.8, 42.0, 41.5, 38.2, 32.9, 25.7, 25.2, 22.4, 18.7, 14.1, 13.9, 13.8, 13.5. **FTIR** $\nu_{max/cm^{-1}}$ (neat) 2967, 2875, 2032, 1697, 1425, 1171. **HRMS (ESI):** m/z calculated for $[C_{14}H_{27}NO_3Na]^+$: 280.1883; found 280.1883.

**1-oxo-1-phenylpropan-2-yl benzyl(methyl)carbamate (5i)**

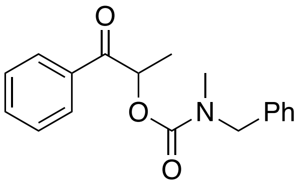

The title compound was synthesized according to the GPB using (*Z*)-*tert*-butyldimethyl((1-phenylprop-1-en-1-yl)oxy)silane (0.1 mmol, 25 mg) as substrate. The final compound was isolated by flash column chromatography (FCC) using silica gel as stationary phase and a gradient of DCM:MeOH as eluent (0 to 2% of MeOH) as a colorless oil (24 mg, 80% isolated yield). **$^1$H NMR (400 MHz, $CDCl_3$-*d*)** δ 8.00 (d, *J* = 7.5 Hz, 2H), 7.64 – 7.58 (m, 1H), 7.55 – 7.46 (m, 2H), 7.41 – 7.30 (m, 4H), 7.23 (d, *J* = 7.5 Hz, 1H), 6.01 (dq, *J* = 14.0, 7.0 Hz, 1H), 4.68 – 4.44 (m, 2H), 2.94 – 2.88 (two singlets, rotamers, 3H) 1.56 (dd, *J* = 14.5, 7.0 Hz, 3H).

**$^{13}$C NMR (100 MHz, $CDCl_3$-*d*)** δ 198.0, 197.9, 156.1, 155.6, 137.2, 134.8, 133.4, 128.8, 128.6, 128.5, 127.7, 127.6, 127.4, 127.4, 72.4, 72.2, 52.6, 52.5, 34.2, 33.7, 17.3, 17.2.

For complete characterization see: Y. Peng, J. Liu, C. Qi, G. Yuan, J. Li, H. Jiang, *Chem. Commun.*, **2017**, *53*, 2665-2668.

**1-oxo-1-phenylpropan-2-yl dibenzylcarbamate (5j)**

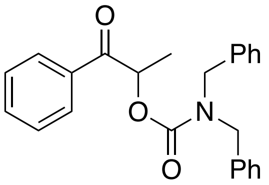


The title compound was synthesized according to the GPB using (*Z*)-*tert*-butyldimethyl((1-phenylprop-1-en-1-yl)oxy)silane (0.1 mmol, 25 mg) as substrate. The final compound was isolated by flash column chromatography (FCC) using silica gel as stationary phase and a gradient of DCM:MeOH as eluent (0 to 2% of MeOH) as a colorless oil (29 mg, 79% isolated yield). **$^{1}$H NMR (400 MHz, $CDCl_3$-*d*)** δ 8.01 (dd, *J* = 8.5, 1.3 Hz, 2H), 7.64 – 7.56 (m, 1H), 7.54 – 7.45 (m, 2H), 7.42 – 7.23 (m, 10H), 6.06 (q, *J* = 7.0 Hz, 1H), 4.46 (dd, *J* = 22.5, 7.5 Hz, 4H), 1.57 (d, *J* = 7.0 Hz, 3H). **$^{13}$C NMR (100 MHz, $CDCl_3$-*d*)** δ 197.8, 156.0, 137.1, 137.0, 134.8, 133.3, 128.8, 128.6, 128.6, 128.0, 127.9, 127.5, 126.7, 72.6, 49.3, 29.2, 17.2.

For complete characterization see: Y. Peng, J. Liu, C. Qi, G. Yuan, J. Li, H. Jiang, *Chem. Commun.*, **2017**, *53*, 2665-2668.

**1-oxo-1-phenylpropan-2-yl diisobutylcarbamate (5k)**

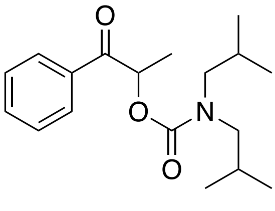


The title compound was synthesized according to the GPB using (*Z*)-*tert*-butyldimethyl((1-phenylprop-1-en-1-yl)oxy)silane (0.1 mmol, 25 mg) as substrate. The final compound was isolated by flash column chromatography (FCC) using silica gel as stationary phase and a gradient of DCM:MeOH as eluent (0 to 2% of MeOH) as a colorless oil (18 mg, 60% isolated yield). **$^{1}$H NMR (400 MHz, $CDCl_3$-*d*)** δ 7.98 – 7.91 (m, 2H), 7.58 – 7.52 (m, 1H), 7.48 – 7.41

(m, 2H), 5.93 (q, $J$ = 7.0 Hz, 1H), 3.22– 2.95 (m, 4H), 1.96 (tt, $J$ = 14.0, 7.0 Hz, 2H), 1.50 (d, $J$ = 7.0 Hz, 3H), 0.93– 0.77 (m, 12H). **$^{13}$C NMR (100 MHz, $CDCl_3$-*d*)** δ 198.1, 155.9, 134.9, 133.2, 128.6, 128.5, 71.7, 55.2, 54.8, 27.4, 26.9, 20.2, 20.1, 20.0, 19.9, 17.1.

For complete characterization see: Y. Peng, J. Liu, C. Qi, G. Yuan, J. Li, H. Jiang, *Chem. Commun.*, **2017**, *53*, 2665-2668.

**1-oxo-1-phenylpropan-2-yl pyrrolidine-1-carboxylate (5l)**

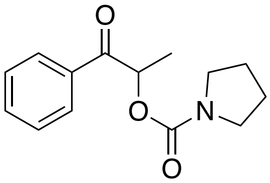

The title compound was synthesized according to the GPB using (*Z*)-*tert*-butyldimethyl((1-phenylprop-1-en-1-yl)oxy)silane (0.1 mmol, 25 mg) as substrate. The final compound was isolated by flash column chromatography (FCC) using silica gel as stationary phase and a gradient of DCM:MeOH as eluent (0 to 2% of MeOH) as a colorless oil (14 mg, 58% isolated yield). **$^{1}$H NMR (400 MHz, $CDCl_3$-*d*)** δ 7.97 (dd, $J$ = 8.5, 1.5 Hz, 2H), 7.60 – 7.54 (m, 1H), 7.50 – 7.43 (m, 2H), 5.94 (q, $J$ = 7.0 Hz, 1H), 3.56 – 3.24 (m, 4H), 1.97–1.80 (m, 4H), 1.51 (d, $J$ = 7.0 Hz, 3H). **$^{13}$C NMR (100 MHz, $CDCl_3$-*d*)** δ 198.2, 154.2, 134.8, 133.3, 128.6, 128.6, 71.5, 46.2, 45.9, 25.7, 24.9, 17.3.

For complete characterization see: Y. Peng, J. Liu, C. Qi, G. Yuan, J. Li, H. Jiang, *Chem. Commun.*, **2017**, *53*, 2665-2668.

**ethyl 2-((diethylcarbamoyl)oxy)-3-oxo-3-phenylpropanoate (7a)**

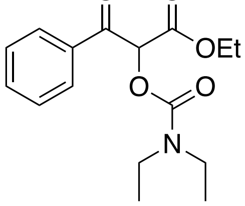

The title compound was synthesized according to the GPB using ethyl 3-oxo-3-phenylpropanoate (0.1 mmol, 19 mg) as substrate. The final compound was isolated by flash column chromatography (FCC) using silica gel as stationary phase and a gradient of

DCM:MeOH as eluent (0 to 2% of MeOH) as a colorless oil (28 mg, 82% isolated yield) as a 1.3:1 keto/enol mixture. **$^1$H NMR (400 MHz, CDCl$_3$-*d*)** δ 8.11 – 8.05 (m, 2H, enol tautomer), 8.11 – 8.05 (m, 2H, keto tautomer), 7.65 – 7.53 (m, 1H, keto tautomer, 1H, enol tautomer), 7.52– 7.42 (m, 2H, keto tautomer, 2H, enol tautomer), 6.43 (s, 1H, keto tautomer), 4.33 (dq, *J* = 7.0, 2.0 Hz, 2H, enol tautomer), 4.25 (dq, *J* = 7.0, 2.5 Hz, 2H, keto tautomer) 3.39 – 3.18 (m, 4H, keto tautomer, 4H, enol tautomer), 1.37 – 0.98 (m, 9H, keto tautomer, 9H, enol tautomer). **$^{13}$C NMR (100 MHz, CDCl$_3$-*d*)** δ 190.9, 185.9, 166.1, 164.1, 153.9, 151.0, 134.5, 133.9, 133.5, 132.3, 129.9, 129.2, 128.6, 128.3, 87.5, 74.8, 63.7, 62.1, 42.4, 42.4, 41.9, 41.7, 14.1, 13.9, 13.9, 13.7, 13.3, 13.0. **FTIR** $\nu_{max/cm}^{-1}$ (neat) 2978, 2929, 2073, 1706, 1596, 1448, 1401, 1133, 1024, 698. **HRMS (ESI):** m/z calculated for $[C_{16}H_{21}NO_5Na]^+$: 330.1312; found: 330.1327

**2,4-dioxopentan-3-yl diethylcarbamate (7b)**

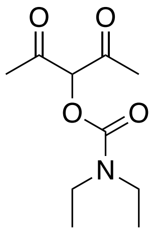

The title compound was synthesized according to the GPB using pentane-2,4-dione (0.1 mmol, 10 mg) as substrate. The final compound was isolated by flash column chromatography (FCC) using silica gel as stationary phase and a gradient of DCM:MeOH as eluent (0 to 2% of MeOH) as a colorless oil (12 mg, 56% isolated yield) as a 2.7:1 keto/enol mixture. **$^1$H NMR (400 MHz, CDCl$_3$-*d*)** δ 14.36 (s, 1H, enol tautomer), 5.49 (s, 1H, keto tautomer), 3.49 – 3.26 (m, 4H, keto tautomer, 4H, enol tautomer), 2.29 (s, 6H, keto tautomer), 2.04 (s, 6H, enol tautomer) 1.31 – 1.09 (m, 6H, keto tautomer, 6H, enol tautomer). **$^{13}$C NMR (100 MHz, CDCl$_3$-*d*)** δ 200.2, 185.3, 153.9, 153.8, 128.7, 85.5, 77.3, 42.6, 42.4, 41.9, 41.8, 27.4, 20.8, 14.4, 14.0, 13.5, 13.4. **FTIR** $\nu_{max/cm}^{-1}$ (neat) 2973, 2928, 2496, 2360, 1726, 1700, 1635, 1424, 1270, 1078, 763. **HRMS (ESI):** m/z calculated for $[C_{10}H_{17}NO_4Na]^+$: 238.1050; found: 238.1045

**methyl 3-(diethylamino)-2-((diethylcarbamoyl)oxy)-3-oxopropanoate (7c)**

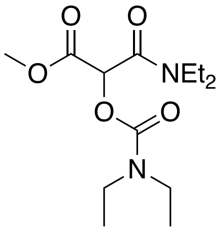


The title compound was synthesized according to the GPB using methyl 3-(diethylamino)-3-oxopropanoate (0.1 mmol, 17 mg) as substrate. The final compound was isolated by flash column chromatography (FCC) using silica gel as stationary phase and a gradient of DCM:MeOH as eluent (0 to 2% of MeOH) as a colorless oil (20 mg, 68% isolated yield). **$^1$H NMR (400 MHz, $CDCl_3$-*d*)** δ 5.88 (s, 1H), 3.80 (s, 1H), 3.53 – 3.28 (m, 8H), 1.24 (t, *J* = 7.0 Hz, 3H), 1.14 (t, *J* = 7.0 Hz, 3H). **$^{13}$C NMR (100 MHz, $CDCl_3$-*d*)** δ 167.0, 164.0, 154.1, 70.6, 52.7, 42.3, 42.1, 41.7, 40.6, 14.2, 13.9, 13.3, 12.6. **FTIR** $\nu_{max/cm}{}^{-1}$ (neat) 2956, 1748, 1707, 1428, 1269, 1236, 1160, 1021, 763. **HRMS (ESI):** m/z calculated for $[C_{13}H_{24}N_2O_5Na]^+$: 311.1577; found: 311.1559.

**dimethyl 2-((diethylcarbamoyl)oxy)malonate (7d)**

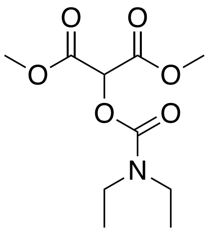


The title compound was synthesized according to the GPB using dimethyl malonate (0.1 mmol, 13 mg) as substrate. The final compound was isolated by flash column chromatography (FCC) using silica gel as stationary phase and a gradient of DCM:MeOH as eluent (0 to 2% of MeOH) as a colorless oil (19 mg, 78% isolated yield). **$^1$H NMR (400 MHz, $CDCl_3$-*d*)** δ 5.61 (s, 1H), 3.83 (s, 1H), 3.44 – 3.27 (m, 4H), 1.21 (t, *J* = 7.0 Hz, 3H), 1.15 (t, *J* = 7.0 Hz, 3H). **$^{13}$C NMR (100 MHz, $CDCl_3$-*d*)** δ 165.8, 153.9, 72.1, 53.2, 42.5, 41.9, 14.0, 13.4. **FTIR** $\nu_{max/cm}{}^{-1}$ (neat) 2954, 2923, 1733, 1435, 1254, 700. **HRMS (ESI):** m/z calculated for $[C_{10}H_{17}NO_6Na]^+$: 270.0948; found: 270.0977.

**1-oxo-1-phenylpropan-2-yl-benzoate (8a)**

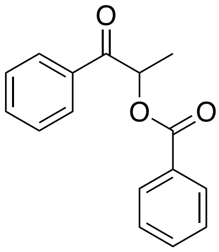

The title compound was synthesized according to the GPC using (*Z*)-*tert*-butyldimethyl((1-phenylprop-1-en-1-yl)oxy)silane (0.1 mmol, 25 mg) as substrate and benzoic acid (0.2 mmol, 24 mg) as nucleophile source. The product was isolated by flash column chromatography (FCC) using silica gel as stationary phase and a gradient of pentane:EtOAc as eluent (5 to 10% of EtOAc) a white solid (25 mg, 0.098 mmol, 98%). **$^{1}$H NMR** (400 MHz, $CDCl_3$): δ = 8.16 – 8.07 (m, 2H), 8.04 – 7.96 (m, 2H), 7.65 – 7.55 (m, 2H), 7.53 – 7.38 (m, 4H), 6.21 (q, J = 7.0 Hz, 1H), 1.68 (d, J = 7.0 Hz, 3H).**$^{13}$C NMR** (100 MHz, $CDCl_3$): δ = 196.9, 166.1, 134.6, 133.7, 133.4, 133.0, 129.6, 128.9, 128.6, 128.5, 72.0, 17.3.

For complete characterization see: M. Uyanik, D. Suzuki, T. Yasui, K. Ishihara, *Angew. Chem. Int. Ed.* **2011**, *50*, 5331.

**1-oxo-1-phenylpropan-2-yl 4-methoxybenzoate (8b)**

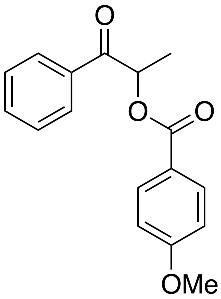

The title compound was synthesized according to the GPC using (*Z*)-*tert*-butyldimethyl((1-phenylprop-1-en-1-yl)oxy)silane (0.1 mmol, 25 mg) as substrate and *p*-methoxybenzoic acid (0.2 mmol, 30 mg) as nucleophile source. The product was isolated by flash column chromatography (FCC) using silica gel as stationary phase and a gradient of pentane:EtOAc as eluent (5 to 10% of EtOAc) a white solid (25 mg, 0.09 mmol, 89%). **$^{1}$H NMR** (400 MHz, $CDCl_3$): δ = 8.25 – 7.86 (m, 3H), 7.69 – 7.55 (m, 1H), 7.50 – 7.43 (m, 2H), 6.92 (d, *J* = 7.0 Hz, 2H), 6.18 (q, *J* = 7.0 Hz, 1H), 3.86 (s, 3H), 1.65 (d, *J* = 7.0 Hz, 3H). **$^{13}$C NMR** (100 MHz, $CDCl_3$): δ = 197.1, 165.8, 163.8, 134.7, 133.6, 132.1, 128.9, 128.7, 122.0, 113.8, 71.7, 55.6, 17.3.

For complete characterization see: B. Landers, C. Berini, C. Wang, O. Navarro, *J. Org. Chem.* **2011**, *76*, 1390–1397.

**1-oxo-1-phenylpropan-2-yl 4-(trifluoromethyl)benzoate (8c)**

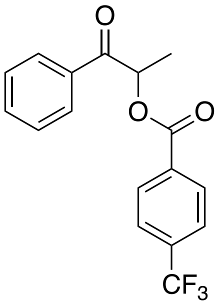

The title compound was synthesized according to the GPC using (*Z*)-*tert*-butyldimethyl((1-phenylprop-1-en-1-yl)oxy)silane (0.1 mmol, 25 mg) as substrate and *p*-trifluoromethylbenzoic acid (0.2 mmol, 38 mg) as nucleophile source. The product was isolated by flash column chromatography (FCC) using silica gel as stationary phase and a gradient of pentane:EtOAc as eluent (5 to 10% of EtOAc) a white solid (26 mg, 0.08 mmol, 80%). $^{1}$**H NMR** (400 MHz, $CDCl_3$): δ = 8.23 – 8.19 (m, 2H), 8.02 – 7.97 (m, 2H), 7.74 – 7.69 (m, 2H), 7.65 – 7.58 (m, 1H), 7.54 – 7.47 (m, 2H), 6.24 (q, *J* = 7.0 Hz, 1H), 1.70 (d, *J* = 7.0 Hz, 3H). $^{13}$**C NMR** (100 MHz, $CDCl_3$): δ = 196.2, 164.8, 142.7, 134.8 (q, *J* = 32.5 Hz), 133.8, 132.8, 130.3, 128.9, 128.5, 127.7 (q, *J* = 188.5 Hz), 125.5 (q, *J* = 4.0 Hz), 72.4, 29.8, 17.2. $^{19}$**F NMR (376 MHz, $CDCl_3$-d)** δ – 63.2 (s).

For complete characterization see: B. Landers, C. Berini, C. Wang, O. Navarro, *J. Org. Chem.* **2011**, *76*, 1390–1397.

**2-phenoxy-1-phenyl-propan-1-one (8d)**

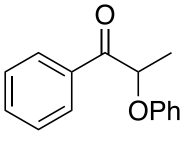

The title compound was synthesized according to the GPD using (*Z*)-*tert*-butyldimethyl((1-phenylprop-1-en-1-yl)oxy)silane (0.1 mmol, 25 mg) as substrate and *p*-trifluoromethylbenzoic acid (0.2 mmol, 20 mg) as nucleophile source. The product was isolated by flash column chromatography (FCC) using silica gel as stationary phase and a gradient of pentane:EtOAc as eluent (5 to 10% of EtOAc) a colourless oil (15 mg, 0.07 mmol, 66%). $^{1}$**H NMR** (400 MHz, $CDCl_3$): δ 8.08 (dd, *J* = 8.5, 1.5 Hz, 2H), 7.63 – 7.54 (m, 1H), 7.51 – 7.43 (m, 2H), 7.26 – 7.20 (m, 2H), 6.93 (tt, *J* = 7.5, 1.0 Hz, 1H), 6.89 – 6.85 (m, 2H), 5.48 (q, *J* = 7.0 Hz, 1H), 1.72 (d, *J* = 7.0 Hz, 3H).$^{13}$**C NMR** (100 MHz, $CDCl_3$): δ = 198.9, 157.4, 134.2, 133.6, 129.6, 128.9, 128.7, 121.4, 115.2, 76.6, 18.7

For complete characterization see: K. Polidano, B. D. W. Allen, J. M. J. Williams, L. C. Morril, *ACS Catal.* **2018**, *8*, 6440–6445.

**1-phenyl-2-(phenylthio)propan-1-one (8e)**

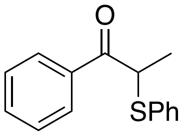

The title compound was synthesized according to the GPC using (*Z*)-*tert*-butyldimethyl((1-phenylprop-1-en-1-yl)oxy)silane (0.1 mmol, 25 mg) as substrate and benzenethiol (0.2 mmol, 22 mg) as nucleophile source. The product was isolated by flash column chromatography (FCC) using silica gel as stationary phase and a gradient of pentane:EtOAc as eluent (5 to 10% of EtOAc) a colourless oil (14 mg, 0.06 mmol, 60%). This compound was also synthetized following GPD (20 mg, 0.08 mmol, 82%). **$^1$H NMR** (400 MHz, $CDCl_3$): δ 7.95 (dd, *J* = 8.5, 1.0 Hz, 2H), 7.60 – 7.53 (m, 1H), 7.48 – 7.42 (m, 2H), 7.35 (dd, *J* = 8.0, 2.0 Hz, 2H), 7.31 – 7.27 (m, 2H), 4.63 (q, *J* = 7.0 Hz, 1H), 1.54 (d, *J* = 7.0 Hz, 3H). **$^{13}$C NMR** (100 MHz, $CDCl_3$): δ = 196.3, 136.6, 135.7, 134.6, 133.1, 128.9, 128.6, 128.6, 128.6, 46.2, 17.0.

For complete characterization see: Yoshikawa, T.; Mori, S.; Shindo, M. *Tetrahedron* **2009**, *65*, 8832.

**1-phenyl-2-(phenylthio)propan-1-one (8f)**

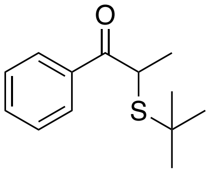

The title compound was synthesized according to the GPD using (*Z*)-*tert*-butyldimethyl((1-phenylprop-1-en-1-yl)oxy)silane (0.1 mmol, 25 mg) as substrate and benzenethiol (0.2 mmol, 18 mg) as nucleophile source. The product was isolated by flash column chromatography (FCC) using silica gel as stationary phase and a gradient of pentane:EtOAc as eluent (5 to 10% of EtOAc) a colourless oil (22 mg, 0.1 mmol, 99%). **$^1$H NMR** (400 MHz, $CDCl_3$): δ 8.02 – 7.99 (m, 2H), 7.60 – 7.54 (m, 1H), 7.51 – 7.46 (m, 2H), 4.37 (q, *J* = 7.0 Hz, 1H), 1.61 (d, *J* = 7.0 Hz, 3H), 1.34 (s, 9H). **$^{13}$C NMR** (100 MHz, $CDCl_3$): δ = 199.6, 135.7, 133.0, 128.7, 128.6, 44.7, 42.4, 31.5, 20.3. **FTIR** $\nu_{max/cm^{-1}}$ (neat) 2958, 2923, 2854, 1679, 1595, 1447, 1230, 950. **HRMS (ESI):** m/z calculated for $[C_{13}H_{18}OSNa]^+$: 245.0971; found: 245.0932.

**2-(benzylamino)-1-phenylpropan-1-one (8g)**

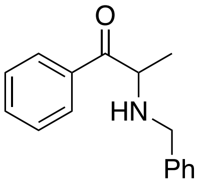

The title compound was synthesized according to the GPD using (*Z*)-*tert*-butyldimethyl((1-phenylprop-1-en-1-yl)oxy)silane (0.1 mmol, 25 mg) as substrate and benzylamine (0.2 mmol, 20 mg) as nucleophile source. The product was isolated by flash column chromatography (FCC) using silica gel as stationary phase and a gradient of pentane:EtOAc as eluent (5 to 10% of EtOAc) a colourless oil (16 mg, 0.07 mmol, 67%). **$^1$H NMR** (400 MHz, $CDCl_3$): δ 7.97 – 7.88 (m, 2H), 7.64 – 7.56 (m, 1H), 7.48 (t, *J* = 7.5 Hz, 2H), 7.39 – 7.24 (m, 5H), 4.35 (q, *J* = 7.0 Hz, 1H), 3.82 (d, *J* = 13.0 Hz, 1H), 3.66 (d, *J* = 13.0 Hz, 1H), 2.19 – 2.02 (bs, 1H), 1.33 (d, *J* = 7.0 Hz, 3H). **$^{13}$C NMR** (100 MHz, $CDCl_3$): δ = 203.6, 139.9, 135.6, 133.4, 128.8, 128.4, 128.4, 128.3, 127.1, 57.1, 52.0, 20.0.

For complete characterization see: Chengkou, L.; Zheng, F.; Zhao, Y.; Qingwen, L.; Shiyu, G.; Kai, G. *RSC Adv*., **2016**, *6*, 25167.

**2-(cyclopropylamino)-1-phenylpropan-1-one (8h)**

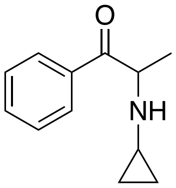

The title compound was synthesized according to the GPC using (*Z*)-*tert*-butyldimethyl((1-phenylprop-1-en-1-yl)oxy)silane (0.1 mmol, 25 mg) as substrate and cyclopropylamine (0.2 mmol, 21 mg) as nucleophile source. The product was isolated by flash column chromatography (FCC) using silica gel as stationary phase and a gradient of pentane:EtOAc as eluent (5 to 10% of EtOAc) a colourless oil (8 mg, 0.04 mmol, 40%). This compound was also synthetized following GPD (11 mg, 0.06 mmol, 60%). **$^1$H NMR** (400 MHz, $CDCl_3$): δ 8.04 – 7.95 (m, 2H), 7.62 – 7.57 (m, 1H), 7.53 – 7.46 (t, *J* = 7.5 Hz, 2H), 4.44 (q, *J* = 7.0 Hz, 1H), 2.16– 2.08 (m, 1H), 1.94 (bs, 1H), 1.29 (d, *J* = 7.0 Hz, 3H). 0.50 – 0.35 (m, 4H). **$^{13}$C NMR** (100 MHz, $CDCl_3$): δ = 203.7, 135.7, 133.3, 128.7, 128.3, 57.9, 28.9, 20.0, 6.6, 6.3. **FTIR** $\nu_{max/cm}^{-1}$ (neat) 1685, 1551, 1418, 1333, 1170, 757, 707. **HRMS (ESI):** m/z calculated for $[C_{12}H_{15}NOH]^+$: 190.1226; found: 190.1231.

**1-phenyl-2-(piperidin-1-yl)propan-1-one (8i)**

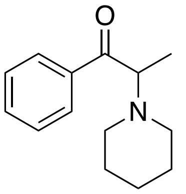

The title compound was synthesized according to the GPC using (*Z*)-*tert*-butyldimethyl((1-phenylprop-1-en-1-yl)oxy)silane (0.1 mmol, 25 mg) as substrate and piperidine (0.2 mmol, 17 mg) as nucleophile source. The product was isolated by flash column chromatography (FCC) using silica gel as stationary phase and a gradient of pentane:EtOAc as eluent (5 to 10% of EtOAc) a colourless oil (12 mg, 0.05 mmol, 53%). This compound was also synthetized following GPD (17 mg, 0.08 mmol, 77%). **$^1$H NMR** (400 MHz, $CDCl_3$): δ 8.29 – 8.00 (m, 2H), 7.56 – 7.51 (m, 1H), 7.47 – 7.40 (m, 2H), 4.07 (q, *J* = 7.0 Hz, 1H), 2.66 – 2.42 (m, 4H), 1.59 – 1.48 (m, 4H), 1.41 (q, *J* = 6.5 Hz, 2H), 1.26 (d, *J* = 7.0 Hz, 3H).**$^{13}$C NMR** (100 MHz, $CDCl_3$): δ =198.1, 154.7, 134.8, 133.3, 128.7, 128.6, 128.5, 71.8, 45.0, 29.7, 24.4, 17.2.

For complete characterization see: Lamani, M.; Prabhu, K. R. Chem. Eur. J., **2012**, 12, 14638.

**2-morpholino-1-phenylpropan-1-one (8j)**

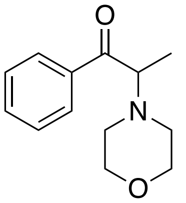

The title compound was synthesized according to the GPC using (*Z*)-*tert*-butyldimethyl((1-phenylprop-1-en-1-yl)oxy)silane (0.1 mmol, 25 mg) as substrate and morpholine (0.2 mmol, 17 mg) as nucleophile source. The product was isolated by flash column chromatography (FCC) using silica gel as stationary phase and a gradient of pentane:EtOAc as eluent (5 to 10% of EtOAc) a colourless oil (13 mg, 0.06 mmol, 60%). This compound was also synthetized following GPD (17 mg, 0.08 mmol, 80%). **$^1$H NMR** (400 MHz, $CDCl_3$) δ 8.13 – 8.09 (m, 2H), 7.60 – 7.53 (m, 1H), 7.50 – 7.42 (m, 1H), 4.08 (q, *J* = 7.0 Hz, 1H), 3.75 – 3.62 (m, 4H), 2.70 – 2.52 (m, 2H), 1.30 (d, *J* = 7.0 Hz, 3H). **$^{13}$C NMR** (100 MHz, $CDCl_3$): δ = 200.2, 136.3, 133.0, 128.8, 128.4, 67.1, 64.8, 50.0, 11.6.

For complete characterization see: Lamani, M.; Prabhu, K. R. Chem. Eur. J., **2012**, 12, 14638.

**dimethyl 2-(1-oxo-1-phenylpropan-2-yl)malonate (8k)**

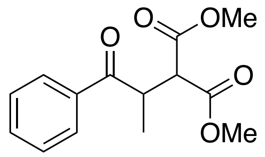

The title compound was synthesized according to the GPC using (*Z*)-*tert*-butyldimethyl((1-phenylprop-1-en-1-yl)oxy)silane (0.1 mmol, 25 mg) as substrate and dimethylmalonate (0.2 mmol, 26 mg) as nucleophile source. The product was isolated by flash column chromatography (FCC) using silica gel as stationary phase and a gradient of pentane:EtOAc as eluent (5 to 10% of EtOAc) a colourless oil (13 mg, 0.05 mmol, 50%). This compound was also synthetized following GPD (18 mg, 0.07 mmol, 72%). **$^1$H NMR** (400 MHz, $CDCl_3$): δ 8.04 – 7.91 (m, 2H), 7.62 – 7.53 (m, 1H), 7.52 – 7.43 (m, 2H), 4.20 (dq, *J* = 11.0, 7.0 Hz, 1H), 4.02 (d, *J* = 11.0 Hz, 1H), 3.80 (s, 3H), 3.65 (s, 3H), 1.19 (d, *J* = 7.0 Hz, 3H).**$^{13}$C NMR** (100 MHz, $CDCl_3$): δ = 201.5, 169.1, 168.8, 135.5, 133.3, 128.7, 128.5, 54.5, 52.7, 40.7, 15.9.

For complete characterization see: Liu, J.; Vasamsetty, L.; Anwar, M.; Yang, S.; Xu, W.; Liu, J.; Nagaraju, S.; Fang, X. *ACS Catal.* **2020**, *10*, 2882.

**2-azido-1-phenylpropan-1-one (8l)**

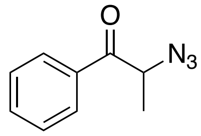

The title compound was synthesized according to the GPC using (Z)-tert-butyldimethyl((1-phenylprop-1-en-1-yl)oxy)silane (0.1 mmol, 25 mg) as substrate and diphenylphosphoryl azide (0.2 mmol, 26 mg) as nucleophile source. The product was isolated by flash column chromatography (FCC) using silica gel as stationary phase and a gradient of pentane:EtOAc as eluent (5 to 10% of EtOAc) a colourless oil (9 mg, 0.05 mmol, 50%). This compound was also synthetized following GPD (13 mg, 0.07 mmol, 70%). **$^1$H NMR** (400 MHz, $CDCl_3$): δ 8.08 – 7.68 (m, 2H), 7.71 – 7.55 (m, 1H), 7.58 – 7.47 (m, 2H), 4.71 (q, *J* = 7.0 Hz, 1H), 1.58 (d, *J* = 7.0 Hz, 3H). **$^{13}$C NMR** (100 MHz, $CDCl_3$): δ = 196.8, 134.4, 134.0, 129.0, 128.8, 58.5, 16.6.

For complete characterization see: Shibatomi, K., Kitahara, K., Sasaki, N., Kawasaki, Y., Fujisawa, I., Iwasa. S. *Nat. Commun*, **2017**, *8*, 15600.

**1-oxo-1-phenylpropan-2-yl (tert-butoxycarbonyl)glycinate (8m)**

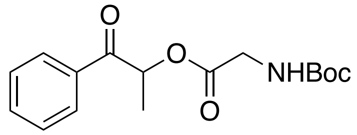

The title compound was synthesized according to the GPC using (*Z*)-*tert*-butyldimethyl((1-phenylprop-1-en-1-yl)oxy)silane (0.1 mmol, 25 mg) as substrate and (tert-butoxycarbonyl)glycine (0.2 mmol, 36 mg) as nucleophile source. The product was isolated by flash column chromatography (FCC) using deactivated silica gel (silica in a 1% $Et_3N$ solution in DCM overnight) as stationary phase and a gradient of pentane:EtOAc as eluent (10% of EtOAc) a colourless oil (17 mg, 0.06 mmol, 55%) with a 90% purity (impurity belonging to $NEt_3$ ). **$^1$H NMR** (400 MHz, $CDCl_3$): δ 7.95 – 7.90 (m, 2H), 7.66 – 7.57 (m, 1H), 7.49 (dd, *J* = 8.5, 7.0 Hz, 2H), 6.03 (q, *J* = 7.0 Hz, 1H), 4.97 (bs, 1H), 4.11 (dd, *J* = 18.5, 6.5 Hz, 1H), 3.96 (dd, *J* = 18.0, 5.0 Hz, 1H), 1.56 (d, J = 7.0 Hz, 3H), 1.44 (s, 9H).**$^{13}$C NMR** (100 MHz, $CDCl_3$): δ = 196.3, 169.9, 134.2, 133.8, 128.9, 128.5, 77.2, 72.3, 45.7, 28.3, 17.2. **FTIR** $\nu_{max/cm^{-1}}$ (neat) 2256, 2925, 1694, 1595, 1449, 1227, 907, 700. **HRMS (ESI):** m/z calculated for $[C_{16}H_{21}NO_5SNa]^+$: 330.1312; found: 330.1292.

**1-oxo-1-phenylpropan-2-yl 5-((3aS,4S,6aR)-2-oxohexahydro-1H-thieno[3,4-d]imidazol-4-yl)pentanoate (8n)**

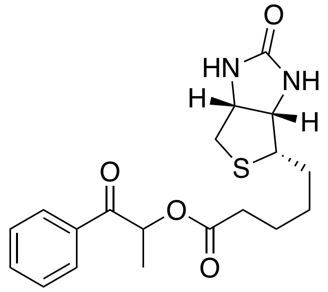

The title compound was synthesized according to the GPC using (*Z*)-*tert*-butyldimethyl((1-phenylprop-1-en-1-yl)oxy)silane (0.1 mmol, 25 mg) as substrate and biotin (0.2 mmol, 48 mg) as nucleophile source. The product was isolated by flash column chromatography (FCC) using silica gel as stationary phase and a gradient of pentane:EtOAc as eluent (100% of EtOAc) a white solid (16 mg, 0.04 mmol, 40%) as a mixture of diastereomers (1:1 mixture) (90% purity). **$^1$H NMR** (400 MHz, $CDCl_3$): δ 7.92 (ddd, *J* = 8.5, 6.0, 1.5 Hz, 2H), 7.63 – 7.53 (m, 1H), 7.53 – 7.40 (m, 2H), 6.25 (bs, 1H), 6.05 – 5.90 (m, 1H), 5.61 (bs, 1H), 4.55 – 4.42 (m, 1H), 4.33 – 4.22 (m, 1H), 3.21 – 3.05 (m, 1H), 2.95 – 2.83 (m, 1H), 2.72 (d, *J* = 13.0 Hz, 1H), 2.56 – 2.29 (m, 2H), 1.81 – 1.61 (m, 4H), 1.61 – 1.36 (m, 5H).**$^{13}$C NMR** (100 MHz, $CDCl_3$): δ = 197.3, 197.2, 173.2, 173.2, 163.8, 134.3, 134.3, 133.7, 128.8, 128.8, 128.5, 128.5, 71.3, 61.9, 61.8, 60.2, 55.6, 55.5, 40.6, 40.5, 33.6, 33.6, 28.2, 28.1, 28.1, 24.8, 24.7, 17.2. **FTIR** $\nu_{max/cm^{-1}}$ (neat) 2966, 2360, 2335, 1769, 1732, 1698, 1196, 701. **HRMS (ESI):** m/z calculated for $[C_{19}H_{24}N_2O_4SNa]^+$: 399.1349; found: 399.1381.

**1-oxo-1-phenylpropan-2-yl 2-acetoxybenzoate (8o)**

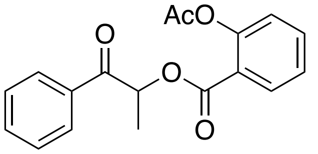


The title compound was synthesized according to the GPC using (Z)-tert-butyldimethyl((1-phenylprop-1-en-1-yl)oxy)silane (0.1 mmol, 25 mg) as substrate and acerylsalicylic acid (0.2 mmol, 36 mg) as nucleophile source. The product was isolated by flash column chromatography (FCC) using silica gel as stationary phase and a gradient of pentane:EtOAc as eluent (5 to 10% of EtOAc) a colourless oil (31mg, 0.1 mmol, 98%). **$^1$H NMR** (400 MHz, $CDCl_3$): δ 8.10 (dd, *J* = 8.0, 1.5 Hz, 1H), 8.03 – 7.92 (m, 2H), 7.66 – 7.53 (m, 2H), 7.53 – 7.44 (m, 2H), 7.32 (td, *J* = 8.0, 1.5 Hz, 1H), 7.11 (dd, *J* = 8.0, 1.5 Hz, 1H), 6.20 (q, *J* = 7.0 Hz, 1H), 2.32 (s, 3H), 1.63 (d, *J* = 7.0 Hz, 3H). **$^{13}$C NMR** (100 MHz, $CDCl_3$): δ = 196.4, 169.7, 163.9, 150.8, 134.5, 134.2, 133.7, 132.1, 128.9, 128.6, 128.6, 126.1, 123.9, 123.0, 71.9, 21.1, 17.3. **FTIR** $\nu_{max/cm}^{-1}$ (neat) 3065, 2963, 2360, 2335, 1769, 1718, 1697, 1194, 1090. **HRMS (ESI):** m/z calculated for $[C_{18}H_{16}O_4K]^+$: 335.0680; found: 335.0599.

**1-oxo-1-phenylpropan-2-yl 2-(4-isobutylphenyl)propanoate (8p)**

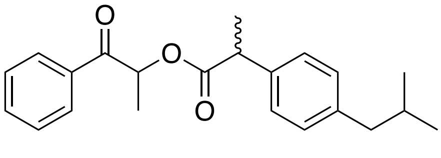


The title compound was synthesized according to the GPC using (*Z*)-*tert*-butyldimethyl((1-phenylprop-1-en-1-yl)oxy)silane (0.1 mmol, 25 mg) as substrate and 2-(4-isobutylphenyl)propanoic acid (0.2 mmol, 41 mg) as nucleophile source. The product was isolated by flash column chromatography (FCC) using silica gel as stationary phase and a gradient of pentane:EtOAc as eluent (5 to 10% of EtOAc) a colourless oil (30 mg, 0.09 mmol, 89%) as a 1:1 mixture of diastereomers. **$^1$H NMR** (400 MHz, $CDCl_3$): δ 8.04 – 7.98 (m, 1H), 7.94 – 7.89 (m, 1H), 7.64 (dt, *J* = 22.0, 7.5 Hz, 1H), 7.55 (t, *J* = 7.5 Hz, 1H), 7.47 (t, *J* = 7.5 Hz, 1H), 7.29 (dd, *J* = 16.5, 8.0 Hz, 2H), 7.17 (dd, *J* = 13.0, 8.0 Hz, 2H), 6.05 – 5.97 (m, 1H), 3.98 – 3.83 (m, 1H), 2.62 – 2.50 (m, 2H), 1.65 – 1.54 (m, 6H), 1.00 (d, *J* = 7.0 Hz, 6H). **$^{13}$C NMR** (100 MHz $CDCl_3$): δ 197.1, 196.7, 173.3, 174.0, 140.6, 140.5, 140.3, 138.2, 137.3, 136.9, 134.5, 134.5, 1 33.5, 133.3, 129.3, 129.3, 129.3, 129.2, 128.7, 128.6, 128.5, 128.4, 127.3, 127.3, 71.8, 71.6,

45.1, 45.1, 44.9, 44.7, 30.2, 30.2, 25.4, 22.4, 22.4, 22.4, 18.6, 18.5, 16.9, 16.8. **FTIR** $\nu_{max/cm}^{-1}$ (neat) 3272, 2929, 2860, 1696, 1545, 1450, 1229, 1090, 701. **HRMS (ESI):** m/z calculated for $[C_{22}H_{26}O_3Na]^+$: 361.1774; found: 361.1721

**1-oxo-1-phenylpropan-2-yl oleate (8q)**

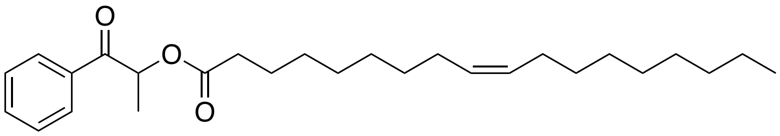

The title compound was synthesized according to the GPC using (*Z*)-*tert*-butyldimethyl((1-phenylprop-1-en-1-yl)oxy)silane (0.1 mmol, 25 mg) as substrate and oleic acid (0.2 mmol, 56 mg) as nucleophile source. The product was isolated by flash column chromatography (FCC) using silica gel as stationary phase and a gradient of pentane:EtOAc as eluent (5 to 10% of EtOAc) a colourless oil (29 mg, 0.07 mmol, 70%). **$^1$H NMR** (400 MHz, $CDCl_3$): δ 8.04 – 7.91 (m, 2H), 7.59 (t, *J* = 7.5 Hz, 1H), 7.47 (t, *J* = 7.5 Hz, 2H), 5.97 (q, *J* = 7.0 Hz, 1H), 5.43 – 5.22 (m, 2H), 2.39 (td, *J* = 7.5, 2.5 Hz, 2H), 2.01 (q, *J* = 6.5 Hz, 3H), 1.64 (q, *J* = 7.5 Hz, 2H), 1.52 (d, *J* = 7.0 Hz, 3H), 1.34 – 1.23 (m, 20H), 0.88 (t, *J* = 6.5 Hz, 3H).**$^{13}$C NMR** (100 MHz,$CDCl_3$): δ 197.2, 173.4, 134.6, 133.6, 130.1, 129.9, 128.8, 128.6, 71.3, 34.1, 32.0, 29.9, 29.9, 29.8, 29.7, 29.5, 29.3, 29.2, 29.2, 27.4, 27.3, 24.5, 22.8, 17.2, 14.2. **FTIR** $\nu_{max/cm}^{-1}$ (neat) 2925, 1694, 1647, 1448, 1598, 1254, 1224, 1112, 1069, 967, 708. **HRMS (ESI):** m/z calculated for $[C_{27}H_{42}O_3Na]^+$: 437.3026; found: 437.3017.

## **$^1$H NMR and $^{13}$C NMR of products**

## 1-Oxo-1-phenylpropan-2-yl diethylcarbamate (5a)

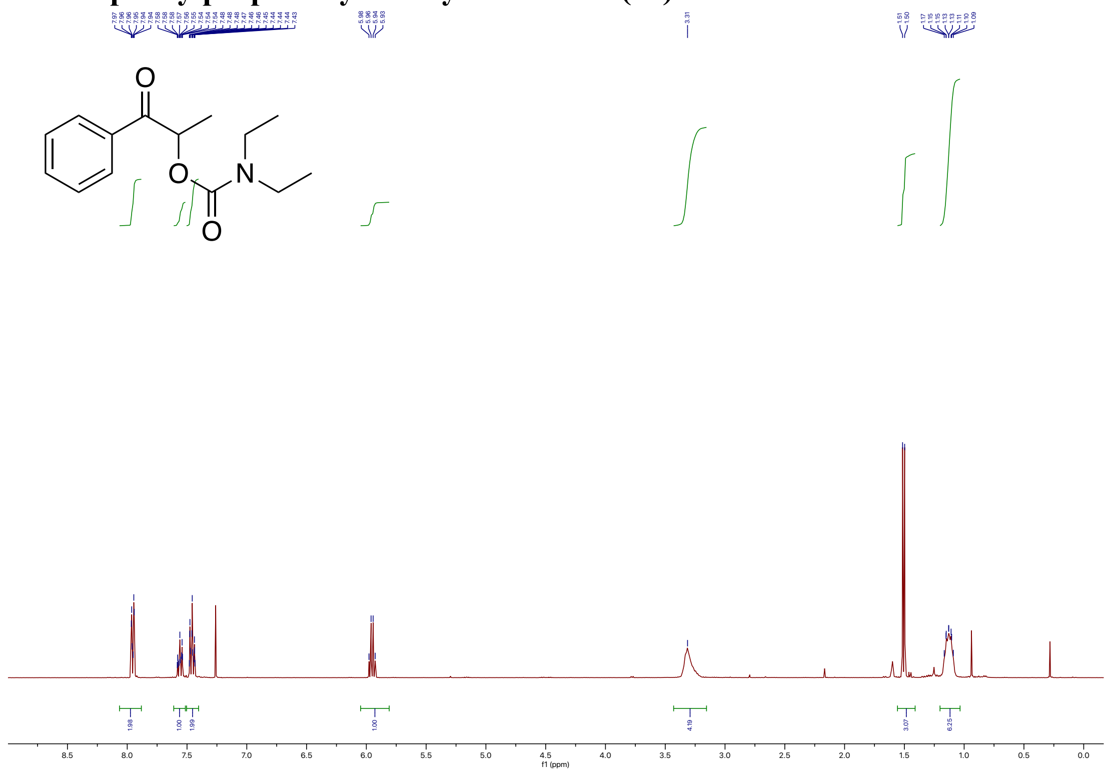


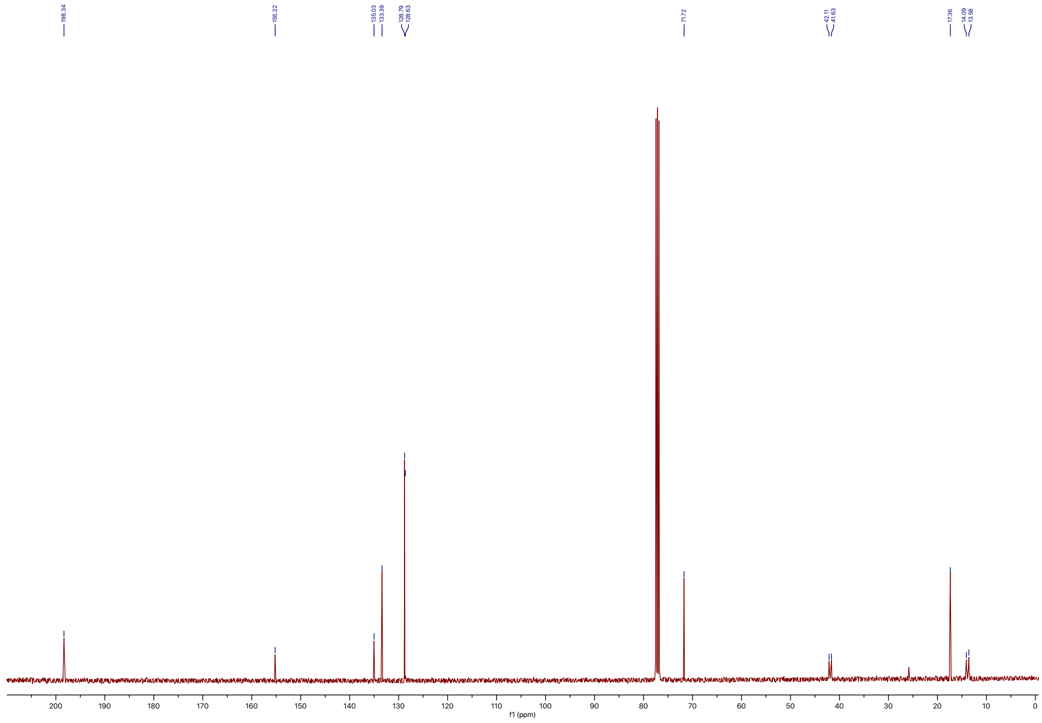


## 1-(4-Methoxyphenyl)-1-oxopropan-2-yl diethylcarbamate (5b)

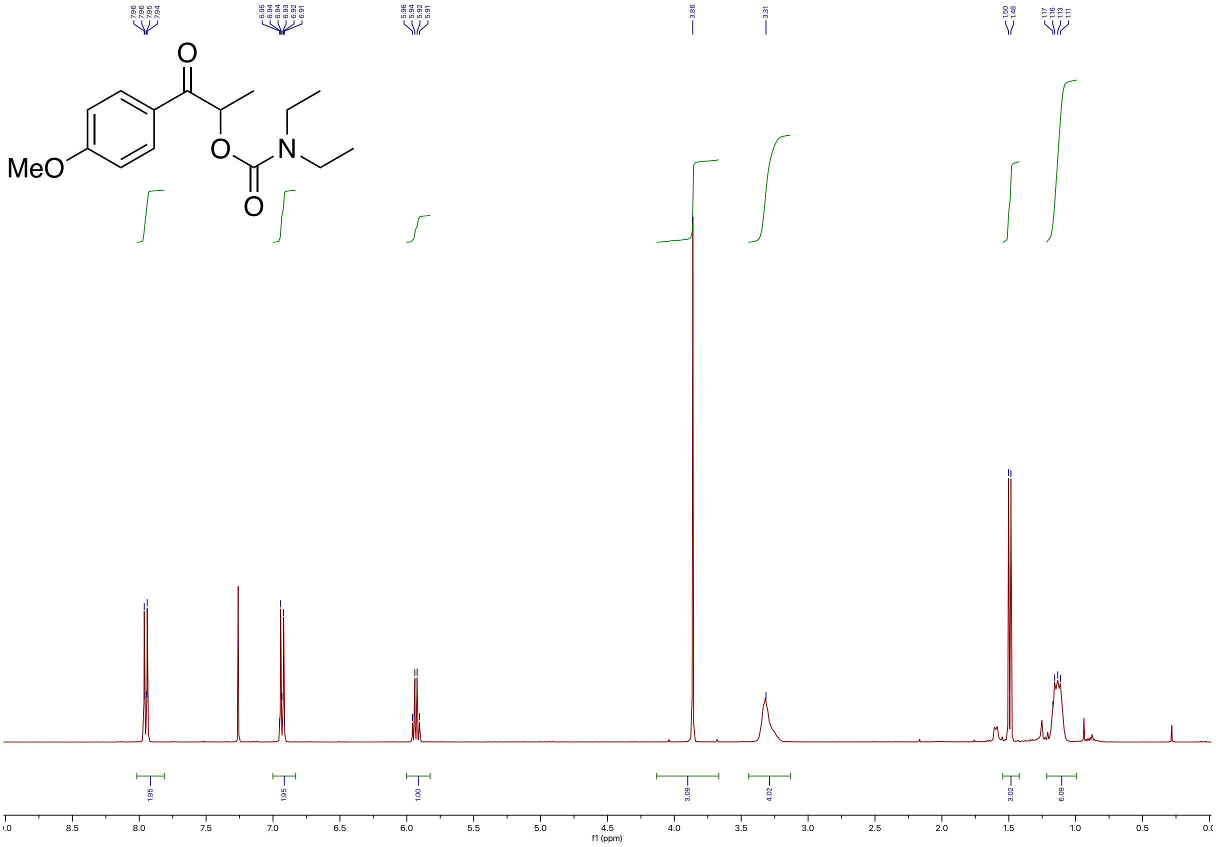

7.96
7.96
7.95
7.94
6.95
6.94
6.94
6.93
6.92
6.91
5.96
5.94
5.92
5.91
3.86
3.31
1.50
1.48
1.17
1.16
1.13
1.11
MeO
O
O
N
O
1.95
1.95
1.00
3.09
4.02
3.02
6.09
f1 (ppm)


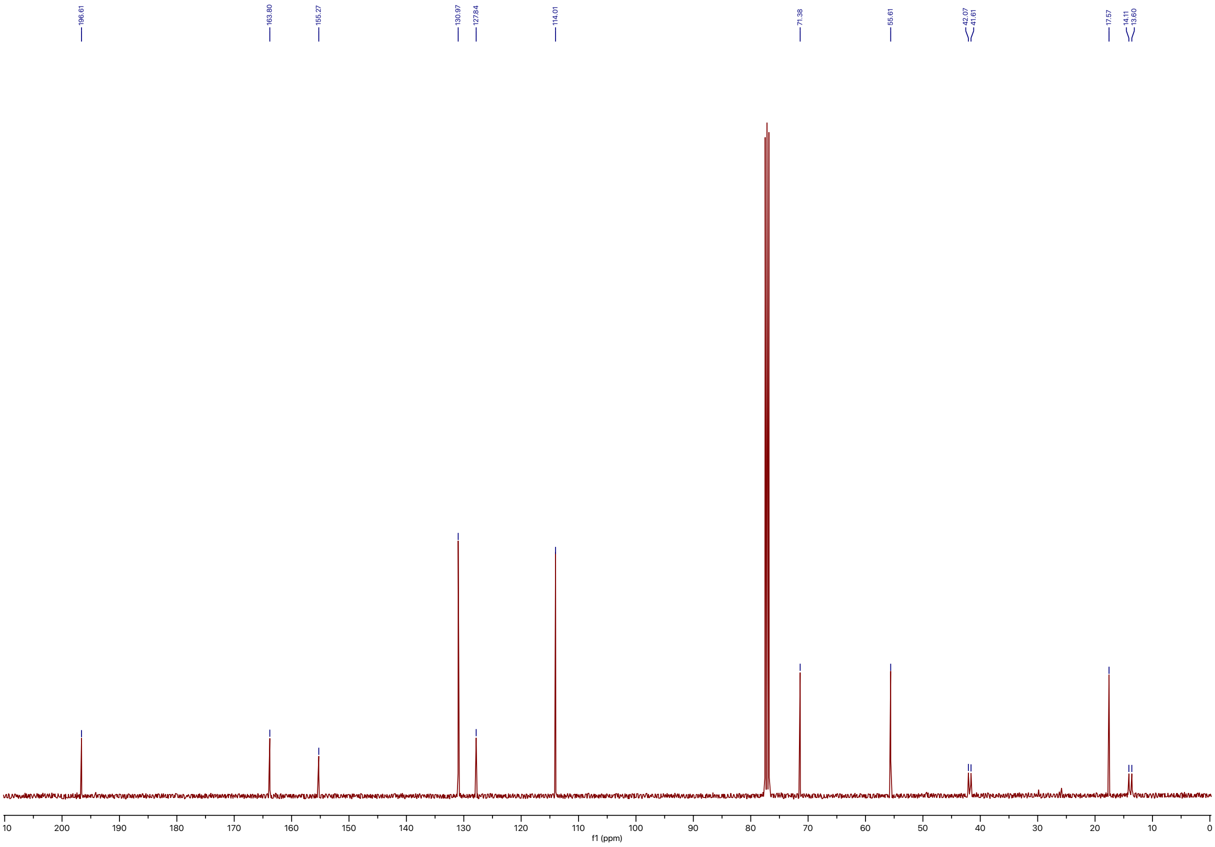

196.61
163.80
155.27
130.97
127.84
114.01
71.38
55.61
42.07
41.61
17.57
14.11
13.60
f1 (ppm)

## 1-Oxo-1-(*p*-tolyl)propan-2-yl diethylcarbamate (5c)

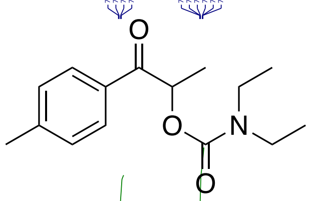

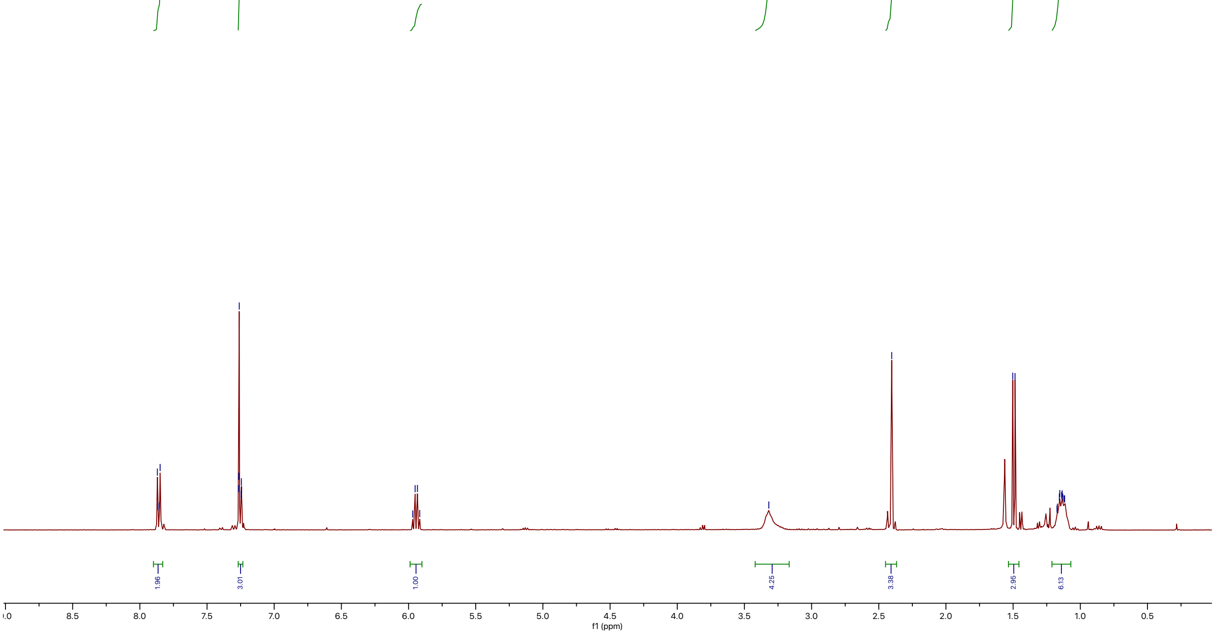

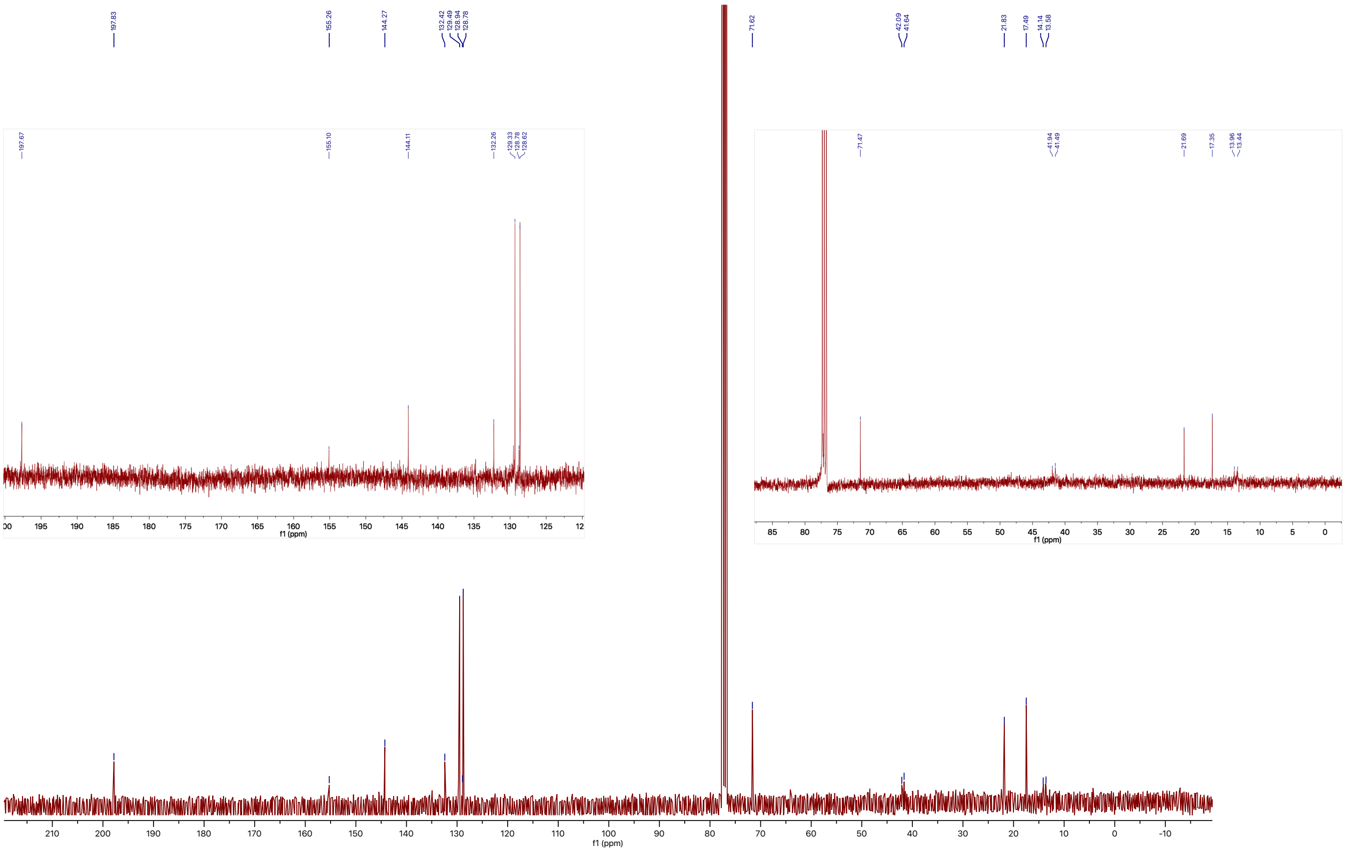

## 1-(4-fluorophenyl)-1-oxopropan-2-yl diethylcarbamate (5d)

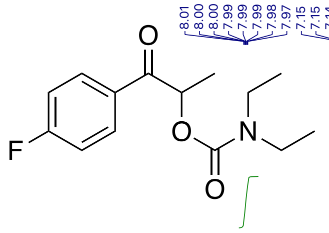

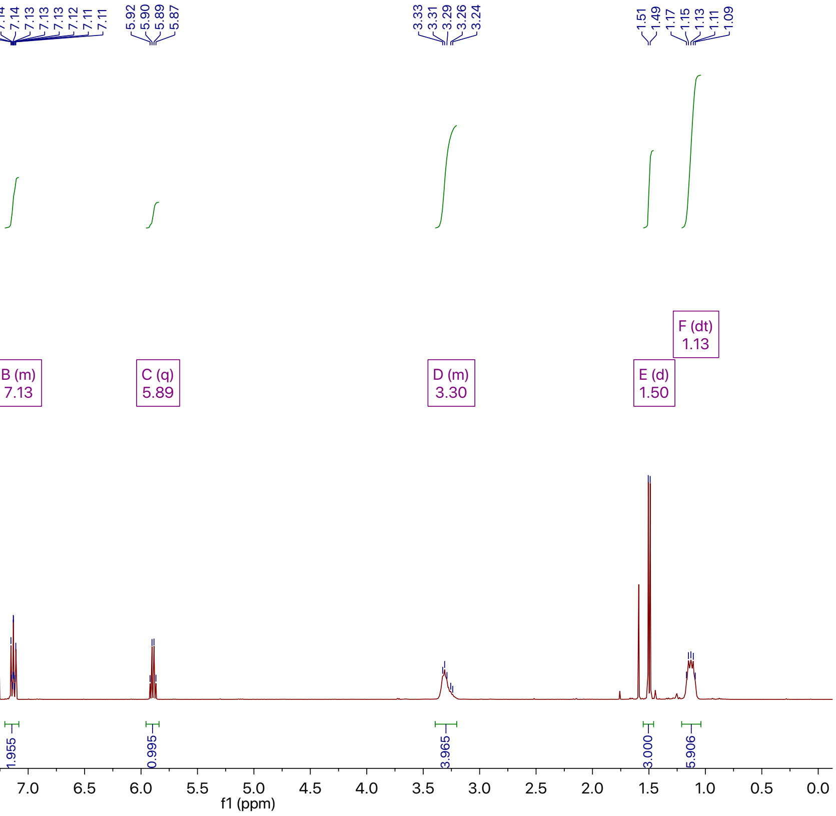


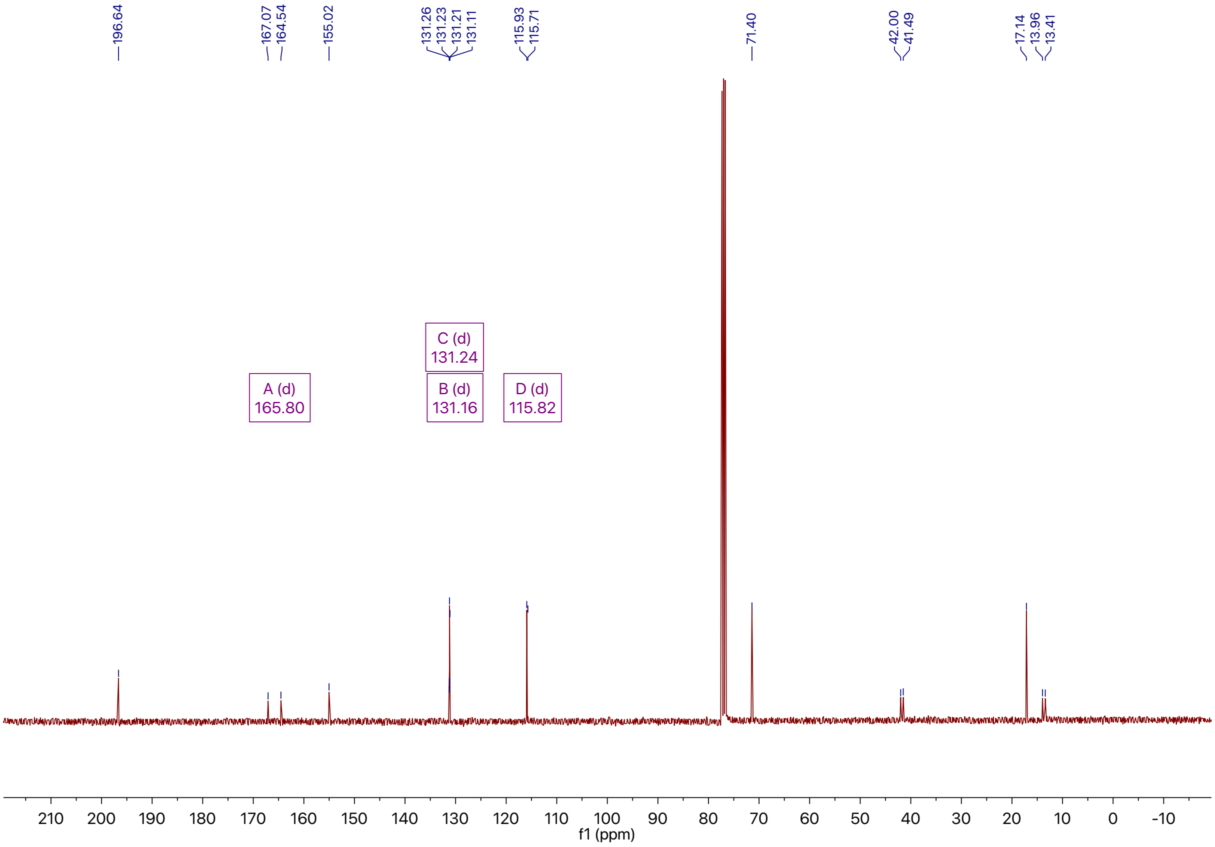

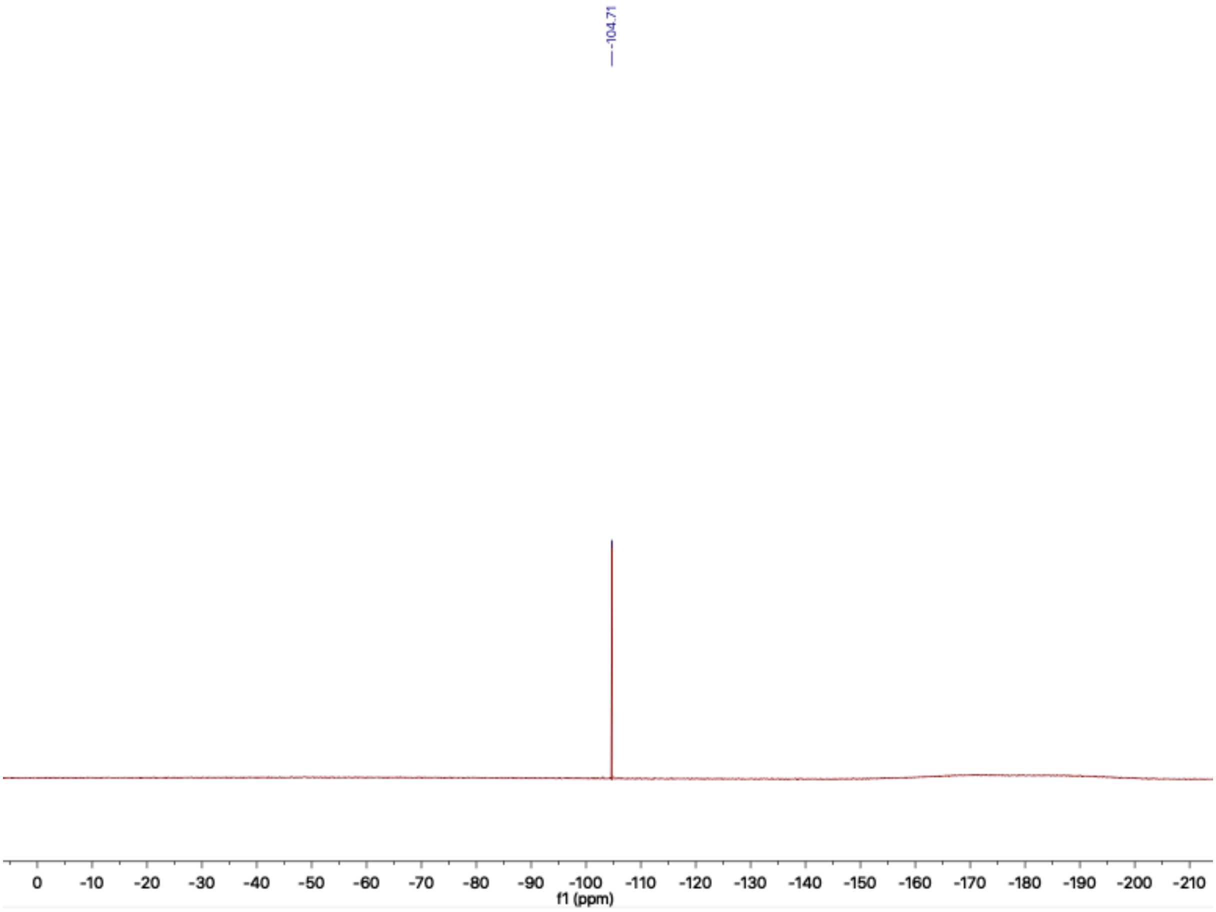
-104.71
0 -10 -20 -30 -40 -50 -60 -70 -80 -90 -100 -110 -120 -130 -140 -150 -160 -170 -180 -190 -200 -210
f1 (ppm)

**1-oxo-1-(thiophen-2-yl)propan-2-yl diethylcarbamate (5e)**

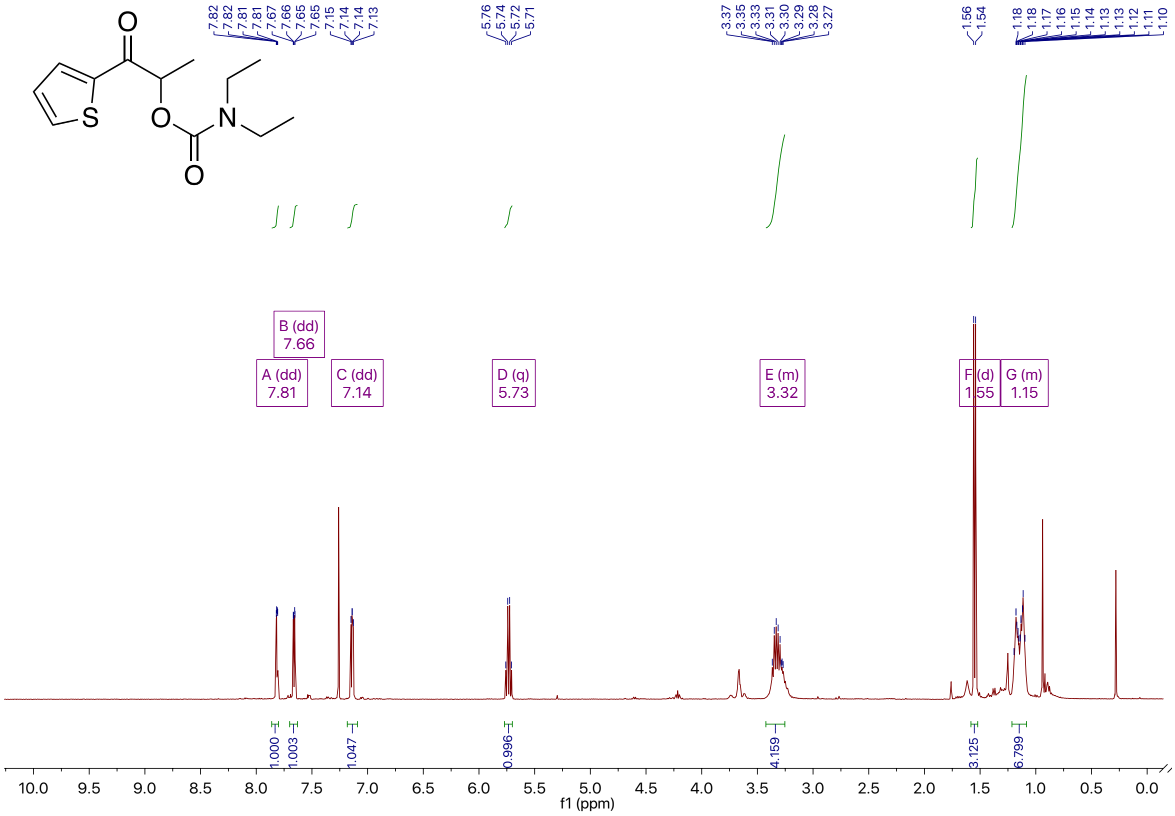

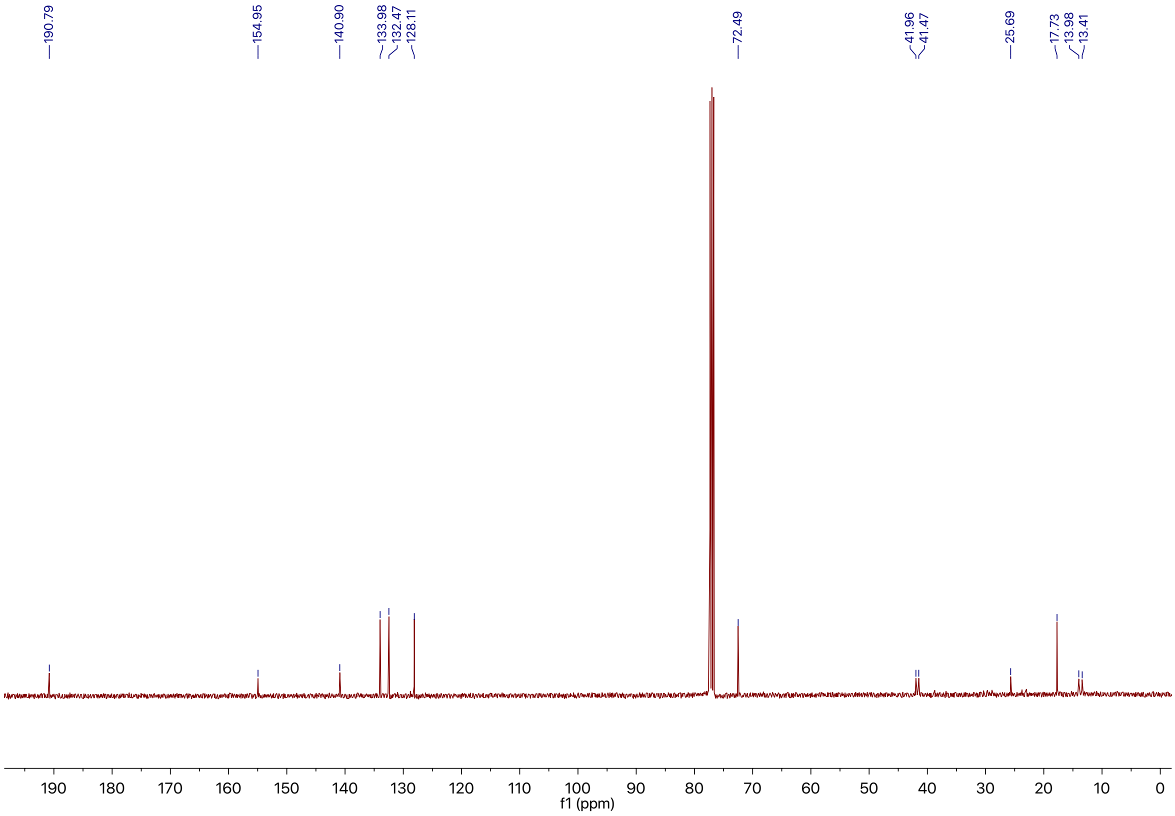

## 1-oxo-1-phenylbutan-2-yl diethylcarbamate (5f)

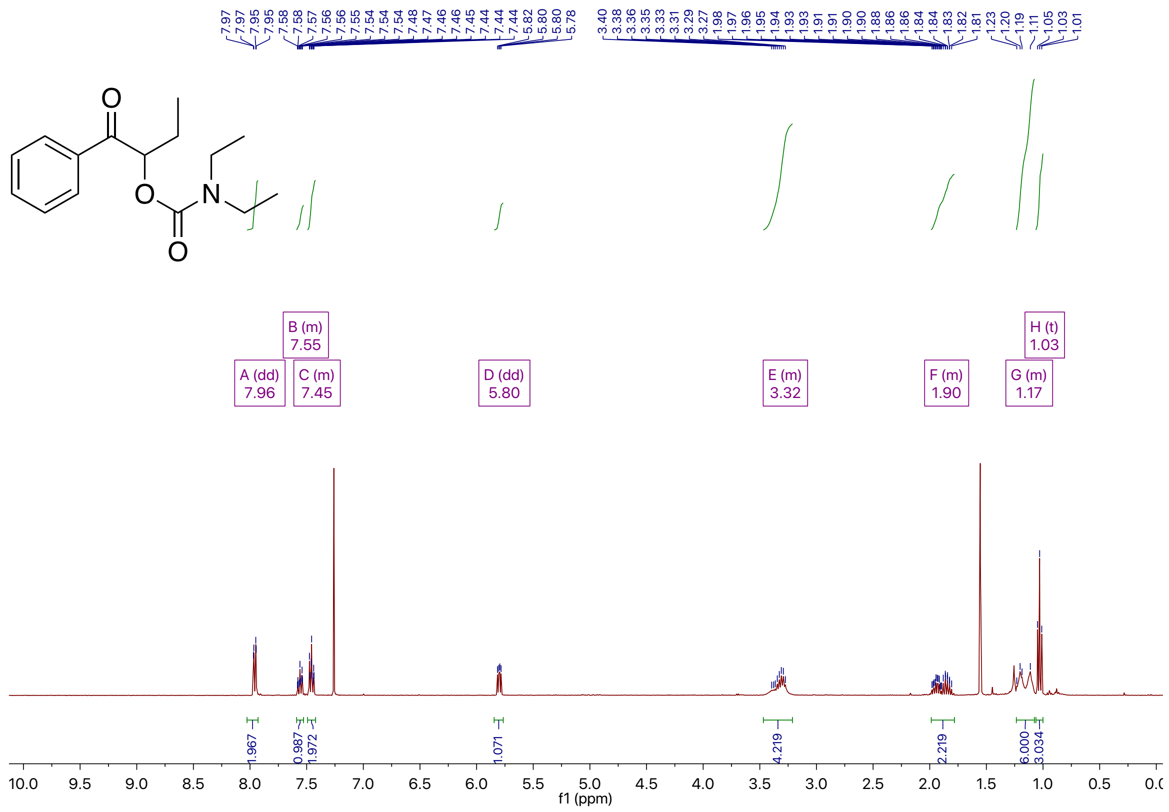

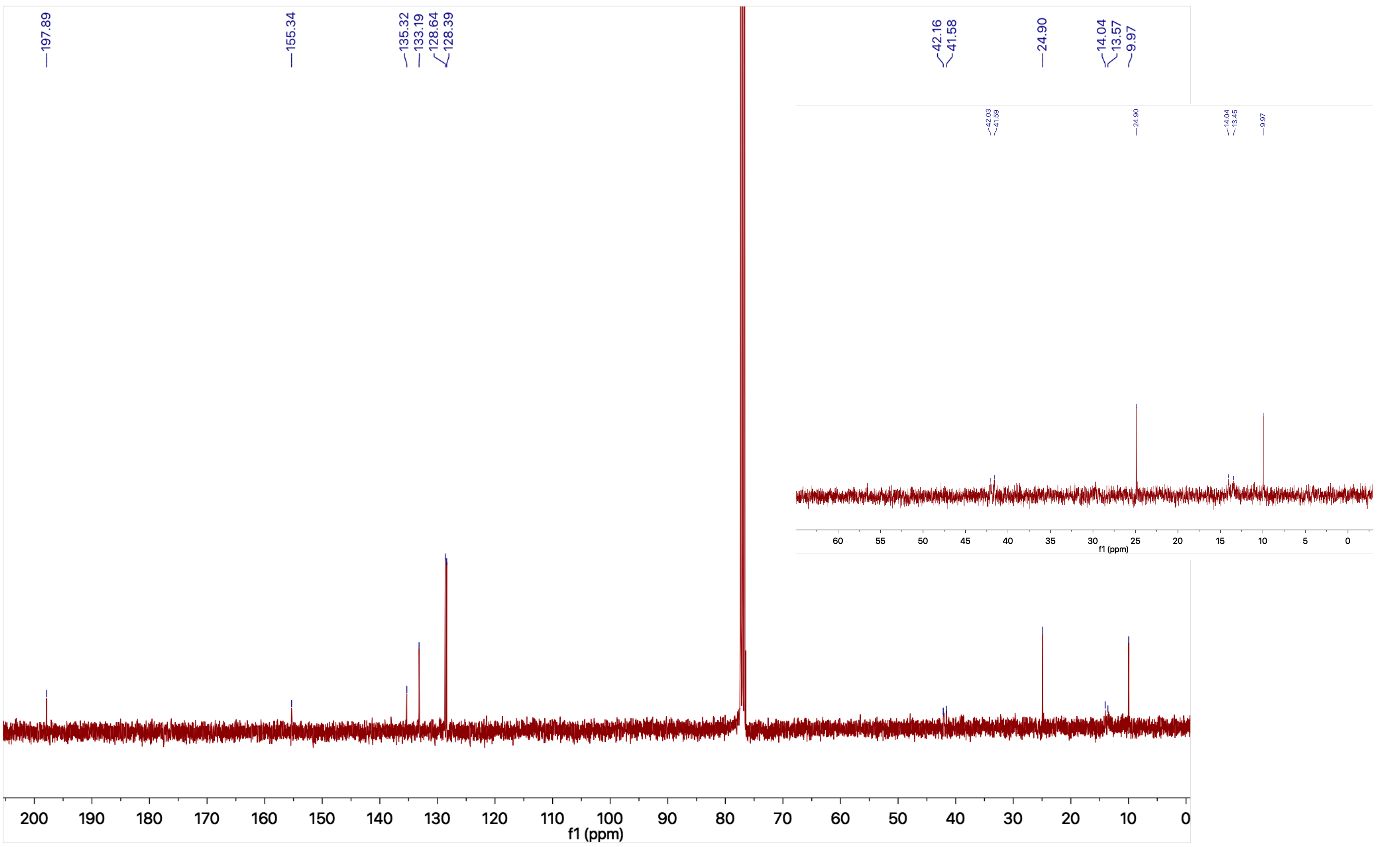

## 4-oxoheptan-3-yl diethylcarbamate (5g)

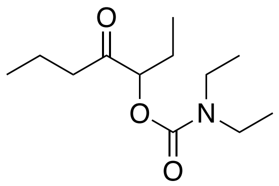

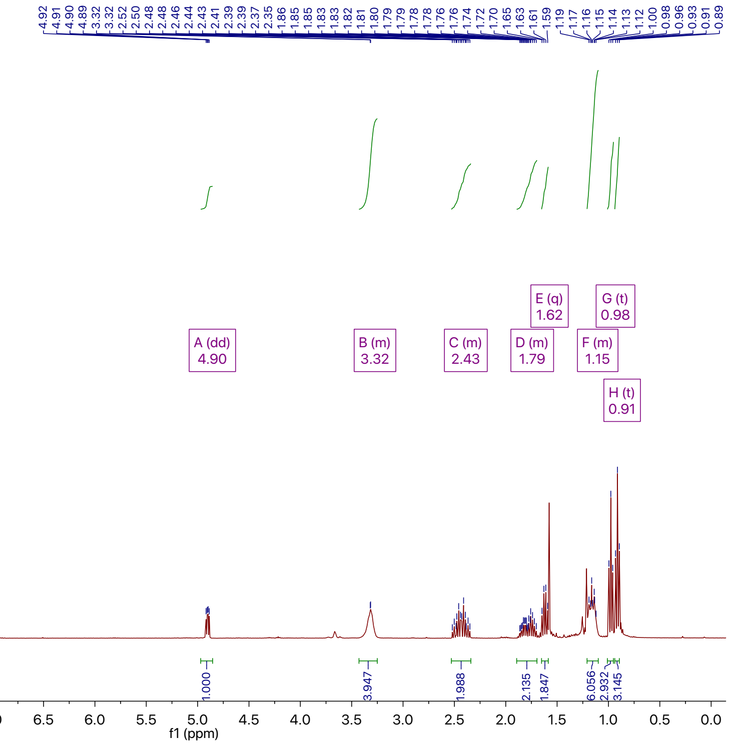

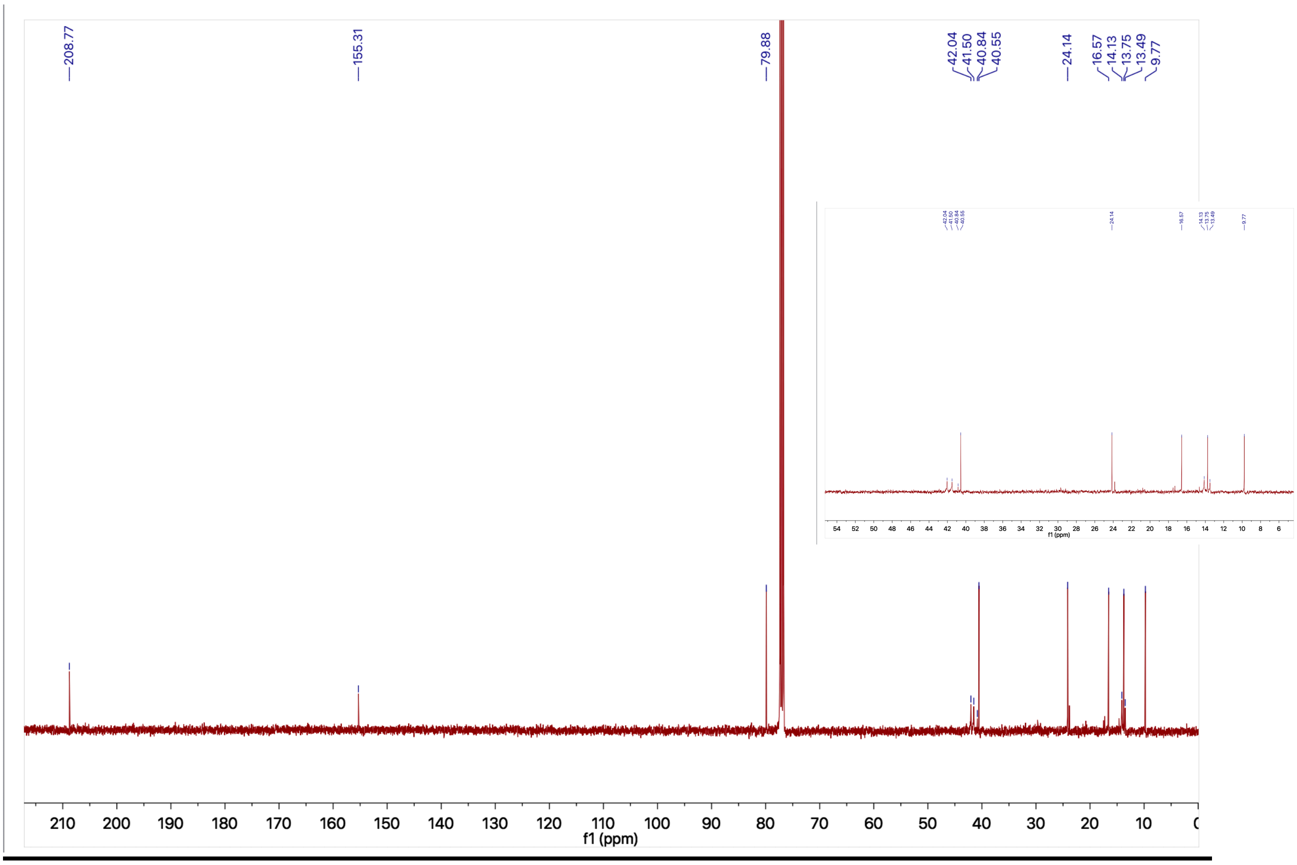

**5-oxononan-4-yl diethylcarbamate (5h)**

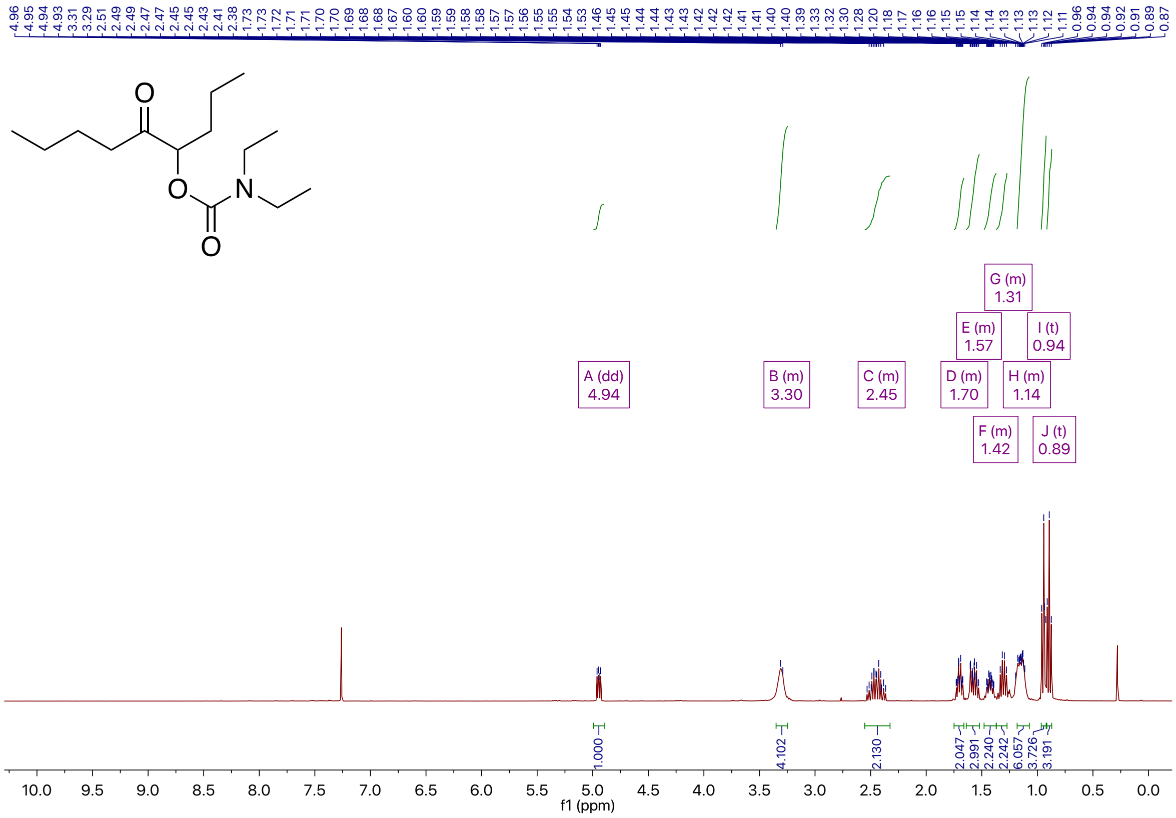

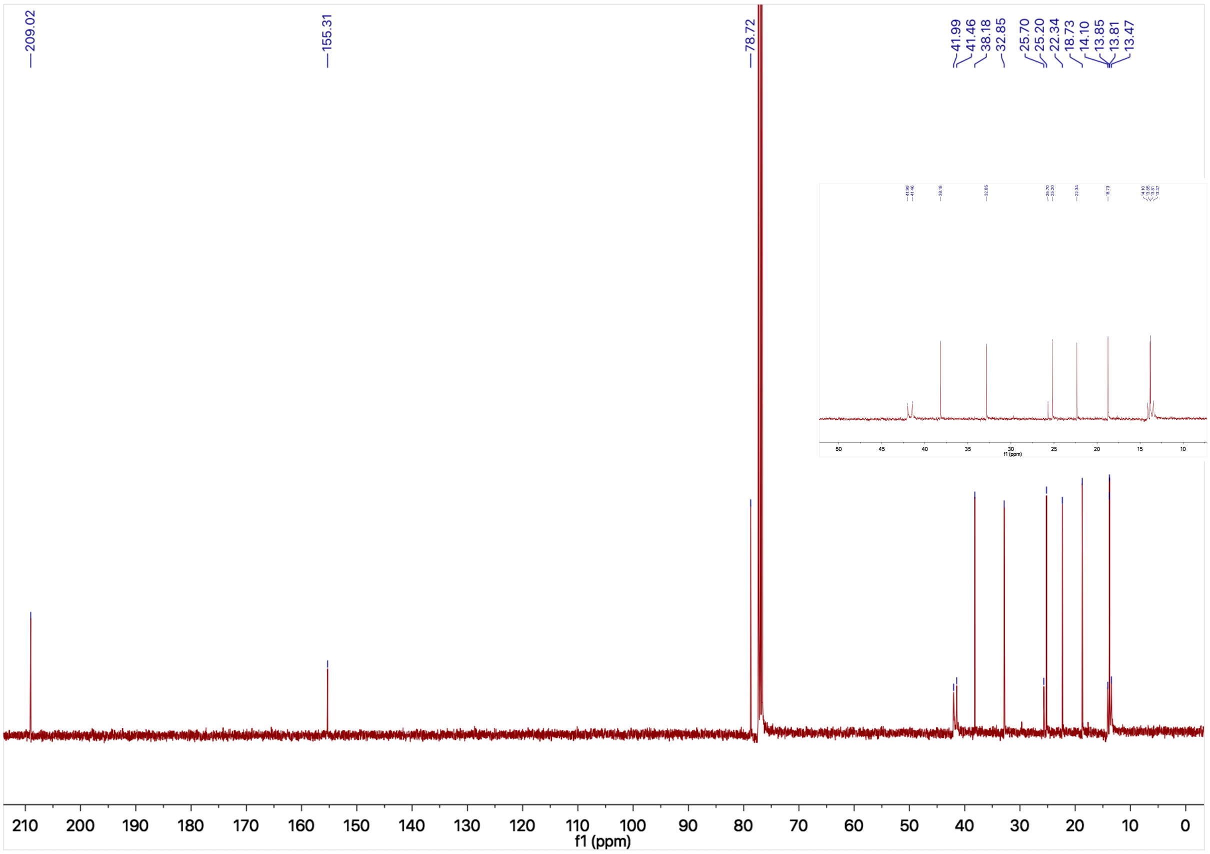

## 1-oxo-1-phenylpropan-2-yl benzyl(methyl)carbamate (5i)

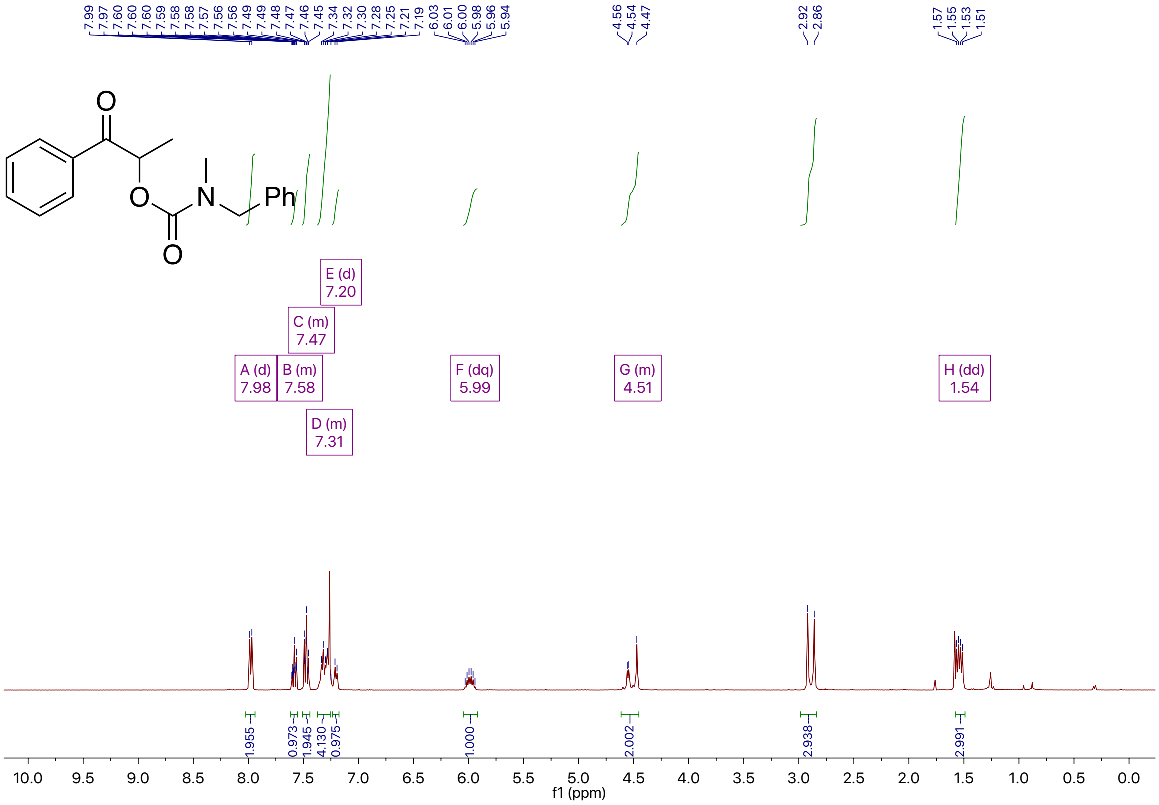

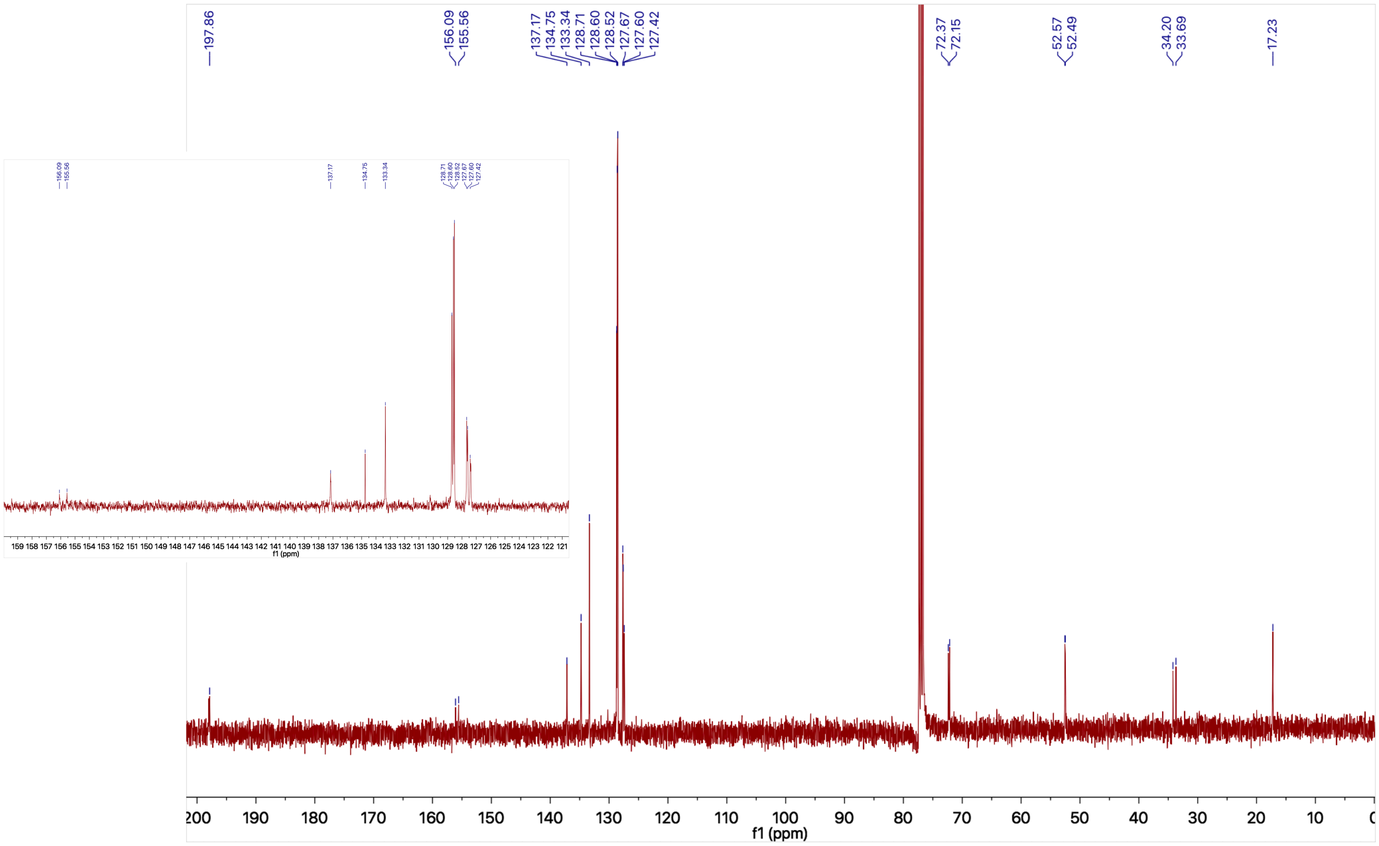

**1-oxo-1-phenylpropan-2-yl dibenzylcarbamate (5j)**

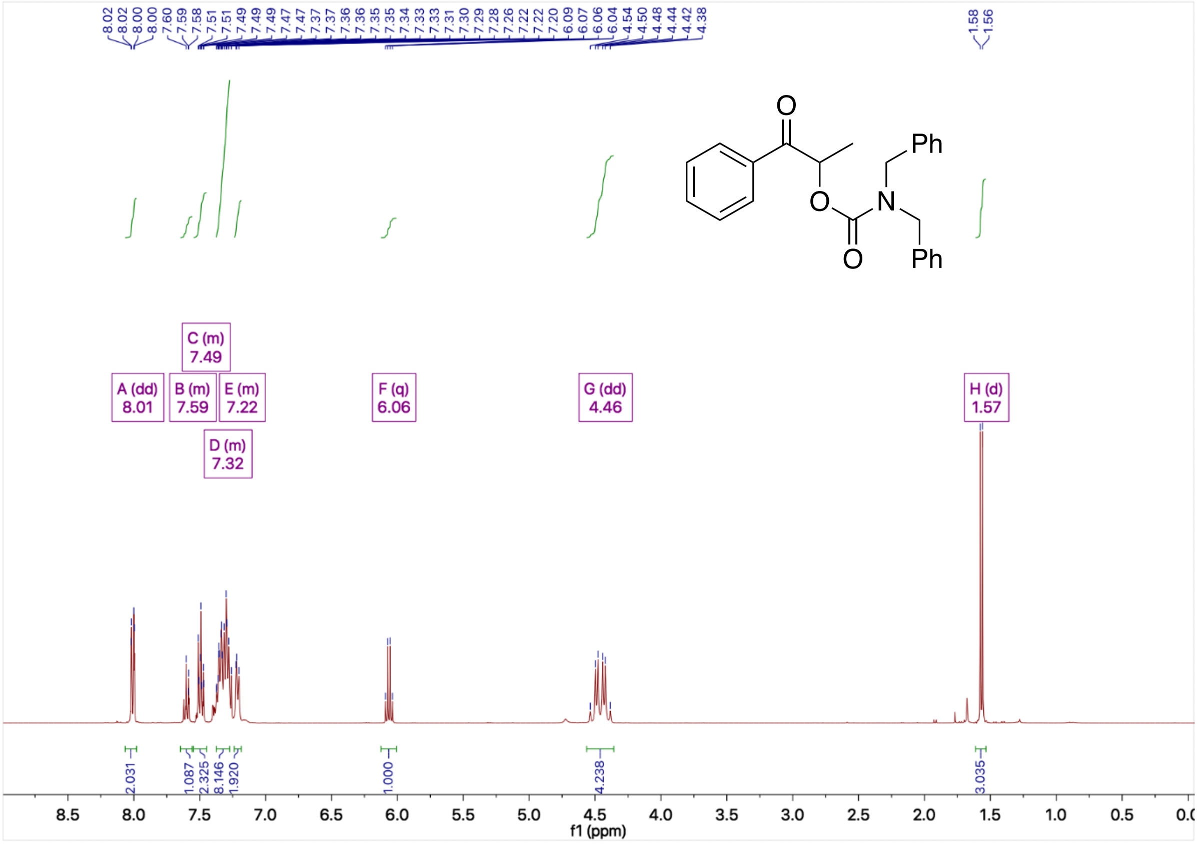

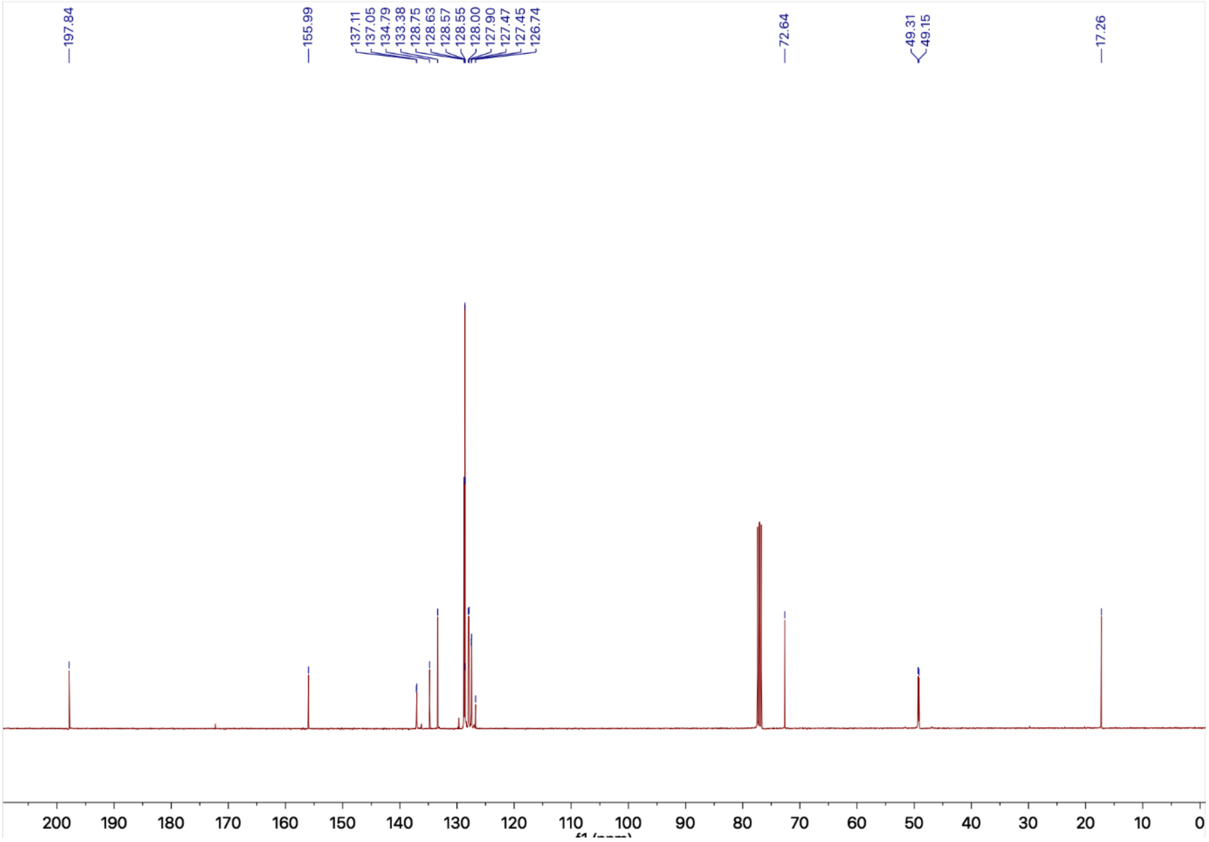

**1-oxo-1-phenylpropan-2-yl diisobutylcarbamate (5k)**

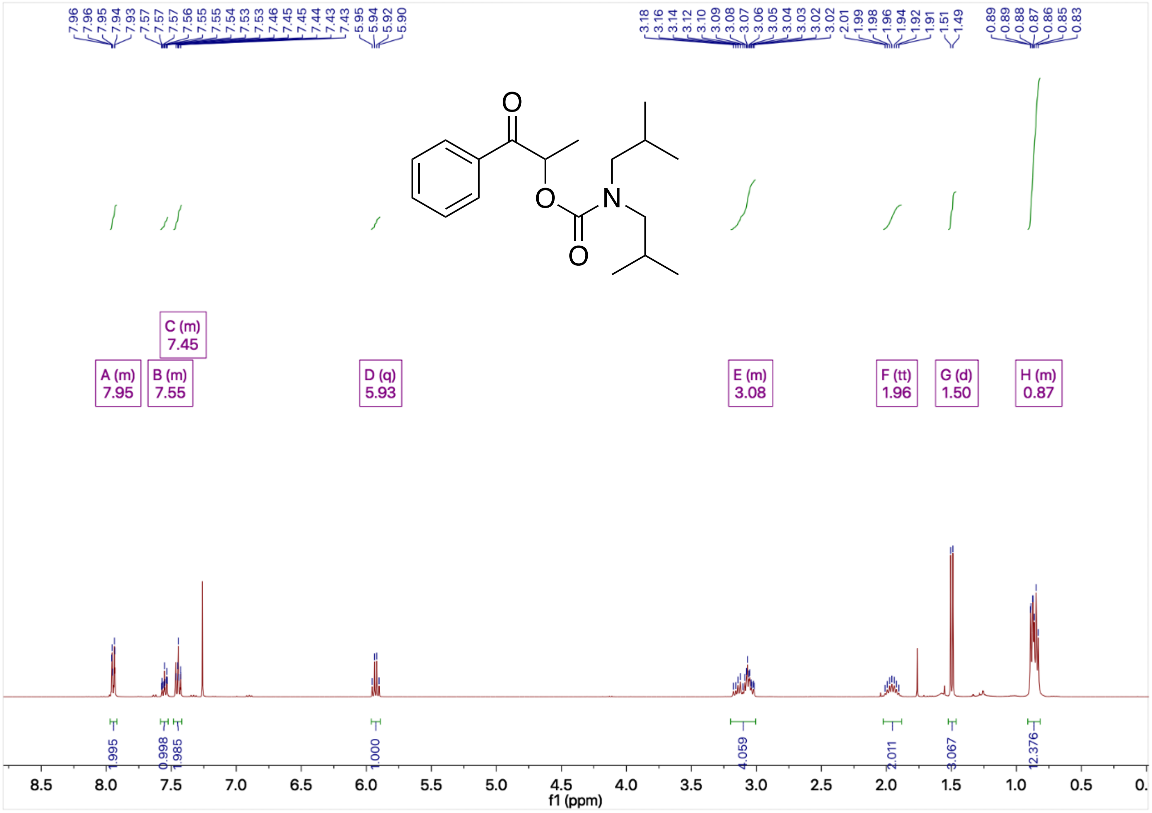

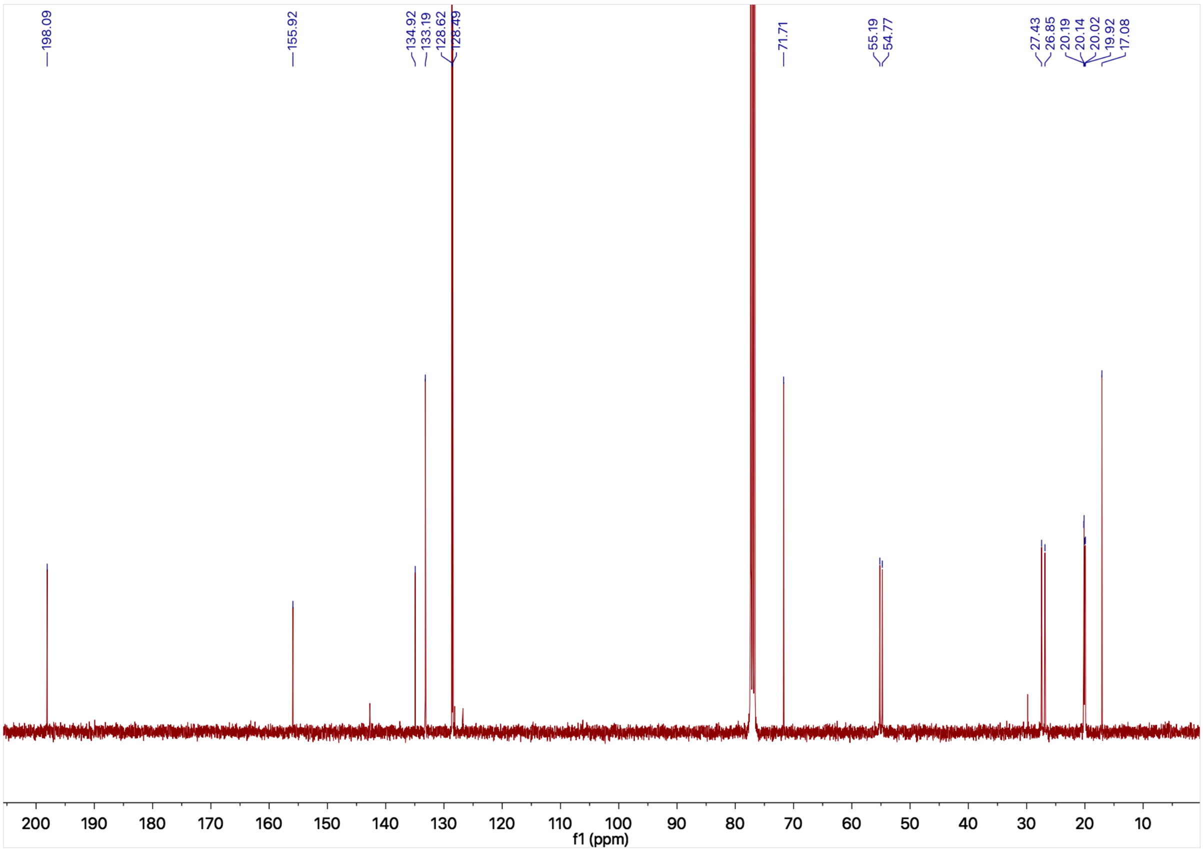

**1-oxo-1-phenylpropan-2-yl pyrrolidine-1-carboxylate (5l)**

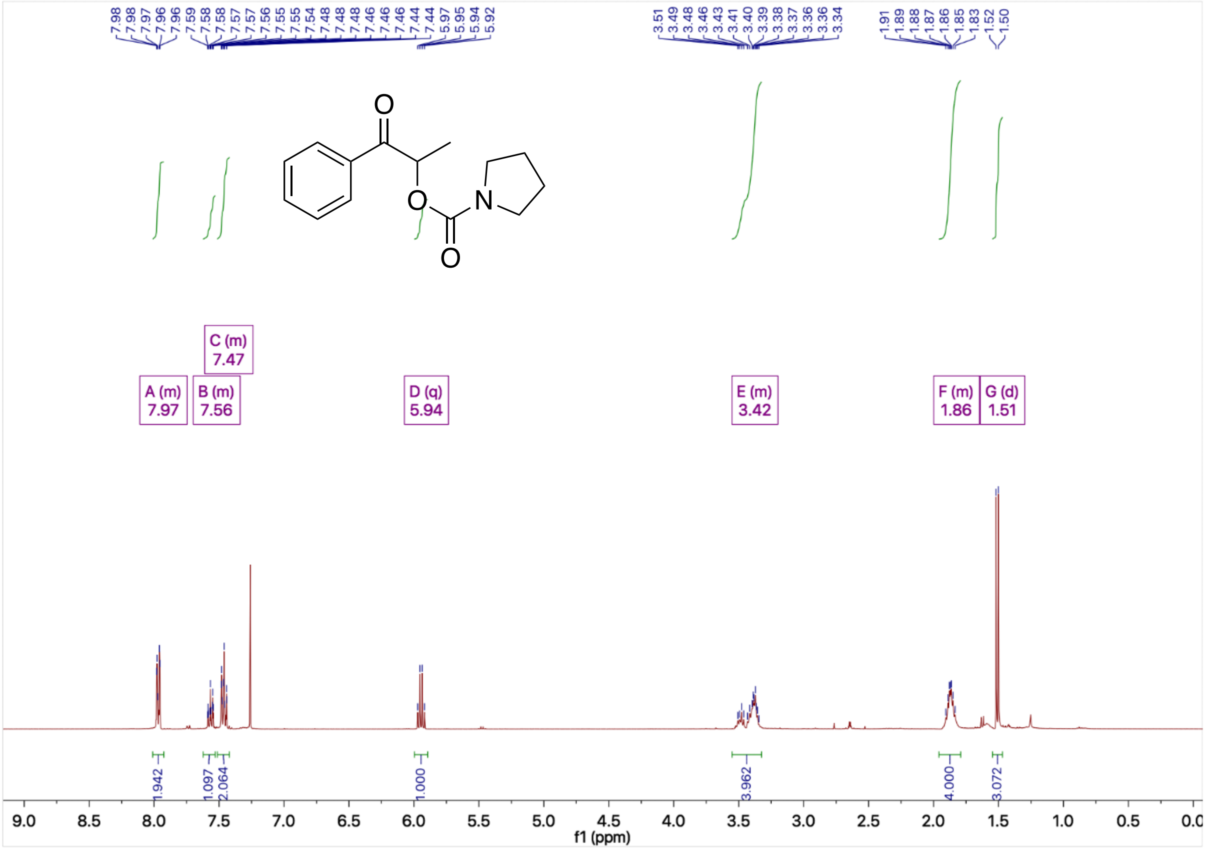


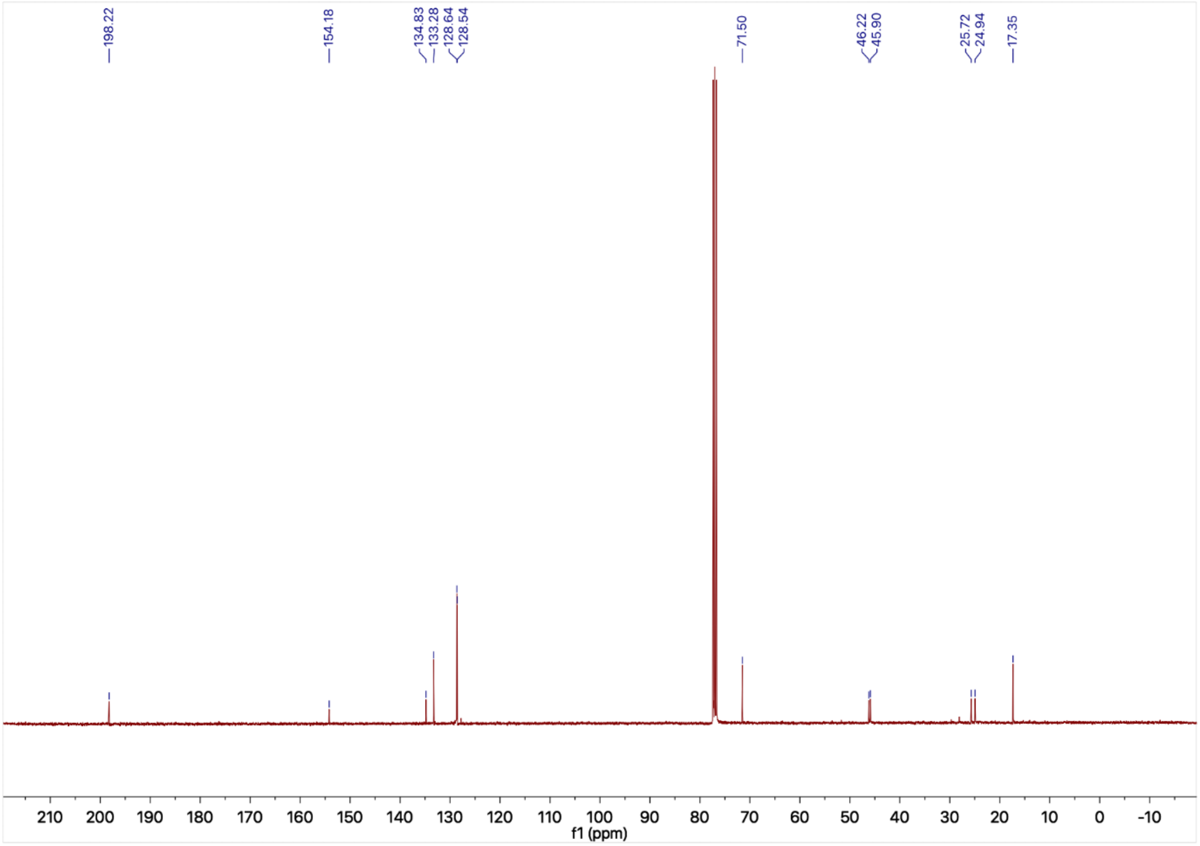

**ethyl 2-((diethylcarbamoyl)oxy)-3-oxo-3-phenylpropanoate (7a)**

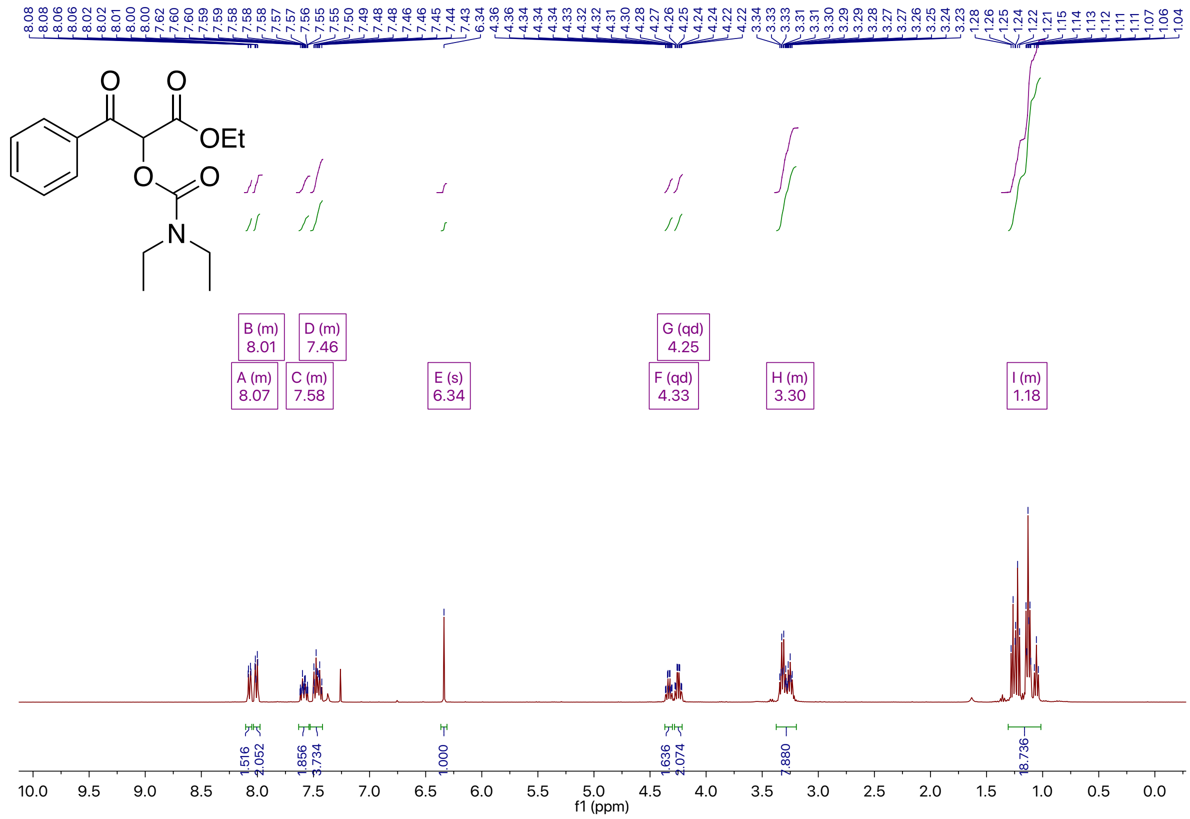

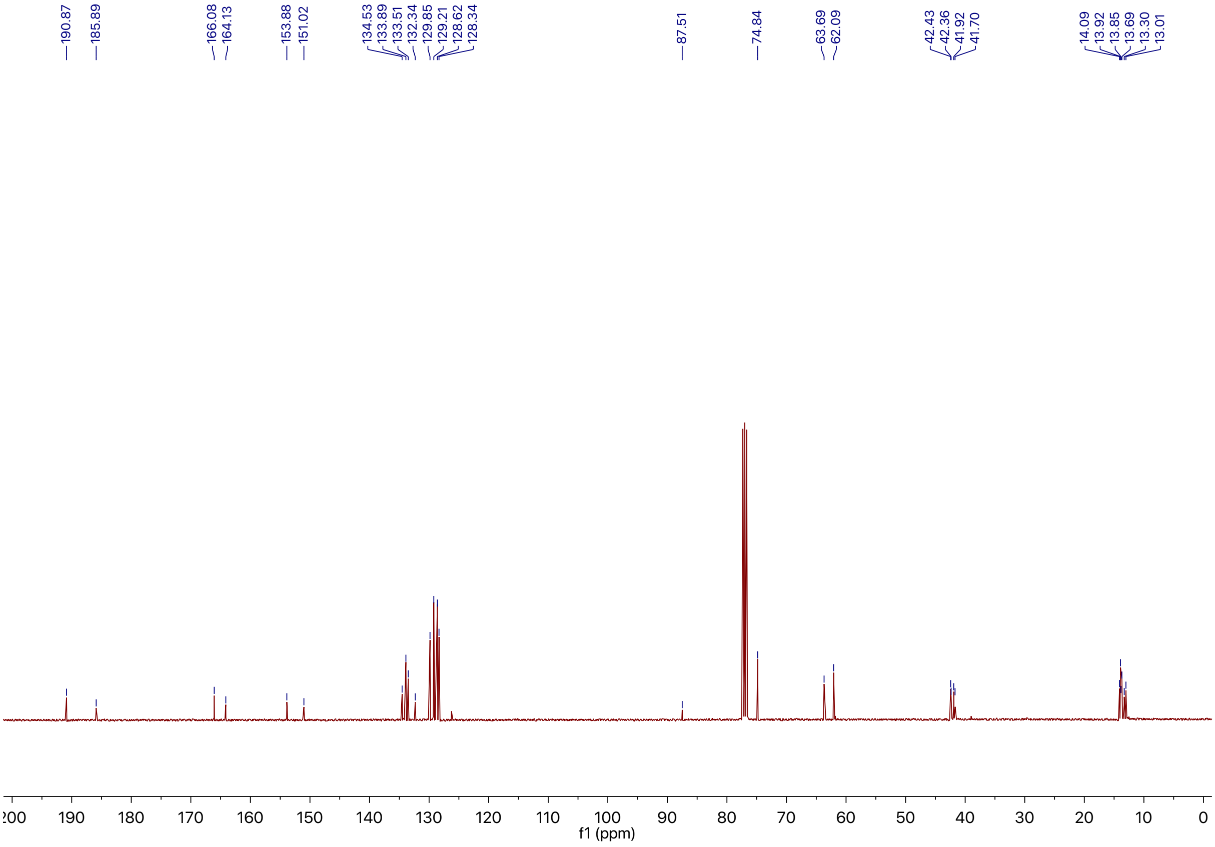

## 2,4-dioxopentan-3-yl diethylcarbamate (7b)

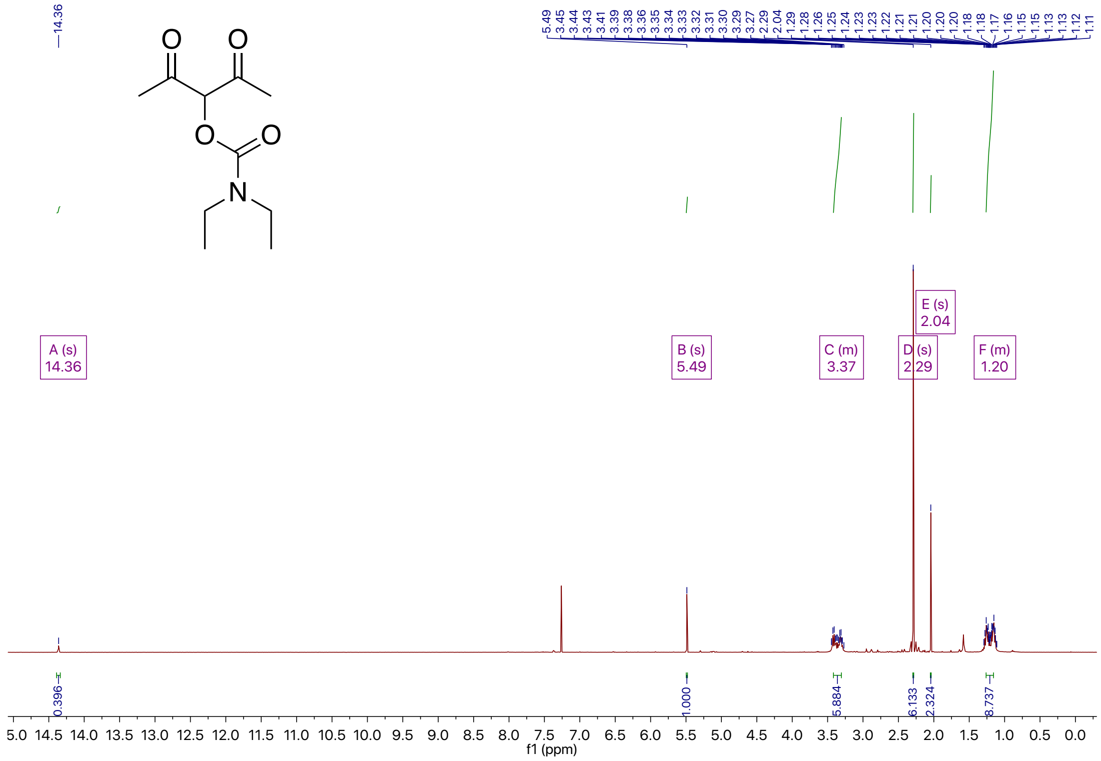


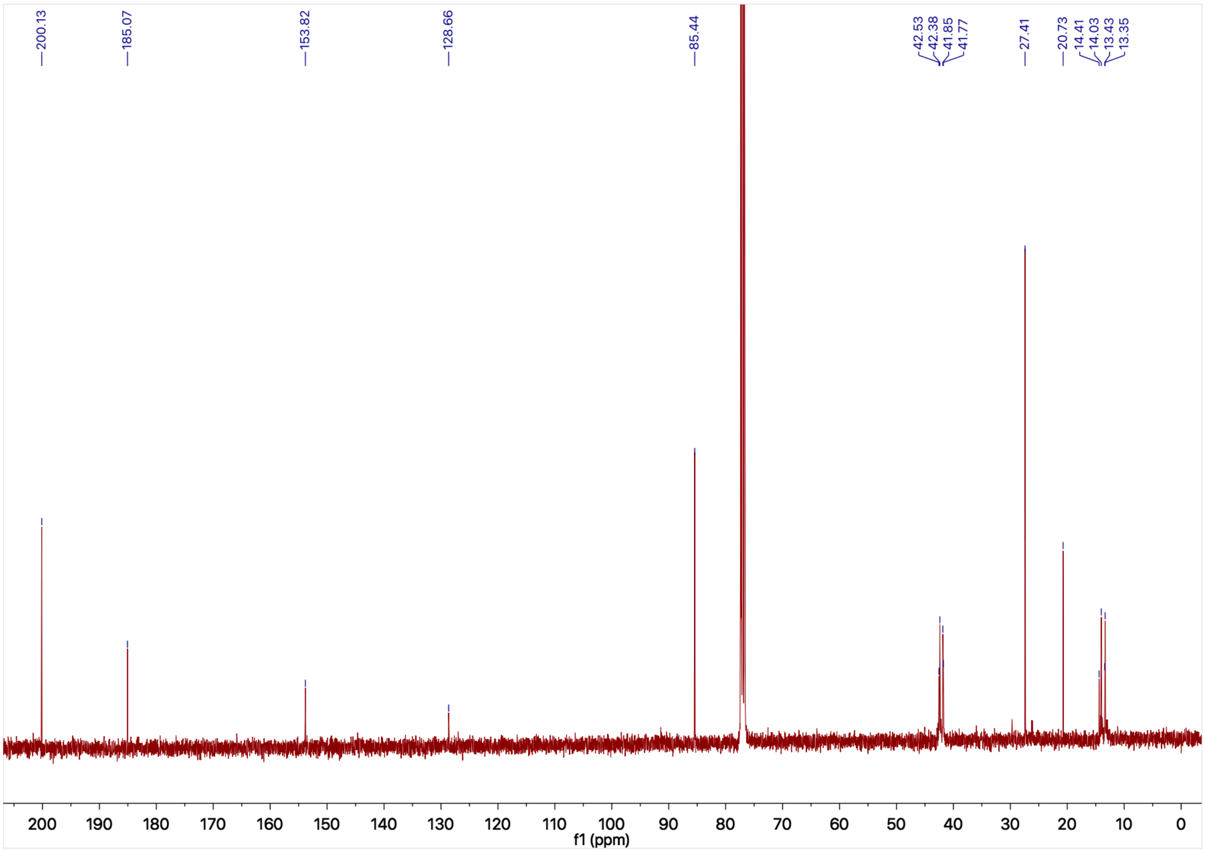

**methyl 3-(diethylamino)-2-((diethylcarbamoyl)oxy)-3-oxopropanoate (7c)**

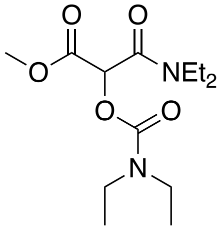


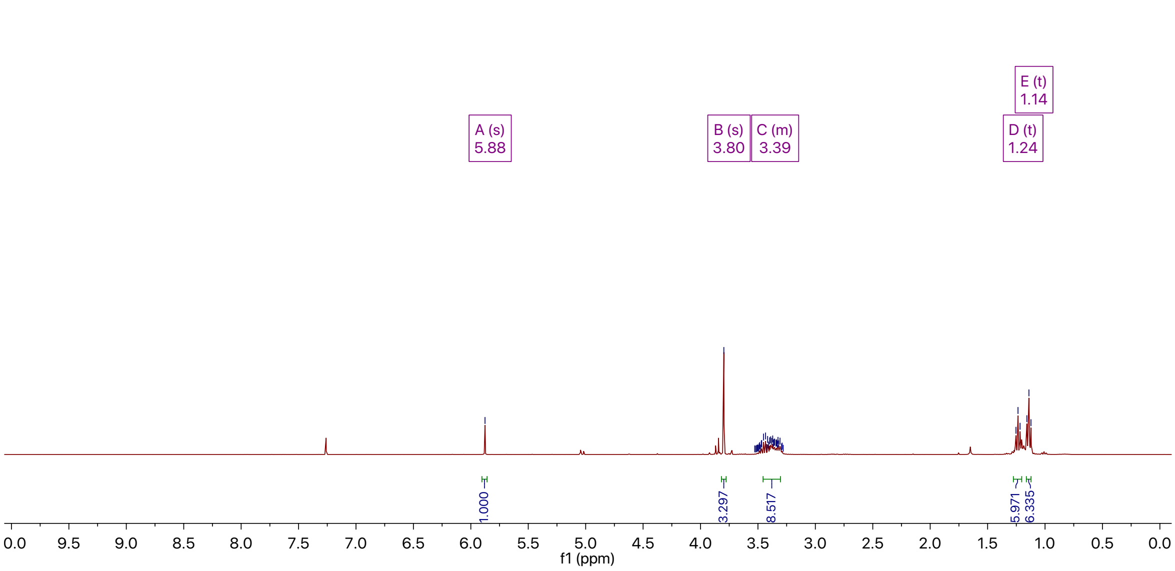


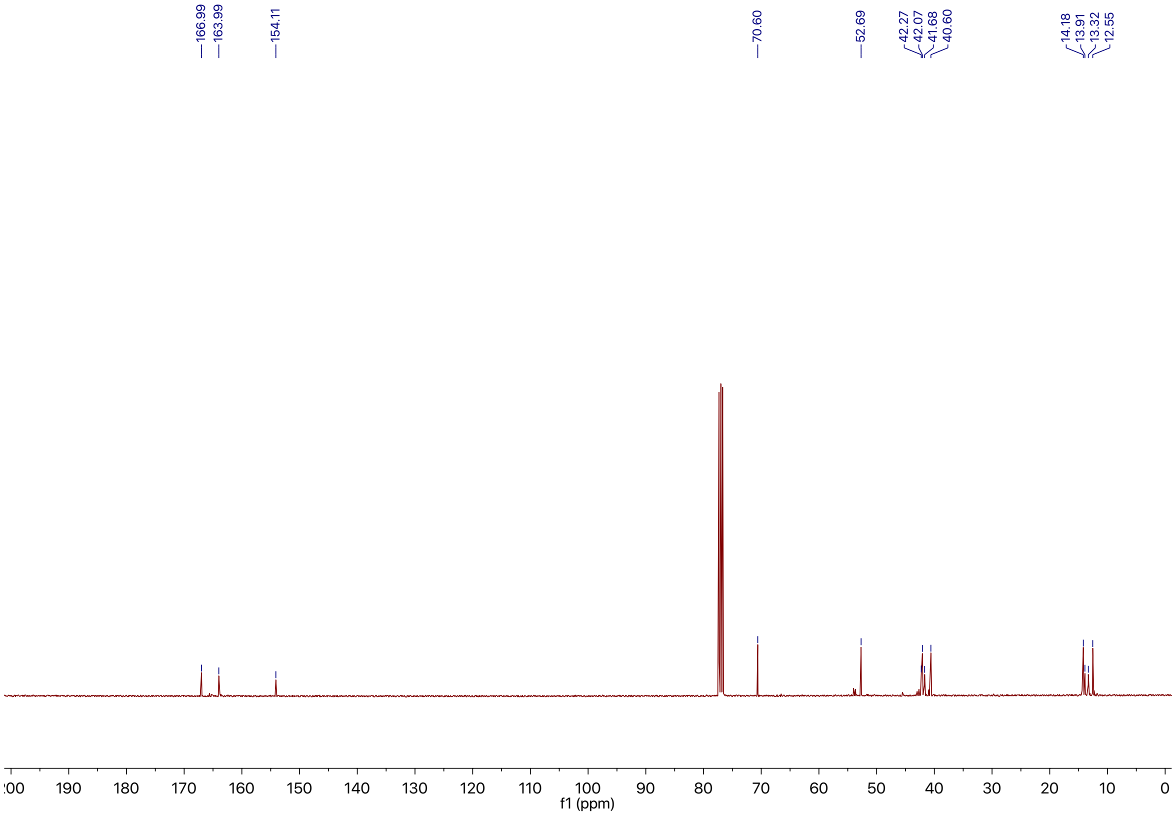

**dimethyl 2-((diethylcarbamoyl)oxy)malonate (7d)**

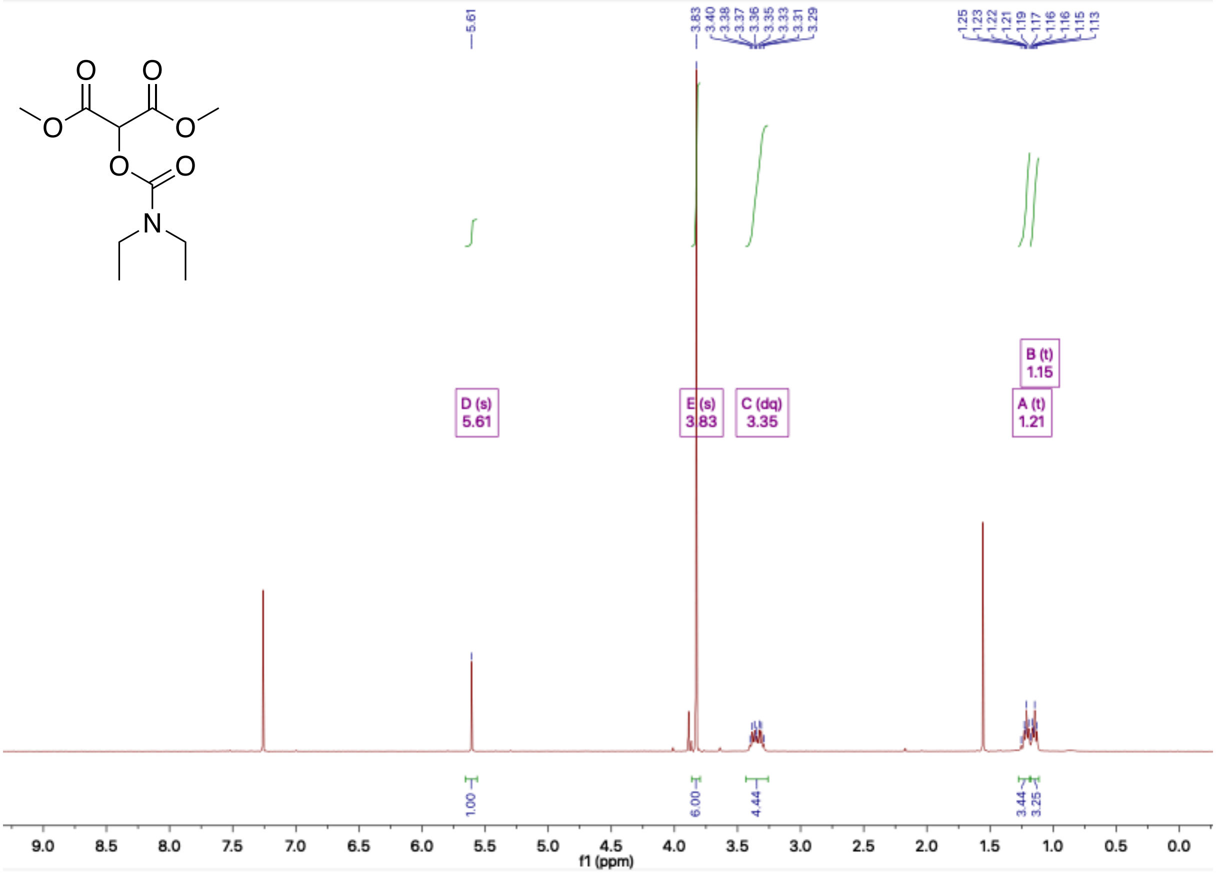


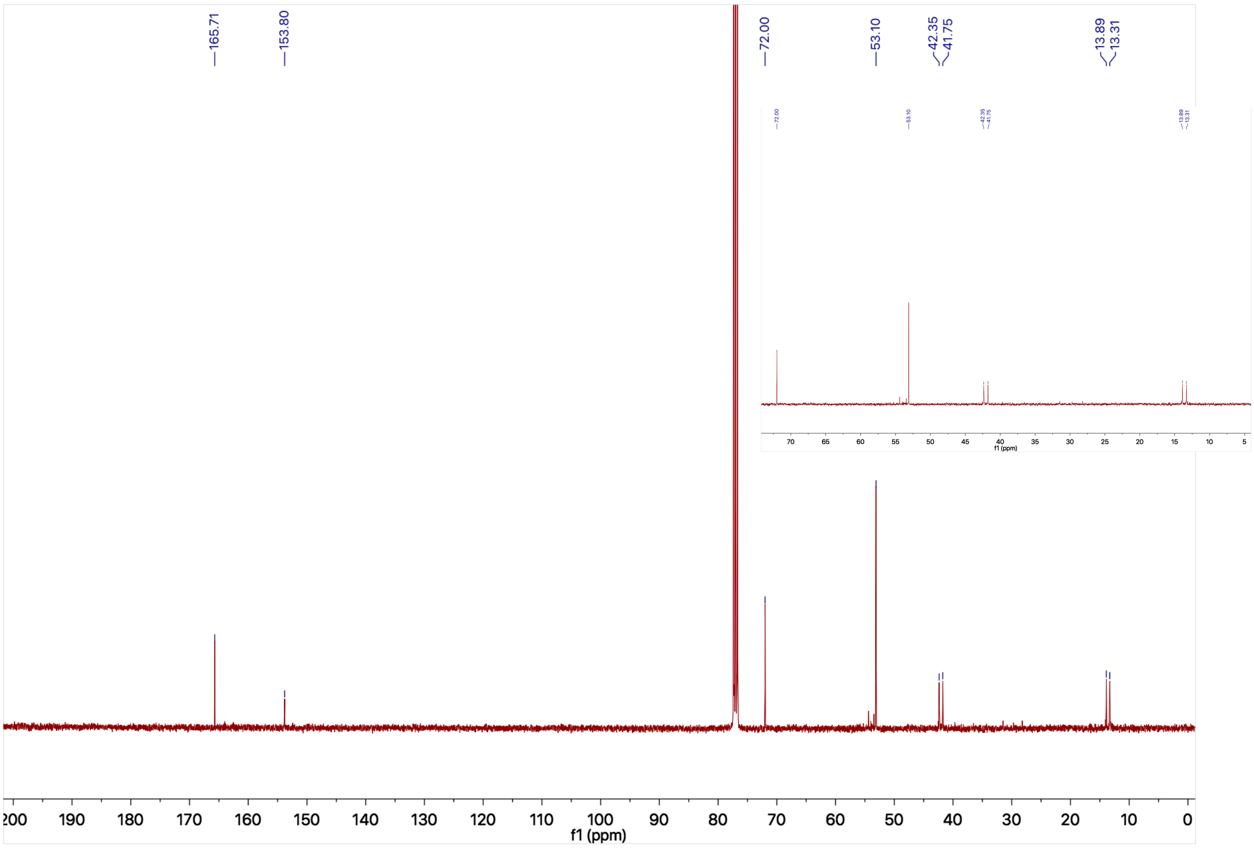

**1-oxo-1-phenylpropan-2-yl-benzoate (8a)**

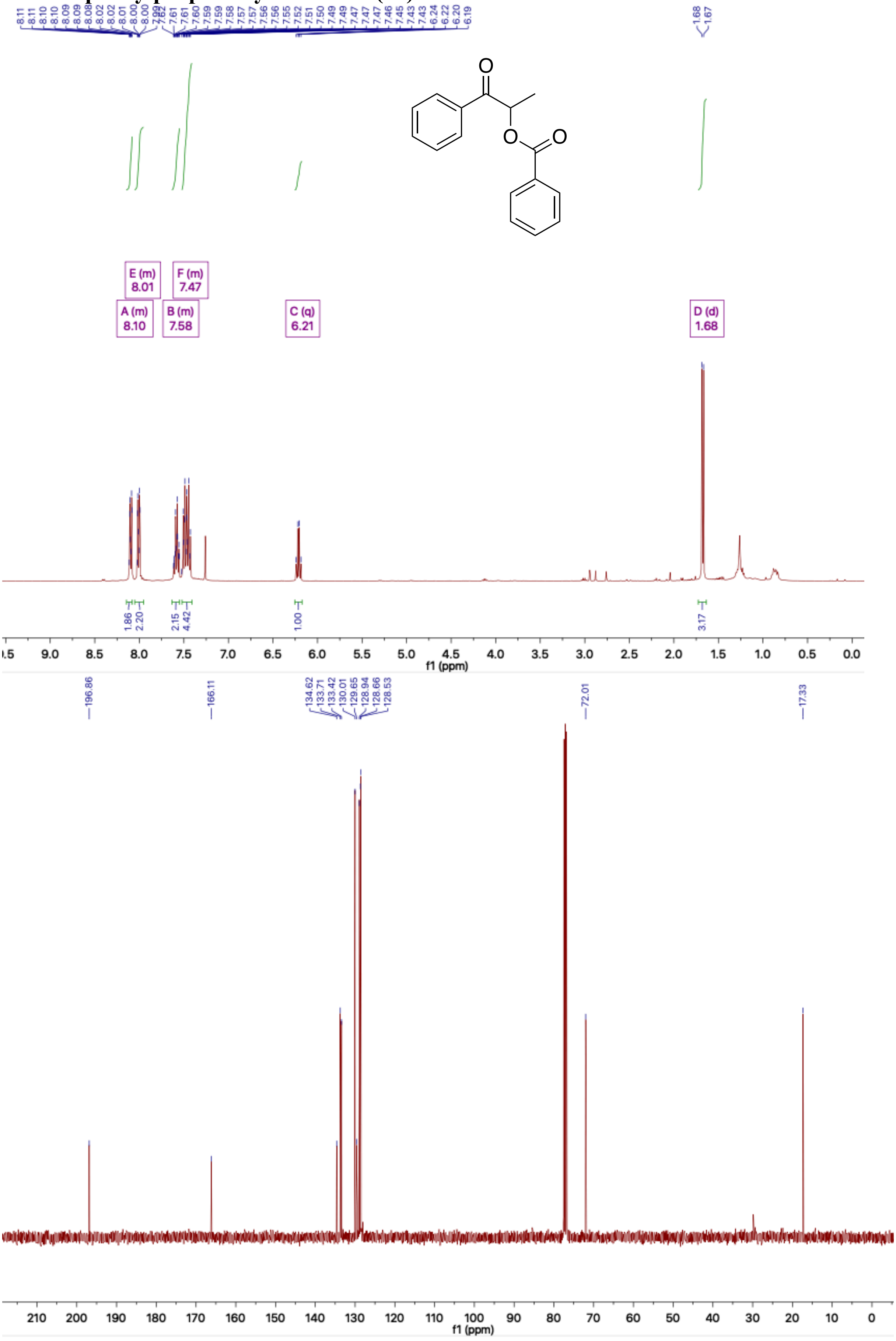

**1-oxo-1-phenylpropan-2-yl 4-methoxybenzoate (8b)**

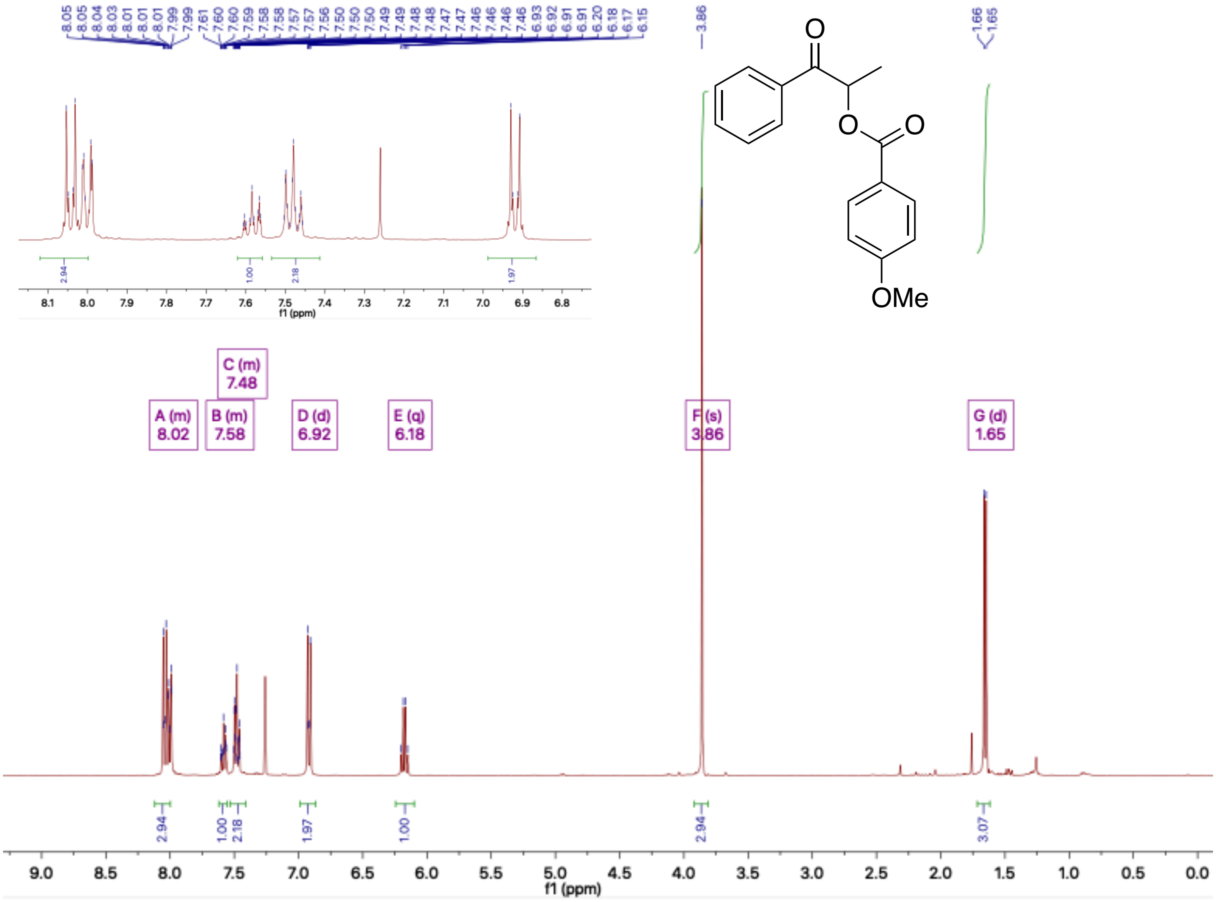

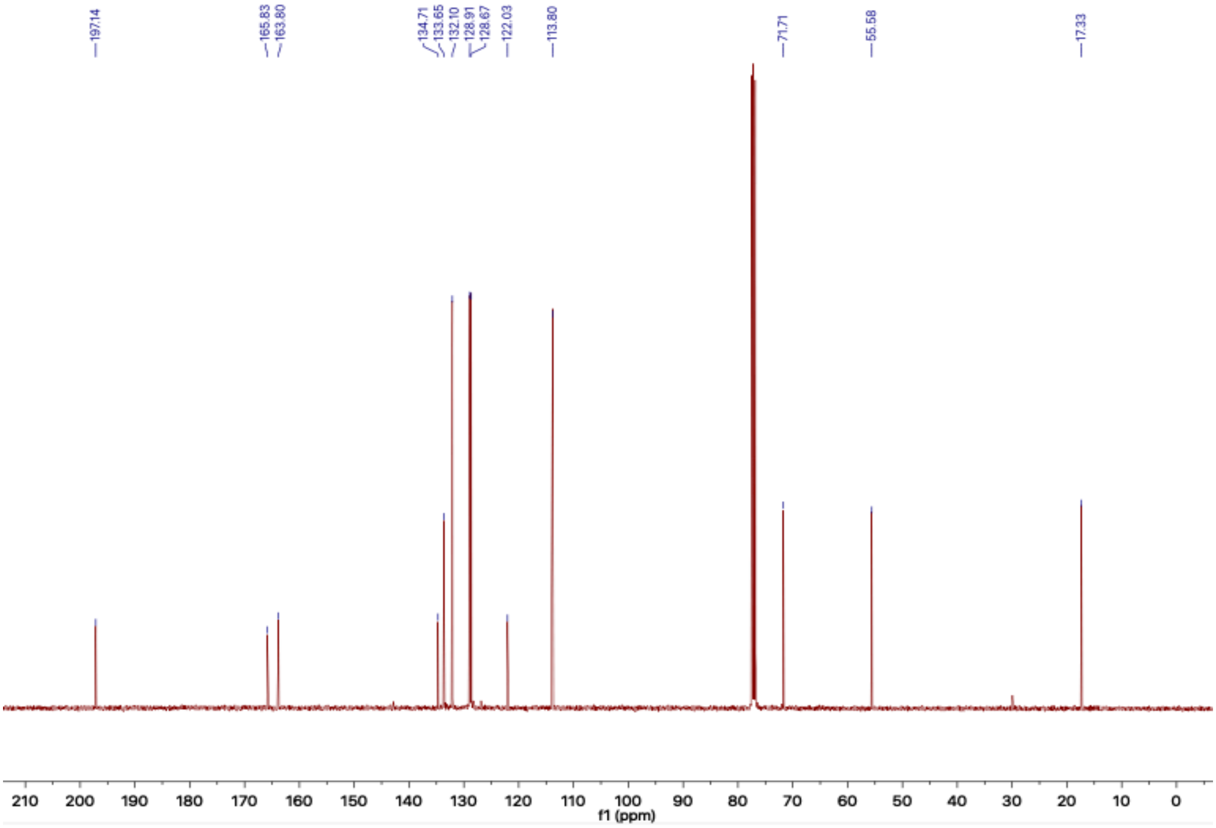

**1-oxo-1-phenylpropan-2-yl 4-(trifluoromethyl)benzoate (8c)**

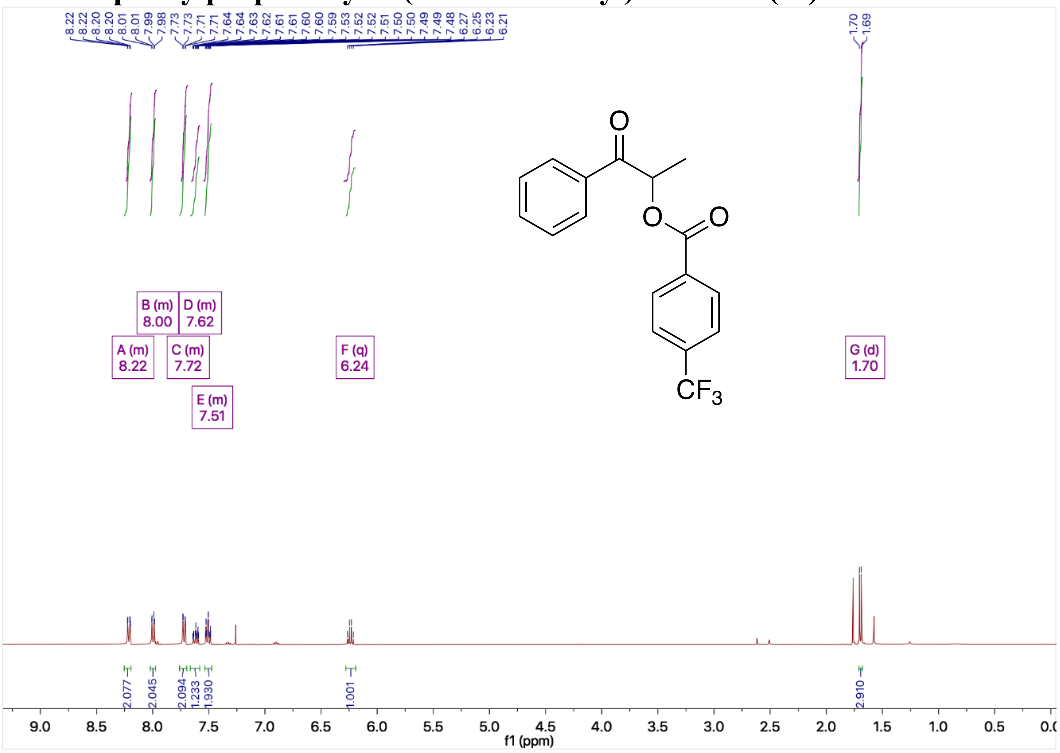

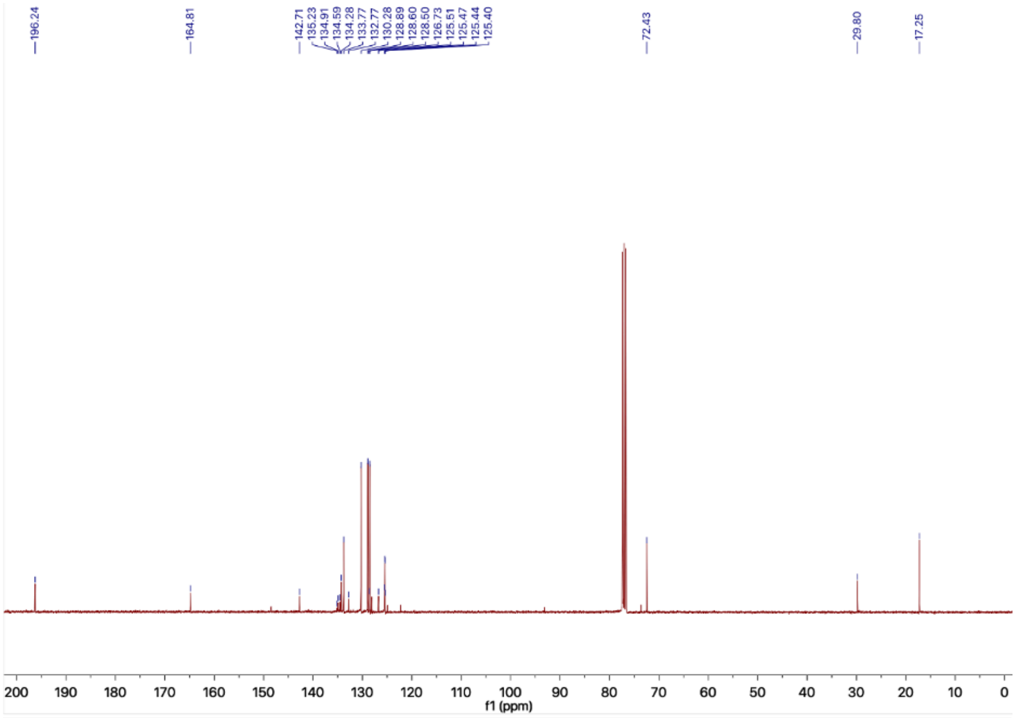
196.24
164.81
142.71
135.23
134.91
134.59
134.28
133.77
132.77
130.28
128.89
128.60
128.50
126.73
125.51
125.47
125.44
125.40
72.43
29.80
17.25
200 190 180 170 160 150 140 130 120 110 100 90 80 70 60 50 40 30 20 10 0
f1 (ppm)

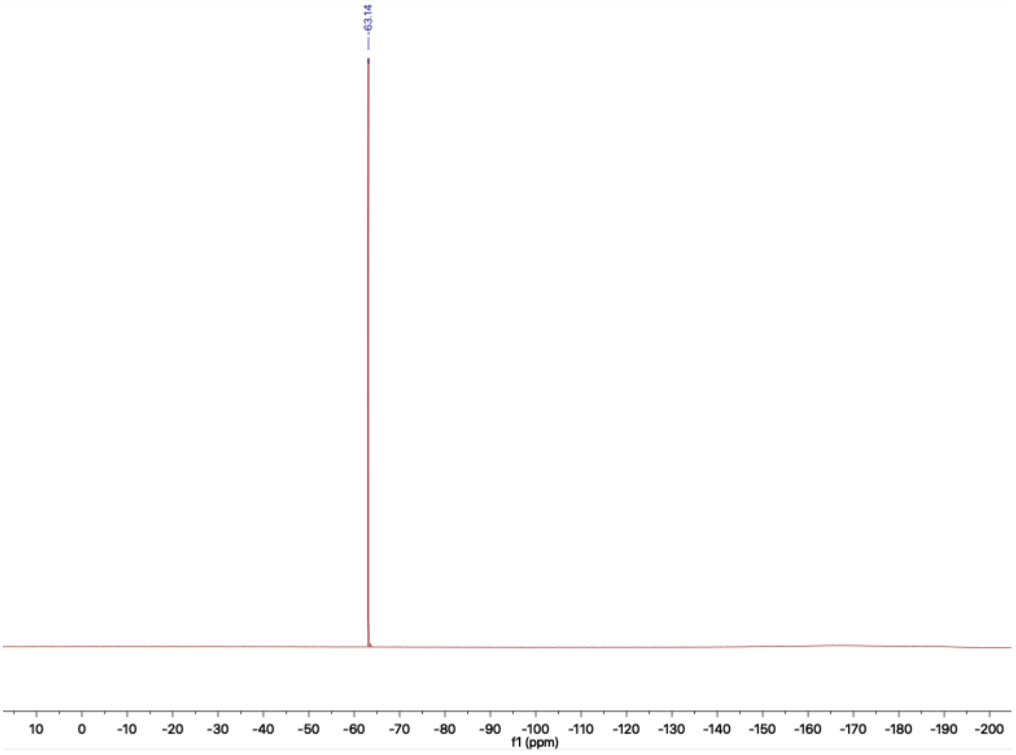
-63.14
10 0 -10 -20 -30 -40 -50 -60 -70 -80 -90 -100 -110 -120 -130 -140 -150 -160 -170 -180 -190 -200
f1 (ppm)

**2-phenoxy-1-phenyl-propan-1-one (8d)**

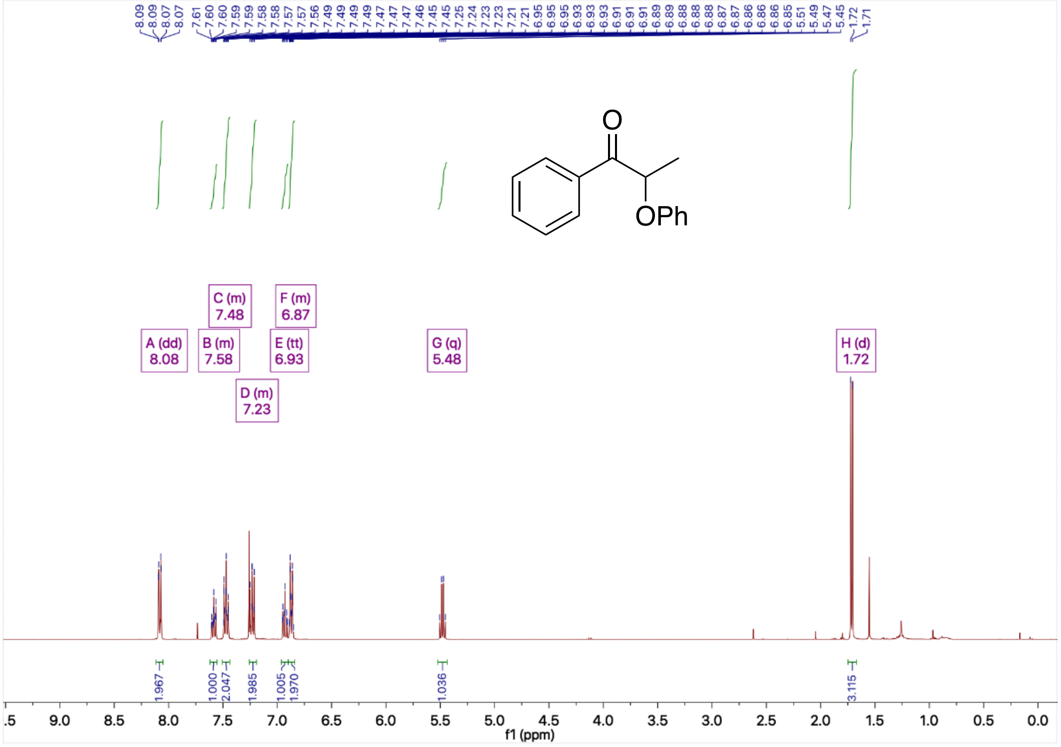


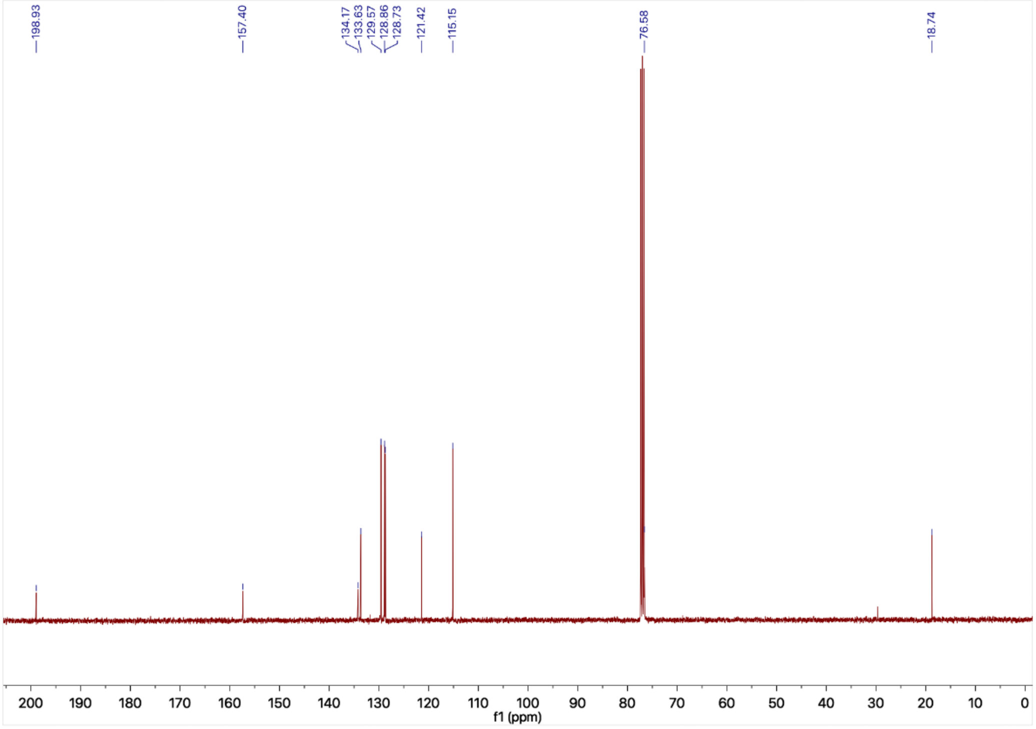

**1-phenyl-2-(phenylthio)propan-1-one (8e)**

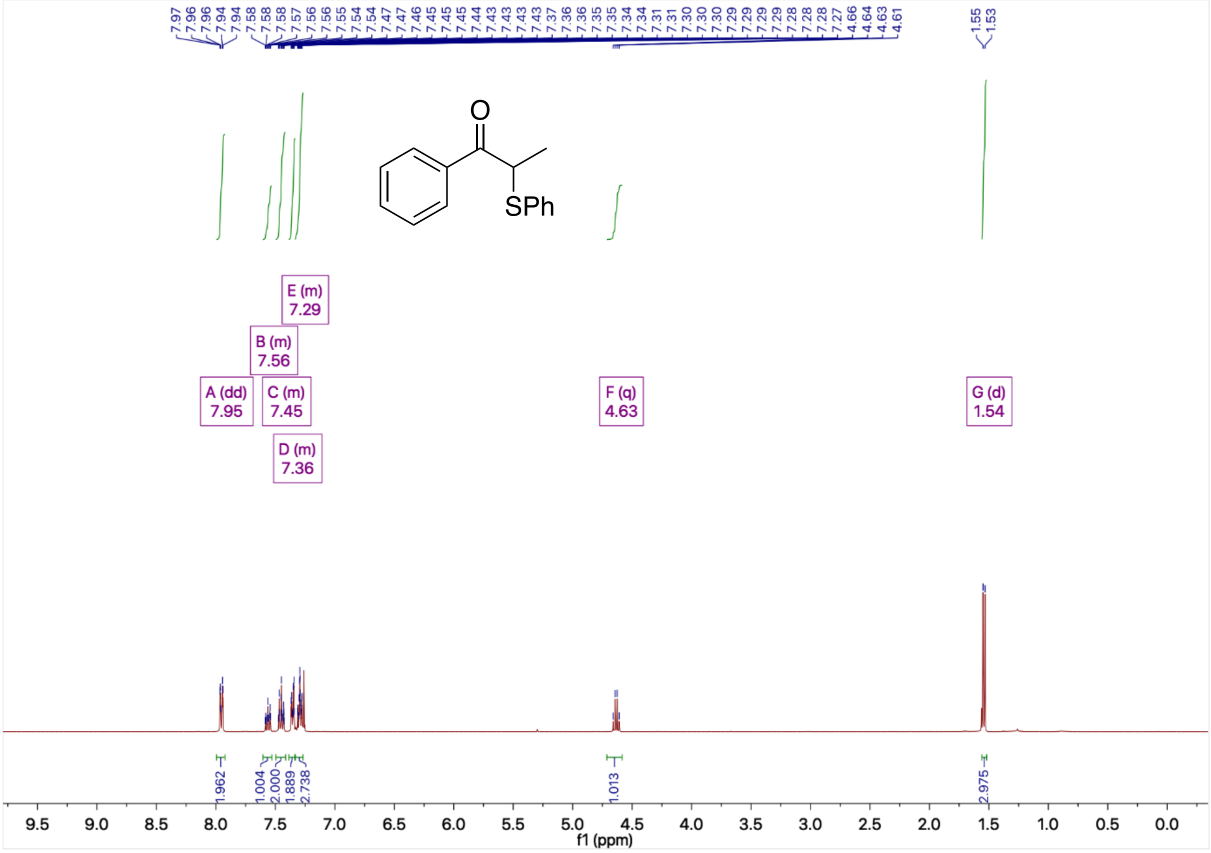


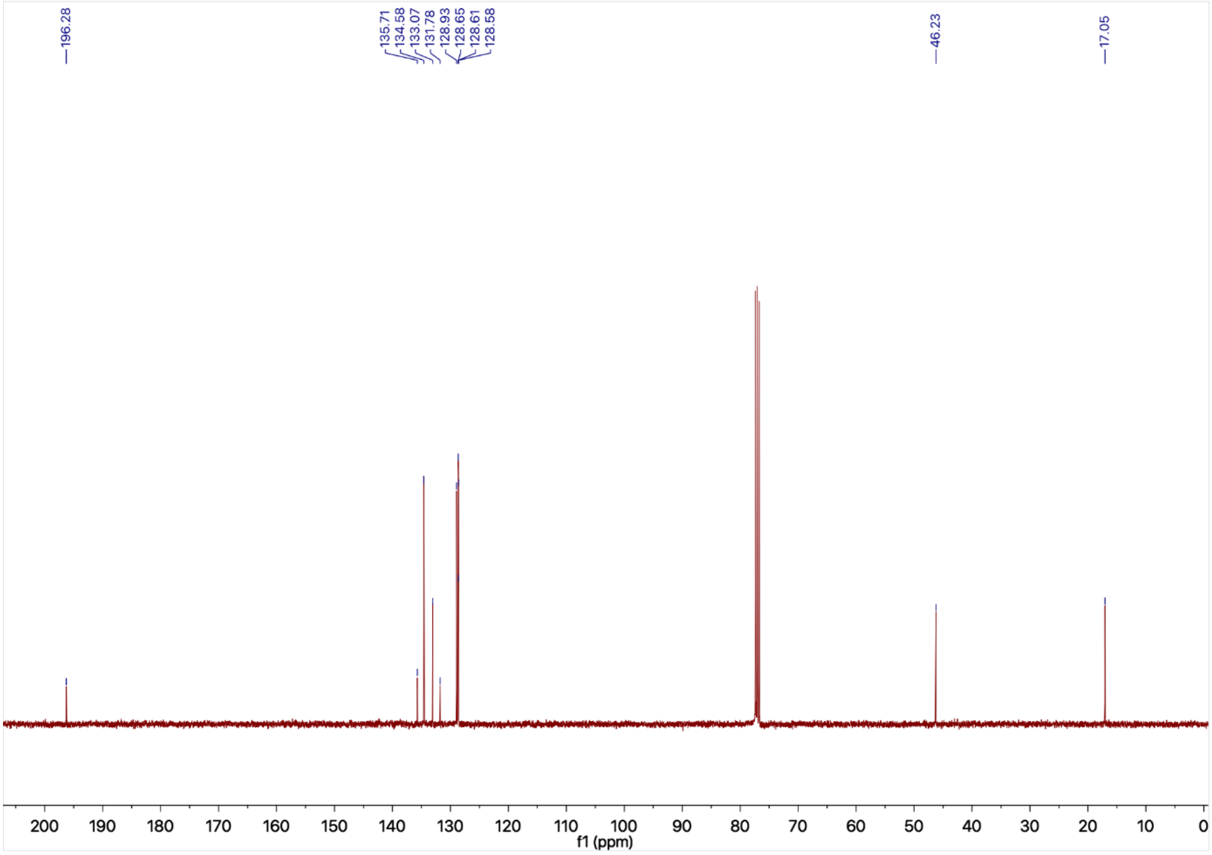

**1-phenyl-2-(phenylthio)propan-1-one (8f)**

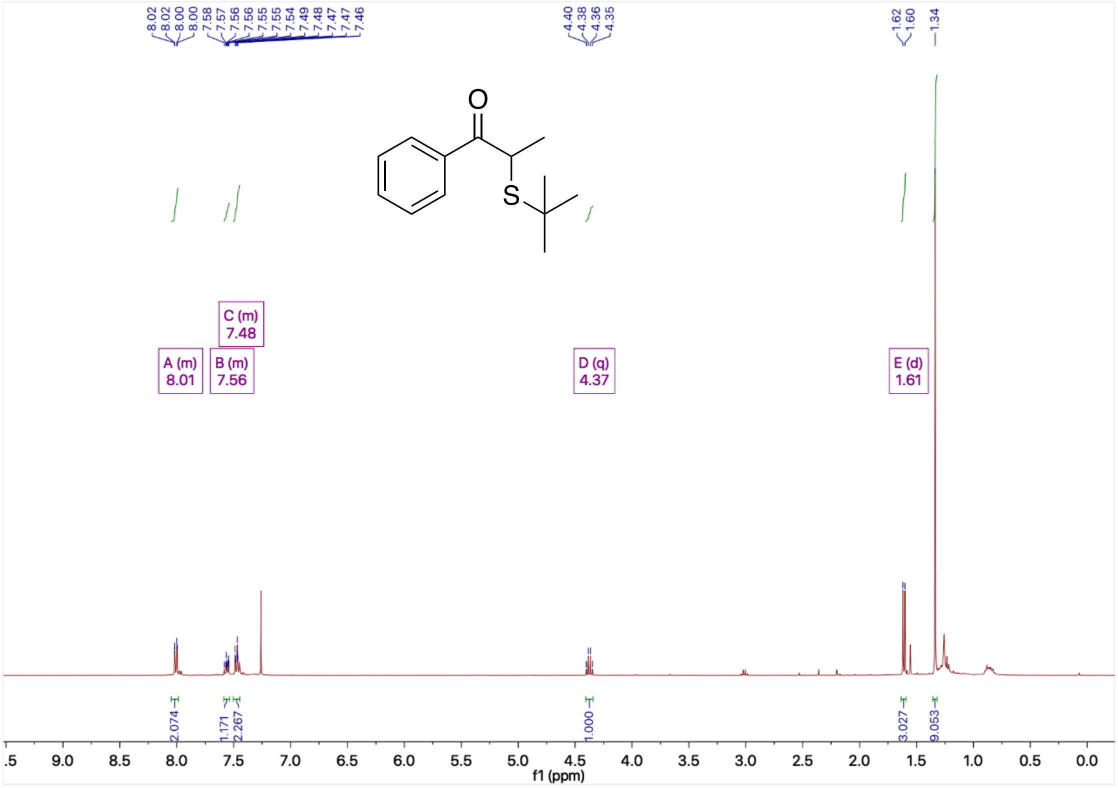

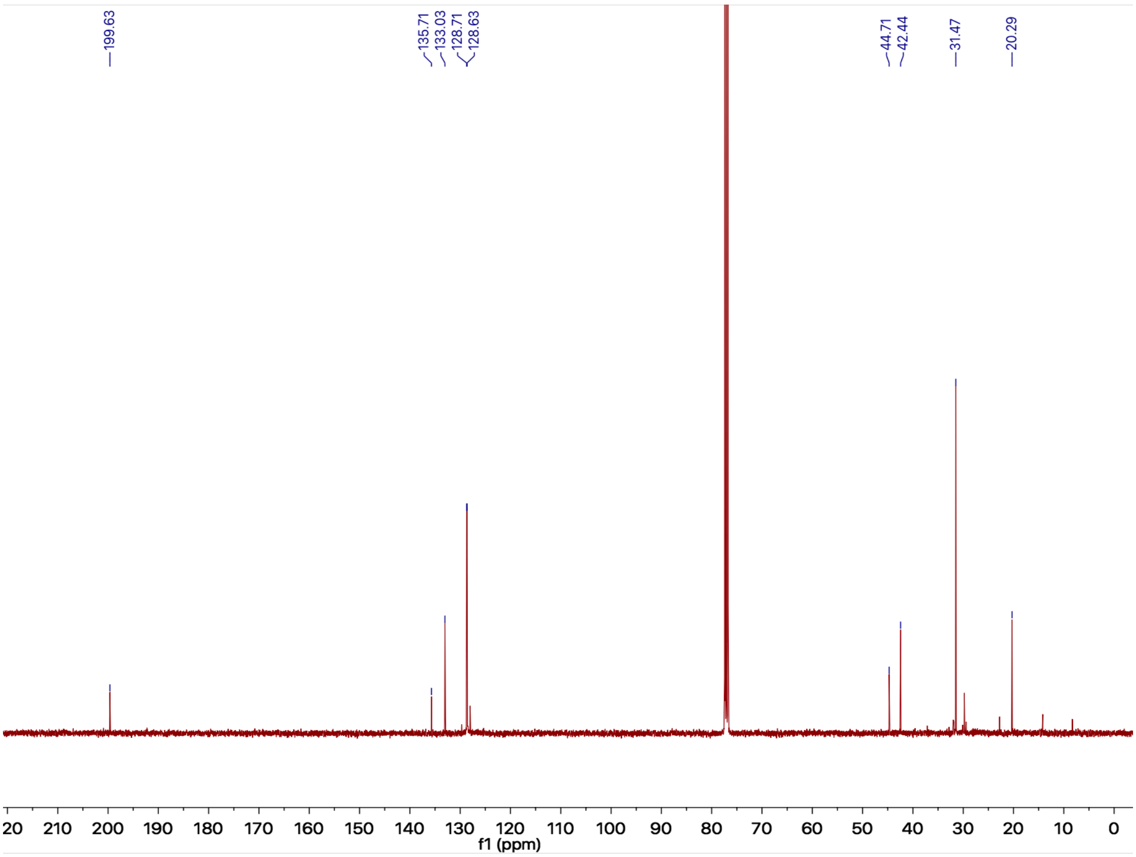

## 2-(benzylamino)-1-phenylpropan-1-one (8g)

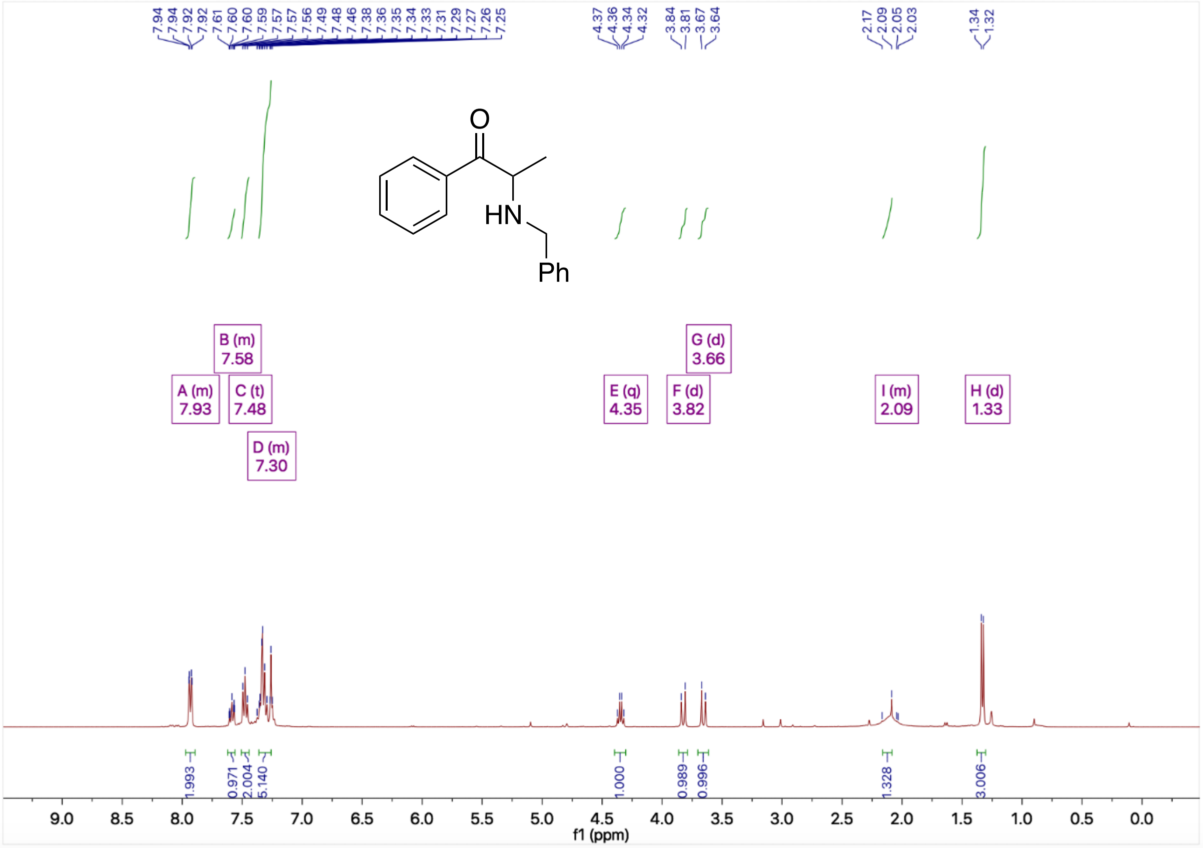

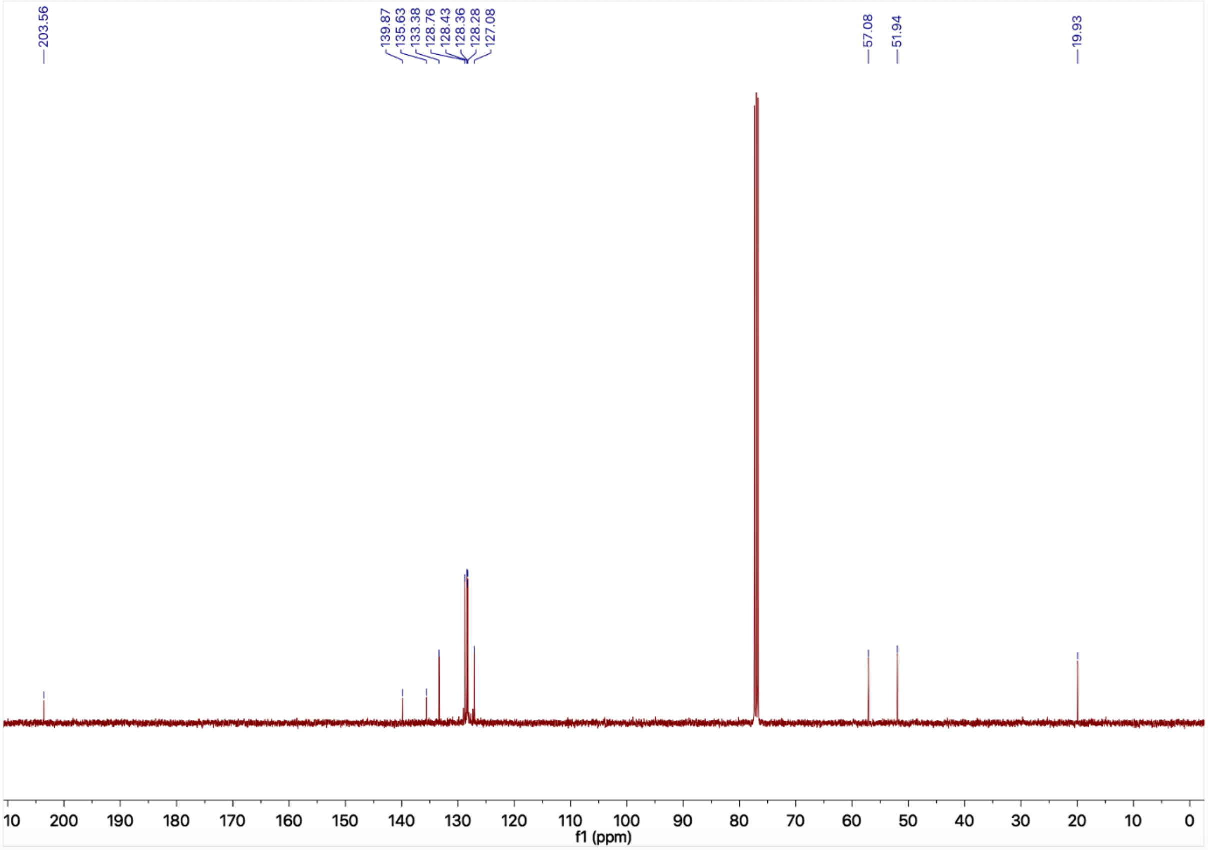

**2-(cyclopropylamino)-1-phenylpropan-1-one (8h)**

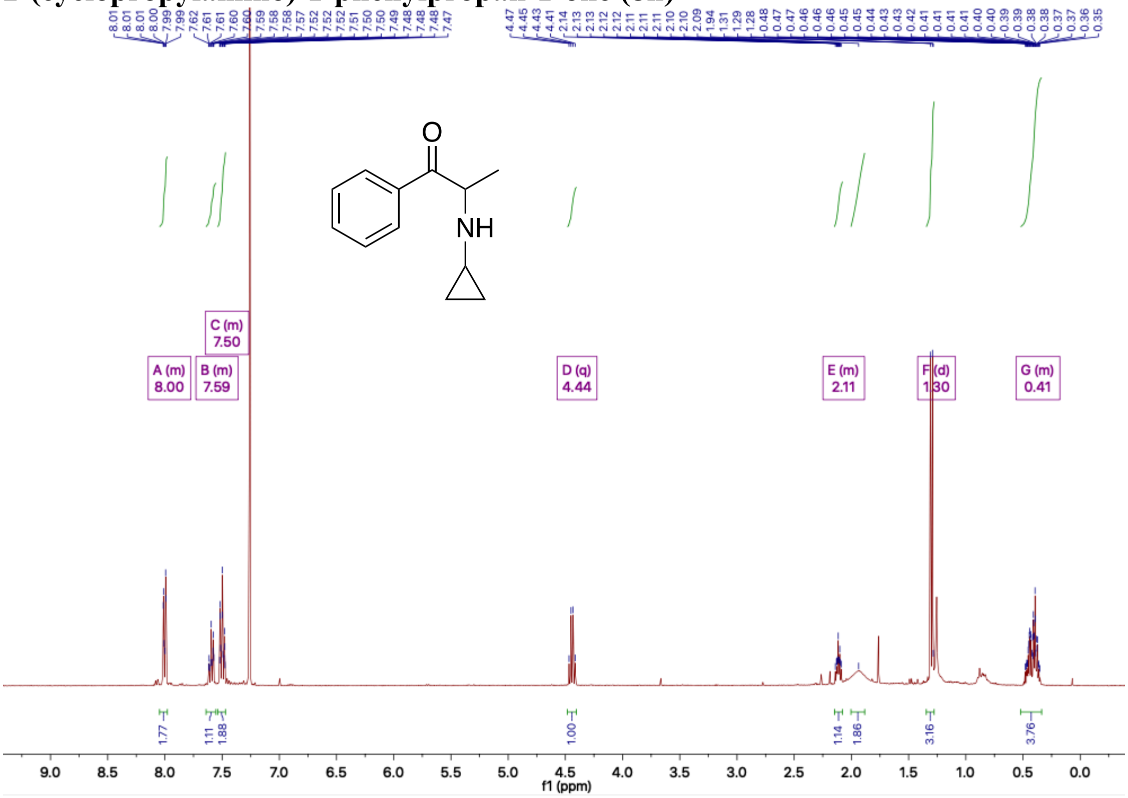

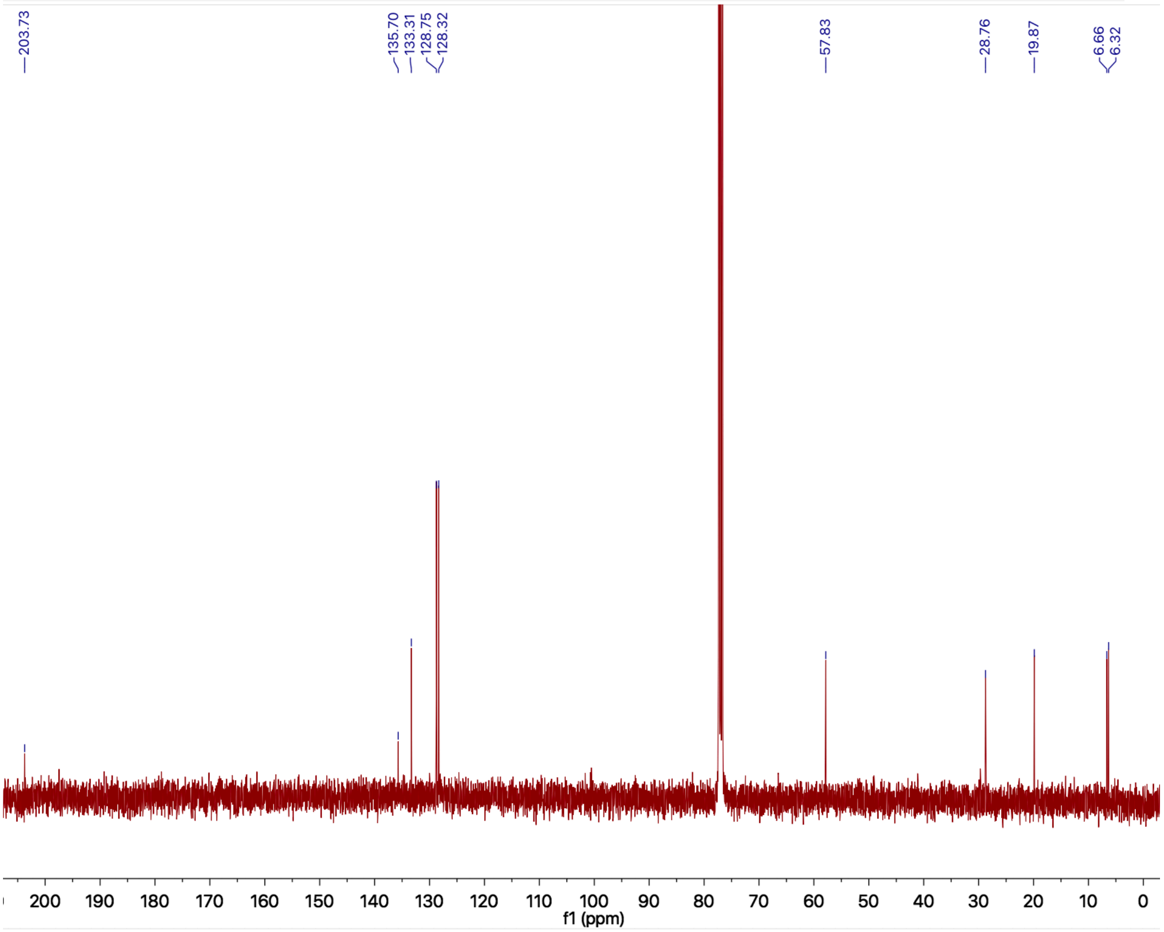

**1-phenyl-2-(piperidin-1-yl)propan-1-one (8i)**

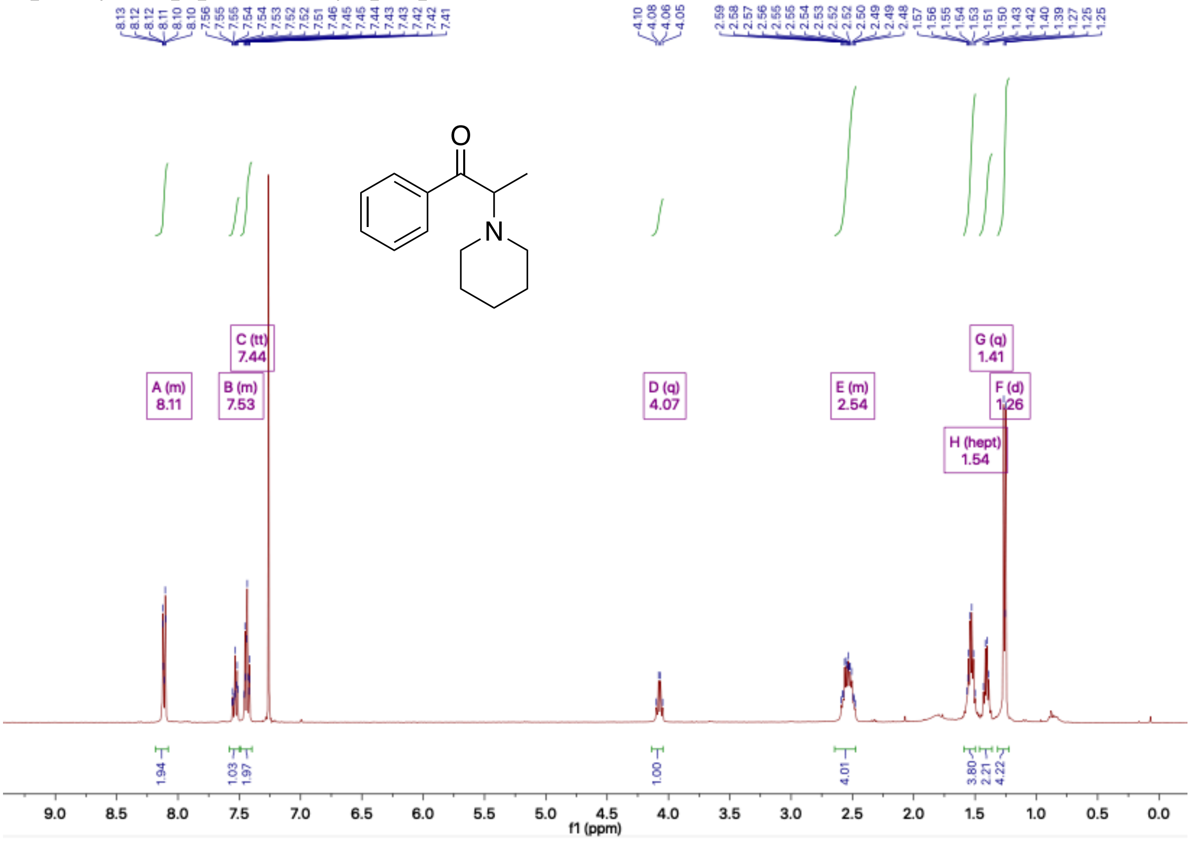


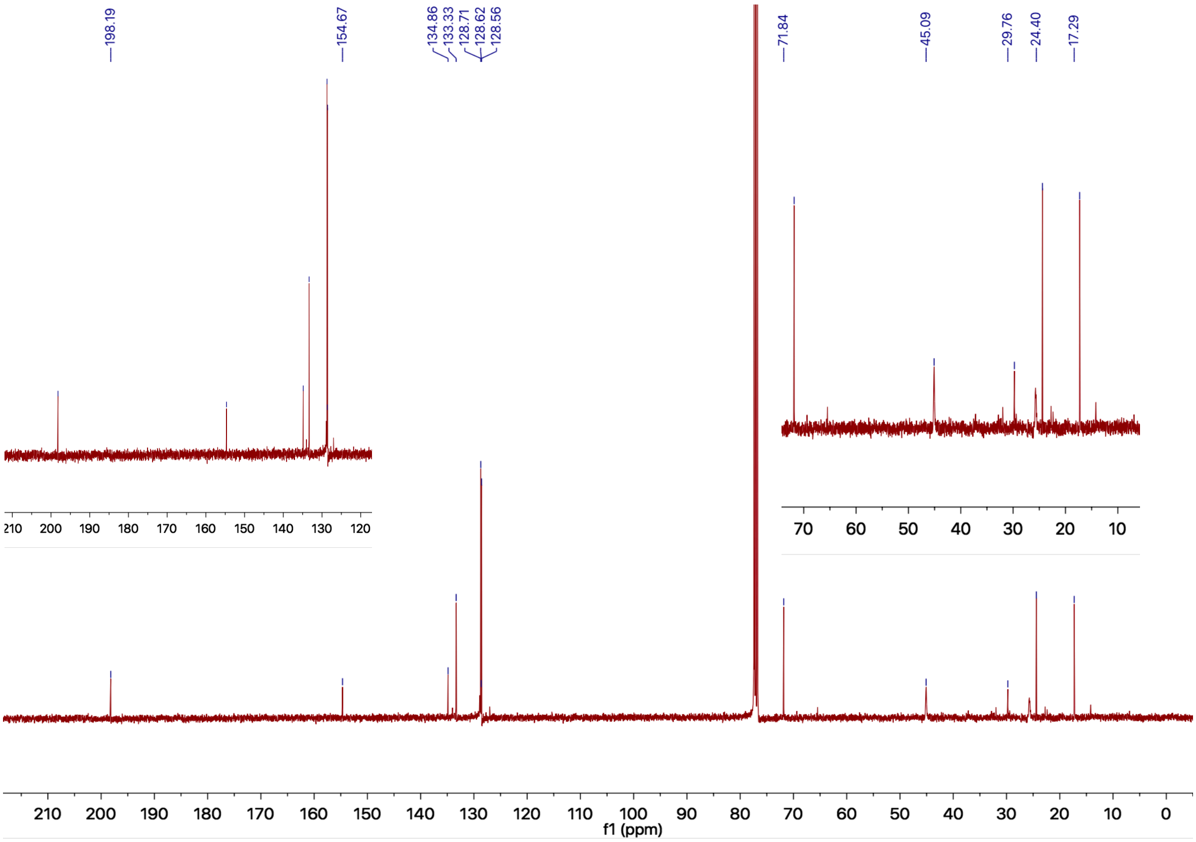


**2-morpholino-1-phenylpropan-1-one (8j)**

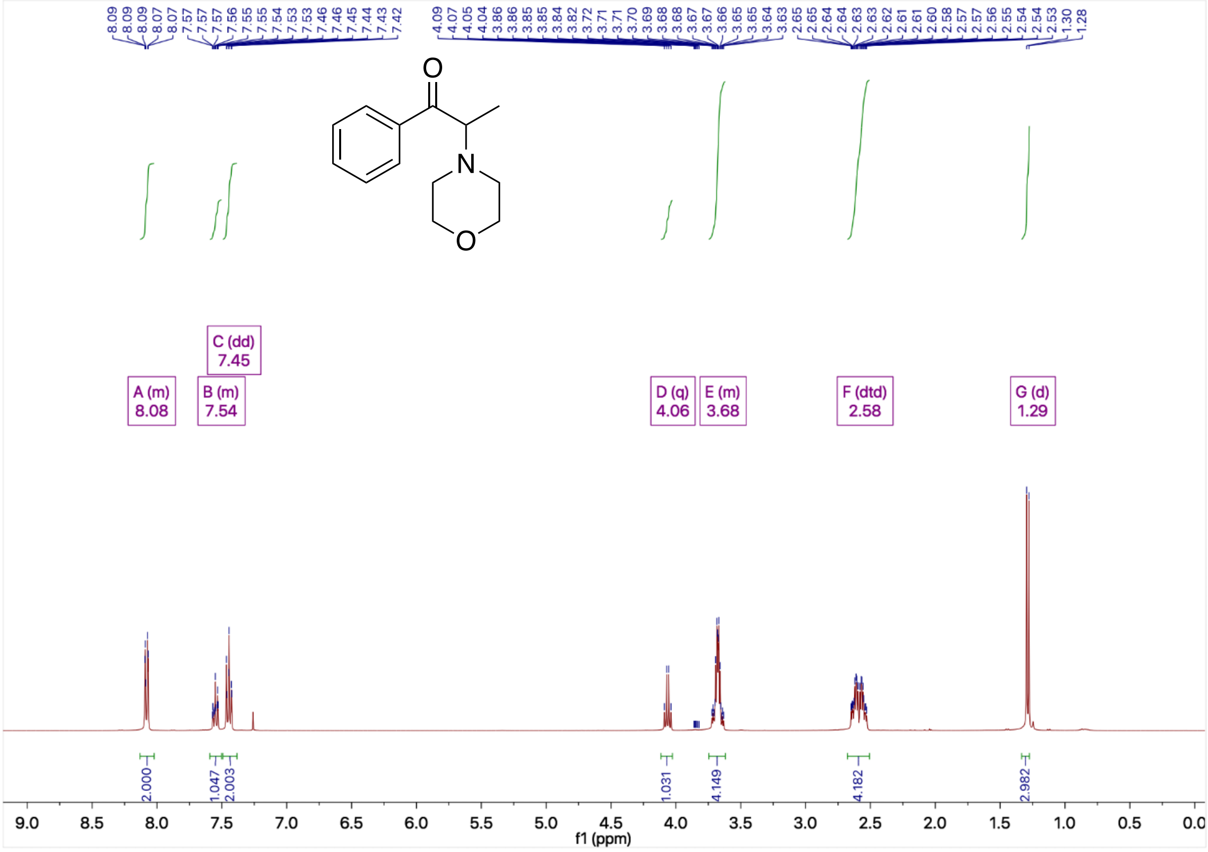

8.09
8.09
8.09
8.07
8.07
7.57
7.57
7.57
7.56
7.55
7.55
7.54
7.53
7.53
7.46
7.46
7.45
7.44
7.43
7.42
4.09
4.07
4.05
4.04
3.86
3.86
3.85
3.85
3.84
3.82
3.72
3.71
3.71
3.70
3.69
3.68
3.68
3.67
3.67
3.66
3.65
3.65
3.64
3.63
2.65
2.65
2.64
2.64
2.63
2.63
2.62
2.61
2.61
2.60
2.58
2.57
2.57
2.56
2.55
2.54
2.54
2.53
1.30
1.28
O
N
O
C (dd)
7.45
A (m)
8.08
B (m)
7.54
D (q)
4.06
E (m)
3.68
F (dtd)
2.58
G (d)
1.29
2.000
1.047
2.003
1.031
4.149
4.182
2.982
9.0
8.5
8.0
7.5
7.0
6.5
6.0
5.5
5.0
4.5
4.0
3.5
3.0
2.5
2.0
1.5
1.0
0.5
0.0
f1 (ppm)


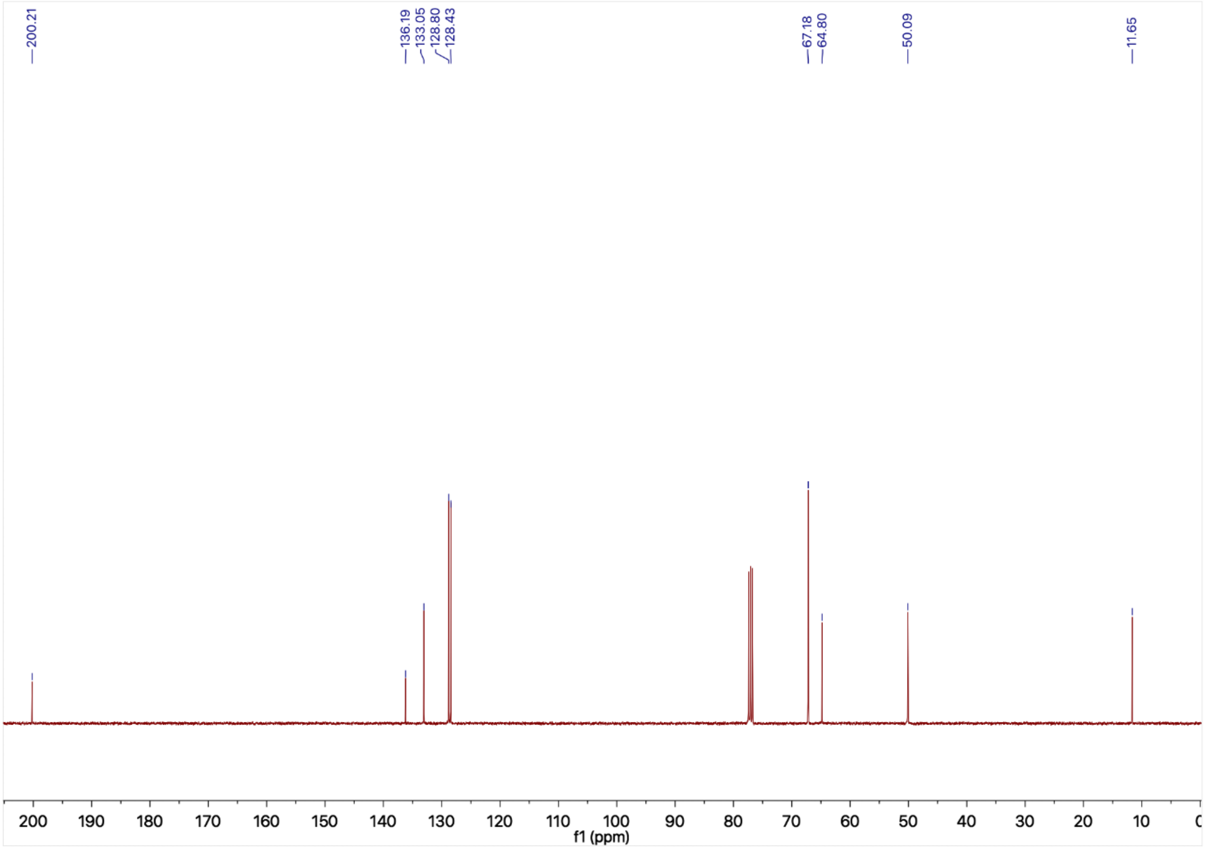

200.21
136.19
133.05
128.80
128.43
67.18
64.80
50.09
11.65
200
190
180
170
160
150
140
130
120
110
100
90
80
70
60
50
40
30
20
10
0
f1 (ppm)

**dimethyl 2-(1-oxo-1-phenylpropan-2-yl)malonate (8k)**

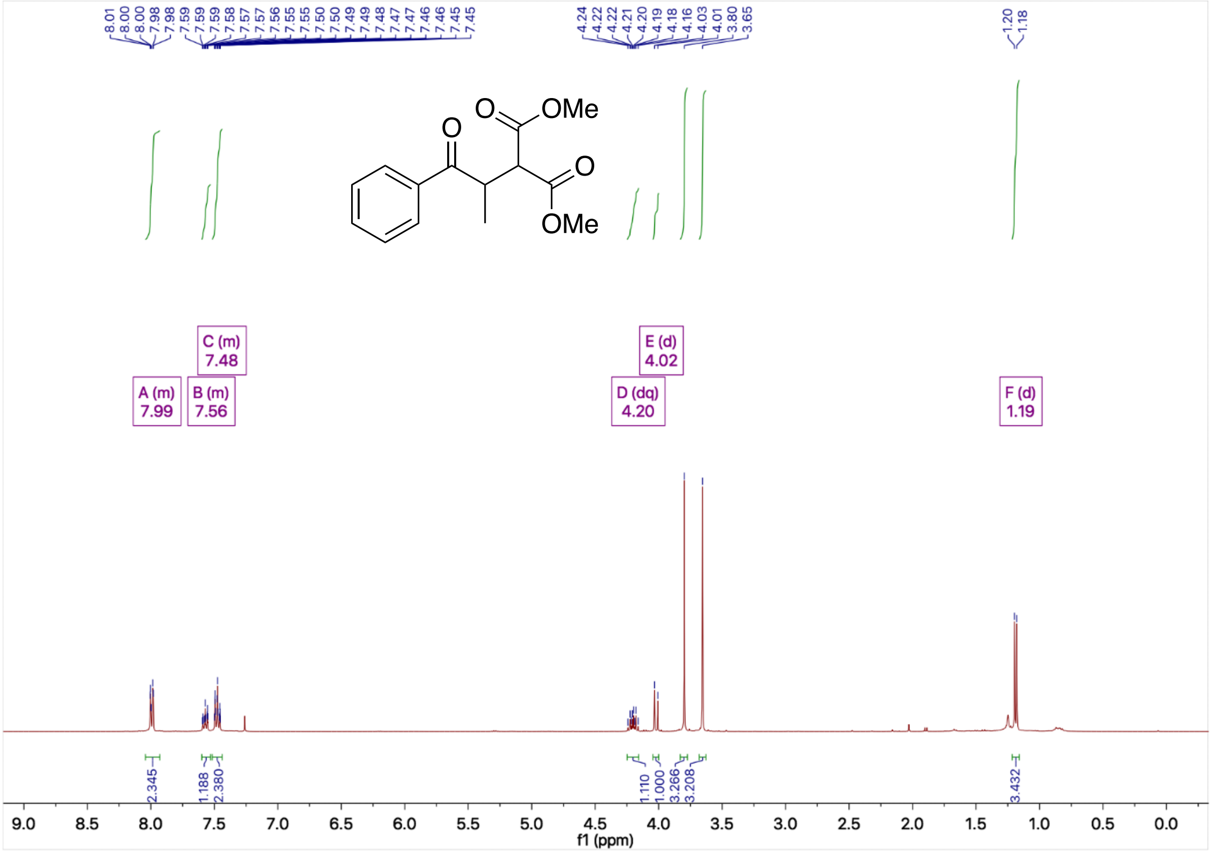


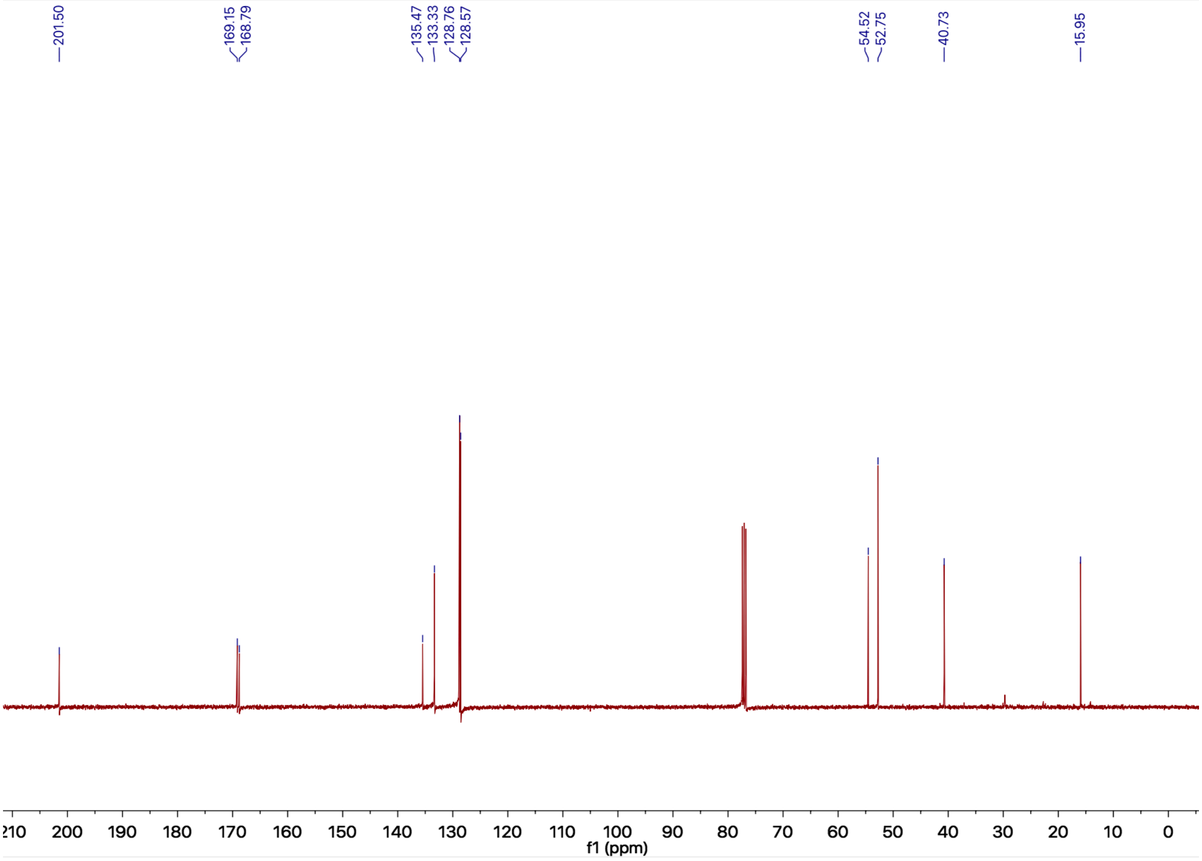

**2-azido-1-phenylpropan-1-one (8l)**

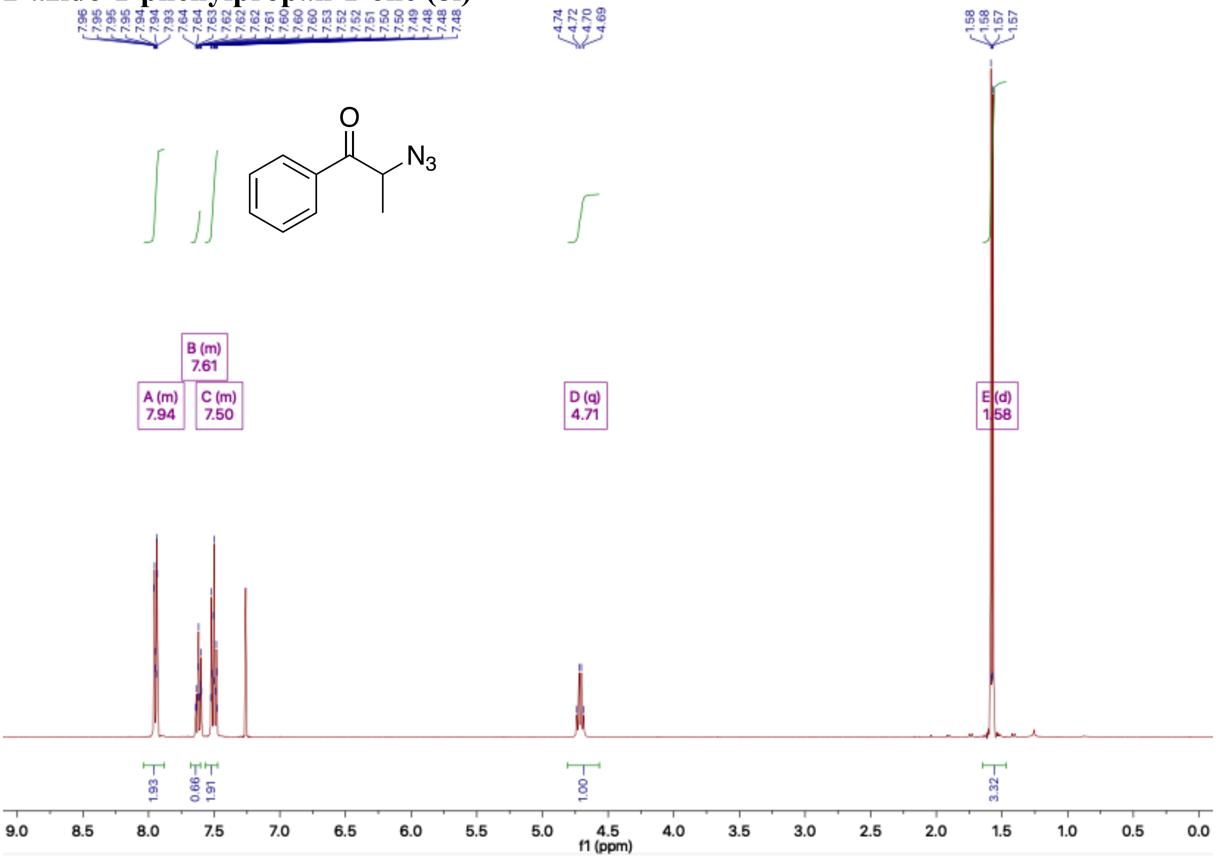

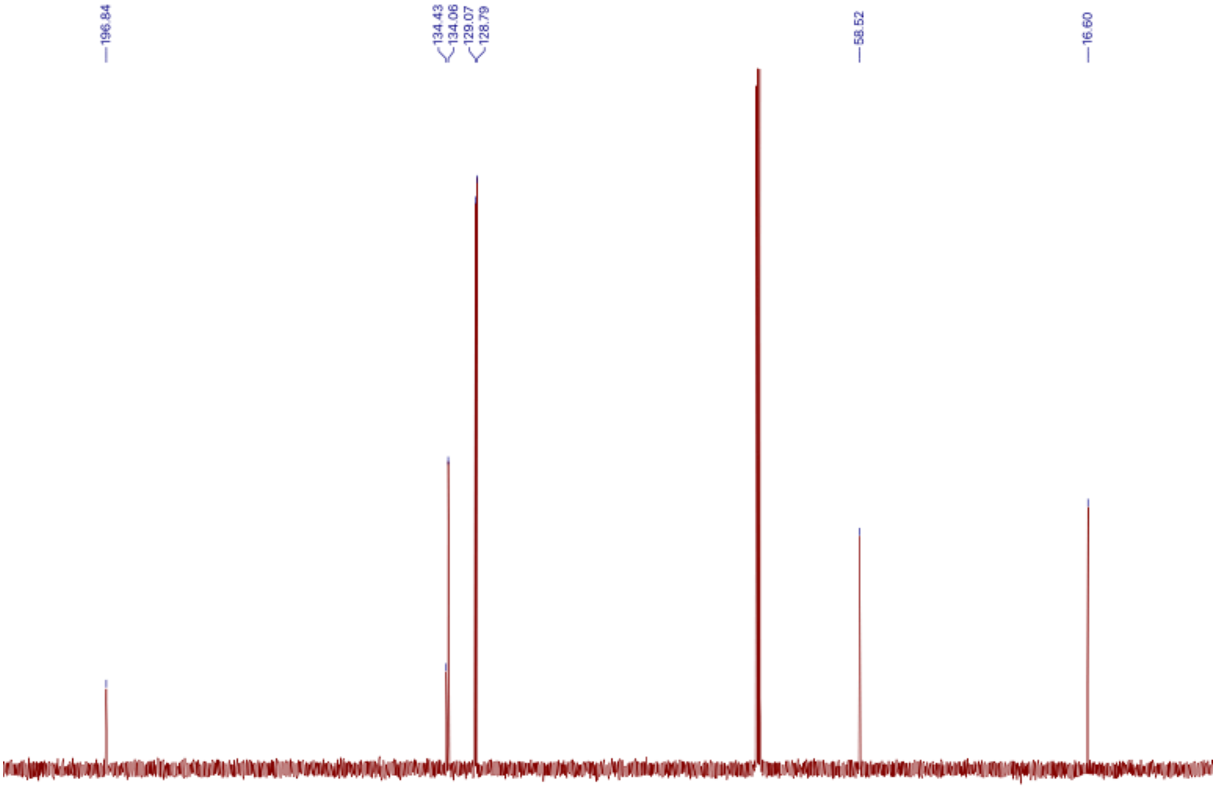

**1-oxo-1-phenylpropan-2-yl (tert-butoxycarbonyl)glycinate (8m)**

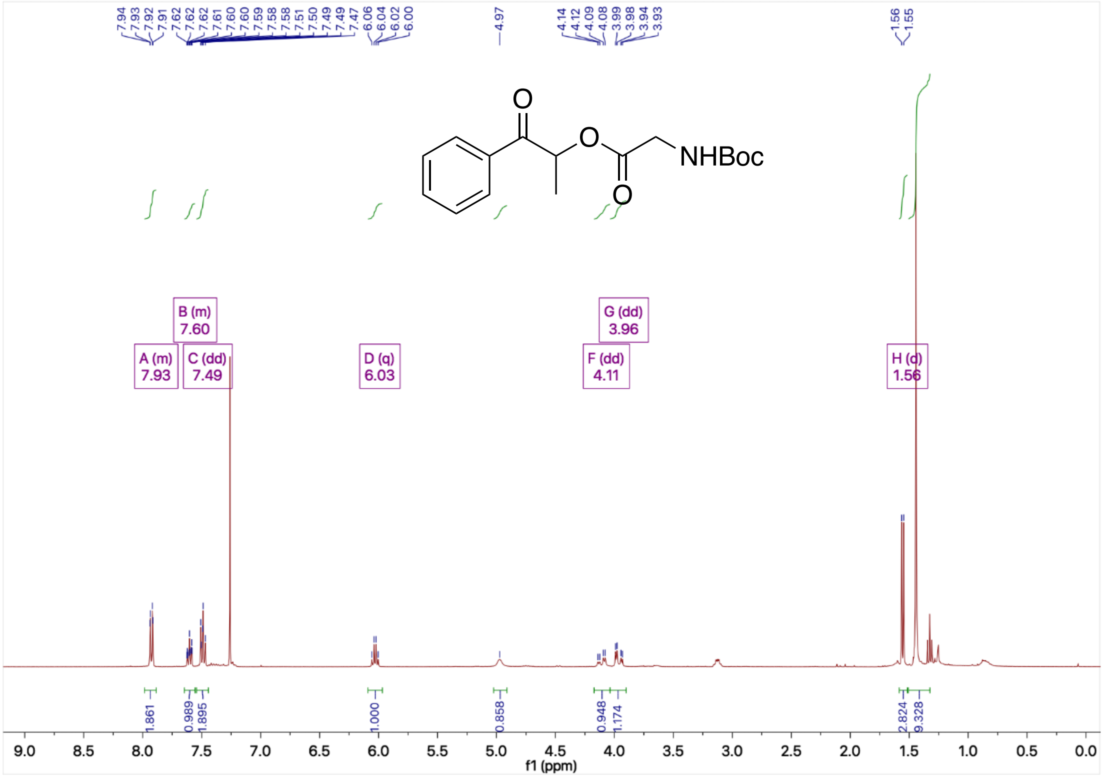

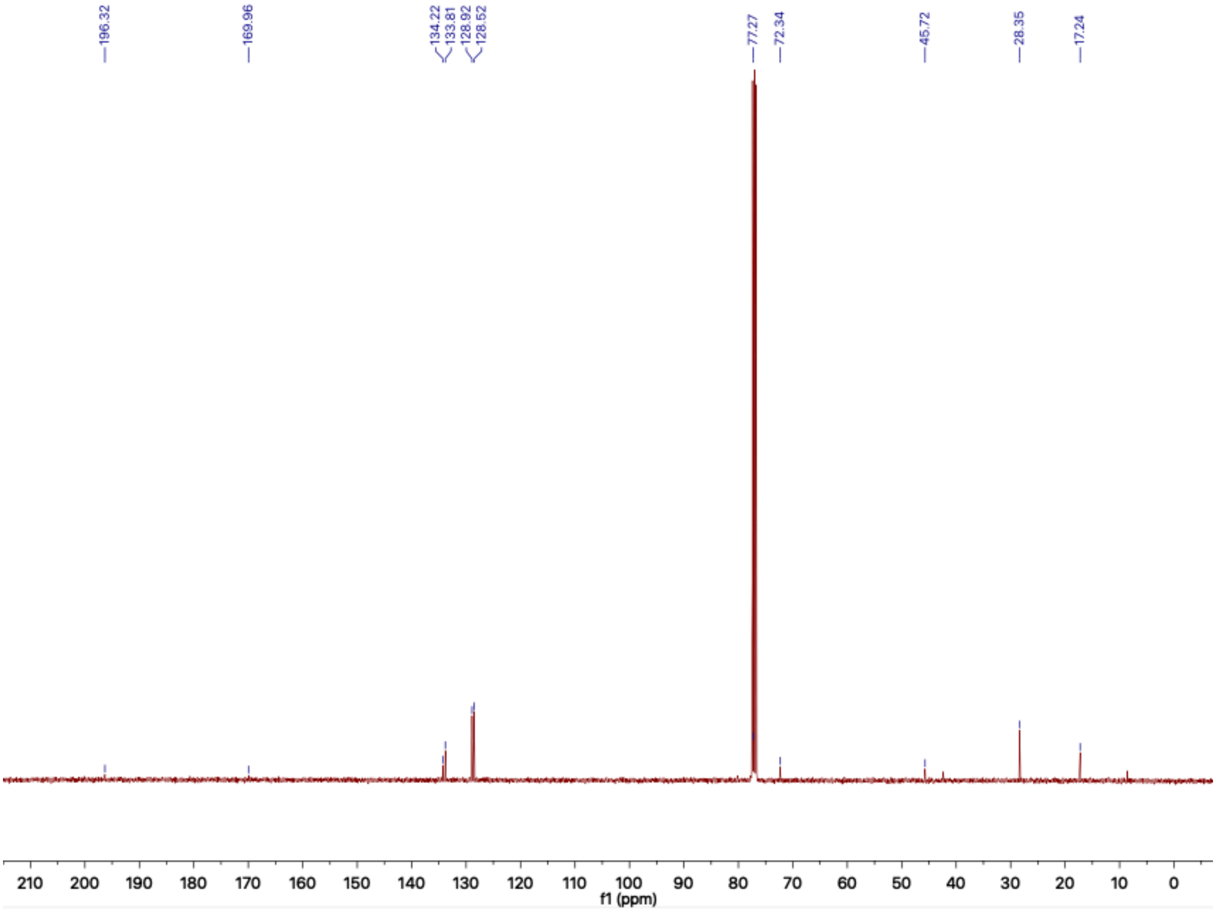

**1-oxo-1-phenylpropan-2-yl 5-((3aS,4S,6aR)-2-oxohexahydro-1H-thieno[3,4-d]imidazol-4-yl)pentanoate (8n)** (Mixture 1:1 of diastereomers)

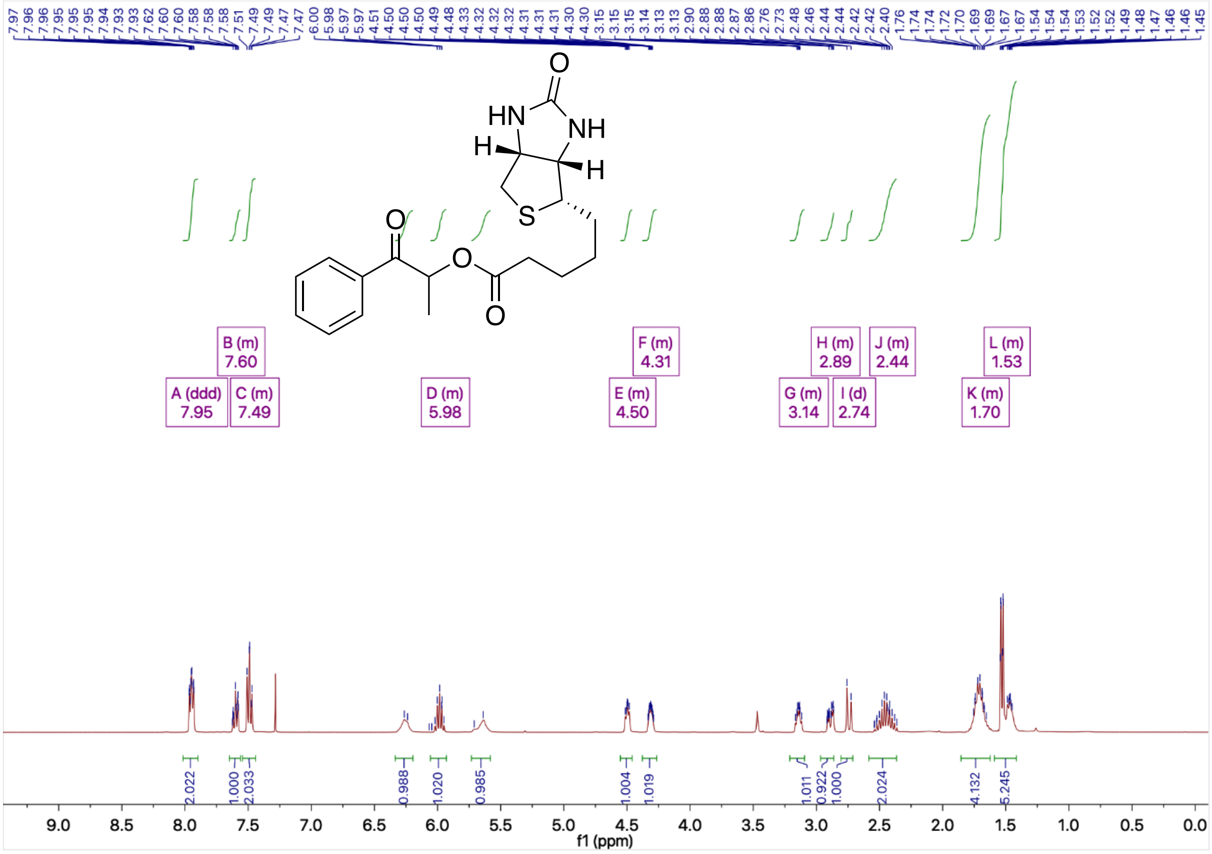

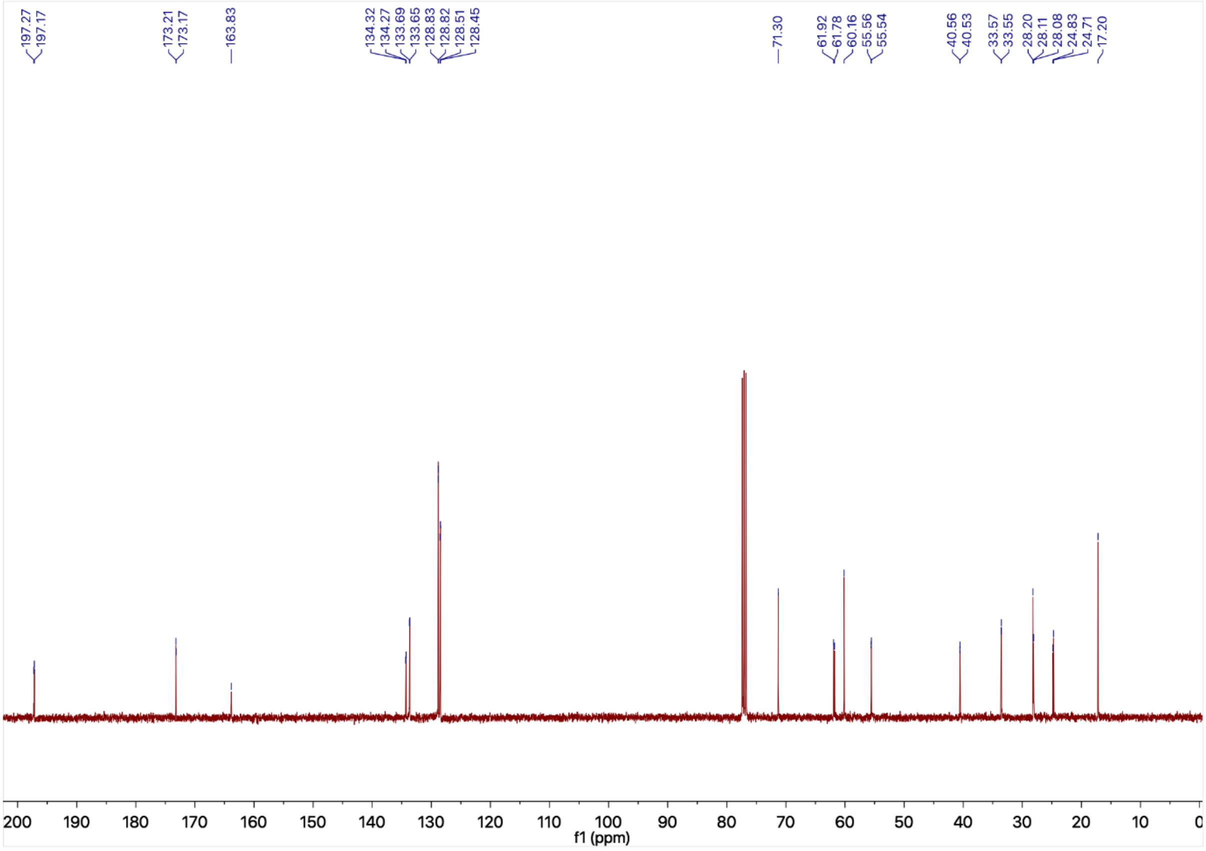

**1-oxo-1-phenylpropan-2-yl 2-acetoxybenzoate (8o)**

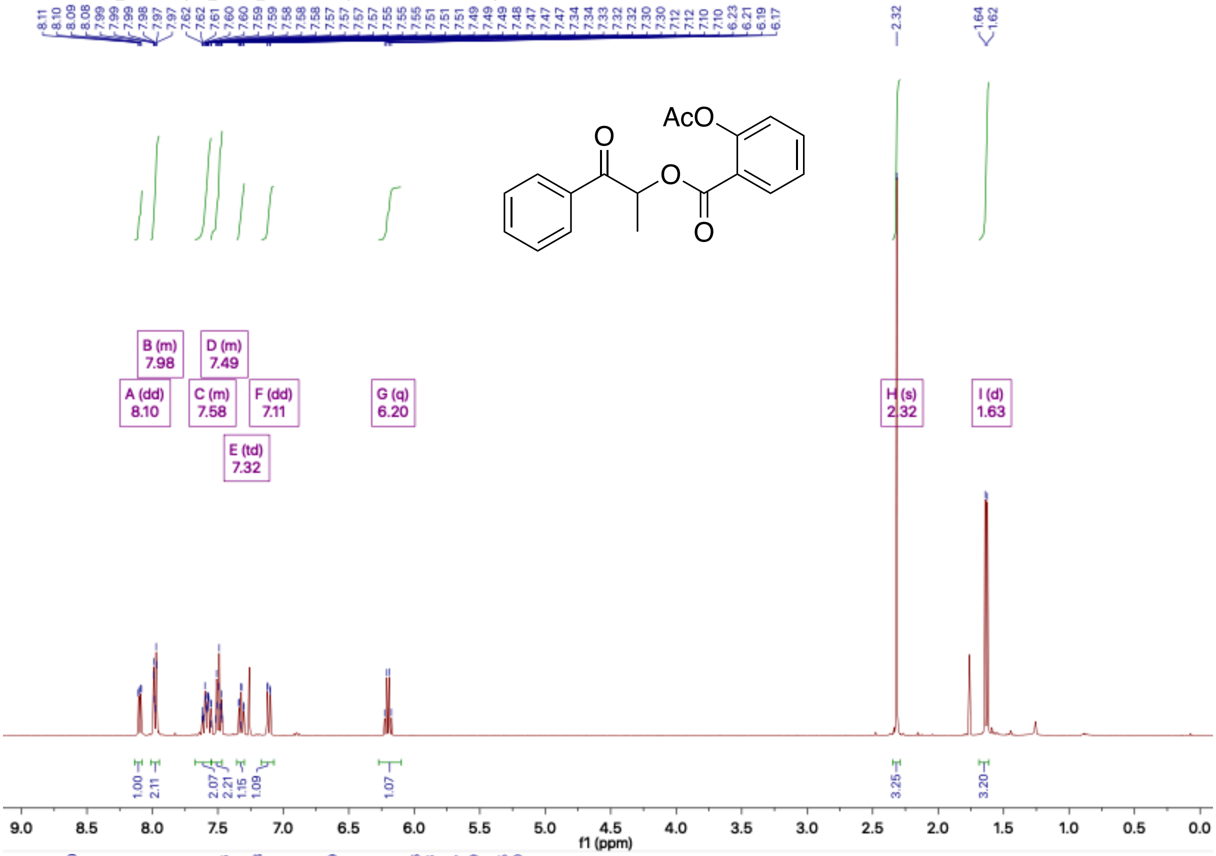


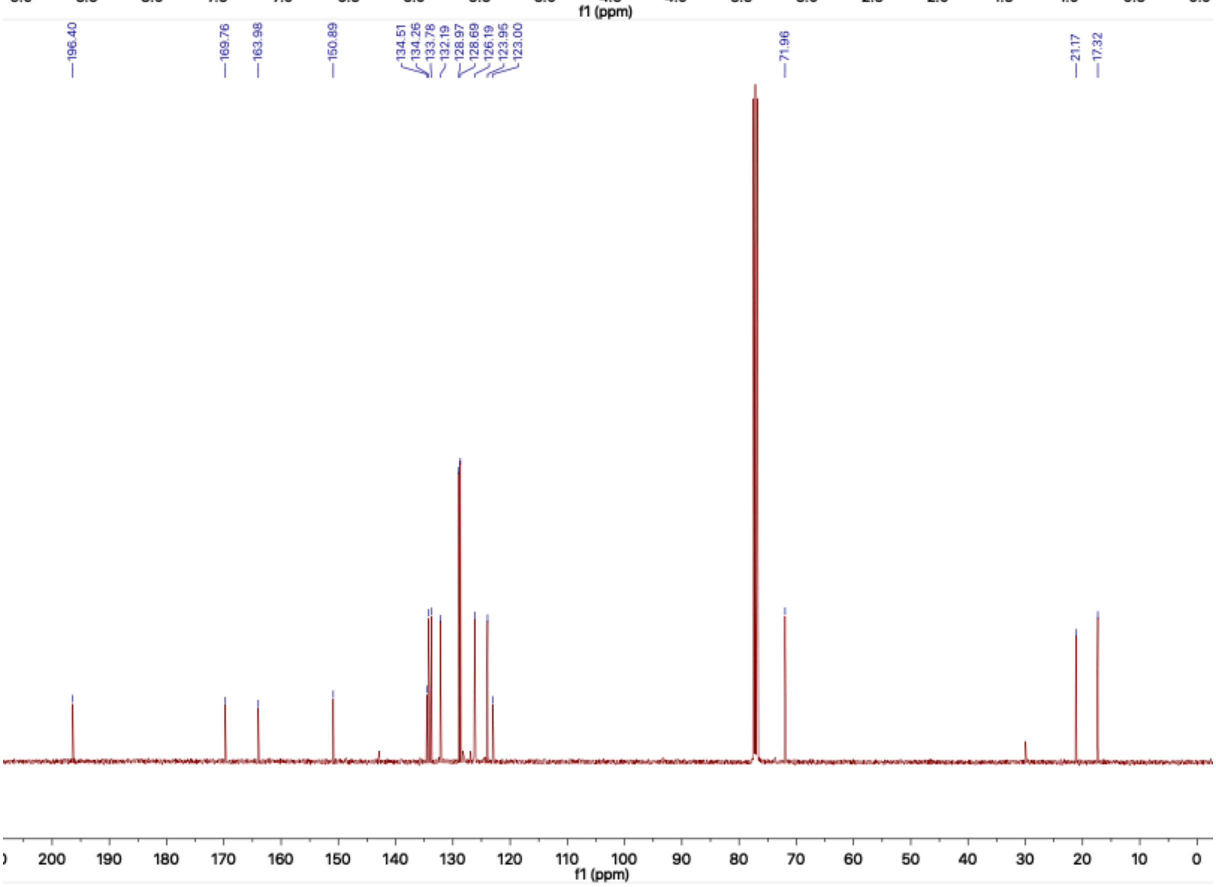

**1-oxo-1-phenylpropan-2-yl 2-(4-isobutylphenyl)propanoate (8p)** (Mixture 1:1 of diastereomers)

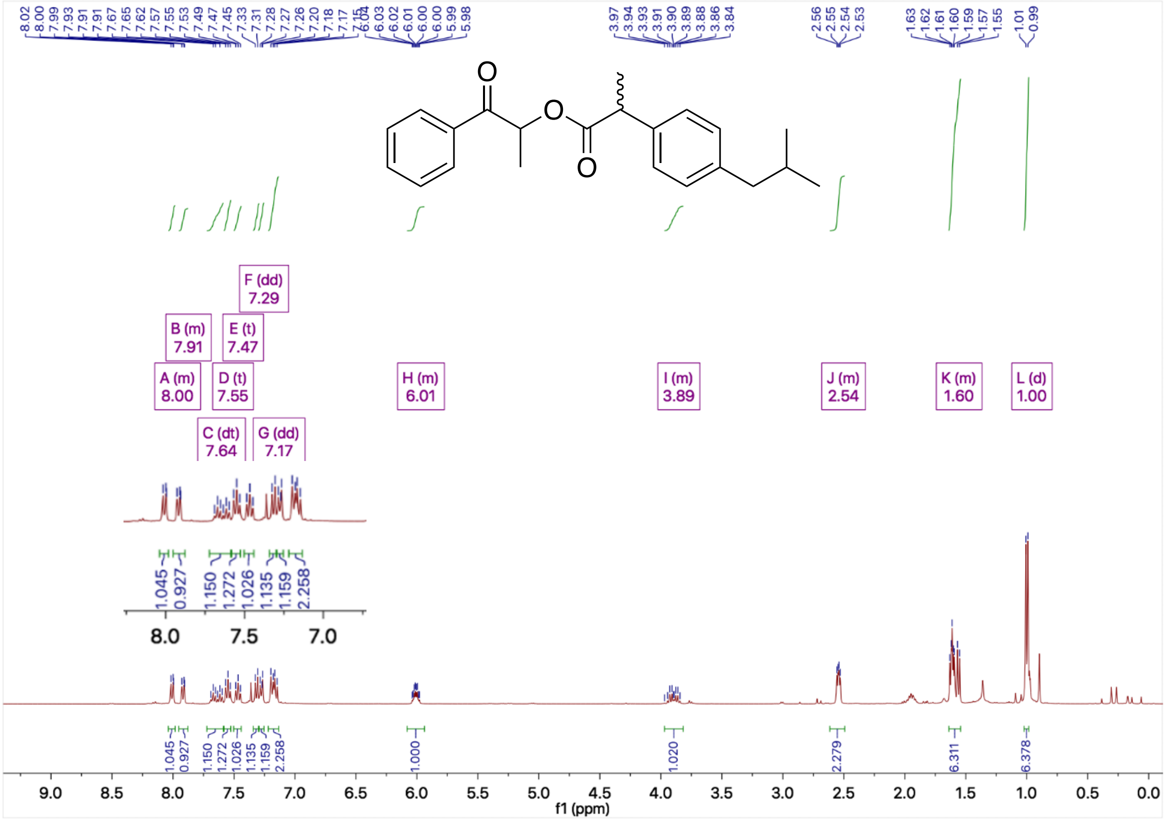

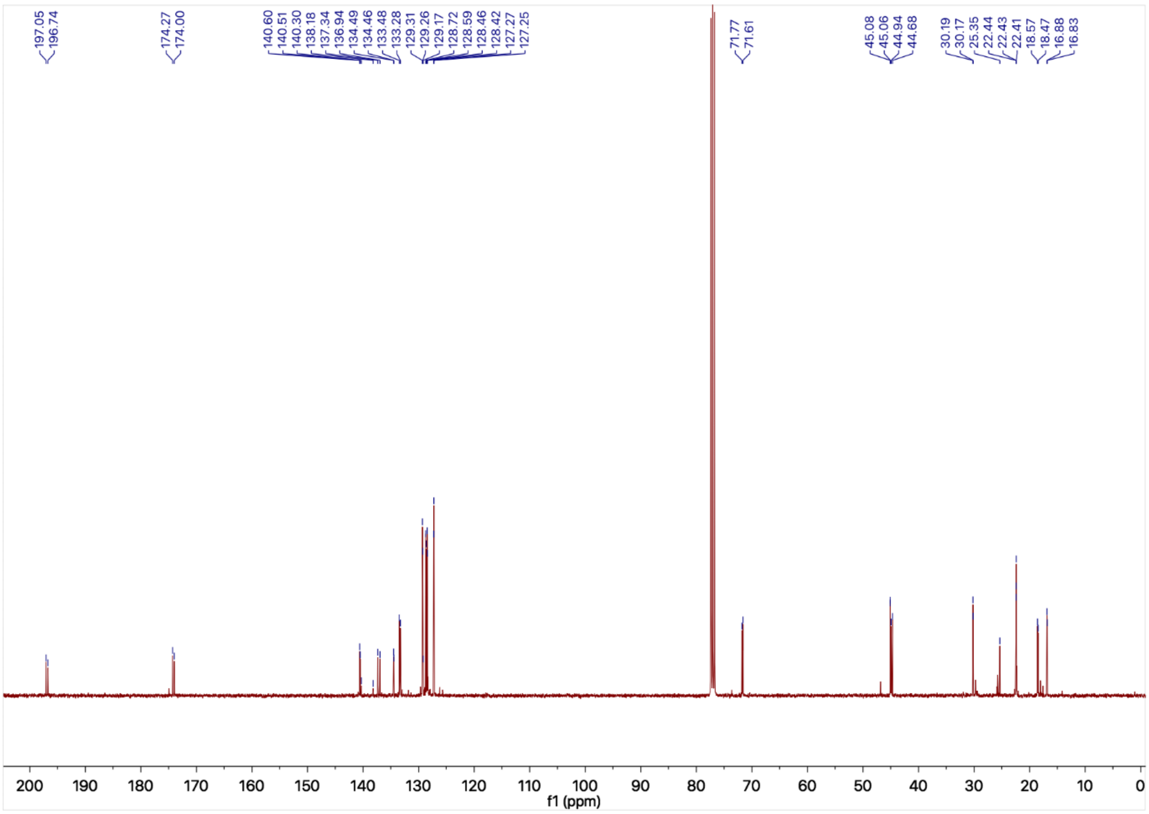

**1-oxo-1-phenylpropan-2-yl (Z)-hexadec-8-enoate (8q)**

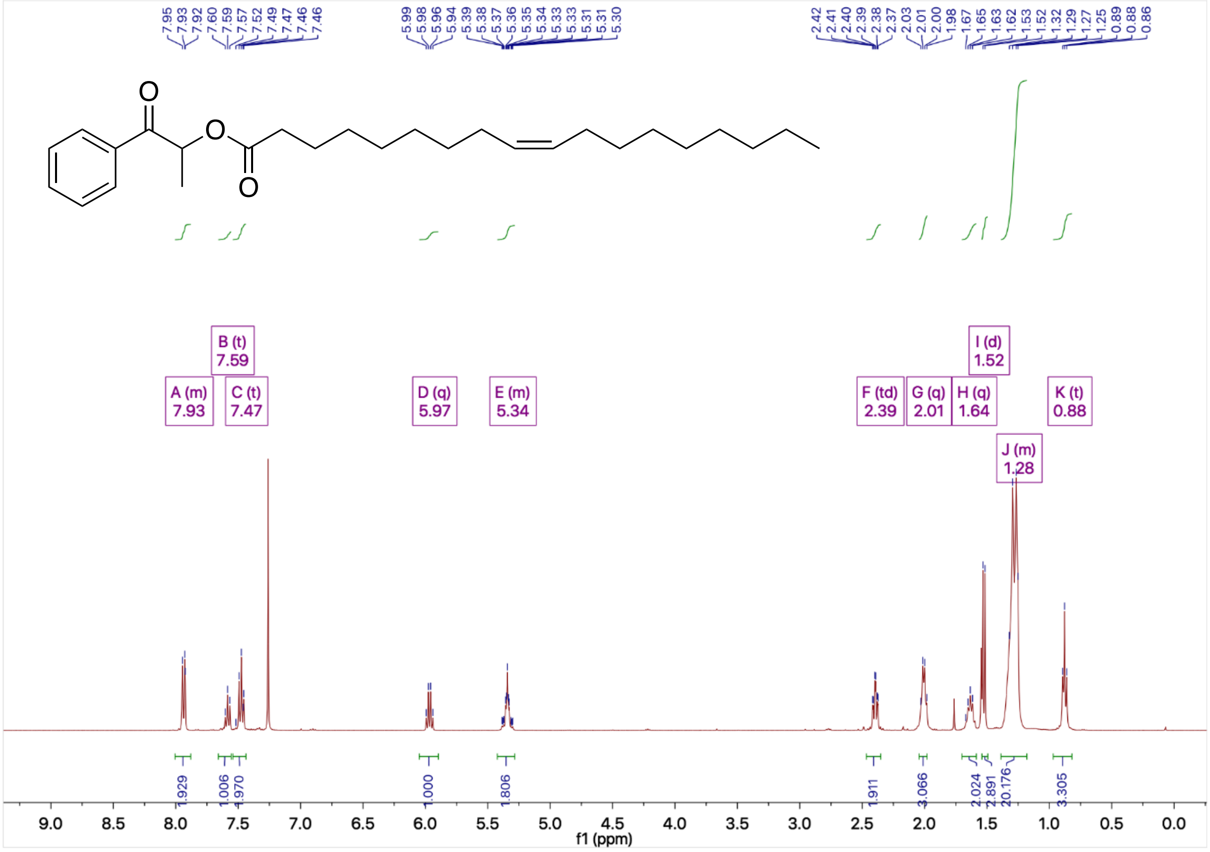

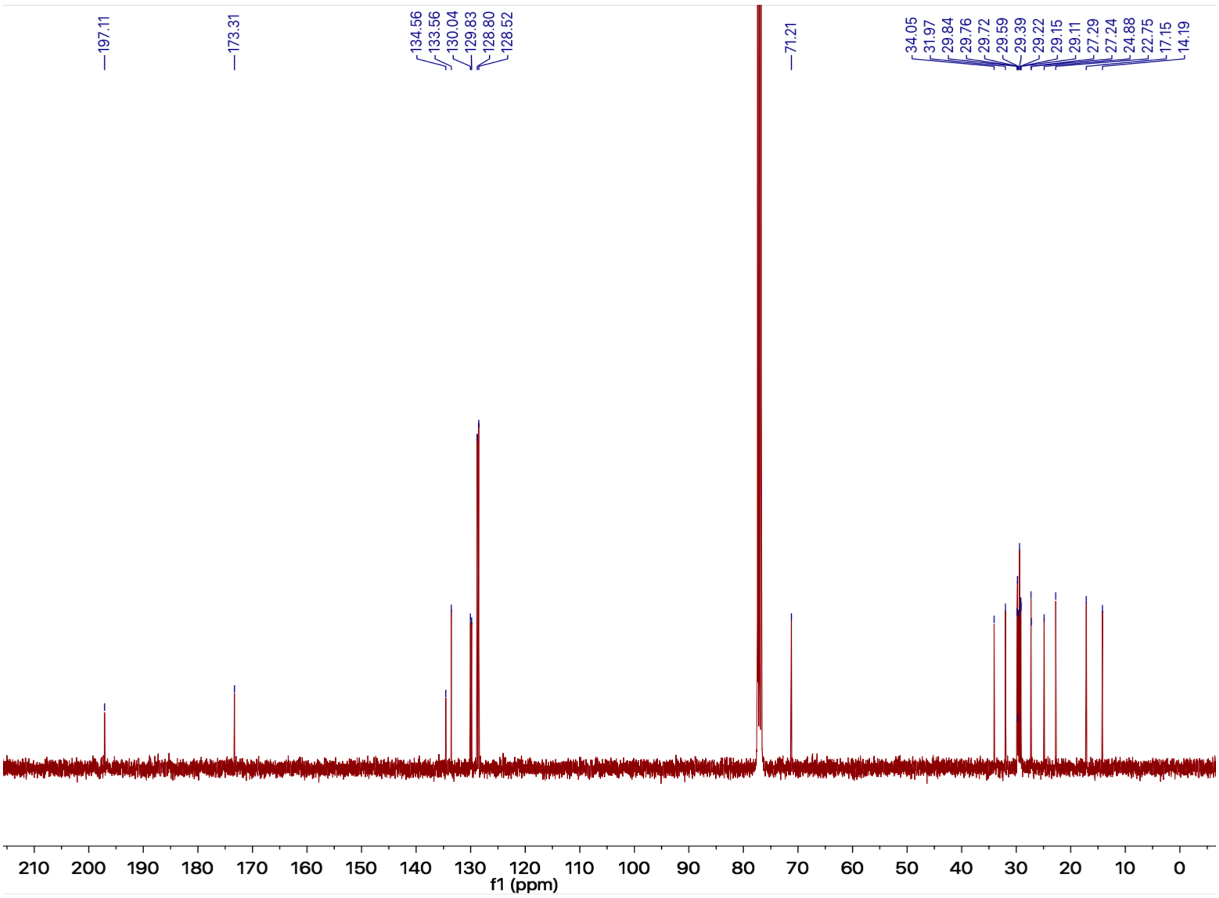

# Computational details

All density functional theory calculations were performed with the Gaussian 16 software.[3] The calculations were performed using B3LYP[4] and M06[5] functional. Geometry optimizations and frequency calculations were performed with the B3LYP functional and 6-31G** basis set for all the atoms except for Iodine and Bromine that SDD ref basis set was used. The energies presented in the manuscript have been refined by single-point calculations on the previously optimized structures, by applying the M06 functional and Def2TZVPP[6] basis set in a solvent model (IEFPCM, solvent = n,n-DiMethylFormamide).[7] The values correspond to Free Gibbs energies and are given in kcal/mol. These energies are relative to the initial mixtures of the enolate and the hypervalent iodine reagent, marked as G = 0.0 kcal/mol. The critical stationary points were characterized by frequency calculations in order to verify that they have the right number of imaginary frequencies, and the intrinsic reaction coordinates (IRC)[8] were followed for $TS_{II\text{-}III}$ to verify the energy profiles connecting the key transition structures to the correct associated local minima for one of the pathways.

# Optimized enolonium intermediates 3D structures

O-enolonium-II

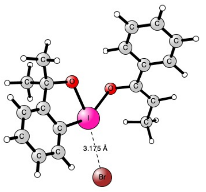


C-enolonium-II´

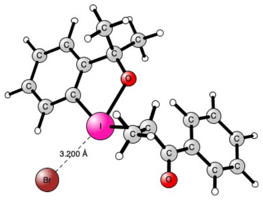


C-enolonium-II

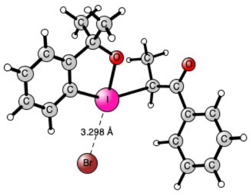


C—enolonium-III

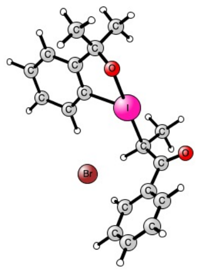

# Cartesian coordinates

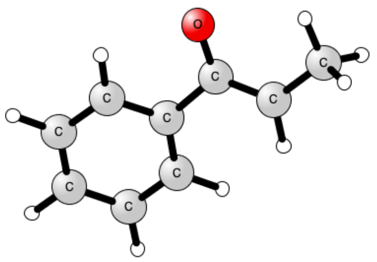

**I**

**Thermal correction to Gibbs Free energy = 0.116643 Hartree**
**Electronic energy = -423.548147508 Hartree**

| Center Number | Atomic Number | Atomic Type | Coordinates (Angstroms) X | Y | Z |
|---|---|---|---|---|---|
| 1 | 1 | 0 | 4.164417 | -1.070647 | -0.719006 |
| 2 | 6 | 0 | 3.637003 | -0.523143 | 0.085226 |
| 3 | 1 | 0 | 3.826702 | 0.546216 | -0.057327 |
| 4 | 1 | 0 | 4.133075 | -0.830630 | 1.025561 |
| 5 | 6 | 0 | 2.151945 | -0.727364 | 0.086398 |

| | | | | | |
|---|---|---|---|---|---|
| 6 | 1 | 0 | 1.773248 | -1.733566 | 0.245103 |
| 7 | 8 | 0 | 1.614059 | 1.578585 | -0.177766 |
| 8 | 6 | 0 | 1.287877 | 0.348972 | -0.047650 |
| 9 | 1 | 0 | -0.577985 | 2.139555 | 0.098978 |
| 10 | 6 | 0 | -1.068252 | 1.170991 | 0.068379 |
| 11 | 6 | 0 | -0.210520 | 0.059873 | -0.019355 |
| 12 | 6 | 0 | -2.454349 | 1.025432 | 0.102957 |
| 13 | 6 | 0 | -0.811979 | -1.209958 | -0.095536 |
| 14 | 1 | 0 | -3.090846 | 1.906947 | 0.176733 |
| 15 | 1 | 0 | -0.185881 | -2.091259 | -0.198694 |
| 16 | 6 | 0 | -3.034188 | -0.244605 | 0.038439 |
| 17 | 6 | 0 | -2.198628 | -1.362235 | -0.065217 |
| 18 | 1 | 0 | -4.115924 | -0.363711 | 0.059356 |
| 19 | 1 | 0 | -2.632730 | -2.359373 | -0.130419 |

---------------------------------------------------------------------------------------------

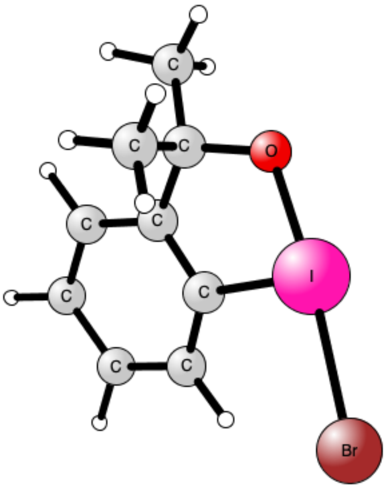


**2c**
**Thermal correction to Gibbs Free energy = 0.126542 Hartree**
**Electronic energy = -3295.85408326 Hartree**

| Center Number | Atomic Number | Atomic Type | Coordinates (Angstroms) X | Y | Z |
|---|---|---|---|---|---|
| 1 | 35 | 0 | -3.098141 | 0.383553 | 0.156129 |
| 2 | 1 | 0 | 3.524473 | -0.856747 | -1.735939 |
| 3 | 53 | 0 | -0.581346 | -1.001017 | -0.164799 |
| 4 | 6 | 0 | 3.664622 | -1.144567 | -0.691709 |
| 5 | 1 | 0 | -1.080328 | 2.237523 | -0.228491 |
| 6 | 1 | 0 | 3.893503 | -2.212297 | -0.650845 |
| 7 | 6 | 0 | -0.004905 | 2.110292 | -0.159581 |
| 8 | 1 | 0 | 4.517524 | -0.596571 | -0.278826 |
| 9 | 6 | 0 | 0.569850 | 0.857051 | -0.105685 |
| 10 | 8 | 0 | 1.403655 | -1.715405 | -0.482908 |
| 11 | 6 | 0 | 0.869123 | 3.202469 | -0.115346 |
| 12 | 6 | 0 | 2.384613 | -0.875626 | 0.115924 |

| | | | | | |
|---|---|---|---|---|---|
| 13 | 6 | 0 | 1.924765 | 0.583785 | -0.000484 |
| 14 | 1 | 0 | 0.464936 | 4.208621 | -0.164423 |
| 15 | 6 | 0 | 2.245186 | 2.994673 | -0.010401 |
| 16 | 6 | 0 | 2.768461 | 1.701789 | 0.051182 |
| 17 | 6 | 0 | 2.584692 | -1.236105 | 1.601778 |
| 18 | 1 | 0 | 2.918662 | 3.845507 | 0.024867 |
| 19 | 1 | 0 | 3.840838 | 1.557043 | 0.139374 |
| 20 | 1 | 0 | 2.877889 | -2.286602 | 1.683730 |
| 21 | 1 | 0 | 3.360939 | -0.615650 | 2.060600 |
| 22 | 1 | 0 | 1.660152 | -1.090592 | 2.169010 |

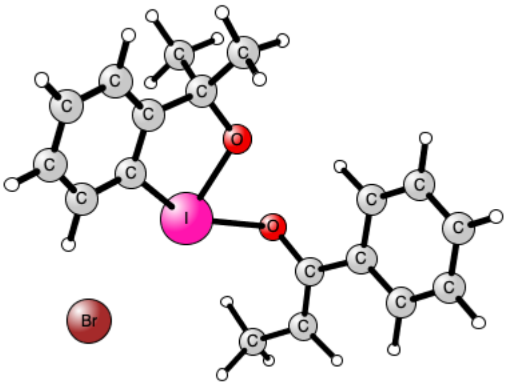


**O-enolonium-II**
**Thermal correction to Gibbs Free energy = 0.261818 Hartree**
**Electronic energy = -3719.42401829 Hartree**

| Center Number | Atomic Number | Atomic Type | Coordinates (Angstroms) X | Y | Z |
|---|---|---|---|---|---|
| 1 | 53 | 0 | -0.436768 | 0.417929 | 0.326810 |
| 2 | 8 | 0 | 1.760991 | 0.483092 | 1.352060 |
| 3 | 8 | 0 | 0.000280 | -1.687043 | 0.649261 |
| 4 | 6 | 0 | -1.146339 | -2.482874 | 0.842533 |
| 5 | 6 | 0 | -2.239395 | -0.614747 | -0.402578 |
| 6 | 6 | 0 | -3.242348 | -0.007127 | -1.147993 |
| 7 | 6 | 0 | -2.296751 | -1.950359 | -0.024150 |
| 8 | 6 | 0 | -4.343529 | -0.775363 | -1.539247 |
| 9 | 6 | 0 | -3.412257 | -2.703329 | -0.411224 |
| 10 | 6 | 0 | -4.428106 | -2.118991 | -1.170139 |
| 11 | 6 | 0 | 2.751871 | 0.926029 | 0.597191 |
| 12 | 6 | 0 | 3.099039 | 2.248070 | 0.545248 |
| 13 | 1 | 0 | -5.285986 | -2.714870 | -1.472203 |
| 14 | 1 | 0 | -3.490306 | -3.748320 | -0.122666 |

| | | | | | |
|---|---|---|---|---|---|
| 15 | 1 | 0 | -3.169764 | 1.045540 | -1.405055 |
| 16 | 1 | 0 | -5.133418 | -0.318798 | -2.130185 |
| 17 | 35 | 0 | -1.768624 | 3.201215 | -0.423561 |
| 18 | 6 | 0 | 3.535427 | -0.115969 | -0.152961 |
| 19 | 6 | 0 | 3.268998 | -1.469982 | 0.115264 |
| 20 | 6 | 0 | 4.522584 | 0.179898 | -1.111929 |
| 21 | 6 | 0 | 3.976546 | -2.484124 | -0.531284 |
| 22 | 6 | 0 | 5.230150 | -0.833208 | -1.756143 |
| 23 | 6 | 0 | 4.965577 | -2.175166 | -1.466499 |
| 24 | 1 | 0 | 2.482270 | -1.697023 | 0.825116 |
| 25 | 1 | 0 | 3.748982 | -3.523870 | -0.305418 |
| 26 | 1 | 0 | 4.731039 | 1.212733 | -1.373057 |
| 27 | 1 | 0 | 5.985949 | -0.574288 | -2.494814 |
| 28 | 1 | 0 | 5.515605 | -2.965593 | -1.972082 |
| 29 | 6 | 0 | 2.367366 | 3.326278 | 1.283404 |
| 30 | 1 | 0 | 3.978143 | 2.545408 | -0.020086 |
| 31 | 1 | 0 | 3.053250 | 3.943257 | 1.883176 |
| 32 | 1 | 0 | 1.623582 | 2.885676 | 1.950938 |
| 33 | 1 | 0 | 1.839154 | 4.010241 | 0.603948 |
| 34 | 6 | 0 | -1.572021 | -2.470818 | 2.328830 |
| 35 | 1 | 0 | -2.444515 | -3.110367 | 2.505837 |
| 36 | 1 | 0 | -1.826179 | -1.454100 | 2.643163 |
| 37 | 1 | 0 | -0.740620 | -2.821179 | 2.948530 |
| 38 | 6 | 0 | -0.719464 | -3.900476 | 0.415601 |
| 39 | 1 | 0 | -0.446155 | -3.907647 | -0.642296 |
| 40 | 1 | 0 | -1.517617 | -4.631946 | 0.584803 |
| 41 | 1 | 0 | 0.152869 | -4.202494 | 1.001944 |

---------------------------------------------------------------------------------

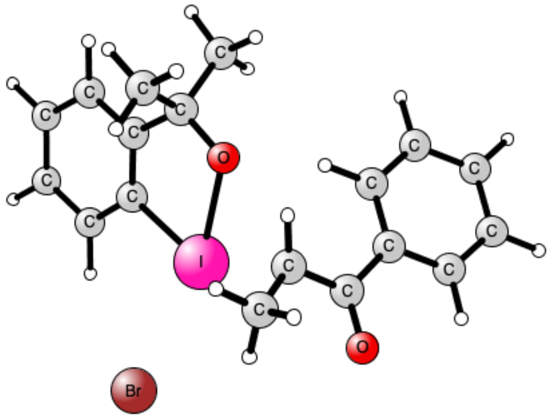


**C-enolonium-II**
**Thermal correction to Gibbs Free energy = 0.263687 Hartree**
**Electronic energy = -3719.422314 Hartree**

| Center Number | Atomic Number | Atomic Type | Coordinates (Angstroms) X | Y | Z |
|---|---|---|---|---|---|
| 1 | 53 | 0 | -0.412299 | -0.581957 | 0.355690 |

| | | | | | |
|---|---|---|---|---|---|
| 2 | 6 | 0 | 1.782561 | -0.540509 | 1.692811 |
| 3 | 6 | 0 | 2.835640 | -0.998249 | 0.782010 |
| 4 | 8 | 0 | 3.252769 | -2.164894 | 0.826551 |
| 5 | 8 | 0 | 0.083413 | 1.626276 | 0.965435 |
| 6 | 6 | 0 | -0.910354 | 2.579249 | 0.807758 |
| 7 | 6 | 0 | -2.122217 | 0.675730 | -0.336290 |
| 8 | 6 | 0 | -2.095327 | 2.016189 | -0.010044 |
| 9 | 6 | 0 | -3.160780 | 0.084678 | -1.050407 |
| 10 | 6 | 0 | -3.178210 | 2.810537 | -0.420731 |
| 11 | 6 | 0 | -4.225578 | 0.892161 | -1.458027 |
| 12 | 6 | 0 | -4.233440 | 2.253086 | -1.141658 |
| 13 | 1 | 0 | -5.063235 | 2.881957 | -1.455457 |
| 14 | 1 | 0 | -3.197003 | 3.869323 | -0.174612 |
| 15 | 1 | 0 | -3.141363 | -0.979774 | -1.271192 |
| 16 | 1 | 0 | -5.046623 | 0.453119 | -2.019061 |
| 17 | 6 | 0 | 3.461361 | -0.037506 | -0.202132 |
| 18 | 35 | 0 | -1.878253 | -3.237190 | -0.664466 |
| 19 | 6 | 0 | -0.299762 | 3.792332 | 0.063386 |
| 20 | 6 | 0 | -1.430445 | 3.008740 | 2.203616 |
| 21 | 1 | 0 | -1.015278 | 4.612666 | -0.064493 |
| 22 | 1 | 0 | 0.555859 | 4.167269 | 0.634344 |
| 23 | 1 | 0 | 0.052574 | 3.479891 | -0.923758 |
| 24 | 1 | 0 | -0.597253 | 3.407602 | 2.791871 |
| 25 | 1 | 0 | -1.834565 | 2.138103 | 2.728501 |
| 26 | 1 | 0 | -2.215179 | 3.772077 | 2.143158 |
| 27 | 6 | 0 | 1.474945 | -1.457008 | 2.851985 |
| 28 | 1 | 0 | 1.224400 | -2.466125 | 2.511620 |
| 29 | 1 | 0 | 0.649201 | -1.070251 | 3.455647 |
| 30 | 1 | 0 | 2.356708 | -1.560953 | 3.501605 |
| 31 | 6 | 0 | 4.620067 | -0.465684 | -0.868716 |
| 32 | 6 | 0 | 2.956230 | 1.244994 | -0.469378 |
| 33 | 6 | 0 | 3.610622 | 2.077350 | -1.380129 |
| 34 | 6 | 0 | 5.272502 | 0.369212 | -1.772359 |
| 35 | 6 | 0 | 4.769446 | 1.647724 | -2.030020 |
| 36 | 1 | 0 | 4.983033 | -1.465801 | -0.655641 |
| 37 | 1 | 0 | 6.170888 | 0.024598 | -2.279055 |
| 38 | 1 | 0 | 5.274030 | 2.301469 | -2.737679 |
| 39 | 1 | 0 | 3.206273 | 3.065305 | -1.586809 |
| 40 | 1 | 0 | 2.037765 | 1.582075 | 0.005409 |
| 41 | 1 | 0 | 1.737468 | 0.523617 | 1.894421 |

---------------------------------------------------------------------------------------

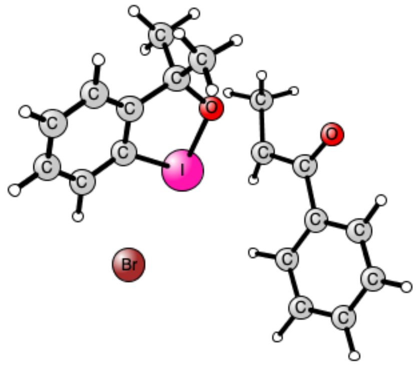


**C-enolonium-II′**
**Thermal correction to Gibbs Free energy = 0.263181Hartree**
**Electronic energy = -3719.426807 Hartree**

| Center Number | Atomic Number | Atomic Type | Coordinates (Angstroms) X | Y | Z |
|---|---|---|---|---|---|
| 1 | 53 | 0 | -0.339349 | 0.232992 | 0.432605 |
| 2 | 6 | 0 | 1.525559 | -1.078542 | 1.603438 |
| 3 | 6 | 0 | 2.341634 | -1.782897 | 0.614966 |
| 4 | 8 | 0 | 2.311278 | -3.016689 | 0.506786 |
| 5 | 8 | 0 | -0.990182 | -1.856668 | 0.104658 |
| 6 | 6 | 0 | -2.370821 | -2.049397 | -0.040901 |
| 7 | 6 | 0 | -2.336226 | 0.391556 | -0.560221 |
| 8 | 6 | 0 | -3.036342 | -0.797251 | -0.645715 |
| 9 | 6 | 0 | -2.843629 | 1.603713 | -1.014488 |
| 10 | 6 | 0 | -4.315317 | -0.773930 | -1.219451 |
| 11 | 6 | 0 | -4.115399 | 1.613012 | -1.596856 |
| 12 | 6 | 0 | -4.846666 | 0.426048 | -1.697268 |
| 13 | 1 | 0 | -5.836281 | 0.434354 | -2.147940 |
| 14 | 1 | 0 | -4.899877 | -1.687685 | -1.295457 |
| 15 | 1 | 0 | -2.263991 | 2.518038 | -0.912185 |
| 16 | 1 | 0 | -4.532987 | 2.545228 | -1.969441 |
| 17 | 6 | 0 | 3.275734 | -0.998876 | -0.285963 |
| 18 | 35 | 0 | 0.359510 | 3.445520 | 0.691527 |
| 19 | 6 | 0 | -3.020041 | -2.348740 | 1.331417 |
| 20 | 6 | 0 | -2.514682 | -3.262438 | -0.983767 |
| 21 | 1 | 0 | -4.099841 | -2.518189 | 1.246097 |
| 22 | 1 | 0 | -2.555770 | -3.237868 | 1.770301 |
| 23 | 1 | 0 | -2.860215 | -1.507371 | 2.012971 |
| 24 | 1 | 0 | -1.972626 | -4.111761 | -0.558526 |
| 25 | 1 | 0 | -2.080499 | -3.029437 | -1.959156 |
| 26 | 1 | 0 | -3.563172 | -3.551629 | -1.119238 |
| 27 | 6 | 0 | 0.885424 | -1.934546 | 2.669281 |
| 28 | 1 | 0 | 0.256612 | -2.697566 | 2.208924 |
| 29 | 1 | 0 | 0.273990 | -1.330524 | 3.347552 |
| 30 | 1 | 0 | 1.649316 | -2.451209 | 3.269210 |
| 31 | 6 | 0 | 4.109381 | -1.739170 | -1.139317 |
| 32 | 6 | 0 | 3.366788 | 0.402026 | -0.309321 |
| 33 | 6 | 0 | 4.266516 | 1.040287 | -1.165443 |

| | | | | | |
|---|---|---|---|---|---|
| 34 | 6 | 0 | 5.011182 | -1.104005 | -1.989041 |
| 35 | 6 | 0 | 5.092936 | 0.291606 | -2.004976 |
| 36 | 1 | 0 | 4.020071 | -2.820169 | -1.109339 |
| 37 | 1 | 0 | 5.649693 | -1.694680 | -2.642145 |
| 38 | 1 | 0 | 5.792851 | 0.791779 | -2.670171 |
| 39 | 1 | 0 | 4.309992 | 2.125695 | -1.175039 |
| 40 | 1 | 0 | 2.734271 | 1.019843 | 0.319519 |
| 41 | 1 | 0 | 1.956182 | -0.142485 | 1.962753 |

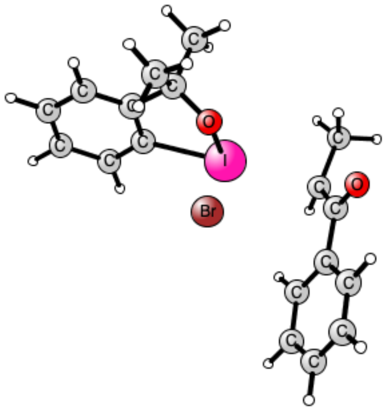


**$TS_{II\text{-}III}$**
**Thermal correction to Gibbs Free energy = 0.263578 Hartree**
**Electronic energy = -3719.40155282 Hartree**

| Center Number | Atomic Number | Atomic Type | Coordinates (Angstroms) X | Y | Z |
|---|---|---|---|---|---|
| 1 | 53 | 0 | -0.351721 | -0.190531 | 0.333420 |
| 2 | 6 | 0 | 2.056309 | -0.227210 | 1.748980 |
| 3 | 6 | 0 | 2.784870 | -1.146700 | 0.936380 |
| 4 | 8 | 0 | 2.800310 | -2.384860 | 1.143500 |
| 5 | 8 | 0 | -1.387880 | -2.087521 | -0.354180 |
| 6 | 6 | 0 | -2.780680 | -1.985462 | -0.178590 |
| 7 | 6 | 0 | -2.376721 | 0.486229 | -0.497170 |
| 8 | 6 | 0 | -3.252811 | -0.595132 | -0.623360 |
| 9 | 6 | 0 | -2.718301 | 1.780508 | -0.848150 |
| 10 | 6 | 0 | -4.534891 | -0.324662 | -1.122460 |
| 11 | 6 | 0 | -4.000832 | 2.023258 | -1.355920 |
| 12 | 6 | 0 | -4.905991 | 0.971078 | -1.488550 |
| 13 | 1 | 0 | -5.903541 | 1.154617 | -1.880240 |
| 14 | 1 | 0 | -5.250220 | -1.135952 | -1.232170 |
| 15 | 1 | 0 | -2.004312 | 2.587239 | -0.717210 |
| 16 | 1 | 0 | -4.284592 | 3.033068 | -1.641790 |
| 17 | 6 | 0 | 3.569319 | -0.625749 | -0.258540 |
| 18 | 35 | 0 | 0.209548 | 2.793219 | 0.774850 |
| 19 | 6 | 0 | -3.192400 | -2.222132 | 1.294360 |
| 20 | 6 | 0 | -3.360030 | -3.109392 | -1.064150 |

| | | | | | |
|---|---|---|---|---|---|
| 21 | 1 | 0 | -4.281340 | -2.197382 | 1.417260 |
| 22 | 1 | 0 | -2.818810 | -3.197642 | 1.621850 |
| 23 | 1 | 0 | -2.761200 | -1.455162 | 1.944050 |
| 24 | 1 | 0 | -2.906419 | -4.058412 | -0.766370 |
| 25 | 1 | 0 | -3.120900 | -2.923922 | -2.114160 |
| 26 | 1 | 0 | -4.447390 | -3.196852 | -0.953570 |
| 27 | 6 | 0 | 1.553579 | -0.708710 | 3.08745 |
| 28 | 1 | 0 | 1.110950 | -1.706940 | 2.999310 |
| 29 | 1 | 0 | 0.808129 | -0.025370 | 3.509990 |
| 30 | 1 | 0 | 2.368859 | -0.800300 | 3.823840 |
| 31 | 6 | 0 | 4.401950 | -1.537839 | -0.924640 |
| 32 | 6 | 0 | 3.522249 | 0.697711 | -0.726670 |
| 33 | 6 | 0 | 4.291969 | 1.092161 | -1.822730 |
| 34 | 6 | 0 | 5.175930 | -1.144089 | -2.015050 |
| 35 | 6 | 0 | 5.125559 | 0.176221 | -2.469600 |
| 36 | 1 | 0 | 4.410680 | -2.557699 | -0.553500 |
| 37 | 1 | 0 | 5.817820 | -1.867199 | -2.514450 |
| 38 | 1 | 0 | 5.725089 | 0.487681 | -3.322200 |
| 39 | 1 | 0 | 4.234778 | 2.119781 | -2.174090 |
| 40 | 1 | 0 | 2.871369 | 1.424750 | -0.251300 |
| 41 | 1 | 0 | 2.284169 | 0.829650 | 1.669740 |

---------------------------------------------------------------------------------

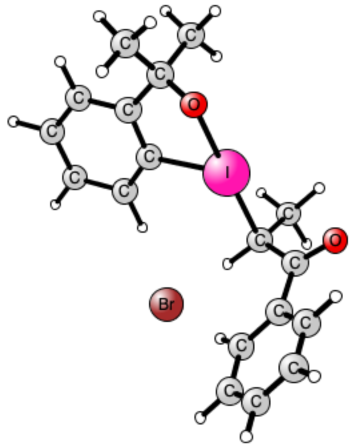


**C-enolonium-III**
**Thermal correction to Gibbs Free energy = 0.265212 Hartree**
**Electronic energy = -3719.44654707 Hartree**

| Center Number | Atomic Number | Atomic Type | Coordinates (Angstroms) X | Y | Z |
|---|---|---|---|---|---|
| 1 | 53 | 0 | -0.692445 | -1.127107 | 0.373740 |
| 2 | 6 | 0 | 1.230049 | -0.048503 | 1.280961 |
| 3 | 6 | 0 | 2.160330 | -1.200353 | 1.070648 |
| 4 | 8 | 0 | 2.074230 | -2.200276 | 1.792492 |
| 5 | 8 | 0 | -2.624788 | -2.107999 | -0.412947 |
| 6 | 6 | 0 | -3.702940 | -1.252423 | -0.290284 |
| 7 | 6 | 0 | -1.922718 | 0.566249 | -0.352005 |
| 8 | 6 | 0 | -3.238268 | 0.191749 | -0.600868 |
| 9 | 6 | 0 | -1.399057 | 1.829960 | -0.548183 |

| | | | | | |
|---|---|---|---|---|---|
| 10 | 6 | 0 | -4.074113 | 1.199325 | -1.103482 |
| 11 | 6 | 0 | -2.264231 | 2.805363 | -1.062241 |
| 12 | 6 | 0 | -3.592301 | 2.488352 | -1.341604 |
| 13 | 1 | 0 | -4.260994 | 3.245710 | -1.743353 |
| 14 | 1 | 0 | -5.114054 | 0.967315 | -1.313253 |
| 15 | 1 | 0 | -0.370085 | 2.105775 | -0.315351 |
| 16 | 1 | 0 | -1.872520 | 3.803830 | -1.231669 |
| 17 | 6 | 0 | 3.130947 | -1.201143 | -0.075245 |
| 18 | 35 | 0 | 1.677502 | 3.458586 | 0.396507 |
| 19 | 6 | 0 | -4.285679 | -1.270290 | 1.151972 |
| 20 | 6 | 0 | -4.786742 | -1.721635 | -1.284430 |
| 21 | 1 | 0 | -5.153330 | -0.608446 | 1.261909 |
| 22 | 1 | 0 | -4.586944 | -2.293950 | 1.398853 |
| 23 | 1 | 0 | -3.527100 | -0.957093 | 1.875917 |
| 24 | 1 | 0 | -5.009113 | -2.770954 | -1.069974 |
| 25 | 1 | 0 | -4.409909 | -1.654137 | -2.308779 |
| 26 | 1 | 0 | -5.720760 | -1.151901 | -1.208314 |
| 27 | 6 | 0 | 0.923886 | 0.260444 | 2.733846 |
| 28 | 1 | 0 | 0.655915 | -0.643499 | 3.286824 |
| 29 | 1 | 0 | 0.123478 | 0.999540 | 2.814948 |
| 30 | 1 | 0 | 1.814257 | 0.700622 | 3.200421 |
| 31 | 6 | 0 | 3.701258 | -2.434843 | -0.435964 |
| 32 | 6 | 0 | 3.502813 | -0.040377 | -0.771487 |
| 33 | 6 | 0 | 4.429453 | -0.125374 | -1.813971 |
| 34 | 6 | 0 | 4.613769 | -2.515018 | -1.482359 |
| 35 | 6 | 0 | 4.980856 | -1.355815 | -2.174529 |
| 36 | 1 | 0 | 3.406548 | -3.314941 | 0.125982 |
| 37 | 1 | 0 | 5.040232 | -3.475567 | -1.760479 |
| 38 | 1 | 0 | 5.696000 | -1.413279 | -2.991606 |
| 39 | 1 | 0 | 4.716892 | 0.778813 | -2.342896 |
| 40 | 1 | 0 | 3.093638 | 0.931210 | -0.501593 |
| 41 | 1 | 0 | 1.425471 | 0.849347 | 0.695440 |

---------------------------------------------------------------------

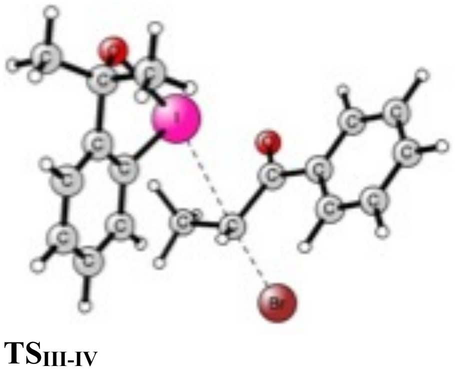

**$TS_{III-IV}$**
**Thermal correction to Gibbs Free energy = 0.261631 Hartree**
**Electronic energy = -3719.417787 Hartree**

| Center Number | Atomic Number | Atomic Type | Coordinates (Angstroms) X | Y | Z |
|---|---|---|---|---|---|

---------------------------------------------------------------------

| | | | | | |
|---|---|---|---|---|---|
| 1 | 6 | 0 | -3.580678 | -2.606797 | 1.839123 |
| 2 | 6 | 0 | -4.181385 | -1.449435 | 1.343840 |
| 3 | 6 | 0 | -3.499466 | -0.548139 | 0.495762 |
| 4 | 6 | 0 | -2.270437 | -2.912685 | 1.493482 |
| 5 | 6 | 0 | -2.182982 | -0.873254 | 0.202245 |
| 6 | 6 | 0 | -1.559125 | -2.026152 | 0.678897 |
| 7 | 1 | 0 | -4.149202 | -3.279174 | 2.478454 |
| 8 | 1 | 0 | -1.788160 | -3.822413 | 1.854547 |
| 9 | 1 | 0 | -5.208611 | -1.227767 | 1.619291 |
| 10 | 1 | 0 | -0.533003 | -2.251504 | 0.400567 |
| 11 | 6 | 0 | -4.162158 | 0.754772 | -0.071734 |
| 12 | 6 | 0 | -4.072833 | 1.827238 | 1.069832 |
| 13 | 1 | 0 | -4.567487 | 1.528013 | 2.001929 |
| 14 | 1 | 0 | -4.532941 | 2.756135 | 0.702678 |
| 15 | 1 | 0 | -3.019783 | 2.040546 | 1.283305 |
| 16 | 6 | 0 | -5.669003 | 0.474377 | -0.383509 |
| 17 | 1 | 0 | -6.284436 | 0.214473 | 0.488875 |
| 18 | 1 | 0 | -5.739901 | -0.327719 | -1.116256 |
| 19 | 1 | 0 | -6.080955 | 1.381766 | -0.831435 |
| 20 | 8 | 0 | -3.577331 | 1.160569 | -1.209927 |
| 21 | 5 | 0 | -0.962541 | 0.478694 | -1.007230 |
| 22 | 8 | 0 | 2.554338 | 0.677554 | -2.124458 |
| 23 | 6 | 0 | 2.359508 | 0.358044 | -0.949208 |
| 24 | 6 | 0 | 2.103059 | -1.107707 | -0.596462 |
| 25 | 6 | 0 | 2.470459 | 1.399951 | 0.135717 |
| 26 | 6 | 0 | 1.782449 | -2.002099 | -1.785143 |
| 27 | 1 | 0 | 1.399411 | -1.215866 | 0.292031 |
| 28 | 1 | 0 | 1.716945 | -3.047781 | -1.420633 |
| 29 | 1 | 0 | 2.598116 | -1.921557 | -2.582772 |
| 30 | 1 | 0 | 0.791050 | -1.685160 | -2.257579 |
| 31 | 6 | 0 | 2.658448 | 2.725521 | -0.262163 |
| 32 | 6 | 0 | 2.415153 | 1.097519 | 1.508958 |
| 33 | 6 | 0 | 2.794491 | 3.740485 | 0.679495 |
| 34 | 6 | 0 | 2.555332 | 2.113606 | 2.451426 |
| 35 | 6 | 0 | 2.744057 | 3.432980 | 2.045958 |
| 36 | 1 | 0 | 2.854247 | 4.226350 | 2.788626 |
| 37 | 1 | 0 | 2.521100 | 1.868658 | 3.514008 |
| 38 | 1 | 0 | 2.702168 | 2.942656 | -1.330981 |
| 39 | 1 | 0 | 2.936401 | 4.771245 | 0.360855 |
| 40 | 1 | 0 | 2.286369 | 0.082856 | 1.840662 |
| 41 | 35 | 0 | 3.916227 | -1.985682 | 0.302658 |

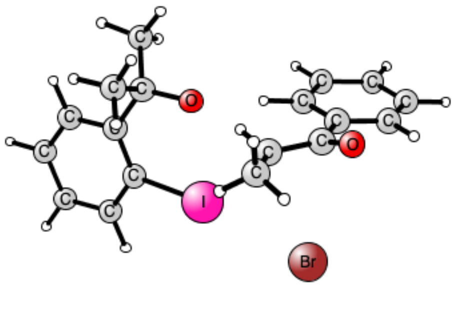

**$TS_{II'-IV}$**
**Thermal correction to Gibbs Free energy = 0.263793 Hartree**
**Electronic energy = -3719.39328463 Hartree**

| Center Number | Atomic Number | Atomic Type | Coordinates (Angstroms) X | Y | Z |
|---|---|---|---|---|---|
| 1 | 53 | 0 | -0.627230 | -1.072882 | -0.344581 |
| 2 | 6 | 0 | 1.476179 | -0.123879 | 1.617539 |
| 3 | 6 | 0 | 2.729638 | 0.518503 | 1.148309 |
| 4 | 8 | 0 | 3.764968 | 0.314995 | 1.787799 |
| 5 | 8 | 0 | -1.134924 | 1.291817 | 0.979099 |
| 6 | 6 | 0 | -2.471694 | 1.391925 | 1.191759 |
| 7 | 6 | 0 | -2.715841 | -0.481565 | -0.579641 |
| 8 | 6 | 0 | -3.290562 | 0.557234 | 0.152789 |
| 9 | 6 | 0 | -3.453900 | -1.241886 | -1.490391 |
| 10 | 6 | 0 | -4.661413 | 0.794022 | -0.057091 |
| 11 | 6 | 0 | -4.807310 | -0.973248 | -1.692401 |
| 12 | 6 | 0 | -5.413922 | 0.050591 | -0.964311 |
| 13 | 1 | 0 | -6.470112 | 0.271009 | -1.100871 |
| 14 | 1 | 0 | -5.151554 | 1.582121 | 0.506969 |
| 15 | 1 | 0 | -2.971568 | -2.043166 | -2.043611 |
| 16 | 1 | 0 | -5.377299 | -1.560079 | -2.408401 |
| 17 | 6 | 0 | 2.713116 | 1.509043 | 0.026339 |
| 18 | 35 | 0 | 2.588612 | -2.413097 | -0.377071 |
| 19 | 6 | 0 | -2.864456 | 2.895495 | 1.073419 |
| 20 | 6 | 0 | -2.862083 | 0.872325 | 2.607989 |
| 21 | 1 | 0 | -3.913696 | 3.108413 | 1.312189 |
| 22 | 1 | 0 | -2.234757 | 3.456666 | 1.770159 |
| 23 | 1 | 0 | -2.652647 | 3.250945 | 0.060709 |
| 24 | 1 | 0 | -2.318804 | 1.456465 | 3.359259 |
| 25 | 1 | 0 | -2.557841 | -0.174675 | 2.708219 |
| 26 | 1 | 0 | -3.937473 | 0.941673 | 2.816749 |
| 27 | 6 | 0 | 1.562980 | -1.124349 | 2.708189 |
| 28 | 1 | 0 | 2.462741 | -1.733077 | 2.632319 |
| 29 | 1 | 0 | 0.670081 | -1.752080 | 2.741829 |
| 30 | 1 | 0 | 1.600069 | -0.564759 | 3.660629 |
| 31 | 6 | 0 | 3.955886 | 1.867585 | -0.522551 |
| 32 | 6 | 0 | 1.542315 | 2.123011 | -0.449251 |
| 33 | 6 | 0 | 1.633454 | 3.082661 | -1.460951 |
| 34 | 6 | 0 | 4.034294 | 2.811825 | -1.540331 |
| 35 | 6 | 0 | 2.868553 | 3.424913 | -2.012131 |
| 36 | 1 | 0 | 4.845266 | 1.383946 | -0.133041 |
| 37 | 1 | 0 | 4.999464 | 3.070007 | -1.968981 |
| 38 | 1 | 0 | 2.925632 | 4.164303 | -2.807781 |
| 39 | 1 | 0 | 0.725633 | 3.559820 | -1.820811 |
| 40 | 1 | 0 | 0.561896 | 1.880950 | -0.040821 |
| 41 | 1 | 0 | 0.544968 | 0.442930 | 1.536589 |

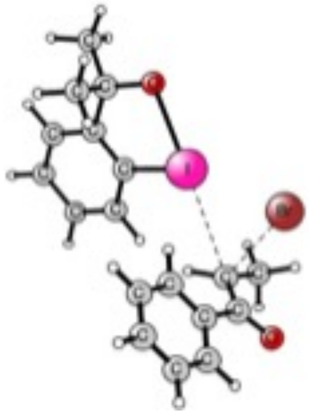

**TS´$_{III-IV}$**
**Thermal correction to Gibbs Free energy = 0.260107 Hartree**
**Electronic energy = -3719.38445940 Hartree**

| Center Number | Atomic Number | Atomic Type | Coordinates (Angstroms) X | Y | Z |
|---|---|---|---|---|---|
| 1 | 6 | 0 | -4.197921 | 2.484349 | 1.604310 |
| 2 | 6 | 0 | -4.510900 | 1.654409 | 0.526840 |
| 3 | 6 | 0 | -3.616040 | 0.679229 | 0.058790 |
| 4 | 6 | 0 | -2.963531 | 2.362170 | 2.241400 |
| 5 | 6 | 0 | -2.385160 | 0.589430 | 0.709930 |
| 6 | 6 | 0 | -2.048660 | 1.411180 | 1.784360 |
| 7 | 1 | 0 | -4.919681 | 3.222388 | 1.945980 |
| 8 | 1 | 0 | -5.476381 | 1.763388 | 0.041450 |
| 9 | 1 | 0 | -2.709841 | 2.997270 | 3.086240 |
| 10 | 1 | 0 | -1.084340 | 1.309861 | 2.273880 |
| 11 | 6 | 0 | -3.921789 | -0.258891 | -1.143660 |
| 12 | 6 | 0 | -5.430859 | -0.641812 | -1.158730 |
| 13 | 1 | 0 | -5.701509 | -1.122232 | -0.214490 |
| 14 | 1 | 0 | -5.583749 | -1.361952 | -1.967110 |
| 15 | 1 | 0 | -6.095740 | 0.214158 | -1.331470 |
| 16 | 6 | 0 | -3.585650 | 0.519529 | -2.450970 |
| 17 | 1 | 0 | -4.160850 | 1.448599 | -2.556960 |
| 18 | 1 | 0 | -3.795669 | -0.127831 | -3.309090 |
| 19 | 8 | 0 | -3.217629 | -1.420581 | -1.038880 |
| 20 | 53 | 0 | -0.888699 | -0.830639 | 0.013890 |
| 21 | 8 | 0 | 4.160501 | -0.139617 | 1.850000 |
| 22 | 6 | 0 | 3.208840 | 0.235973 | 1.154880 |
| 23 | 6 | 0 | 1.839590 | -0.051898 | 1.596960 |
| 24 | 6 | 0 | 3.440110 | 1.156933 | -0.010010 |
| 25 | 6 | 0 | 1.648841 | -0.898768 | 2.807480 |
| 26 | 6 | 0 | 4.695759 | 1.772834 | -0.114370 |
| 27 | 6 | 0 | 2.461930 | 1.435623 | -0.975120 |
| 28 | 6 | 0 | 2.732919 | 2.326553 | -2.013850 |
| 29 | 6 | 0 | 4.965179 | 2.663624 | -1.150530 |
| 30 | 6 | 0 | 3.981559 | 2.945803 | -2.102810 |
| 31 | 1 | 0 | 0.593991 | -1.101679 | 2.998000 |
| 32 | 1 | 0 | 2.073471 | -0.378108 | 3.682210 |

| | | | | | |
|---|---|---|---|---|---|
| 33 | 1 | 0 | 2.188461 | -1.842248 | 2.706220 |
| 34 | 1 | 0 | 1.110650 | 0.721902 | 1.389730 |
| 35 | 1 | 0 | 1.504430 | 0.928722 | -0.934890 |
| 36 | 1 | 0 | 1.970579 | 2.525842 | -2.762090 |
| 37 | 1 | 0 | 4.190378 | 3.637753 | -2.915110 |
| 38 | 1 | 0 | 5.941599 | 3.137014 | -1.219320 |
| 39 | 1 | 0 | 5.443560 | 1.530424 | 0.633590 |
| 40 | 35 | 0 | 2.248672 | -2.402388 | -0.414000 |
| 41 | 1 | 0 | -2.520090 | 0.768140 | -2.471220 |

---------------------------------------------------------------------------------

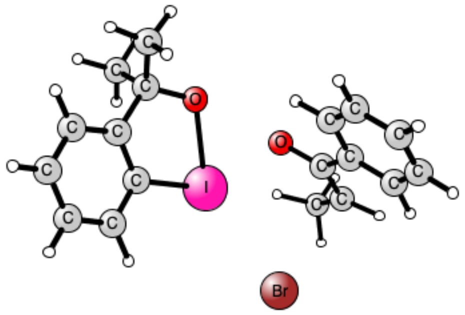


**TS$_{II-IV}$**
**Thermal correction to Gibbs Free energy = 0.262049 Hartree**
**Electronic energy = -3719.39411722 Hartree**

| Center Number | Atomic Number | Atomic Type | Coordinates (Angstroms) X | Y | Z |
|---|---|---|---|---|---|
| 1 | 53 | 0 | 0.534621 | -0.619891 | 0.166150 |
| 2 | 8 | 0 | -1.329249 | 0.516339 | 1.652210 |
| 3 | 8 | 0 | 1.516571 | 1.422369 | 0.945640 |
| 4 | 6 | 0 | 2.895031 | 1.354490 | 1.008800 |
| 5 | 6 | 0 | 2.584451 | -0.591221 | -0.564680 |
| 6 | 6 | 0 | 3.000881 | -1.540810 | -1.494810 |
| 7 | 6 | 0 | 3.445311 | 0.371610 | -0.052500 |
| 8 | 6 | 0 | 4.325851 | -1.528430 | -1.936920 |
| 9 | 6 | 0 | 4.775621 | 0.361900 | -0.495480 |
| 10 | 6 | 0 | 5.213281 | -0.575790 | -1.432390 |
| 11 | 6 | 0 | -2.470059 | 0.475719 | 1.063070 |
| 12 | 6 | 0 | -3.425099 | -0.479231 | 1.428340 |
| 13 | 1 | 0 | 6.247161 | -0.561010 | -1.768890 |
| 14 | 1 | 0 | 5.478711 | 1.094080 | -0.107120 |
| 15 | 1 | 0 | 2.299841 | -2.278621 | -1.876420 |
| 16 | 1 | 0 | 4.659601 | -2.257280 | -2.671190 |
| 17 | 35 | 0 | -2.239759 | -2.574451 | -0.595640 |
| 18 | 6 | 0 | -2.742219 | 1.499119 | 0.001690 |
| 19 | 6 | 0 | -1.775399 | 2.499899 | -0.201100 |
| 20 | 6 | 0 | -3.899039 | 1.515848 | -0.796590 |
| 21 | 6 | 0 | -1.971719 | 3.494909 | -1.158460 |
| 22 | 6 | 0 | -4.093769 | 2.512988 | -1.750550 |
| 23 | 6 | 0 | -3.133379 | 3.512029 | -1.933050 |

| | | | | | |
|---|---|---|---|---|---|
| 24 | 1 | 0 | -0.867999 | 2.463309 | 0.393690 |
| 25 | 1 | 0 | -1.210500 | 4.258189 | -1.302170 |
| 26 | 1 | 0 | -4.643139 | 0.732928 | -0.694180 |
| 27 | 1 | 0 | -4.993439 | 2.503818 | -2.361490 |
| 28 | 1 | 0 | -3.286120 | 4.288259 | -2.679350 |
| 29 | 6 | 0 | -3.211769 | -1.411611 | 2.571630 |
| 30 | 1 | 0 | -4.418679 | -0.434042 | 0.994710 |
| 31 | 1 | 0 | -3.683179 | -2.380301 | 2.387460 |
| 32 | 1 | 0 | -3.652759 | -0.995691 | 3.493830 |
| 33 | 1 | 0 | -2.144549 | -1.551711 | 2.749570 |
| 34 | 6 | 0 | 3.368971 | 0.878830 | 2.408210 |
| 35 | 1 | 0 | 4.462271 | 0.834350 | 2.490590 |
| 36 | 1 | 0 | 2.966491 | -0.117210 | 2.616670 |
| 37 | 1 | 0 | 2.982311 | 1.564890 | 3.168880 |
| 38 | 6 | 0 | 3.411711 | 2.790010 | 0.735270 |
| 39 | 1 | 0 | 3.113131 | 3.106340 | -0.267560 |
| 40 | 1 | 0 | 4.501701 | 2.870290 | 0.826970 |
| 41 | 1 | 0 | 2.955251 | 3.468230 | 1.461820 |

---------------------------------------------------------------------------------

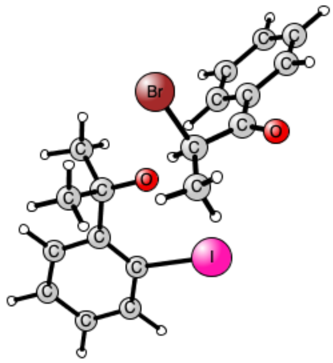


**INT-IV**
**Thermal correction to Gibbs Free energy = 0.261088 Hartree**
**Electronic energy = -3719.44383328 Hartree**

| Center Number | Atomic Number | Atomic Type | Coordinates (Angstroms) X | Y | Z |
|---|---|---|---|---|---|
| 1 | 6 | 0 | 5.281730 | 1.355658 | 1.038290 |
| 2 | 6 | 0 | 3.940028 | 1.682896 | 1.200424 |
| 3 | 6 | 0 | 2.879650 | 0.918096 | 0.663352 |
| 4 | 6 | 0 | 5.628525 | 0.216960 | 0.309598 |
| 5 | 6 | 0 | 3.273310 | -0.206154 | -0.057160 |
| 6 | 6 | 0 | 4.614466 | -0.562918 | -0.238973 |
| 7 | 1 | 0 | 6.051742 | 1.986307 | 1.477806 |
| 8 | 1 | 0 | 6.669322 | -0.063646 | 0.166674 |
| 9 | 1 | 0 | 3.689573 | 2.573703 | 1.768783 |
| 10 | 1 | 0 | 4.861654 | -1.451595 | -0.811155 |
| 11 | 6 | 0 | 1.386557 | 1.405951 | 0.922018 |
| 12 | 6 | 0 | 1.282117 | 2.842645 | 0.305768 |
| 13 | 1 | 0 | 2.009814 | 3.571618 | 0.687257 |

| | | | | | |
|---|---|---|---|---|---|
| 14 | 1 | 0 | 0.273962 | 3.218561 | 0.510394 |
| 15 | 1 | 0 | 1.401843 | 2.770003 | -0.780480 |
| 16 | 6 | 0 | 1.212582 | 1.509090 | 2.476197 |
| 17 | 1 | 0 | 1.920490 | 2.172523 | 2.991068 |
| 18 | 1 | 0 | 1.299754 | 0.506612 | 2.908916 |
| 19 | 1 | 0 | 0.197369 | 1.872831 | 2.667147 |
| 20 | 8 | 0 | 0.464260 | 0.593497 | 0.398907 |
| 21 | 53 | 0 | 1.907543 | -1.630346 | -1.047290 |
| 22 | 35 | 0 | -3.212373 | 2.315430 | -0.251299 |
| 23 | 8 | 0 | -3.504660 | -0.834565 | -1.972671 |
| 24 | 6 | 0 | -2.871753 | -0.428899 | -1.003894 |
| 25 | 6 | 0 | -4.010913 | -2.028690 | 0.509847 |
| 26 | 1 | 0 | -2.633814 | 1.263847 | -3.164540 |
| 27 | 6 | 0 | -2.021680 | 0.833499 | -1.126087 |
| 28 | 6 | 0 | -2.923838 | -1.162333 | 0.300728 |
| 29 | 6 | 0 | -4.116566 | -2.756147 | 1.690353 |
| 30 | 6 | 0 | -1.920238 | -1.047194 | 1.277432 |
| 31 | 1 | 0 | -4.760330 | -2.111738 | -0.271081 |
| 32 | 6 | 0 | -3.121304 | -2.639563 | 2.667644 |
| 33 | 6 | 0 | -2.030278 | -1.797064 | 2.452695 |
| 34 | 1 | 0 | - 4.967217 | -3.414173 | 1.851412 |
| 35 | 1 | 0 | -1.035035 | -0.417742 | 1.115166 |
| 36 | 6 | 0 | -1.731124 | 1.233541 | -2.550953 |
| 37 | 1 | 0 | -3.197284 | -3.210490 | 3.590644 |
| 38 | 1 | 0 | -1.247576 | -1.718448 | 3.202797 |
| 39 | 1 | 0 | -1.218865 | 2.197459 | -2.581015 |
| 40 | 1 | 0 | -1.113154 | 0.855824 | -0.490920 |
| 41 | 1 | 0 | -1.053373 | 0.479160 | -2.970602 |

---------------------------------------------------------------------------

**Reaction coordinates for the silyl transfer process:**

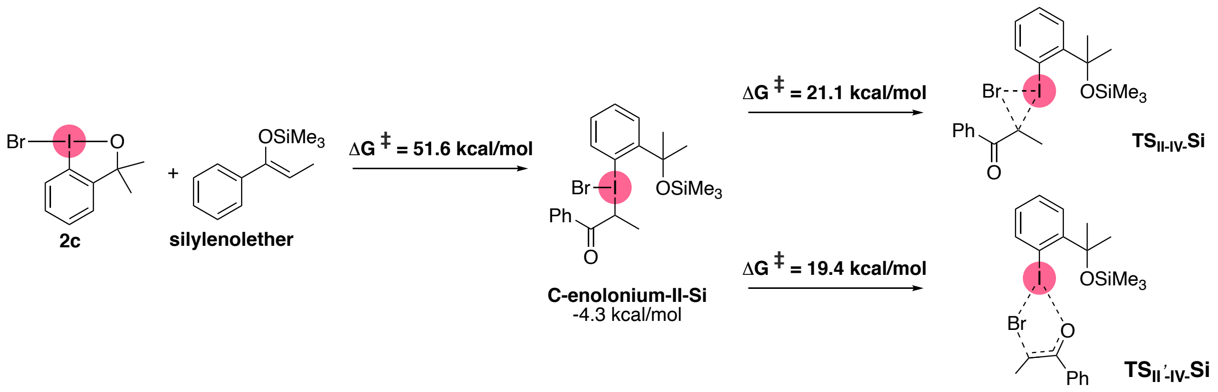


**Scheme S6.** Reaction profile in which the silyl group participates.

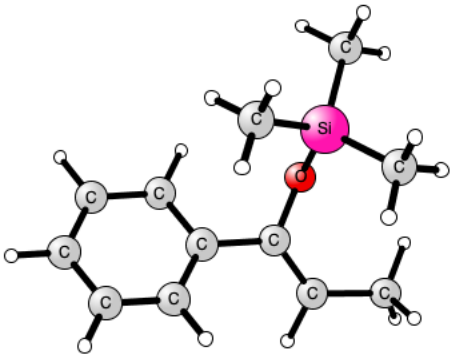


**Silylenolether**

**Thermal correction to Gibbs Free energy = 0.222949 Hartree**

**Electronic energy = -832.696767830 Hartree**

| Center Number | Atomic Number | Atomic Type | Coordinates (Angstroms) X | Y | Z |
|---|---|---|---|---|---|
| 1 | 6 | 0 | 2.999898 | -1.297956 | -0.827157 |
| 2 | 6 | 0 | 3.923823 | -0.778425 | 0.082507 |
| 3 | 6 | 0 | 3.572248 | 0.326107 | 0.862796 |
| 4 | 6 | 0 | 2.311418 | 0.907171 | 0.732815 |
| 5 | 6 | 0 | 1.376850 | 0.401411 | -0.188045 |
| 6 | 6 | 0 | 1.738667 | -0.717140 | -0.958732 |
| 7 | 6 | 0 | 0.042924 | 1.033676 | -0.369412 |
| 8 | 6 | 0 | -0.168330 | 2.356743 | -0.242500 |
| 9 | 6 | 0 | -1.468301 | 3.070292 | -0.459553 |
| 10 | 8 | 0 | -0.959591 | 0.185468 | -0.796367 |
| 11 | 14 | 0 | -1.924304 | -0.824268 | 0.174839 |
| 12 | 6 | 0 | -3.029444 | 0.227591 | 1.278988 |
| 13 | 6 | 0 | -2.933959 | -1.802027 | -1.071031 |
| 14 | 6 | 0 | -0.863294 | -1.959687 | 1.238639 |
| 15 | 1 | 0 | -2.289503 | -2.406717 | -1.717520 |
| 16 | 1 | 0 | -1.348279 | 3.885434 | -1.183962 |
| 17 | 1 | 0 | -2.242088 | 2.395052 | -0.828744 |
| 18 | 1 | 0 | 2.039894 | 1.746971 | 1.364759 |
| 19 | 1 | 0 | -3.526827 | -1.138530 | -1.709109 |
| 20 | 1 | 0 | -0.207206 | -2.589037 | 0.629869 |
| 21 | 1 | 0 | -3.625570 | -2.479892 | -0.558791 |
| 22 | 1 | 0 | 4.276610 | 0.728978 | 1.584909 |
| 23 | 1 | 0 | -1.832538 | 3.530745 | 0.468311 |
| 24 | 1 | 0 | 0.691613 | 2.970974 | 0.009323 |
| 25 | 1 | 0 | -1.507593 | -2.617004 | 1.834095 |
| 26 | 1 | 0 | -0.234317 | -1.393101 | 1.932823 |
| 27 | 1 | 0 | 4.904112 | -1.233473 | 0.188245 |
| 28 | 1 | 0 | 1.026498 | -1.119879 | -1.670818 |
| 29 | 1 | 0 | -3.708789 | 0.852891 | 0.691141 |
| 30 | 1 | 0 | -2.438091 | 0.886203 | 1.923876 |
| 31 | 1 | 0 | -3.637910 | -0.413281 | 1.927318 |
| 32 | 1 | 0 | 3.261967 | -2.156860 | -1.438431 |

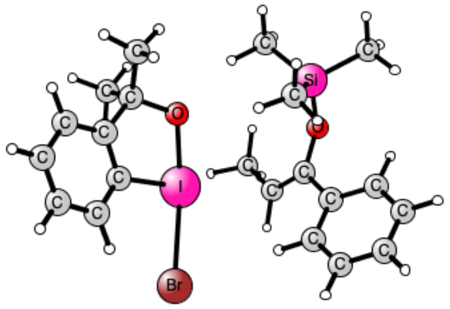


**C-enolonium-II-Si**

**Thermal correction to Gibbs Free energy = 0.367387 Hartree**

**Electronic energy = -4128.55906819 Hartree**

| Center Number | Atomic Number | Atomic Type | Coordinates (Angstroms) X | Y | Z |
|---|---|---|---|---|---|
| 1 | 6 | 0 | -3.059253 | -3.180956 | -1.336669 |
| 2 | 6 | 0 | -4.289293 | -2.947735 | -1.956729 |
| 3 | 6 | 0 | -5.016641 | -1.803264 | -1.627109 |
| 4 | 6 | 0 | -4.518061 | -0.898995 | -0.689639 |
| 5 | 6 | 0 | -3.281811 | -1.121706 | -0.058919 |
| 6 | 6 | 0 | -2.558972 | -2.278947 | -0.399769 |
| 7 | 6 | 0 | -2.791780 | -0.157917 | 0.963011 |
| 8 | 6 | 0 | -2.015830 | -0.512477 | 2.012661 |
| 9 | 6 | 0 | -1.611359 | 0.397582 | 3.136471 |
| 10 | 8 | 0 | -3.288569 | 1.114134 | 0.873011 |
| 11 | 14 | 0 | -3.014467 | 2.401434 | -0.198149 |
| 12 | 6 | 0 | -4.719587 | 3.104005 | -0.576069 |
| 13 | 8 | 0 | 1.032852 | 1.742179 | -0.074249 |
| 14 | 6 | 0 | 2.333163 | 2.326908 | -0.036239 |
| 15 | 6 | 0 | 2.226214 | 3.566918 | -0.938209 |
| 16 | 53 | 0 | 1.063940 | -0.352061 | 0.355091 |
| 17 | 6 | 0 | 3.053210 | -0.042753 | -0.485159 |
| 18 | 6 | 0 | 3.890519 | -1.072243 | -0.869609 |
| 19 | 6 | 0 | 5.135720 | -0.713235 | -1.396979 |
| 20 | 6 | 0 | 5.488181 | 0.632535 | -1.506459 |
| 21 | 6 | 0 | 4.607852 | 1.634456 | -1.094209 |
| 22 | 6 | 0 | 3.349582 | 1.310287 | -0.570079 |
| 23 | 35 | 0 | 1.473007 | -3.203281 | 0.673931 |
| 24 | 6 | 0 | 2.708563 | 2.724498 | 1.405211 |
| 25 | 6 | 0 | -1.971226 | 3.676993 | 0.712231 |
| 26 | 6 | 0 | -2.128708 | 1.840503 | -1.760749 |
| 27 | 1 | 0 | 2.730382 | 1.847788 | 2.059921 |
| 28 | 1 | 0 | 3.594398 | -2.110523 | -0.762069 |
| 29 | 1 | 0 | 1.967074 | 3.427759 | 1.794911 |
| 30 | 1 | 0 | 3.695794 | 3.195677 | 1.442491 |
| 31 | 1 | 0 | 5.821069 | -1.490775 | -1.719479 |
| 32 | 1 | 0 | 6.455921 | 0.906544 | -1.915429 |
| 33 | 1 | 0 | 4.899143 | 2.676846 | -1.180159 |
| 34 | 1 | 0 | 1.429335 | 4.214259 | -0.564069 |
| 35 | 1 | 0 | 3.159145 | 4.139397 | -0.936339 |
| 36 | 1 | 0 | 1.987854 | 3.274358 | -1.963199 |

| | | | | | |
|---|---|---|---|---|---|
| 37 | 1 | 0 | -0.969446 | 3.279311 | 0.904501 |
| 38 | 1 | 0 | -0.549619 | 0.286181 | 3.387011 |
| 39 | 1 | 0 | -1.805138 | 1.444192 | 2.895281 |
| 40 | 1 | 0 | -5.096360 | -0.022224 | -0.419929 |
| 41 | 1 | 0 | -2.425056 | 3.960833 | 1.667671 |
| 42 | 1 | 0 | -1.089828 | 1.569572 | -1.546999 |
| 43 | 1 | 0 | -1.860195 | 4.588172 | 0.112121 |
| 44 | 1 | 0 | -5.978461 | -1.614563 | -2.096029 |
| 45 | 1 | 0 | -2.171660 | 0.155413 | 4.049671 |
| 46 | 1 | 0 | -1.764041 | -1.564738 | 2.111061 |
| 47 | 1 | 0 | -2.107387 | 2.666033 | -2.482679 |
| 48 | 1 | 0 | -2.625109 | 0.990763 | -2.237879 |
| 49 | 1 | 0 | -4.674593 | -3.650075 | -2.690239 |
| 50 | 1 | 0 | -1.594292 | -2.477498 | 0.055901 |
| 51 | 1 | 0 | -5.263936 | 3.327666 | 0.347511 |
| 52 | 1 | 0 | -5.325827 | 2.405006 | -1.161339 |
| 53 | 1 | 0 | -4.639606 | 4.034865 | -1.149109 |
| 54 | 1 | 0 | -2.478254 | -4.063507 | -1.588339 |

---------------------------------------------------------------------------

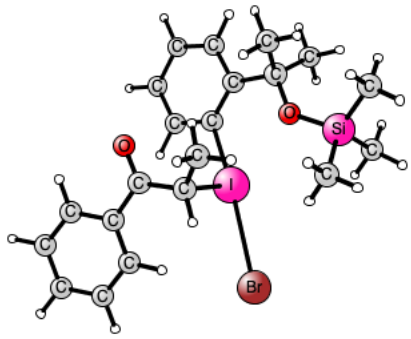


**C-enolonium-II-Si**
**Thermal correction to Gibbs Free energy = 0.370520Hartree**
**Electronic energy = -4128.57805182 Hartree**

| Center Number | Atomic Number | Atomic Type | Coordinates (Angstroms) X | Y | Z |
|---|---|---|---|---|---|
| 1 | 35 | 0 | -1.531937 | -3.456609 | -0.504959 |
| 2 | 1 | 0 | 3.863357 | 1.083022 | -1.885278 |
| 3 | 53 | 0 | -0.135913 | -0.275979 | -0.670887 |
| 4 | 6 | 0 | 4.218333 | 1.468191 | -0.925693 |
| 5 | 1 | 0 | -1.331838 | 2.295601 | -1.475981 |
| 6 | 1 | 0 | 5.106769 | 0.906486 | -0.626408 |
| 7 | 6 | 0 | -0.358461 | 2.662871 | -1.173556 |
| 8 | 1 | 0 | 4.519671 | 2.508215 | -1.065986 |
| 9 | 6 | 0 | 0.616598 | 1.776739 | -0.692056 |
| 10 | 8 | 0 | 2.730077 | -0.023500 | 0.305174 |
| 11 | 6 | 0 | -0.079093 | 4.021169 | -1.268197 |
| 12 | 6 | 0 | 3.116584 | 1.335408 | 0.148496 |
| 13 | 6 | 0 | 1.903681 | 2.185974 | -0.314262 |
| 14 | 1 | 0 | -0.835676 | 4.704808 | -1.638563 |
| 15 | 6 | 0 | 1.180274 | 4.475802 | -0.881933 |
| 16 | 6 | 0 | 2.134933 | 3.574921 | -0.425309 |

| | | | | | |
|---|---|---|---|---|---|
| 17 | 6 | 0 | 3.612414 | 1.845199 | 1.518656 |
| 18 | 1 | 0 | 1.428063 | 5.530653 | -0.943791 |
| 19 | 1 | 0 | 3.109480 | 3.961560 | -0.150592 |
| 20 | 1 | 0 | 4.481668 | 1.261524 | 1.829069 |
| 21 | 1 | 0 | 3.906687 | 2.896330 | 1.494427 |
| 22 | 1 | 0 | 2.826220 | 1.722455 | 2.268364 |
| 23 | 1 | 0 | -4.119286 | 2.817369 | 1.073665 |
| 24 | 1 | 0 | -6.450762 | 2.573035 | 0.237676 |
| 25 | 1 | 0 | 0.521653 | -0.798415 | 2.461026 |
| 26 | 6 | 0 | -4.481563 | 1.859609 | 0.717099 |
| 27 | 6 | 0 | -5.782589 | 1.717655 | 0.249486 |
| 28 | 8 | 0 | -1.863767 | 2.092190 | 1.643853 |
| 29 | 1 | 0 | 0.259886 | 0.974991 | 2.428430 |
| 30 | 6 | 0 | -0.221899 | -0.002396 | 2.473166 |
| 31 | 6 | 0 | -2.235655 | 0.987934 | 1.263371 |
| 32 | 6 | 0 | -1.240709 | -0.170418 | 1.381993 |
| 33 | 6 | 0 | -3.606403 | 0.755988 | 0.739193 |
| 34 | 6 | 0 | -6.228470 | 0.471163 | -0.205434 |
| 35 | 1 | 0 | -7.244862 | 0.359142 | -0.570870 |
| 36 | 1 | 0 | -0.768596 | -0.057443 | 3.424254 |
| 37 | 6 | 0 | -4.062255 | -0.493191 | 0.281120 |
| 38 | 6 | 0 | -5.367676 | -0.628626 | -0.190450 |
| 39 | 1 | 0 | -3.414208 | -1.363686 | 0.270896 |
| 40 | 1 | 0 | -5.711906 | -1.595015 | -0.545089 |
| 41 | 1 | 0 | -1.670070 | -1.169215 | 1.319967 |
| 42 | 14 | 0 | 3.621565 | -1.467521 | 0.314349 |
| 43 | 6 | 0 | 3.939760 | -2.043849 | -1.452840 |
| 44 | 1 | 0 | 4.622461 | -1.380445 | -1.992506 |
| 45 | 1 | 0 | 4.386465 | -3.044999 | -1.444637 |
| 46 | 1 | 0 | 3.005541 | -2.102140 | -2.022050 |
| 47 | 6 | 0 | 5.251463 | -1.342623 | 1.260193 |
| 48 | 1 | 0 | 5.720064 | -2.334400 | 1.266840 |
| 49 | 1 | 0 | 5.970964 | -0.647460 | 0.817306 |
| 50 | 1 | 0 | 5.091900 | -1.049893 | 2.303172 |
| 51 | 6 | 0 | 2.523530 | -2.709319 | 1.202098 |
| 52 | 1 | 0 | 1.555316 | -2.839814 | 0.709443 |
| 53 | 1 | 0 | 3.019600 | -3.686788 | 1.223303 |
| 54 | 1 | 0 | 2.343468 | -2.410719 | 2.240554 |

---------------------------------------------------------------------------------------------

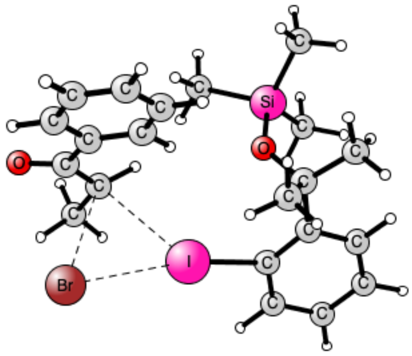


**TS$_{II-IV}$-Si**

**Thermal correction to Gibbs Free energy = 0.369736 Hartree**
**Electronic energy = -4128.53683553 Hartree**

| Center Number | Atomic Number | Atomic Type | Coordinates (Angstroms) X | Y | Z |
|---|---|---|---|---|---|
| 1 | 6 | 0 | -4.864937 | -2.244883 | -0.311725 |
| 2 | 6 | 0 | -4.375271 | -1.025277 | -0.778337 |
| 3 | 6 | 0 | -3.011195 | -0.679803 | -0.746907 |
| 4 | 6 | 0 | -3.990221 | -3.184729 | 0.222677 |
| 5 | 6 | 0 | -2.151330 | -1.672982 | -0.232613 |
| 6 | 6 | 0 | -2.627461 | -2.893337 | 0.259096 |
| 7 | 1 | 0 | -5.930248 | -2.447253 | -0.362036 |
| 8 | 1 | 0 | -5.089386 | -0.316060 | -1.175183 |
| 9 | 1 | 0 | -4.349678 | -4.135892 | 0.602347 |
| 10 | 1 | 0 | -1.935196 | -3.624573 | 0.660606 |
| 11 | 6 | 0 | -2.571417 | 0.726835 | -1.232697 |
| 12 | 6 | 0 | -1.649483 | 0.640495 | -2.463745 |
| 13 | 1 | 0 | -0.715533 | 0.124764 | -2.253665 |
| 14 | 1 | 0 | -1.410643 | 1.652218 | -2.804360 |
| 15 | 1 | 0 | -2.159907 | 0.115530 | -3.276807 |
| 16 | 6 | 0 | -3.762566 | 1.609792 | -1.649214 |
| 17 | 1 | 0 | -4.275082 | 1.196625 | -2.521817 |
| 18 | 1 | 0 | -3.377919 | 2.593299 | -1.927095 |
| 19 | 1 | 0 | -4.498222 | 1.742331 | -0.852900 |
| 20 | 8 | 0 | -1.828121 | 1.364213 | -0.171030 |
| 21 | 53 | 0 | 0.036163 | -1.614081 | -0.219302 |
| 22 | 8 | 0 | 4.067300 | 0.543419 | 1.920987 |
| 23 | 6 | 0 | 3.054384 | 0.660683 | 1.233521 |
| 24 | 6 | 0 | 1.856376 | -0.072339 | 1.782864 |
| 25 | 6 | 0 | 2.951536 | 1.603111 | 0.101513 |
| 26 | 6 | 0 | 2.029988 | -1.083728 | 2.823456 |
| 27 | 6 | 0 | 4.140131 | 2.227384 | -0.324336 |
| 28 | 6 | 0 | 1.735328 | 1.925366 | -0.528421 |
| 29 | 6 | 0 | 1.716514 | 2.857120 | -1.564937 |
| 30 | 6 | 0 | 4.115059 | 3.148914 | -1.363592 |
| 31 | 6 | 0 | 2.901054 | 3.466750 | -1.985425 |
| 32 | 1 | 0 | 1.155940 | -1.726503 | 2.928882 |
| 33 | 1 | 0 | 2.086940 | -0.497402 | 3.762959 |
| 34 | 1 | 0 | 2.958105 | -1.643913 | 2.724560 |
| 35 | 1 | 0 | 0.897998 | 0.435052 | 1.732181 |
| 36 | 1 | 0 | 0.800496 | 1.464612 | -0.230674 |
| 37 | 1 | 0 | 0.775321 | 3.106216 | -2.043814 |
| 38 | 1 | 0 | 2.880439 | 4.188362 | -2.796421 |
| 39 | 1 | 0 | 5.035313 | 3.621617 | -1.691498 |
| 40 | 1 | 0 | 5.070334 | 1.974659 | 0.172348 |
| 41 | 35 | 0 | 3.497786 | -2.144644 | -0.442670 |
| 42 | 14 | 0 | -2.408004 | 2.239762 | 1.162780 |
| 43 | 6 | 0 | -3.814269 | 1.353460 | 2.056142 |
| 44 | 1 | 0 | -4.729912 | 1.277844 | 1.462556 |
| 45 | 1 | 0 | -3.519967 | 0.340282 | 2.348946 |
| 46 | 1 | 0 | -4.059339 | 1.906498 | 2.970836 |

| | | | | | |
|---|---|---|---|---|---|
| 47 | 6 | 0 | -2.926017 | 3.987848 | 0.672607 |
| 48 | 1 | 0 | -3.093367 | 4.584048 | 1.577498 |
| 49 | 1 | 0 | -2.139521 | 4.482677 | 0.092196 |
| 50 | 1 | 0 | -3.846966 | 4.013756 | 0.083755 |
| 51 | 6 | 0 | -0.943578 | 2.371650 | 2.339245 |
| 52 | 1 | 0 | -0.701739 | 1.404455 | 2.793105 |
| 53 | 1 | 0 | -0.050687 | 2.769906 | 1.846756 |
| 54 | 1 | 0 | -1.193834 | 3.053995 | 3.159605 |

---------------------------------------------------------------------------------

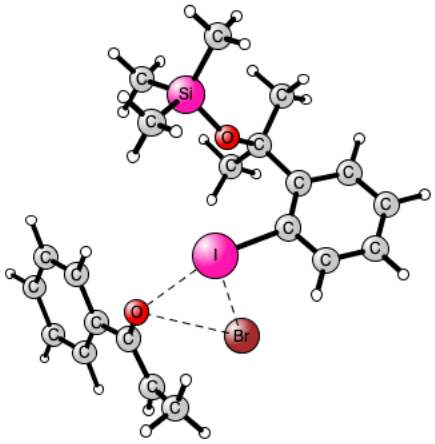


**TSII'-IV-Si**
**Thermal correction to Gibbs Free energy = 0.368305 Hartree**
**Electronic energy = -4128.53810044 Hartree**

| Center Number | Atomic Number | Atomic Type | Coordinates (Angstroms) X | Y | Z |
|---|---|---|---|---|---|
| 1 | 6 | 0 | 3.286842 | -3.659778 | -1.024350 |
| 2 | 6 | 0 | 3.403260 | -2.299632 | -1.309674 |
| 3 | 6 | 0 | 2.557573 | -1.323392 | -0.751941 |
| 4 | 6 | 0 | 2.300448 | -4.100011 | -0.147794 |
| 5 | 6 | 0 | 1.564954 | -1.800959 | 0.131794 |
| 6 | 6 | 0 | 1.438369 | -3.164810 | 0.422878 |
| 7 | 1 | 0 | 3.970787 | -4.364644 | -1.487245 |
| 8 | 1 | 0 | 2.192354 | -5.153254 | 0.092426 |
| 9 | 1 | 0 | 4.186963 | -1.993468 | -1.990091 |
| 10 | 1 | 0 | 0.661318 | -3.498837 | 1.100920 |
| 11 | 6 | 0 | 2.756161 | 0.164535 | -1.139437 |
| 12 | 6 | 0 | 1.579348 | 0.637281 | -2.018919 |
| 13 | 1 | 0 | 1.522457 | 0.030688 | -2.928139 |
| 14 | 1 | 0 | 1.726699 | 1.680105 | -2.313597 |
| 15 | 1 | 0 | 0.628581 | 0.560037 | -1.491341 |
| 16 | 6 | 0 | 4.061877 | 0.399299 | -1.919227 |
| 17 | 1 | 0 | 4.055459 | -0.107240 | -2.887623 |
| 18 | 1 | 0 | 4.936608 | 0.070274 | -1.352568 |
| 19 | 1 | 0 | 4.164278 | 1.468412 | -2.121707 |
| 20 | 8 | 0 | 2.792699 | 0.899698 | 0.088868 |
| 21 | 53 | 0 | 0.087601 | -0.587788 | 1.151694 |
| 22 | 35 | 0 | -2.065132 | -2.091511 | -1.128893 |
| 23 | 8 | 0 | -2.281234 | 0.412362 | 1.753289 |

| | | | | | |
|---|---|---|---|---|---|
| 24 | 6 | 0 | -3.250459 | 0.311111 | 0.976396 |
| 25 | 6 | 0 | -4.033018 | -0.932419 | 1.033380 |
| 26 | 6 | 0 | -3.609955 | 1.391805 | 0.028381 |
| 27 | 6 | 0 | -3.886904 | -1.876355 | 2.138507 |
| 28 | 1 | 0 | -4.841133 | -1.091047 | 0.328194 |
| 29 | 1 | 0 | -4.121978 | -2.899521 | 1.837318 |
| 30 | 1 | 0 | -4.659715 | -1.593947 | 2.880344 |
| 31 | 1 | 0 | -2.916944 | -1.794406 | 2.629167 |
| 32 | 6 | 0 | -2.780041 | 2.528794 | -0.009706 |
| 33 | 6 | 0 | -4.740613 | 1.341837 | -0.810084 |
| 34 | 6 | 0 | -3.064611 | 3.581878 | -0.871329 |
| 35 | 6 | 0 | -5.024108 | 2.401392 | -1.669221 |
| 36 | 6 | 0 | -4.187870 | 3.520160 | -1.704478 |
| 37 | 1 | 0 | -4.411129 | 4.342880 | -2.376855 |
| 38 | 1 | 0 | -5.898039 | 2.353467 | -2.310786 |
| 39 | 1 | 0 | -1.917043 | 2.565558 | 0.645330 |
| 40 | 1 | 0 | -2.415781 | 4.451703 | -0.895935 |
| 41 | 1 | 0 | -5.410126 | 0.489450 | -0.802058 |
| 42 | 14 | 0 | 3.332357 | 2.425040 | 0.546127 |
| 43 | 6 | 0 | 2.613597 | 2.662362 | 2.269530 |
| 44 | 1 | 0 | 2.918325 | 3.629466 | 2.685428 |
| 45 | 1 | 0 | 1.519559 | 2.630666 | 2.250008 |
| 46 | 1 | 0 | 2.959341 | 1.879968 | 2.953648 |
| 47 | 6 | 0 | 5.217387 | 2.493957 | 0.650074 |
| 48 | 1 | 0 | 5.697749 | 2.430313 | -0.331009 |
| 49 | 1 | 0 | 5.529075 | 3.438723 | 1.111516 |
| 50 | 1 | 0 | 5.607381 | 1.678791 | 1.269565 |
| 51 | 6 | 0 | 2.708746 | 3.794149 | -0.596548 |
| 52 | 1 | 0 | 1.616184 | 3.787200 | -0.671487 |
| 53 | 1 | 0 | 3.007912 | 4.769599 | -0.194403 |
| 54 | 1 | 0 | 3.117004 | 3.720493 | -1.609974 |

---------------------------------------------------------------------

## References


1) G. C. Geary, E. G. Hope, K. Singh, A. M. Stuart, *Chem. Commun.,* **2013**, *49*, 9263-0265.
2) Y. Peng, J. Liu, C. Qi, G. Yuan, J. Li, H. Jiang, *Chem. Commun*., **2017**, *53*, 2665-2668.
3) Gaussian 16, Revision C.01, M. J. Frisch, G. W. Trucks, H. B. Schlegel, G. E. Scuseria, M. A. Robb, J. R. Cheeseman, G. Scalmani, V. Barone, G. A. Petersson, H. Nakatsuji, X. Li, M. Caricato, A. V. Marenich, J. Bloino, B. G. Janesko, R. Gomperts, B. Mennucci, H. P. Hratchian, J. V. Ortiz, A. F. Izmaylov, J. L. Sonnenberg, D. Williams-Young, F. Ding, F. Lipparini, F. Egidi, J. Goings, B. Peng, A. Petrone, T. Henderson, D. Ranasinghe, V. G. Zakrzewski, J. Gao, N. Rega, G. Zheng, W. Liang, M. Hada, M. Ehara, K. Toyota, R. Fukuda, J. Hasegawa, M. Ishida, T. Nakajima, Y. Honda, O. Kitao, H. Nakai, T. Vreven, K. Throssell, J. A. Montgomery, Jr., J. E. Peralta, F. Ogliaro, M. J. Bearpark, J. J. Heyd, E. N. Brothers, K. N. Kudin, V. N. Staroverov, T. A. Keith, R. Kobayashi, J. Normand, K. Raghavachari, A. P. Rendell, J. C. Burant, S. S. Iyengar, J. Tomasi, M. Cossi, J. M. Millam, M. Klene, C. Adamo, R. Cammi, J. W. Ochterski, R. L. Martin, K. Morokuma, O. Farkas, J. B. Foresman, and D. J. Fox, Gaussian, Inc., Wallingford CT, **2016**.
4) A. D. Becke, *J. Chem. Phys.,* **1993**, *98*, 5648-5652.
5) Y. Zhao, D. G, Truhlar, *Theor. Chem. Acc.*, 2**008**, *120*, 215-241.
6) F. Weigend, *Phys. Chem. Chem. Phys.,* **2006**, *8*, 1057-1065.
7) (a) E. Cancès, B. Mennucci; J. Tomassi, J. *Chem. Phys.,* **1997**, *107*, 3032-3041. (b) M. Cossi, V. Barone, B. Mennucci, J. Tomasi, *Chem. Phys. Lett.,* **1998**, *286*, 253-260. (c) J. Tomasi, B. Mennucci, E. Cancès, *J. Mol. Struct.,* **1999**, *464*, 211-226.
8) C. González, H. B. Schlegel, *J. Phys. Chem.* **1990**, *94*, 5523-5527.
